# The waste-environmental-economic nexus facilitates city-specific cost-effective transition for China’s municipal solid waste treatment

**DOI:** 10.1016/j.xinn.2026.101315

**Published:** 2026-02-12

**Authors:** Hao Li, Xiaolong Lu, Fang Liu, Zhe Li, Xianmei Liu, Bin Lu, Shijun Ma, Baojing Gu, Chuanbin Zhou, Zhaohua Wang

**Affiliations:** 1 School of Economics, Beijing Institute of Technology, Beijing 100081, China; 2 Digital Economy and Policy Intelligentization Key Laboratory of Ministry of Industry and Information Technology, Beijing 100081, China; 3 Center for Sustainable Development and Smart Decision, Beijing Institute of Technology, Beijing 100081, China; 4 College of Economics and Management, College of Environment and Resources, College of Carbon Neutral Zhejiang A&F University, Hangzhou 311300, China; 5 Zhejiang Province Key Think Tank: Institute of Ecological Civilization and Institute of Carbon Neutrality, Zhejiang A&F University, Hangzhou 311300, China; 6 Zhejiang Key Laboratory of Ecological Environmental Damage Control and Value Transformation Zhejiang A&F University, Zhejiang 311300, China; 7 School of Economics, Hebei University of Economics and Business, Shijiazhuang 050062, P.R. China; 8 Bartlett School of Sustainable Construction, University College London, London WC1H 9BT, UK; 9 College of Environmental and Resource Sciences, Zhejiang University, Hangzhou 310058, China; 10 State Key Laboratory of Regional and Urban Ecology, Research Center for Eco-Environmental Sciences, Chinese Academy of Sciences, Beijing 100083, China

**Keywords:** waste-environment-economy nexus, municipal solid waste, city-specific strategies, cost-benefit analysis

## Abstract

The growing challenge of municipal solid waste (MSW) generation requires systematic solutions based on the waste-environment-economy (WEE) nexus. Here, we develop an integrated assessment framework that combines bottom-up economic costing, life cycle monetization, and scenario analysis to evaluate nine MSW treatment strategies in 352 Chinese cities. Our approach integrates 18 environmental impact categories with key economic performance indicators. The results demonstrate that waste-to-material strategies could reduce China’s total environmental costs of MSW treatment by 36.9%–78.3%, primarily driven by substantial reductions in greenhouse gas emissions and freshwater ecotoxicity. However, the WEE nexus highlights notable regional trade-offs between economic and environmental outcomes, largely due to variations in waste composition and local infrastructure. By using a composite benefit index to identify optimal MSW management pathways, we find that integrated strategies, combining incineration with material recycling (either alone or combined with bioconversion), prove cost-effective in over 84% of the studied cities. In contrast, a bioconversion-dominated strategy is most effective for the remaining cities. These findings provide spatially targeted guidance for facilitating city-specific transitions toward feasible MSW management in China.

## Public summary

• A waste-environmental-economic nexus framework is developed to identify optimal strategies.
• Adopting waste-to-material strategies could reduce total environmental costs by 36.9%–78.3%.
• Significant trade-offs exist between economic and environmental outcomes across regions.
• Integrated WtE and WtM strategies are expected to yield superior outcomes for >84% of cities.

## Graphical abstract

**Figure:**
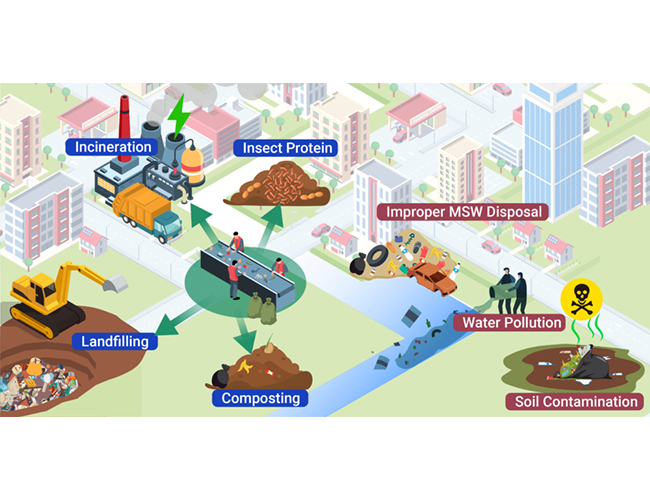

## Introduction

The growing generation of municipal solid waste (MSW) is a significant global challenge, driven by population growth, rapid urbanization, and changing consumption patterns. Poor waste management threatens the environment, public health, and climate stability.^1^^,^^2^ China, the world’s largest MSW producer, contributes over 10% of global waste and faces an acute crisis. Projections indicate a potential doubling of its waste volume by 2060, intensifying pressures on already strained management systems.^3^ Despite significant policy efforts, many Chinese cities still struggle with waste overflow, inadequate treatment capacity, and environmental damage.^4^^,^^5^ Addressing these challenges requires a shift toward integrated, spatially tailored strategies that ensure safe disposal and improve resource recovery. Within this context, we propose a waste-environmental-economic (WEE) nexus framework to guide city-specific, cost-effective transitions of MSW treatment. Effective MSW management is essential not just for waste disposal but also for alleviating environmental pressures and supporting climate mitigation. In response, China launched a national source-separation initiative in 2019, aiming to diversify its treatment system by 2025. Key targets include treating 65% of MSW via incineration and achieving 60% resource utilization efficiency.^6^ Waste-to-energy (WtE) technologies, especially incineration, are widely used for their ability to reduce waste volume, conserve land, and generate energy.^7^^,^^8^ However, WtE’s long-term sustainability is uncertain due to its reliance on subsidies, uneven distribution of facilities, and low energy recovery efficiency.^9^^,^^10^ These limitations underscore the need to critically evaluate alternative pathways and their inherent trade-offs.

Extensive academic research on MSW treatment technologies has evolved from detailed technical analyses to broader system simulations, and from single-dimensional to multidimensional assessments. Early studies predominantly focused on the environmental, resource, or economic performance of individual treatment technologies.^11^^,^^12^ For instance, numerous life cycle assessment studies have precisely quantified greenhouse gas (GHG) emissions and resource consumption associated with technologies such as incineration, landfilling, or anaerobic digestion, establishing a methodological foundation for uncovering the micro-level impacts of technological units.^13^^,^^14^^,^^15^ However, waste management is a complex system involving classification, collection, transportation, treatment, and disposal. Improving one component does not guarantee overall system improvement. Consequently, research has shifted to macro and systemic approaches, using methods such as material flow analysis, techno-economic assessment, and integrated models to optimize long-term waste management strategies at regional or national levels.^16^^,^^17^ While these macro-scale models facilitate the evaluation of synergies between technology portfolios and emission reduction targets, they often sacrifice spatial resolution for model operability. Their conclusions are generally applicable at national or large regional scales, failing to capture the substantial heterogeneity among cities, thereby limiting their usefulness for local governance.^18^^,^^19^

In terms of assessment dimensions, research has evolved from isolated environmental or economic analyses to integrated frameworks that address multiple dimensions. Monetizing environmental externalities such as carbon emissions and pollution, and integrating them with economic costs, is a crucial step in supporting decision-making.^20^^,^^21^^,^^22^^,^^23^ This approach enables comprehensive net-benefit comparisons across different strategies through a unified monetary metric.^24^^,^^25^ However, significant gaps persist in existing integrated studies. On one hand, most monetary assessments at national or regional levels rely on globally averaged parameters, failing to reflect the considerable variations among Chinese cities in waste composition, treatment costs, environmental carrying capacity, and other localized factors.^26^ On the other hand, while some city-level studies include economic considerations, their environmental cost estimates often rely on simplified assumptions or data from broader administrative scales. These studies fail to fully integrate and spatially represent environmental externalities alongside localized economic costs.^27^^,^^28^ This disconnect makes current assessment frameworks inadequate for identifying optimal treatment pathways specific to each city, limiting their usefulness for formulating context-specific policies, such as those needed for zero-waste cities.

We applied the WEE nexus framework to analyze cost-effective solutions for the MSW treatment transition in 352 Chinese cities, by effectively reconciling the trade-offs between environmental costs and economic performance. Our analysis builds on a bottom-up compilation of historical MSW-related data spanning the past two decades with projections through 2050, covering waste generation, composition, and treatment infrastructure. By integrating facility-level economic data of 1,954 treatment plants with life cycle costing and monetized environmental impacts, we evaluate scenario-specific outcomes from 2020 to 2050. The application of this framework demonstrates that a hybrid pathway integrating incineration with material recycling, in some cases supplemented by bioconversion, is optimal for over 84% of cities, providing a clear, evidence-based rationale for municipal policy transitions.

This study advances the existing literature in three key respects. Methodologically, the WEE framework integrates environmental external costs with detailed economic costs at the urban scale, providing a practical tool for multidimensional trade-off analysis in waste management. Analytically, the high-resolution simulation of 352 cities not only confirms the general effectiveness of hybrid pathways but, more critically, reveals that the optimal configuration of such pathways is highly contingent on local conditions. This highlights the limitation of a one-size-fits-all policy and lays a solid foundation for developing spatially differentiated strategies. In terms of decision-support, the dynamic assessment system constructed in this study translates integrated evaluation results into tailored policy intervention portfolios, such as tiered environmental tax or subsidy schemes, bridging the gap between academic analysis and practical management strategies. Together, these contributions create a scalable, transferable assessment-decision framework. This framework provides a scientific approach to aligning immediate waste management needs with long-term goals of a circular economy and carbon neutrality, applicable in both China and other rapidly urbanizing regions worldwide.

## Materials and methods

### Database construction of China’s MSW treatment

#### MSW treatment plants dataset

We compiled a dataset of 1,954 MSW treatment plants operating across China. Each plant is classified by its primary treatment technology, although individual plants may include multiple facilities or landfill units. Information on plant location, years of operation, treatment capacity, investment, and disposal volumes was systematically collected through extensive online searches, guided by the official registry released by the Ministry of Ecology and Environment of China (https://www.mee.gov.cn/). Key data sources include the Automatic Monitoring Data Public Platform for Household Waste Incineration Power Plants (https://ljgk.envsc.cn/) and the Qichacha (https://www.qcc.com/) enterprise information platform, supplemented by annual local government reports and verified news sources.

#### Historical MSW-related data from 2000 to 2021

Data on MSW generation and treatment for 352 Chinese cities between 2000 and 2021 were primarily obtained from the China Urban-Rural Construction Statistical Yearbook, as detailed in Table S1.^29^^,^^30^ Missing observations were addressed using the data-processing methods described in Ma et al.^31^^,^^32^ This dataset provides a consistent basis for assessing current trends and identifying potential gaps in future MSW treatment capacity at both national and city levels.

#### Projection of MSW generation from 2021 to 2050

MSW generation is closely linked to socio-economic factors, particularly population and per capita gross domestic product. Thus, we applied a multiple linear regression model to project MSW generation from 2022 to 2050 for 292 cities with sufficient historical population and GDP data. For the remaining 60 cities, projections were generated using an autoregressive integrated moving average model. The projection methods refer to Ma et al.^31^^,^^32^ The future MSW generation quantities across cities are presented in Table S6, while detailed forecasts of population, GDP per capita, and MSW generation for 2022–2050 are provided in Text S1.

#### Scenario setting for MSW treatment from 2022 to 2050

Future treatment pathways were defined using a structured, four-step framework to ensure consistency with policy targets, technological trends, and empirical evidence. First, the incineration-focused scenario aligns with China’s 14th Five-Year Plan for Urban Domestic Waste Classification and Treatment Facilities^6^ and references projections from Ma et al.^31^ and Liu et al.,^24^ with baseline incineration rates updated using a consistent methodology. Second, the bioconversion-oriented scenario draws on Wang et al.,^14^ Liu et al.,^33^ and Fang et al.,^34^ with food waste bioconversion rates calibrated to MSW composition and their trajectories toward 2050 reset according to city-specific treatment structures. Third, the recyclable material scenario is primarily based on Fang et al.^34^ and Mu et al.^35^ Finally, a business-as-usual (BAU) scenario and combined strategies such as RR-ID and RR-LBB-ID were developed to examine integrated pathways. Accordingly, we have designed a total of nine distinct MSW treatment scenarios as outlined below. The composition of each scenario is summarized in Table 1. The future MSW disposal structures in various scenarios are presented in Table S7, with the calculation methodology detailed in Figure S1.

• BAU: the future MSW disposal structure remains unchanged from its 2021 configuration throughout the projection period.
• Incineration dominated (ID): the incineration rate continues to increase, reaching near-complete coverage of MSW treatment in China by 2050. This scenario reflects the current national policy orientation that strongly promotes WtE incineration through sustained financial support. Considering the considerable resource and environmental benefits of bioconversion for organic components such as food waste, two additional scenarios emphasizing high-value and refined treatment are proposed:
• Low-rate bioconversion (LB): the bioconversion rates for composting (LBC) and protein recovery (low-rate bioconversion [LBB]) in less-developed and developed cities gradually increase to 30% and 50% by 2050, respectively.
• High-rate bioconversion (HB): the bioconversion rates for composting (HBC) and protein recovery (high-rate bioconversion [HBB]) in less-developed and developed cities rise to 50% and 100% by 2050, respectively.
• In both LB and HB scenarios, organic waste not treated through bioconversion is directed to either incineration or landfilling. Cities are classified as less-developed or developed based on whether their bioconversion rate for organic waste was below or above 1% in 2021.
• Recyclable recovery (RR): recyclable materials, including paper, plastics, glass, textiles, and metals, are recovered rather than incinerated or landfilled, with a recycling rate varying between 0.22 and 0.65.
• Combined scenarios: three integrated scenarios are constructed by combining the above disposal modes: HBB-ID, RR-ID, and RR-HBB-ID. The RR-HBB-ID scenario represents a high-performing resource recovery pathway, in which recyclable materials are recovered for secondary production, food waste undergoes bioconversion for protein recovery, and residual MSW is treated through incineration with energy recovery.

**Table 1:** Overview of MSW treatment scenarios

Scenarios	Scenario narrative	Incineration rate^a^	Bioconversion	Bioconversion disposal rate	Landfill rate	Materials recycling^b^
BAU	ratio of each MSW disposal structure is still same as that in 2021	same as 2021	composting	same as 2021	same as 2021	–
ID	incineration rate gradually increases, and nearly all MSW is combusted by 2050	significantly increasing	composting	significantly decreasing	significantly decreasing	–
LBC	a low proportion of food waste is treated with composting, and the remaining MSW is managed under the BAU scenario	decreasing	composting	low (30% and 50%)	decreasing	–
LBB	a low proportion of food waste is treated using bioconversion technology for protein recovery, and the remaining MSW is managed under the BAU scenario	decreasing	protein recovery	low (30% and 50%)	decreasing	–
HBC	a high proportion of food waste is treated with composting, and the remaining MSW is managed under the BAU scenario	decreasing	composting	high (50% and 100%)	decreasing	–
HBB	a high proportion of food waste is treated using bioconversion technology for protein recovery, and the remaining MSW is disposed according to BAU scenario	decreasing	protein recovery	high (50% and 100%)	decreasing	–
HBB-ID	a high proportion of food waste is treated for protein recovery, and the remaining MSW is managed under the ID scenario	–	protein recovery	high (50% and 100%)	significantly decreasing	–
RR-ID	recyclable materials undergo resource recovery, while the remaining MSW is managed under the ID scenario	significantly increasing	composting	gradually decreasing	significantly decreasing	✓
RR-HBB-ID	recyclable materials are recycled, high proportions of food waste are treated for protein recovery, and the remaining waste is managed under the ID scenario	–	protein recovery	high (50% and 100%)	significantly decreasing	✓

The difference among scenarios is based on the combination of the three modes (ID, LB, and HB) and the strategy of recyclable materials recycling, as well as the employment of biochemical disposal method, leading to distinct MSW treatment structures in each city from 2022 to 2050.

a In scenarios HBB-ID and RR-HBB-ID, the rate of incineration depends on the specific situation of each city considering the increase of bioconversion for food waste.

b Recyclable materials in MSW, including paper, plastics, glass, textiles, and metals, will be recycled based on a fixed collection rate before incineration and landfill. Their recycling rates are 22% for textiles, 44% for glass, 51% for paper, 31% for plastics, and 65% for metals. The classification for cities as less-developed or developed is based on whether the rate of biochemical disposal for organic components was lower or higher than 1% in 2021.

The projected physical composition of MSW was estimated following the methodology established by Ma et al.^32^ This approach employs a data-driven model that integrates field investigation data with key socio-economic factors. A back-propagation neural network was used to capture the relationship between waste composition and its underlying drivers, with data preprocessing applied to ensure consistency with compositional data properties. After validation, the model was used to reconstruct historical trends and estimate waste composition patterns for the period 2000–2021. Because future waste composition is influenced by complex and uncertain factors such as economic development, changing lifestyles, and climate impacts, this study assumes that MSW composition remains fixed at its 2021 level, as detailed in Table S3.

### The revenue from secondary products and the cost of MSW treatment

The economic performance for MSW disposal strategies ($C_{\text{MSW},t}^{k}$) in this study include treatment costs and the revenues from products sale ($R_{\text{recycle},t}^{k}$). The costs for MSW treatment in terms of incineration, landfill, and biochemical disposal ($C_{disposal,t}^{k}$) involve the fixed investment cost ($C_{\text{inv},t}^{k}$) and operational costs ($C_{\text{ope},t}^{k}$).

(1) $$C_{\text{MSW},t}^{k}=R_{\text{recycle},t}^{k}+\sum_{disposal}C_{disposal,t}^{k}$$

(2) $$C_{disposal,t}^{k}=C_{\text{inv},t}^{k}+C_{\text{ope},t}^{k}$$

where *disposal* represents various methods of MSW disposal, including incineration, landfill, and bioconversion. The investment amount for MSW treatment plants is converted into investment cost for processing 1 ton of MSW ($c_{\text{inv},t}^{k}$), which is then multiplied by the quantity of MSW disposed ($Q_{disposal,t}^{k}$) for the calculation of fixed investment, to reflect the differences in waste disposal intensity among cities. For the calculation of $Q_{disposal,t}^{k}$ refer to Text S2.

(3) $$C_{\text{inv},t}^{k}=c_{\text{inv}}^{k}\cdot Q_{disposal,t}^{k}$$

(4) $$c_{\text{inv}}^{k}=\frac{\sum_{i}I_{i}^{k}}{N_{disposal}\cdot \sum_{i}Q_{i}^{k}}$$

where $I_{i}^{k}$ and $Q_{i}^{k}$ are the total investment amount and annual MSW disposal quantity, respectively. N_*disposal*_ is the operational lifespan, which is 20 years for incineration plants, 50 years for landfill sites, and 15 years for biochemical disposal plants, respectively.^18^^,^^36^ *i* represents different MSW treatment plants. In Equation 4, only MSW treatment plants in the same city are taken into consideration.

The economic costs of MSW plant investments were derived from our proprietary dataset, and the average investment cost per unit of MSW treatment was subsequently calculated for each city. Notably, there remain 279 cities in China lacking data on bioconversion plants, while 106 cities lack data on incineration plants, and 215 cities lack data on landfill sites. It is assumed that the investment costs for processing 1 ton of MSW in these cities are at the average level of the provinces in which they are located. Moreover, if a province lacks data on MSW treatment plants, the investment costs in the cities of that province are assumed to be at the average level in China.

The operational cost for MSW disposal is calculated by multiplying the quantity of MSW and operational cost for processing 1 ton of MSW including product revenue (*r*_product_), subsidies (f_subsides_), operation and management cost (c_manage_), energy cost ($c_{\text{energy}}^{k}$), and other costs (c_other_). The energy cost for processing 1 ton of MSW differs among cities due to the different prices of electricity and diesel, while others remain fixed. Operational revenues are mainly from secondary products sale for material recycling or produced products for landfill, incineration, and bioconversion. Operational cost and revenues for MSW management were compiled from multiple sources. Data for incineration were obtained from Zhou and Zhang^21^ and Zhang et al.,^37^ while data for bioconversion were sourced from Liu et al.,^33^ Bohm et al.,^38^ and Xue et al.^39^ Additional parameters for landfill were referenced from official documents, including CNDRC^40^ and CMEE.^41^ Text S3 explains the specific information regarding operational benefits for MSW incineration, landfill, and biochemical disposal.

(5) $$C_{\text{ope},t}^{k}=c_{\text{ope},t}^{k}\times Q_{disposal,t}^{k}$$

(6) $$c_{\text{ope}}^{k}=r_{\text{product}}+f_{\text{subsides}}-c_{\text{manage}}-c_{\text{energy}}^{k}-c_{\text{other}}$$

The technologies for MSW incineration and bioconversion are not yet matured, indicating that they will experience a reduction in capital expenditure, specifically investment cost, due to the effects of accumulated experience and learning.^41^ The decreasing ratios of investment costs for these technologies are shown in Figure S2. This rate is also applied to the operation and management cost, given its dependence on investment cost.

Recycling benefits include sorting and transportation cost ($C_{\text{ST},t}^{k}$) as well as the revenues for secondary products sale ($R_{\text{recycle},j,t}^{k}$), both of which depend on the quantity of collected recyclable materials ($Q_{\text{recycle},j,t}^{k}$). The recyclable materials involved in this study include metals, glass, plastics, paper, and textiles. The calculation of $Q_{\text{recycle},j,t}^{k}$ refers to Text S2.

(7) $$R_{\text{recycle},t}^{k}=\sum_{j}R_{\text{recycle},j,t}^{k}-C_{\text{ST},t}^{k}$$

(8) $$R_{\text{recycle},j,t}^{k}=\left(\varphi_{j}p_{\text{recycle},j}-c_{\text{recycle},j}\right)\times Q_{\text{recycle},j,t}^{k}$$

(9) $$C_{\text{ST},t}^{k}=\left(c_{\text{sort}}+c_{\text{transport}}\right)\times \sum_{j}Q_{\text{recycle},j,t}^{k}$$

where φ_*j*_ is the substitution ratio of recycled materials. p_recycle,*j*_ is the price of recycled materials. c_recycle,*j*_ is the cost for processing 1 ton of recyclable materials. c_sort_ and c_transport_ are the sorting cost and transportation cost for collecting 1 ton of recyclable materials, respectively. *j* represents different recyclable materials. Moreover, it should be noted that the recycling benefits vary due to the differences in MSW composition across different cities.

### Calculation of environmental costs for MSW disposal

To evaluate the environmental impacts of different MSW disposal strategies, we utilized ReCiPe 2016 LCA indicators, which consist of 18 indicators (global warming potential, ozone depletion potential, particulate matter formation potential, photochemical and human oxidant formation potential for ecosystems, ionizing radiation potential, freshwater eutrophication potential, marine eutrophication potential, freshwater ecotoxicity potential, marine ecotoxicity potential, water consumption potential, terrestrial acidification potential, terrestrial ecotoxicity potential, land use, human toxicity potential for cancer, and non-cancer effects, fossil resource scarcity potential, and mineral resource scarcity potential, covering five aspects: air, water, soil, human, and resource depletion, as detailed in Table 2. Chinatax^RCP^, a regionalized monetization model applicable to ReCiPe 2016 LCA indicators, was employed to calculate these 18 indicators across cities in China based on the current national environmental tax framework and regional carbon abatement costs, which represent the most policy-relevant valuation benchmark at the time of analysis. Selection of environmental indicators and their corresponding monetization factors refer to Liu et al., as detailed in Table S11.^33^ Then, the externality costs for MSW disposal ($E_{\text{MSW},t}^{k}$) could be calculated based on these 18 indicators and corresponding monetization factors. It is important to note that, while this approach grounds the cost estimates in actual regulatory mechanisms, the potential future evolution of environmental taxation policies is not modeled, representing a defined boundary condition for this long-term assessment. And the externality costs will vary with MSW disposal structures by 2050 due to differences in the emissions of pollutants from various MSW disposal methods.

(10) $$E_{\text{MSW},t}^{k}=\sum_{l=1}^{18}\left(\omega_{l}^{k}\times M_{l,t}^{k}\right)$$

(11) $$M_{l,t}^{k}=\sum_{disposal}\left(e_{\text{disposal},t}^{l}\times Q_{\text{disposal},t}^{k}\right)$$

where $\omega_{l}^{k}$ is the monetization factor. $M_{l,t}^{k}$ are the equivalent emissions of pollutants from MSW disposal or recycling. $e_{\text{disposal},t}^{l}$ is the emission intensity of MSW disposal (incineration, landfill, and biochemical disposal), respectively. *l* represents different LCA indicators. Moreover, it is noted that the positive impacts of technological improvements on long-term emissions from MSW incineration are considered. Specifically, emissions from MSW incineration are expected to decrease to 90%, 80%, and 70% of that in 2020 by 2030, 2040, and 2050, respectively. The specific calculation of various monetization factors is detailed in Text S4.

**Table 2:** LCA indicators in the ReCiPe model

Type	Name	Unit	Equation
Air related	global warming potential (GWP)	kg CO_2_ eq.	S29
	ozone depletion potential (ODP)	kg CFC-11 eq.	S30-S33
	particulate matter formation (PMFP)	kg PM2.5 eq.	S13-S15
	photochemical oxidant formation potential: ecosystems (EOFP)	kg NOx eq.	
	photochemical oxidant formation potential: humans (HOFP)	kg NOx eq.	
	ionizing radiation potential (IRP)	kBq Co-60 eq.	S40-S41
Water related	freshwater eutrophication (FEP)	kg P eq.	S13-S15
	marine eutrophication (MEP)	kg P eq.	
	freshwater ecotoxicity (FETP)	kg 1,4-DCB eq.	S16
	marine ecotoxicity (METP)	kg 1,4-DCB eq.	S17
	water consumption (WCP)	m^3^ water eq.	–a
Soil related	terrestrial acidification (TAP)	kg SO_2_ eq.	S13-S15
	terrestrial ecotoxicity (TETP)	kg 1,4-DCB eq.	S36-S39
	land use (LU)	m^2^ × year annual cropland eq.	S20-S28
Human related	human toxicity potential, cancer (HT_c_)	kg 1,4-DCB eq.	S34-S45
	human toxicity potential, non-cancer (HT_nc_)	kg 1,4-DCB eq.	
Resource depletion related	fossil resource scarcity (FFP)	kg oil eq.	S19
	mineral resource scarcity (SOP)	kg Cu eq.	S18

The specific explanation on the LCA indicators in the ReCiPe model refers to the work of Liu et al.^33^

a The average resource taxes (or fees) on surface water in each province were used as the monetization factors of the indicator WCP in 31 provinces.

### Approach to identifying the optimal MSW disposal strategy for each city

To comprehensively evaluate the economic performance and environmental performance of different MSW disposal modes and achieve a city-specific trade-off between these two dimensions, a composite benefit index for various MSW disposal modes is constructed. First, the absolute maximum economic (or environmental) cost value among all scenarios is selected as the benchmark for each city. The economic (and environmental) cost values of each scenario are then normalized relative to this maximum value to derive an economic (and environmental) benefit index. This normalization process aims to eliminate the effects of dimensional differences and measure the relative optimization level of economic (or environmental) performance within the range of all considered scenarios. Subsequently, considering that the environmental performance and economic performance are equally important, the composite benefit index ($SCORE_{\text{MSW},t}^{k,s}$) is defined as the sum of the economic benefit index and the environmental benefit index. Based on this framework, by comparing the composite benefit indices across different scenarios, the optimal MSW disposal strategy that balances both economic and environmental performance can be identified for each city.

(12) $$SCORE_{\text{MSW},t}^{k,s}=\frac{-C_{\text{MSW},t}^{k,s}}{\max_{s}\left(\left|C_{\text{MSW},t}^{k,s}\right|\right)}+\frac{-E_{\text{MSW},t}^{k,s}}{\max_{s}\left(\left|E_{\text{MSW},t}^{k,s}\right|\right)}$$

where *s* represents different scenarios.

### Validation and sensitivity analysis

To validate the macro-level accuracy of our city-aggregated bottom-up framework and assess potential deviations, we conducted a two-step verification. First, we compared our aggregated national-level results (e.g., total treatment costs, GHG emissions) for key baseline years with those reported in peer-reviewed national-scale studies (e.g., Liu et al.^33^ and Ma et al.^31^). The differences were found to be within a relatively narrow and acceptable range for macro-level scenario analyses. Second, we performed a sensitivity analysis by perturbing key facility-level parameters, such as introducing plausible variation in operational costs and environmental impact intensities within their documented ranges, and re-aggregating to the city and national levels. The resulting variation in total national environmental and economic costs remained within a bounded and limited margin. These analyses confirm that, while micro-heterogeneity among individual plants exists, our aggregation approach does not introduce significant systematic bias at the macro-scale, and the estimated deviations are not substantial enough to alter the comparative ranking or primary conclusions of our scenario analysis.

## Results

### Trajectories of city-level MSW generation and treatment in China from 2000 to 2050

China’s total MSW generation increased from 113 million tons (Mt) in 2000 to 256 Mt in 2021, with significant variation across cities. Wealthier and more populous areas tend to generate more waste, leading to concentration in eastern coastal regions and provincial capitals. In 2022, the top 5 MSW producers were Shanghai, Beijing, Shenzhen, Chongqing, and Chengdu. Of these, Shenzhen experienced the greatest rise in MSW generation from 2000 to 2021, a trend driven by income and population growth, followed by Chongqing and Chengdu.

**Figure 1:**
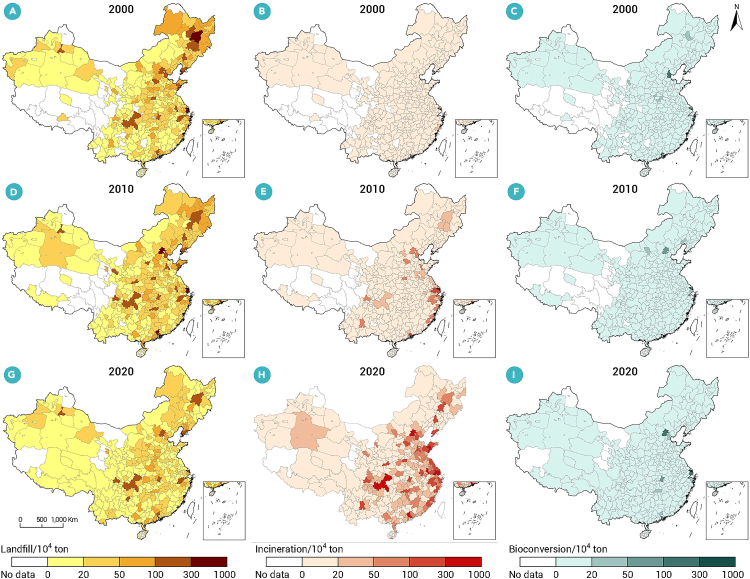
Evolution of MSW treatment in China (A–I) MSW amounts treated by landfill, incineration, and bioconversion for 352 cities in 2000, 2010, and 2020.

The historical evolution of MSW treatment structures across 352 cities is detailed in Figure 1. MSW treatment strategies vary across Chinese cities. While most have significantly adopted incineration, others (e.g., Danzhou, Karamay, Panzhihua, and Yuxi) have followed different paths. The shift from landfilling to incineration with energy recovery is driven by land scarcity, stricter environmental regulations, and the advantages of incineration in reducing waste volume, recovering heat, and generating power.^12^^,^^42^^,^^43^ By 2021, China’s harmless treatment rate for MSW had reached 99.9%, with incineration, landfilling, and bioconversion accounting for 72.5%, 21.1%, and 6.5%, respectively. Notably, 33.8% of cities relied on incineration to treat more than 90% of their MSW, and 46.0% of cities used it for over 80%. High shares of bioconversion were observed in both less-developed cities (e.g., Leshan and Yingkou) and more developed ones (e.g., Beijing and Wuhan).

A panel data regression model, based on projected population and GDP per capita, estimates that MSW generation across the 352 cities will reach 358.6 Mt by 2050, a 52.5% increase from 2020, aligning with earlier projections by Wei et al.^44^ The projected annual growth rate of 1.4% from 2022 to 2050 is similar to the World Bank’s estimate of 1.7% for the East Asia-Pacific region, but lower than the historical rate of 1.9% from 2000 to 2021. By 2050, Shenzhen, Wuhan, and Shanghai are each projected to generate over 10 Mt of MSW annually, followed by Chengdu at 9.6 Mt and Beijing at 8.6 Mt. Nevertheless, waste growth in these major cities is expected to slow after 2021. In contrast, several cities in central and western China, such as Dazhou, Zhongwei, Haidong, Xuchang, and Chuzhou, are projected to experience rapid growth, with MSW generation increasing more than 3-fold. This pattern reflects the general association between city size and waste generation, although this link is expected to weaken as urbanization slows.^45^

To systematically identify feasible MSW treatment strategies for cities across China, we designed and developed nine scenarios based on combinations of four principal disposal methods: landfilling, incineration, bioconversion (for composting and protein recovery), and recyclable materials recovery. For instance, in the ID scenario, incineration increases to 95.8% by 2030 and reaches 99.3% by 2050. Meanwhile, the HBB and LBB scenarios assume that 81.0% and 52.2% of organic waste, respectively, will go to protein recovery via bioconversion by 2050. By 2050, developed cities (including direct-administered municipalities, provincial capitals, and pilots such as Taizhou and Deyang) are expected to treat over 50% of MSW through bioconversion. In contrast, many less-developed cities and other pilots (e.g., Guangyuan and Yichun) will likely still rely mainly on incineration. The RR-HBB-ID scenario offers an ideal integrated framework. It maximizes resource use by combining three streams: recycling materials (e.g., paper, plastics, textiles, metals, and glass), converting food waste at high rates to recover protein, and incinerating residual waste for energy. This approach is based on current city practices and each city’s level of bioconversion technology.

### Environmental costs in future MSW treatment transitions

Physical indicators provide key insights into the environmental burdens and trade-offs of each waste treatment scenario. Under the BAU scenario, GHG emissions from MSW treatment are expected to decrease by about 7.0% from 2020 to 2050, mainly due to technological advancements. The ID strategy achieves a more pronounced reduction, lowering GHG emissions by 19.2% compared with BAU by 2050, which is largely attributable to the mitigation of landfill methane. The most significant emission cuts are associated with WtM strategies. Notably, the RR-HBB-ID scenario attains up to 85.5% GHG reduction through improved resource recovery and the avoidance of emissions from conventional disposal pathways. Advanced incineration with energy recovery offers a relative advantage in fossil resource conservation, with the potential to save approximately 2,700 tons of oil equivalent by substituting primary fuel extraction. Spatial analysis highlights a critical trade-off: although incineration reduces carbon footprints, it may intensify regional air pollution and aquatic toxicity in regions with insufficient emission controls. In contrast, large-scale composting and bioconversion enhance carbon sequestration and soil health but necessitate diligent management to avert nutrient runoff and freshwater eutrophication. These results confirm that optimizing the environmental performance of waste management requires alignment with regional priorities, establishing a foundation for integrated physical and economic assessments. National and city-specific environmental impacts are detailed in Figure S3 and the supplemental information (extended data), respectively.

**Figure 2:**
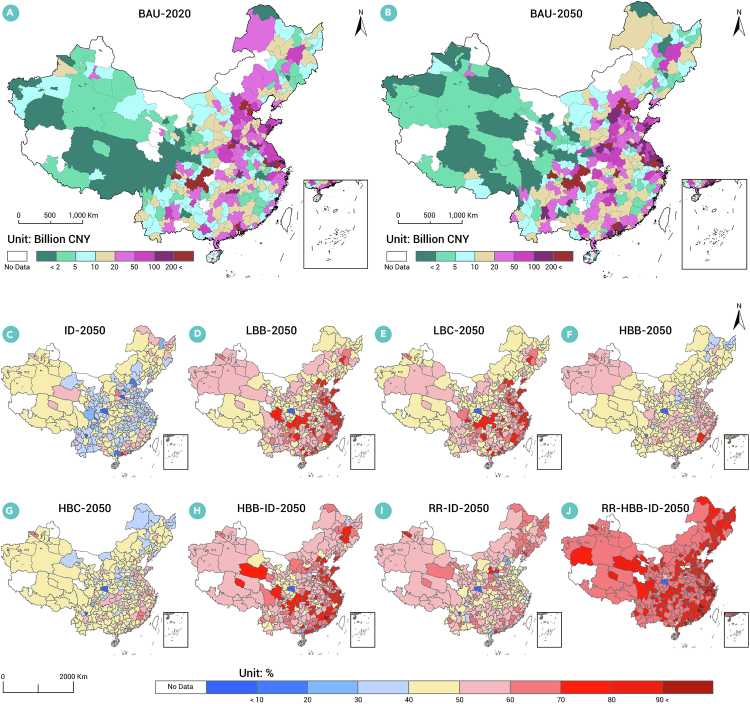
Comparison of environmental costs at city-level across different scenarios (A and B) The monetized environmental costs in 2020 and 2050 under the business-as-usual (BAU) scenario, respectively, based on the assessment of 18 environmental impact categories associated with MSW treatment. (C–J) The differences in environmental costs between the BAU scenario and eight alternative waste-to-energy (WtE) and waste-to-material (WtM) scenarios in 2050. The red color highlights significant reductions in environmental costs when transitioning from the BAU scenario to WtE or WtM strategies.

To quantify environmental externalities in the MSW management system, we convert 18 environmental impact indicators into monetary values using region-specific monetization factors. The significantly higher environmental costs of incineration and landfilling make a strong case for shifting to WtM approaches. Specifically, landfilling incurs environmental costs approximately 1.8 times those of incineration and 228 times higher than traditional composting. In contrast, bioconversion for protein recovery can generate net environmental benefits. Our scenario evaluations, based on full life cycle climate and environmental impacts, provide a standardized framework for designing policy instruments, such as environmental taxes and compensation mechanisms. Comparative environmental costs at the city level are shown in Figure 2.

Under the BAU scenario, total environmental costs increase by 23.0% from 2020 to 2050, primarily driven by growing MSW treatment volumes (Figure 1). However, by 2030, costs stabilize near 2020 levels as reductions in landfill impacts offset higher incineration costs. Shifts to WtE or WtM strategies achieve significant reductions in environmental costs by 2050, with declines ranging from 36.9% for ID scenario to 78.2% for the RR-HBB-ID scenario. However, bioconversion for composting and protein recovery shows minimal cost divergence (Δ ≈ 2%), reflecting reduced reliance on incineration, which lowers its external costs.

There is significant variability in environmental costs across cities, with the greatest burdens concentrated in economically developed and densely populated urban areas. In 2020, Beijing, Tianjin, Suzhou, Chongqing, and Nanjing recorded the highest environmental costs. By 2050, under the BAU scenario, the ranking is expected to change, with Beijing, Tianjin, Suzhou, Wuhan, and Chengdu showing the highest impacts. These megacities have high MSW treatment demands, mainly met through incineration and landfilling, which significantly increase their environmental footprints. While Suzhou and Tianjin generate less MSW than Chongqing, their higher GDP per capita results in greater environmental costs per unit of waste. Rising costs in Wuhan and Chengdu are linked to demographic and economic factors, amplified by national development initiatives such as the Western Development Plan and the Belt and Road infrastructure project. Cities in central and eastern China see the greatest gains from bioconversion adoption, especially in protein recovery compared with composting. However, cities in the western regions may face rising environmental costs due to their continued dependence on incineration for waste disposal.

**Figure 3:**
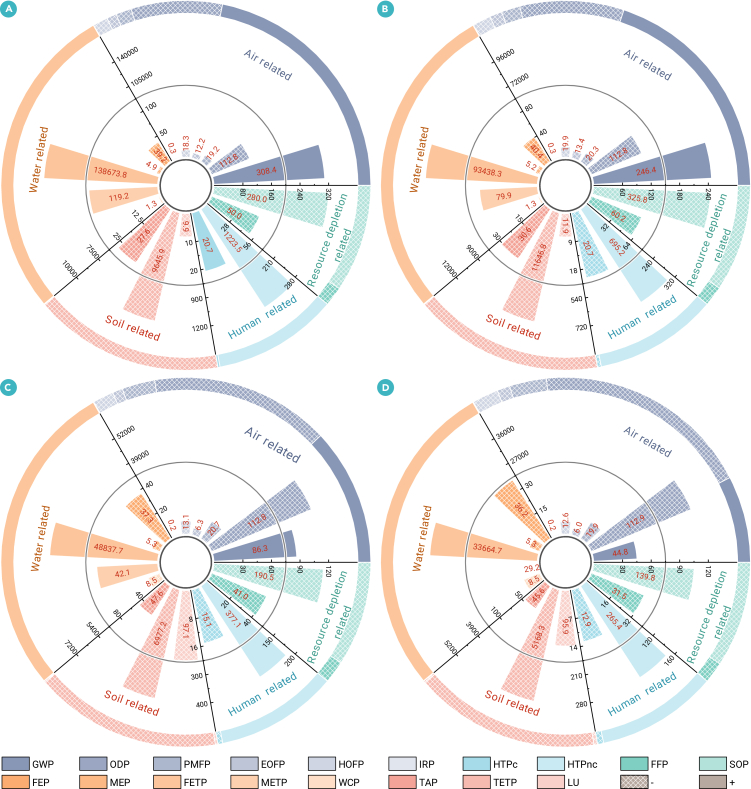
Monetization results of environmental costs in different scenarios (A–D) The monetization results of various environmental cost categories in 2050 under the BAU, ID, HBB-ID, and RR-HBB-ID scenarios. The color-coded bars and rings represent different environmental cost indicators, with white grids indicating environmental benefits and solid colors representing environmental costs. The height of each bar corresponds to the specific monetized value of the respective indicator, while the length of each ring reflects the relative contribution of the indicator within its category. The environmental indicators evaluated in this study include: global warming potential (GWP), ozone depletion potential (ODP), particulate matter formation potential (PMFP), photochemical oxidant formation potential for ecosystems (EOFP) and humans (HOFP), ionizing radiation potential (IRP), freshwater eutrophication potential (FEP), marine eutrophication potential (MEP), freshwater ecotoxicity potential (FETP), marine ecotoxicity potential (METP), water consumption potential (WCP), terrestrial acidification potential (TAP), terrestrial ecotoxicity potential (TETP), land use (LU), human toxicity potential for cancer (HTc) and non-cancer effects (HTnc), fossil resource scarcity potential (FFP), and mineral resource scarcity potential (SOP).

Environmental impact trade-offs are critically shaped by indicator variability (Figure 3). Freshwater ecotoxicity contributes over 98.8% to total environmental costs, while soil-related impacts (particularly terrestrial ecotoxicity) exert net benefits offsetting 4.6%–15.2% of the costs. Bioconversion methods, including composting and protein recovery, significantly reduce freshwater ecotoxicity and enhance resource recovery versus BAU/ID scenarios. However, hybrid WtM-WtE scenarios (HBB-ID and RR-HBB-ID) perform worse than standalone WtE in terms of mineral conservation and human health protection. This limitation arises from the limited capacity of biochemical products (compost and protein substitutes) to fully replace traditional products such as fertilizers and animal-derived proteins. Our findings highlight that substantial life cycle environmental cost reductions require prioritizing biochemical treatment and overcoming substitution barriers.

### Economic-environmental trade-offs in MSW treatment transition

**Figure 4:**
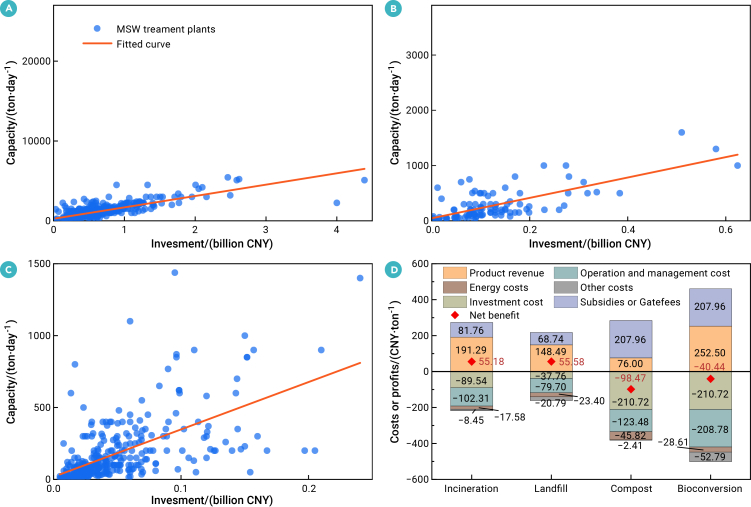
Investment for MSW plants and economic costs (or benefits) of different MSW disposal methods (A–C) Investment amounts and disposal capacities for incineration plants, biochemical disposal plants, and landfill sites in this study. The orange lines represent the fitting curves between investment and capacity for the three kinds of MSW disposal facilities. Additionally, the three panels share identical *x* and *y* axis scaling. (D) Benefits and costs of incineration, landfill, traditional compost and bioconversion for 1 ton of MSW. The colors of the bars denote various revenue and cost items, while the red five-pointed stars represent the net benefit.

**Figure 5:**
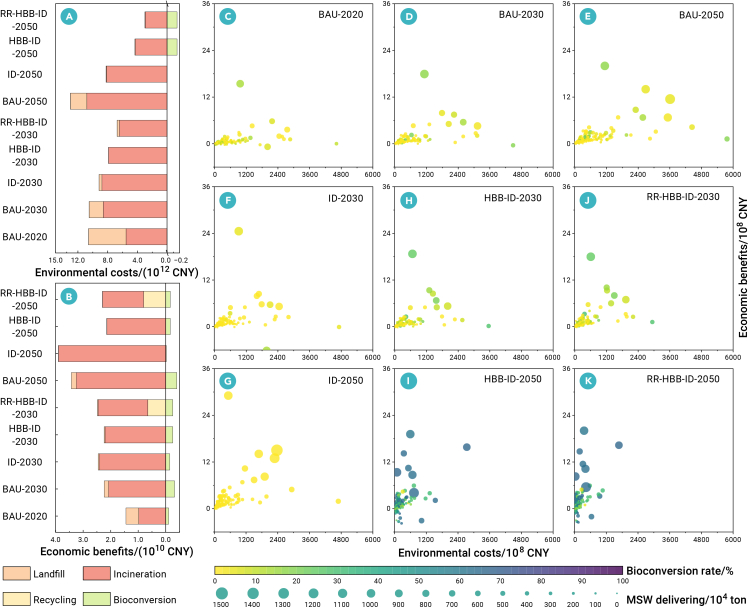
Economic benefits and environmental costs at city and national levels across scenarios (A and B) National-level environmental costs and economic benefits, respectively, across scenarios. The colors of the bars denote various MSW disposal methods, including incineration, landfill, biochemical disposal, and recyclable materials recycling. (C–K) City-level economic benefits and environmental costs under different scenarios. The color of each dot represents the biochemical disposal rate as a percentage of total MSW disposal, while the dot size corresponds to the quantity of MSW generated. It is important to note that this study does not account for the environmental costs associated with the recycling process or the environmental benefits derived from the use of recycled materials, which may result in slightly higher environmental costs in scenarios RR-HBB-ID and RR-ID. Additionally, a few cities, such as Beijing and Shenzhen, are excluded from the graphs due to their significantly higher economic benefits or environmental costs compared with other cities, which would otherwise distort the visualization.

The net economic benefit of each MSW treatment scenario was assessed as the difference between total revenues (from energy/products and government subsidies) and total costs (including investment, operation, management, and other expenses). The formula is: net economic benefit = (product income + subsidies) – (investment + O&M costs + other costs). Notably, a negative economic benefit represents a net cost of MSW treatment. First, investment data for 1,954 MSW treatment plants across 352 cities in China were collected (Figure 4). With MSW disposal fees ranging from CNY 50–200 per ton, significantly lower than environmental costs, targeted environmental taxes or subsidies could incentivize the adoption of WtM strategies. The increasing adoption of waste incineration and bioconversion has notably reduced environmental burdens. While most cities see modest economic gains in all scenarios, disparities between profitable and unprofitable cities widen in WtE and WtM scenarios. As shown in Figure 5, cities operating at a deficit face increased financial pressure, while profitable cities see greater benefits, especially under the HBB-ID and RR-HBB-ID scenarios. These differences are mainly due to variations in facility utilization rates, which affect unit investment costs for biochemical disposal and influence the economic viability of WtE and WtM strategies. For example, cities such as Chongqing, Kunming, Guangzhou, and Dongguan achieve substantial economic benefits under the HBB-ID scenario, whereas Dalian, Harbin, Jinan, Shanghai, Shenzhen, and Suzhou incur higher economic costs. The BAU and ID scenarios impose relatively smaller economic burdens. Some cities, including Shanghai, Suzhou, and Dalian, even achieve modest gains under these scenarios, largely due to lower investment costs associated with incineration.

As shown in Figures 5A and 5B, MSW treatment pathways in China vary significantly across scenarios. From 2020 to 2050, environmental costs rise under the BAU scenario but decrease substantially when shifting to WtE and WtM scenarios, with WtM proving particularly cost-effective. While economic costs under the BAU scenario increase by 125.6% from 2020 to 2050, the implementation of WtM strategies could lead to a 189% reduction compared with 2020. At the national level, the RR-HBB-ID scenario is identified as the most effective for reducing environmental costs, while the RR-ID scenario performs best in improving economic benefits. The combined application of these strategies could lead to a 78.3% reduction in environmental costs and a 33.9% increase in economic benefits by 2050. Consequently, these findings provide strong support for pivoting from landfill-dependent systems toward integrated WtE and WtM approaches.

In general, Chinese cities exhibit four distinct cost profiles shaped by differentials in waste volume, composition, and local infrastructure. Megacities such as Beijing and Shanghai focus on waste reduction, even though they face higher operational costs. Coastal cities with strong regulatory frameworks, such as Xiamen and Quanzhou, maintain low environmental costs through well-managed waste systems. Inland industrial cities such as Zhengzhou have higher environmental costs, mainly due to their continued dependence on landfilling. Cities such as Xi’an and Hanzhong benefit from advantageous waste composition, characterized by lower organic content and elevated shares of paper and wood, which collectively mitigate contamination risks and reduce treatment expenses. This marked regional variation underscores the need for city-specific waste management strategies. Megacities are best served by integrated or rapid-reduction treatment solutions, while smaller cities can improve outcomes by aligning treatment technologies with local waste characteristics and modernizing infrastructure.

### City-specific solutions in WEE nexus development

**Figure 6:**
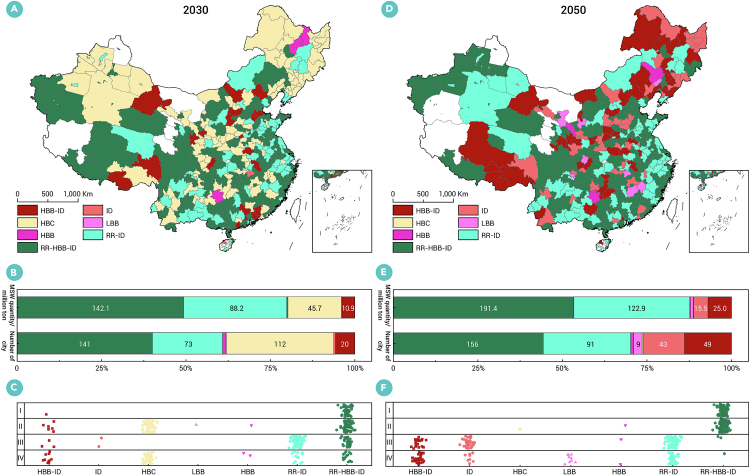
Cost-effective MSW treatment solutions across China’s cities (A and D) City-level optimal MSW treatment strategies balancing economic and environmental performance in 2030 and 2050, respectively. (B and E) The national penetration rates of various MSW treatment strategies, respectively, in 2030 and 2050, categorized by both the number of cities and MSW treatment quantity. The red numbers represent the number of cities and MSW treatment quantity of the other strategies. (C and F) The relationship between the comprehensive optimal MSW treatment strategy and both environmentally optimal and economically optimal strategies across cities. Ⅰ shows that the environmentally optimal strategy and the economically optimal strategy are the same. Ⅱ and Ⅲ show that the comprehensive optimal strategy is consistent with the environmentally or economically optimal strategy, respectively. Ⅳ shows that the comprehensive optimal strategy is neither an environmentally nor an economically optimal strategy, which means a compromise strategy. The colors of above panels represent different MSW treatment strategies.

Our city-level analysis reveals that synergistic waste treatment pathways, particularly the RR-HBB-ID model, strike the optimal balance between economic and environmental costs and are poised to become the dominant future strategy. As shown in Figure 6, macro-scale projections further indicate that, by 2030, 141 and 73 cities are projected to adopt the RR-HBB-ID and RR-ID models, respectively. By 2050, the number of cities implementing the RR-HBB-ID model is expected to reach 156, with an additional 91 and 49 cities adopting the RR-ID and HBB-ID models, while only 43 cities are anticipated to retain the singular ID mode. This indicates that integrated MSW treatment strategies, including RR-HBB-ID, RR-ID, and HBB-ID, are the most optimal and cost-effective solutions for over 84% of the cities studied. This trend underscores a fundamental shift from traditional, discrete treatment modalities toward integrated pathways that combine energy recovery with material recycling.

However, by 2050, most cities will face a trade-off between economic and environmental performance, requiring local policymakers to choose between the economically focused RR-ID strategy and the environmentally focused RR-HBB-ID strategy. For instance, cities with greater economic sensitivity, such as Jilin and Jinan, are better suited for the RR-ID strategy, while those focusing on environmental goals, such as Xiamen and Weihai, will benefit more from the RR-HBB-ID strategy. This difference is mainly due to significant variations in the economic costs of applying bioconversion technologies across cities. Notably, for cities such as Beijing, Chongqing, and Chengdu, the RR-HBB-ID model offers an optimal solution, balancing both economic and environmental benefits.

Overall, our analysis points to a nationwide transition, shifting from heavy reliance on WtE conversion to an integrated resource recovery system that develops both WtE and WtM pathways together. This synergistic approach delivers substantial environmental benefits at a manageable economic cost, proving more cost-effective and sustainable than energy-focused pathways alone. This transition will accelerate GHG mitigation and represent a significant step forward for urban waste management in China, moving closer to circular economy goals.

## Discussion

MSW management is closely tied to key global challenges such as climate change, resource scarcity, and public health. As the world’s largest producer of MSW, China has focused on waste incineration, especially in high-density cities such as Shanghai, Shenzhen, and Guangzhou. This focus is driven by incineration’s efficiency in reducing waste volume, generating reliable energy, and addressing waste accumulation quickly. In contrast, bioconversion technologies promote sustainability through resource recycling, GHG reduction, and value-added products, but are limited by high operational costs, slow processing speeds, and scalability challenges. This technological divide reflects the tension between urgent disposal needs and long-term environmental goals.

China has made significant progress in addressing its historical “waste siege,” shifting the core issue from inadequate treatment capacity to structural imbalances and the need for improved resource recovery. By 2024, China’s harmless MSW treatment capacity reached 1.16 million tons per day, well above the annual waste volume of 262 million tons, leaving incineration plants operating at about 60% capacity.^46^^,^^47^ Following the Implementation Plan for the Domestic Waste Sorting System in 2017, MSW classification has been widely implemented, targeting full urban coverage by 2025,^48^ while the 14th Five-Year Plan mandates a recycling rate of no less than 35%,^49^ marking a policy evolution from end-pipe reduction toward integrated source reduction and whole-process resource utilization. However, downstream recycling, refined management, and high-value conversion remain underdeveloped due to profit-driven enterprise behavior, resulting in slow policy implementation and unresolved environmental externalities. This highlights the need for innovative approaches that account for environmental costs and promote sustainable MSW transitions.

Notably, over 84% of Chinese cities achieve optimal cost-effectiveness under the RR-HBB-ID, RR-ID, and HBB-ID scenarios, suggesting that hybrid systems are the most practical solution for most cities. Furthermore, the unexpected net benefits from soil-related impacts show that WtM strategies provide ecosystem services beyond waste management, such as soil restoration and improved agricultural sustainability, aligning waste management with broader goals such as land degradation neutrality and climate adaptation. This study confirms that the RR-HBB-ID approach minimizes both economic and external costs, while achieving the most significant reductions in key environmental impacts, such as GHG emissions and aquatic toxicity.

However, effective implementation requires city-specific solutions. In northern cities with harsh climates and slow waste classification progress, such as Harbin and Datong, regulated incineration remains viable. Developed northern cities, such as Beijing, Jinan, and Tianjin, should adopt hybrid systems combining advanced biological treatment with limited incineration. The Yangtze River Delta should focus on insect-based bioconversion and anaerobic digestion, supported by environmental taxes before 2030. Less-developed southern cities with high populations may implement composting systems with environmental taxes after 2030. Smaller cities need customized solutions that fit their local infrastructure and resource limitations.

Our analysis highlights the importance of technological complementarity in a synergistic hybrid system, rather than advocating for a complete replacement of incineration with biotreatment in megacities. Incineration is crucial for base-load treatment, stabilizing waste streams, and mitigating risks, while WtM bioprocessing focuses on source-separated organics to improve resource recovery and reduce environmental impacts.

We further propose differentiated transition pathways tailored to city types such as megacities, high-density coastal cities, and smaller inland cities, considering factors such as land availability, waste composition, and regional socio-economic conditions. The shift toward integrated WtE and WtM systems will be gradual, relying on the development of sorting systems, technological advances, cost reductions, and supportive policies. This section provides a decision-support framework for optimizing long-term waste management strategies within the limits of treatment capacity, rather than promoting a one-size-fits-all solution.

Notably, emerging bioconversion technologies advance circular economy objectives by producing alternative protein feeds and reducing reliance on conventional agricultural systems, while also underscoring the need to transition from traditional thermal treatment methods. Composting requires low initial investment but is highly dependent on the marketability and distribution efficiency of the compost. In contrast, anaerobic digestion, on the other hand, requires significant capital investment, with profitability tied to long-term energy revenues from biogas use. Protein recovery such as black soldier fly systems employs a diversified income model that combines waste treatment fees with the sale of valorized products, notably insect protein and organic fertilizers, capitalizing on the substantial commercial potential of insect-based commodities.

Metropolitan areas face dual challenges of limited land and public opposition, leading to the adoption of integrated waste treatment parks or small, decentralized facilities within urban infrastructure, such as underground systems or co-located transfer stations, to fit high-density areas. While urban kitchen waste streams provide a consistent feedstock, operational flexibility remains essential to manage input fluctuations.

Consequently, future MSW strategies will favor context-specific integration of complementary processes, such as combining black soldier fly bioconversion with anaerobic digestion or co-composting, to maximize waste reduction, resource recovery, and energy generation, supporting zero-waste cities and circular economy goals. However, constraints such as high operational costs, intensive preprocessing needs, and the lack of standardized product regulations still hinder widespread adoption. To fully realize the potential of biological waste treatment, enhanced policy support, technological improvements, and institutional innovation are necessary to align economic incentives with environmental goals and progress toward a sustainable, resource-efficient waste management system.

To support a targeted transition, region-specific environmental taxes should be imposed on incineration and landfilling, with revenues directed to subsidize WtM infrastructure in underserved areas. Current subsidies for WtE technologies should be gradually redirected to support WtM pathways, especially bioconversion, using market-based incentives such as feed-in tariffs for bio-products and inclusion in carbon credit systems. National standards and certification systems for compost and insect protein products must be established to build market confidence and support the scaling of secondary resource circulation. These coordinated measures would internalize environmental externalities, correct current market failures, and accelerate the shift toward a circular waste management system.

Policy implementation must fully account for regional heterogeneity. Our analysis reveals substantial variations in waste composition, environmental carrying capacity, and economic development levels across Chinese cities, necessitating place-based policy design. In wealthier and environmentally sensitive eastern cities, integrated RR-HBB-ID strategies should be prioritized. Western cities that still rely on incineration need adjusted environmental taxes to manage transition costs, along with technology transfer and financial support. For central cities facing economic and environmental trade-offs, a flexible transition mechanism could provide a choice between RR-ID and RR-HBB-ID pathways based on local conditions.

Furthermore, we recommend implementing a “transition dynamics management” system, where city-level monitoring and evaluation regularly update strategy portfolios to stay aligned with technological advancements and urban development needs. This precision-targeted, adaptive governance framework would accelerate China’s “zero-waste cities” initiative and provide a replicable policy model for MSW transitions worldwide.

This study has several limitations. First, it does not fully incorporate the economic costs and practical impacts of MSW sorting policies. While some direct costs are included in our operational estimates, modeling the effectiveness of sorting remains difficult due to data limitations and systemic complexities. Second, the analysis assumes a constant MSW composition after 2021 as a practical baseline. This simplifies the assessment of treatment strategy transitions, although waste composition will evolve with socio-economic and policy changes. Developing dynamic predictive models for waste streams will be key to improving long-term strategic planning. Third, heterogeneity in technology, operational cost, and environmental impact among MSW treatment plants remains uncharacterized. Future work will aim to incorporate these factors to improve analytical accuracy.

## Resource availability

### Materials availability

This study did not generate new unique materials.

### Data and code availability

• Source data are all provided with this paper in the supplementary files.
• The code that supports the findings of this study are available from the corresponding author upon reasonable request.

## Funding and Acknowledgments

This study is supported by the 10.13039/501100001809National Natural Science Foundation of China (reference nos. 72321002, 72573021, 72141302, 72104023, 72533002, 72222017, 72504023, and 72243001), the 10.13039/501100003787Natural Science Foundation of Hebei Province of China (reference no. G2025207029), Key Research Projects of Philosophy and Social Sciences of China Ministry of Education (reference no. 21JZD027), Beijing Natural Science Foundation (reference no. 9254036), and the 10.13039/501100002858China Postdoctoral Science Foundation (reference no. 2024M764152).

## Author contributions

Z.W. conceived of and designed the study. F.L., X.L., X.L., Z.L., and S.M. processed and analyzed data. H.L. and B.L. led the writing of the paper. B.G. and C.Z. contributed to the review and revision.

## Declaration of interests

The authors declare no competing interests.

## Supplemental information

Document S1. Figures S1–S11, Tables S1–S13, and Files S1 and S2

Document S2. Article plus supplemental information
