# Supplementary material for: The waste-environmental-economic nexus facilitates city-specific cost-effective transition for China’s municipal solid waste treatment
Source: Innovation (Camb). 2026 Feb 12;7(7):101315. doi: 10.1016/j.xinn.2026.101315 (PMC13343423; doi:10.1016/j.xinn.2026.101315)

The Innovation, Volume 7

Supplemental Information

The waste-environmental-economic nexus facilitates city-specific cost-effective transition for China's municipal solid waste treatment

Hao Li, Xiaolong Lu, Fang Liu, Zhe Li, Xianmei Liu, Bin Lu, Shijun Ma, Baojing Gu, Chuanbin Zhou, and Zhaohua Wang

Supplementary files

Supplementary Text 1 Forecasts of population (POP), per capita gross domestic product (PCGDP) and MSW generation from 2022 to 2050

Projection of POP and PCGDP. The POP and PCGDP data from 2022 to 2050 are the basis for forecasting MSW generation in Chinese cities. Based on the historical POP and PCGDP data covering the period from 1988 to 2021, the autoregressive integrated moving average (ARIMA) model is used to forecast POP and PCGDP from 2022 to 2050 for each Chinese city.

$$POP_t^k = a_1 \cdot POP_{t-1}^k + a_2 \cdot POP_{t-2}^k + \cdots + a_n \cdot POP_{t-n}^k + \varepsilon_t + b_1 \cdot \varepsilon_{t-1} + b_2 \cdot \varepsilon_{t-2} + \cdots + b_m \cdot \varepsilon_{t-m} \quad \text{(S1)}$$

$$\begin{aligned} PCGDP_t^k = {} & a_1 \cdot PCGDP_{t-1}^k + a_2 \cdot PCGDP_{t-2}^k + \cdots + a_n \cdot PCGDP_{t-n}^k \\ & + \varepsilon_t + b_1 \cdot \varepsilon_{t-1} + b_2 \cdot \varepsilon_{t-2} + \cdots + b_m \cdot \varepsilon_{t-m} \end{aligned} \quad \text{(S2)}$$

Where POP_t^k and $PCGDP_t^k$ are the population and per capita gross domestic product, respectively. ε_t is the random error. n and m are integers that are often referred to as autoregressive and moving average, respectively. k and t represent different cities and years, respectively.

Projection of MSW generation. The MSW generation is closely related to socioeconomic indicators, especially population (POP) and per capita gross domestic product (PCGDP). Thus, we use multiple linear regression model to forecast the quantity of MSW generation from 2022 to 2050 for the 292 China's cities with historical POP and PCGDP data, while the autoregressive integrated moving average (ARIMA) model is used for the other 60 cities.

$$Q_{\mathrm{MSW},t}^k = a_{\mathrm{POP}} \cdot POP_t^k + a_{\mathrm{PCGDP}} \cdot PCGDP_t^k + a_0 \quad \text{(S3)}$$

$$Q_{\mathrm{MSW},t}^k = a_1 \cdot Q_{\mathrm{MSW},t-1}^k + a_2 \cdot Q_{\mathrm{MSW},t-2}^k + \cdots + a_n \cdot Q_{\mathrm{MSW},t-n}^k + \varepsilon_t + b_1 \cdot \varepsilon_{t-1} + b_2 \cdot \varepsilon_{t-2} + \cdots + b_m \cdot \varepsilon_{t-m} \quad \text{(S4)}$$

Where $Q_{\mathrm{MSW},t}^k$ is the quantity of MSW generation. POP_t^k and $PCGDP_t^k$ are the population and per capita gross domestic product, respectively. a_{POP}, a_{PCGDP} and a_0 are the regression coefficients. ε_t is the random error. a_n and b_m are the coefficients. n and m are integers that are often referred to as autoregressive and moving average, respectively. k and t represent different cities and years, respectively.

Table 1 Data source and processing of MSW treatment amount

Item	Historical Data (2001–2021)	Future Projections (2022–2050)
Data Source	China Urban and Rural Construction Statistical Yearbook	Projections based on socio-economic indicators (POP, PCGDP)
Coverage	352 prefecture-level cities	352 prefecture-level cities
Prediction Methodology	Actual observed values	Multiple linear regression + ARIMA (for generation amount)<br>Logistic curve + proportional allocation

Item	Historical Data (2001–2021)	Future Projections (2022–2050)
		(for treatment structure)

Notes:historical data on municipal solid waste (MSW) generation across cities from 2000 to 2021 were sourced from the China Urban and Rural Construction Statistical Yearbook (MOHURD, 2021), the China City Statistical Yearbook (NBSC, 2018), as well as related datasets and methodologies from Ma et al. (2020, 2024).

Supplementary Text 2 Calculation for the quantities of MSW disposal and recycling from 2022 to 2050 in various scenarios.

In RR-ID, RR-HBC-ID, and RR-HBB-ID scenarios, recyclable materials in MSW are expected to be collected and recycled as much as possible.

$$Q^k_{\text{recycle},j,t} = \alpha_{\text{recycle},j} \times \rho^k_j \times Q^k_{\text{MSW},t} \tag{S5}$$

$$Q^k_{\text{MSW},t}{}' = Q^k_{\text{MSW},t} - \sum_j Q^k_{\text{recycle},j,t} \tag{S6}$$

Where $Q^k_{\text{recycle},j,t}$ is the quantity of collected recyclable materials. $Q^k_{\text{MSW},t}$ is the quantity of MSW generation. $\alpha_{\text{recycle},j}$ is the collection rate. ρ^k_j is the ratio of recyclable materials to the total quantity of MSW. $Q^k_{\text{MSW},t}{}'$ represents the updated quantity of MSW that needs disposal. j represents different recyclable materials.

The disposal of the remaining MSW is either incineration-dominant (ID), bioconversion disposal-dominant (BD), or still the same as that in 2021. In scenario BAU, the ratios of MSW incineration, landfill, and bioconversion disposal ($\xi^k_{\text{inciner},0}$, $\xi^k_{\text{landfill},0}$, and $\xi^k_{\text{bioche},0}$) are expected to remain consistent with the levels in 2021.

$$\begin{cases} Q^k_{\text{inciner},t} = \xi^k_{\text{inciner},0} \times Q^k_{\text{MSW},t} \\ Q^k_{\text{landfill},t} = \xi^k_{\text{landfill},0} \times Q^k_{\text{MSW},t} \\ Q^k_{\text{bioche},t} = \xi^k_{\text{bioche},0} \times Q^k_{\text{MSW},t} \end{cases} \tag{S7}$$

Where $Q^k_{\text{inciner},t}$, $Q^k_{\text{landfill},t}$, and $Q^k_{\text{bioche},t}$ are the quantity of MSW incineration, landfill, and bioconversion disposal, respectively.

With the incineration-dominant strategy, including ID and RR-ID scenarios, the incineration rate ($\alpha^k_{\text{inciner},t}$) is expected to gradually increase, as detailed in Supplementary Text 5, and nearly all remaining MSW will be combusted by 2050. Then, the ratios of landfill and bioconversion disposal ($\zeta^k_{\text{landfill},0}$ and $\zeta^k_{\text{bioche},0}$) in the remaining MSW are expected to remain consistent with the levels in 2021.

$$Q^k_{\text{inciner},t} = \alpha^k_{\text{inciner},t} \times Q^k_{\text{MSW},t} \tag{S8}$$

$$Q^k_{\text{MSW},t}{}' = Q^k_{\text{MSW},t} - Q^k_{\text{inciner},t} \tag{S9}$$

$$\begin{cases} Q^k_{\text{bioche},t} = \zeta^k_{\text{bioche},0} \times Q^k_{\text{MSW},t} \\ Q^k_{\text{landfill},t} = \zeta^k_{\text{landfill},0} \times Q^k_{\text{MSW},t} \end{cases} \tag{S10}$$

With the bioconversion disposal-dominant strategy, including LBC, LBB, HBC, HBB, HBC-ID, HBB-ID, RR-HBC-ID, and RR-HBB-ID scenarios, the organic

components in MSW will be prioritized for bioconversion disposal.

$$Q^k_{\text{bioche},t} = \alpha^k_{\text{bioche},t} \times \rho^k_{\text{organic}} \times Q^k_{\text{MSW},t} \tag{S11}$$

$$Q^k_{\text{MSW},t}{}' = Q^k_{\text{MSW},t} - Q^k_{\text{bioche},t} \tag{S12}$$

Where ρ^k_{organic} is the ratio of organic components to the total quantity of MSW. $\alpha^k_{\text{bioche},t}$ is the bioconversion disposal rate of organic components. However, the rate of this disposal will be constrained by the advancement of bioconversion disposal technologies in different cities. In LBC and LBB scenarios, the bioconversion disposal rates of organic components in less-developed and developed cities are expected to gradually increase to 30% and 50%, respectively. In contrast, in HBC, HBB, HBC-ID, HBB-ID, RR-HBC-ID, and RR-HBB-ID scenarios, these rates are expected to reach 50% and 100%. The classification of cities as less-developed or developed is based on whether the rate of this disposal there was lower or higher than 1% in 2021.

After the bioconversion disposal of certain organic components, the incineration rate of the remaining MSW is expected to gradually increase in HBC-ID, HBB-ID, RR-HBC-ID, and RR-HBB-ID scenarios, as detailed in equation (S8)-(S9). Then, the final remaining MSW will be landfilled.

$$Q^k_{\text{landfill},t} = Q^k_{\text{MSW},t} \tag{S13}$$

In contrast, in BC, LBB, HBC, and HBB scenarios, the ratios of incineration and landfill ($\psi^k_{\text{landfill},0}$ and $\psi^k_{\text{bioche},0}$) in the remaining MSW are expected to remain consistent with the levels in 2021 after the bioconversion disposal of certain organic components.

$$\begin{cases} Q^k_{\text{inciner},t} = \psi^k_{\text{inciner},0} \times Q^k_{\text{MSW},t} \\ Q^k_{\text{landfill},t} = \psi^k_{\text{landfill},0} \times Q^k_{\text{MSW},t} \end{cases} \tag{S14}$$

Moreover, in LBC, HBC, HBC-ID, and RR-HBC-ID scenarios, traditional compost is utilized for the bioconversion disposal of organic components. In contrast, bioconversion is utilized for this disposal in LBB, HBB, HBB-ID, and RR-HBB-ID scenarios.

Supplementary Text 3 Clarification on MSW incineration, landfill and Bioconversion disposal

MSW Incineration: Incineration not only minimizes the physical space required for MSW disposal but also harnesses energy that can be utilized for electricity generation, receiving a strong endorsement from Chinese government. Nevertheless, a significant amount of fly ash, which contains heavy metals, soluble salts, and dioxins, is produced during the incineration process, posing environmental toxicity concerns [1]. There are two primary technologies for MSW incineration: fluidized bed incineration and grate firing incineration. The calculation of operational benefits for MSW incineration considers these two technologies, whose respective ratios are 7.8% and 92.2%, according to data released by the Ministry of Ecology and Environment of China in June 2023 [2]. The converted grid-connected electricity output per ton of MSW is 280 kWh, and a nationwide uniform benchmark feed-in tariff with subsidies contained is implemented, set at 0.65 CNY/kWh [3], according to the price policy for

waste incineration power generation established by the Chinese National Development and Reform Commission. Moreover, we compiled the gate fees for waste incineration projects across various provinces in China, which ranged from 59.65 to 247.24 CNY/ton.

MSW landfill: Landfill is one of the most common methods for MSW disposal worldwide. While it is cost-effective, it poses risks related to greenhouse gas emissions and environmental pollution [4]. Once landfilled, organic components, as well as paper, textiles, and wood and bamboo in MSW, will gradually degrade and produce landfill gas, which contains a significant amount of methane. Therefore, landfill gas is also an energy resource and can provide benefits [5]. Moreover, we compiled the gate fees for waste landfill projects across various provinces in China, which ranged from 15 to 110 CNY/ton.

MSW bioconversion disposal: bioconversion disposal aims to convert organic components in MSW into valuable by-products, demonstrating better environmental performance compared to incineration and landfill. One of the predominant methods is compost. During the composting phase, the organic components are decomposed under aerobic conditions by microorganisms, resulting in the production of compost. This process promotes a circular economy by recycling organic components in MSW back into the ecosystem [6]. On the other hand, bioconversion has emerged as an innovative and sustainable approach for organic waste management. This process involves the utilization of insects to convert various types of organic waste into high-value protein and biomass, creating new revenue streams for organic waste bioconversion disposal[7]. Moreover, we compiled data on the unit subsidy costs for MSW bioconversion disposal projects from various cities, including Beijing, Chongqing, Shenzhen, Changsha, Harbin, Hohhot, Jinan, Qingdao, Zaozhuang, and Xianyang. These cities represent diverse geographical locations and economic development levels. Subsequently, based on three key indicators (population, GDP per capita, and the quantity of organic components in per capita MSW), we assigned the benchmark subsidy values from these representative cities to other cities with similar socio-economic and waste characteristics. The resulting subsidies for MSW bioconversion disposal across different cities range from 168 to 338.6 CNY/ton.

Supplementary Text 4 Calculation for the monetization factors of LCA indicators

The calculation equations on the monetization factors of LCA indicators are presented below, and the specific explanations refer to [8].

PMFP, FEP, MEP, EOFP, HOFP and TAP:

$$\upsilon_{d,l}^{k} = \frac{1}{q_{d,l}^{k}} \times r_{d,l}^{k} \tag{S15}$$

$$\delta_{d,l}^{k} = \frac{s_{d,l}^{k} \times h_{d,l}^{k}}{\sum\left(s_{d,l}^{k} \times h_{d,l}^{k}\right)} \tag{S16}$$

$$\omega_{l}^{k} = \sum\left(\delta_{d,l}^{k} \times \upsilon_{d,l}^{k}\right) \tag{S17}$$

Where $\upsilon_{d,l}^{k}$ is the monetary intermediate factor. $q_{d,l}^{k}$ is the pollutant equivalent

value. $r_{d,l}^{k}$ is the tax on emissions. $\delta_{d,l}^{k}$ is the influence potential coefficient. $s_{d,l}^{k}$ is the characterization factor. $h_{d,l}^{k}$ is the total emissions amount in 2020. l represents different indicators. d represents the pollutants related to the indicators.

FETP and METP:

$$\omega_{\mathrm{FETP}}^{k} = \frac{r_{\mathrm{FETP}}^{k}}{q_{\mathrm{FETP}}^{k}} \tag{S18}$$

$$\omega_{\mathrm{METP}}^{k} = \omega_{\mathrm{FETP}}^{k} \times \frac{\mathrm{SD}_{\mathrm{marine}}}{\mathrm{SD}_{\mathrm{fresh}}} \times \frac{\mathrm{CF}_{\mathrm{marine}}}{\mathrm{CF}_{\mathrm{fresh}}} \tag{S19}$$

Where $\mathrm{SD}_{\mathrm{marine}}$ is the species density in marine water. $\mathrm{SD}_{\mathrm{fresh}}$ is the species density in fresh water. $\mathrm{CF}_{\mathrm{marine}}$ is the conversion factor from midpoint to endpoint for METP. $\mathrm{CF}_{\mathrm{fresh}}$ is the conversion factor from midpoint to endpoint for FETP.

FFP and SOP:

$$\omega_{\mathrm{SOP}}^{k} = \overline{\mathrm{p}}_{\mathrm{Cu,2020}} \times \mathrm{Tax}_{\mathrm{Cu}}^{k} \tag{S20}$$

$$\omega_{\mathrm{FFP}}^{k} = \overline{\mathrm{p}}_{\mathrm{Oil,2011\text{-}2020}} \times \mathrm{Tax}_{\mathrm{Oil}}^{k} \tag{S21}$$

Where $\overline{\mathrm{p}}_{\mathrm{Cu,2020}}$ and $\overline{\mathrm{p}}_{\mathrm{Oil,2011\text{-}2020}}$ are the average prices of copper and oil, respectively. $\mathrm{Tax}_{\mathrm{Cu}}^{k}$ and $\mathrm{Tax}_{\mathrm{Oil}}^{k}$ are the copper tax on mineral processing and oil tax, respectively.

LU:

$$\mathrm{MSA}_{\mathrm{f}\to\mathrm{c}} = \frac{\left(S_{\mathrm{f}} - S_{\mathrm{c}}\right)}{S_{\mathrm{f}}} \tag{S22}$$

$$\mathrm{MSA}_{\mathrm{g}\to\mathrm{c}} = \frac{\left(S_{\mathrm{g}} - S_{\mathrm{c}}\right)}{S_{\mathrm{g}}} \tag{S23}$$

$$\mathrm{MFR}_{\mathrm{CPI}} = \frac{\mathrm{CPI}_{\mathrm{EU,2021}}}{\mathrm{CPI}_{\mathrm{EU,2020}}} \tag{S24}$$

$$\omega'_{\mathrm{LU,f,2020}} = \rho_{\mathrm{f}} \times \mathrm{MSA}_{\mathrm{f}\to\mathrm{c}} \times \frac{\omega'_{\mathrm{LU,f,2021}}}{10{,}000} \times \mathrm{MFR}_{\mathrm{CPI}} \tag{S25}$$

$$\omega'_{\mathrm{LU,g,2020}} = \rho_{\mathrm{g}} \times \mathrm{MSA}_{\mathrm{g}\to\mathrm{c}} \times \frac{\omega'_{\mathrm{LU,f,2021}}}{10{,}000} \times \mathrm{MFR}_{\mathrm{CPI}} \tag{S26}$$

$$\omega'_{\mathrm{LT,f,2020}} = 0.5 \times \rho_{\mathrm{f}} \times \mathrm{MSA}_{\mathrm{f}\to\mathrm{c}} \times \frac{\omega'_{\mathrm{LT,f,2021}}}{10{,}000} \times \frac{1}{\mathrm{N}_{\mathrm{rel,f}}} \times \mathrm{MFR}_{\mathrm{CPI}} \tag{S27}$$

$$\omega'_{\mathrm{LT,g,2020}} = 0.5 \times \rho_{\mathrm{g}} \times \mathrm{MSA}_{\mathrm{g}\to\mathrm{c}} \times \frac{\omega'_{\mathrm{LT,g,2021}}}{10{,}000} \times \frac{1}{\mathrm{N}_{\mathrm{rel,g}}} \times \mathrm{MFR}_{\mathrm{CPI}} \tag{S28}$$

$$\mathrm{MFR}_{\mathrm{PPP}} = \frac{\mathrm{PPP}_{\mathrm{China,2020}}}{\mathrm{PPP}_{\mathrm{EU,2020}}} \tag{S29}$$

$$\omega_{\mathrm{LU,2020}}^{k} = \left(\sum \omega'_{\mathrm{LT,f,2020}} + \sum \omega_{\mathrm{LT,2020}}^{k}\right) \times \mathrm{MFR}_{\mathrm{PPP}} \times \mathrm{MFR}_{\mathrm{lut}}^{m} \tag{S30}$$

Where $\mathrm{MSA}_{\mathrm{f}\to\mathrm{c}}$ is the mean species abundance when forest is transformed to cropland. S_{f} , S_{c} , S_{f} and are the relative species richness of forest, grassland, and annual cropland, respectively. $\mathrm{MSA}_{\mathrm{g}\to\mathrm{c}}$ is the mean species abundance when grassland is transformed to cropland. $\mathrm{CPI}_{U,t}$ is the consumer price index. $\mathrm{MFR}_{\mathrm{CPI}}$ is the modification factor of consumer price index. $\omega'_{\mathrm{LU,f},t}$ and $\omega'_{\mathrm{LU,g},t}$ are the average monetization factor of LU in China. ρ_{f} and ρ_{g} are the proportions of forest biomes grassland biomes to the total global terrestrial area, respectively. $\omega'_{\mathrm{LT,f},t}$ and $\omega'_{\mathrm{LT,g},t}$ are the monetization factor of land transformation. $\mathrm{N}_{\mathrm{rel,f}}$ and $\mathrm{N}_{\mathrm{rel,g}}$ are the recovery time (years) for species richness of forest biomes and grassland biomes, respectively. $\mathrm{PPP}_{U,t}$ is the purchasing power parity. $\mathrm{MFR}_{\mathrm{PPP}}$ is the modification factor of purchasing power parity. $\mathrm{MFR}_{\mathrm{lut}}^{m}$ is the modification factor of land use tax. U represents different countries.

GWP:

$$\bar{\omega}_{\mathrm{GWP}} = \frac{\sum_{s=1}^{5}\left(\mathrm{AC}_s \times \mathrm{AP}_s\right)}{\sum_{s=1}^{5}\mathrm{AP}_s} \tag{S31}$$

Where $\mathrm{AC}_{\mathrm{sec}}$ is the abated amount of CO_2. $\mathrm{AP}_{\mathrm{sec}}$ is the CO_2 abatement cost. s represents different sectors including five major sectors—construction, cement, power, the iron and steel industry, and the petrochemical industry.

ODP:

$$\chi_{\mathrm{ODP}}^{k} = \frac{\mathrm{Income}^{k}}{\overline{\mathrm{Income}}} \tag{S32}$$

$$\omega_{\mathrm{ODP,2006}} = \frac{\mathrm{Invest}_{\mathrm{total}}}{\mathrm{ODS}_{\mathrm{total}}} \tag{S33}$$

$$\mathrm{MF}_{\mathrm{ozone}}^{k} = \frac{\mathrm{OC}_{\mathrm{ozone}}^{k}}{\overline{\mathrm{OC}}_{\mathrm{ozone}}} \tag{S34}$$

$$\omega_{\mathrm{ODP,2020}}^{k} = \omega_{\mathrm{ODP,2006}} \times \chi_{\mathrm{ODP}}^{k} \times \mathrm{MF}_{\mathrm{ozone}}^{k} \times \frac{\mathrm{CPI}_{2020}}{\mathrm{CPI}_{2006}} \tag{S35}$$

Where χ_{ODP}^{k} is the economic modifying factor. Income^{k} is the per capita income. $\overline{\mathrm{Income}}$ is the average per capita income in China. $\mathrm{Invest}_{\mathrm{total}}$ is the total investment in ozone-depleting substances abatement. $\mathrm{ODS}_{\mathrm{total}}$ is the total reduction amount of ozone-depleting substances. $\mathrm{MF}_{\mathrm{ozone}}^{k}$ is the modification factor of ODP. $\mathrm{OC}_{\mathrm{ozone}}^{k}$ is the ozone concentration. $\overline{\mathrm{OC}}_{\mathrm{ozone}}$ is the average ozone concentration in China.

HT_c and HT_{nc}:

$$V_{2020} = \frac{\mathrm{CPI}_{2020}}{\mathrm{CPI}_{2018}} \times V_{2018} \tag{S36}$$

$$\omega_{\mathrm{HT}}^{k} = \sum V_{2020} \times \mathrm{CF}_{\mathrm{(non)cancer}} \times \chi_{\mathrm{HT}}^{k} \tag{S37}$$

Where V_t is the value of life expectancy of Chinese residents. $\mathrm{CF}_{\mathrm{(non)cancer}}$ is the conversion factor.

TETP:

$$\text{Intense}^k = \frac{\text{Consume}_{\text{ph}}^k}{\text{Area}_{\text{sown}}^k} \times \frac{\text{M}_{\text{ph}}^k \times 2}{\text{M}_{\text{P}_2\text{O}_5}^k} \quad \text{(S38)}$$

$$\text{MFR}_{\text{intensity}}^k = \frac{\text{Intense}^k}{\overline{\text{Intense}}} \quad \text{(S39)}$$

$$\omega_{\text{TETP}}' = \omega_{\text{TETP,Stepwise}} \times \frac{\text{CPI}_{\text{EU,2019}}}{\text{CPI}_{\text{EU,2020}}} \times \frac{\text{PPP}_{\text{China,2020}}}{\text{PPP}_{\text{EU,2020}}} \quad \text{(S40)}$$

$$\omega_{\text{TETP}}^k = \sum \omega_{\text{TETP}}' \times \text{MFR}_{\text{intensity}}^k \times \chi_{\text{TETP}}^k \quad \text{(S41)}$$

Where Intense^k is the application intensity of phosphorus. $\text{Consume}_{\text{ph}}^k$ is the consumption amount of phosphate. $\text{Area}_{\text{sown}}^k$ is the total sown area of crops. M_{ph}^k and $\text{M}_{\text{P}_2\text{O}_5}^k$ are the molecular weight of phosphorus and phosphorus pentoxide, respectively. $\text{MFR}_{\text{intensity}}^k$ is the modification factor of fertilization intensity $\overline{\text{Intense}}$ is the average application intensity of phosphorus in China. ω_{TETP}' is the average monetization factor for TETP in China. $\omega_{\text{TETP,Stepwise}}$ is the monetization factor of TETP for the Stepwise model in 2019.

IRP:

$$\text{MFR}^k = \frac{\text{DR}^k}{\overline{\text{DR}}} \quad \text{(S42)}$$

$$\omega_{\text{IR}}^k = \text{IR}_{\text{Ecotax}} \times \frac{\text{CPI}_{\text{EU,2020}}}{\text{CPI}_{\text{EU,2019}}} \times \frac{\text{PPP}_{\text{China,2020}}}{\text{PPP}_{\text{EU,2020}}} \times \text{MFR}^k \times \chi_{\text{IR}}^k \quad \text{(S43)}$$

Where DR^k is the air absorption dose rate of γ-rays. $\overline{\text{DR}}$ is the average air absorption dose rate of γ-rays in China. $\text{IR}_{\text{Ecotax}}$ is the average monetization factor of IR in Ecotax 2006 in Switzerland.

Supplementary Text 5 Increase trend in the incineration rates from 2022 to 2050 in ID, HBC-ID, HBB-ID, RR-ID, RR-HBC-ID, and RR-HBB-ID scenarios

The quantity of MSW incineration in each city follows the logistic curve and grows close to the quantity of MSW generation over time. Notably, as of 2021, there remain 93 cities in China with an incineration rate of 0, with nearly 70% located in the Northwest China, Southwest China and Central China. It is assumed that the incineration rates in these cities will reach the average level of the provinces in which they are located in the future.

$$Q_{\text{inciner},t}^k = \frac{\exp(\beta_0 + \beta_1 \cdot T)}{1 + \exp(\beta_0 + \beta_1 \cdot T)} \times Q_{\text{MSW},t}^k \quad \text{(S44)}$$

Where β_0 and β_1 are the constants. T denotes the year.

Supplementary Text 6 Review on waste management policies in China

Waste management is not only crucial for environmental protection and urban sanitation but also an essential component of the circular economy. Through proper waste sorting and treatment, resource waste can be effectively reduced, enabling resource regeneration and recycling, contributing to building a resource-saving and environmentally friendly society, and promoting harmonious economic and

environmental development.

China's waste management policies have evolved from initial response to systematic promotion and comprehensive deepening, gradually achieving a development path from disposal to waste sorting and refined management, as detailed in Supplementary Table 12. Based on this process, China's waste management policies show three main trends:

(1) Disposal methods have shifted from primarily sanitary landfills to incineration, encouraging the development of waste-to-energy incineration.

Waste incineration is a crucial technological choice for waste resource utilization and greenhouse gas reduction. Since the 12th Five-Year Plan, the National Development and Reform Commission has clearly set development targets for waste incineration plants and related treatment capacity in national urban waste treatment planning, promoting the rapid development of China's waste incineration industry. China's municipal waste incineration volume has maintained rapid growth over the past decade, with incineration treatment volume exceeding landfill volume for the first time in 2019 in terms of proportion of total harmless treatment. The volume increased from 38.76 million tons in 2012 to 232 million tons in 2022, with the proportion of urban waste incineration in harmless treatment reaching 75% by 2022, achieving the 14th Five-Year Plan target.

On the other hand, in August 2023, the National Development and Reform Commission, Ministry of Finance, and National Energy Administration issued the Notice on Implementing Comprehensive Green Power Certificate Coverage to Promote Renewable Energy Power Consumption, which proposed improving green certificate trading and achieving full coverage of renewable energy power through green certificates. This marks the beginning of waste incineration green certificate trading. Through green certificate trading, waste-to-energy enterprises can obtain additional revenue streams, thereby improving their economic benefits. This incentive mechanism can attract more enterprises to participate in waste incineration treatment, alleviate financial pressure in waste treatment, and accelerate the construction and operation of waste incineration facilities.

(2) Waste sorting has been gradually implemented from pilot areas to wider coverage.

After rapid growth during the 13th Five-Year Plan period, the solid waste industry needs efficient, high-quality development, with waste sorting being a key approach to improving waste resource utilization efficiency. In addition to national-level policies, provinces and cities have also successively issued relevant policies to promote the development of municipal waste treatment industry. Currently, 21 provinces (autonomous regions) and 173 cities have issued local regulations and government rules on waste sorting. Forty-six key cities have taken the lead in establishing relatively complete waste sorting systems for collection, transportation, and disposal, as detailed in Supplementary Table 13. The coverage rate of waste sorting in residential communities in cities at prefecture level and above has reached 92.6% [9].

(3) Household food waste management has become stricter, with increased focus on resource utilization.

Household food waste refers to easily perishable organic waste discarded in daily household life, such as fruits and vegetables, food trimmings, leftover food, and fruit peels [10]. It is a major component of household waste in China. Household food waste is characterized by high water content, high organic content, and easy degradability [11]. During urban waste collection and treatment, it can easily contaminate other components, hindering the recovery of valuable materials. Therefore, separate treatment of Household food waste is crucial for reducing mixed waste contamination and improving resource recovery rates.

In 2010, the National Development and Reform Commission and other departments launched 100 pilot cities to promote Household food waste resource utilization and harmless treatment. Since 2019, as various regions accelerated mandatory waste sorting, Shanghai, Beijing, Guangdong, and Fujian have successively issued detailed regulations on Household food waste management. These regulations specify requirements for waste reduction, unified collection and disposal, supervision, and resource utilization. Compared to other types of waste, Household food waste collection has higher standards and stricter inspection requirements to ensure safe, efficient, environmentally friendly treatment and resource utilization.

In conclusion, comprehensive management and high-quality development of urban municipal waste has become a new development trend in China. With accelerating urbanization and improving living standards, urban waste output continues to increase. This poses higher requirements for waste treatment, compelling national and local governments to adopt more efficient and environmentally friendly treatment measures. First, waste sorting has become a crucial part of urban management. Through source separation and refined management, it effectively reduces the difficulty and cost of waste treatment. Second, various regions are actively introducing advanced waste treatment technologies, such as waste-to-energy incineration and kitchen waste resource utilization, improving waste treatment efficiency and resource utilization rates. Furthermore, continuous improvement of policies and regulations, along with rising public environmental awareness, has promoted the establishment and optimization of comprehensive waste management systems. Looking ahead, the goal of high-quality development includes not only achieving harmless waste treatment but also maximizing resource utilization and sustainable environmental protection. Optimizing waste treatment structure and improving municipal waste resource utilization levels will become key industry directions.

Supplementary Text 7 Definition of Environmental Impact Indicators

Impact Category	Indicator Name (Full Name & Abbreviation)	Definition	Unit
Climate Change	Global Warming Potential (GWP)	Measures the contribution of greenhouse gas emissions to global warming over a specified time horizon.	kg CO_2 eq.

**Impact Category**	**Indicator Name (Full Name & Abbreviation)**	**Definition**	**Unit**
Ozone Depletion	Ozone Depletion Potential (ODP)	Quantifies the potential of emissions to destroy the stratospheric ozone layer.	kg CFC-11 eq.
Air Quality (Human Health)	Particulate Matter Formation Potential (PMFP)	Measures the potential of emissions to form particulate matter (PM2.5), which affects human respiratory health.	kg PM2.5 eq.
Air Quality (Ecosystems)	Photochemical Oxidant Formation Potential - Ecosystems (EOFP)	Indicates the potential of emissions to form ground-level ozone (smog), harming ecosystem health.	kg NOx eq.
Air Quality (Human Health)	Photochemical Oxidant Formation Potential - Human Health (HOFP)	Indicates the potential of emissions to form ground-level ozone, affecting human health.	kg NOx eq.
Radiation	Ionizing Radiation Potential (IRP)	Measures the impact of emissions that release ionizing radiation on human health and the environment.	kBq Co-60 eq.
Water Eutrophication	Freshwater Eutrophication Potential (FEP)	Assesses the potential of nutrient emissions to cause over-enrichment and oxygen depletion in freshwater bodies.	kg P eq.
Water Eutrophication	Marine Eutrophication Potential (MEP)	Assesses the potential of nutrient emissions to cause over-enrichment in marine ecosystems.	kg P eq.
Ecotoxicity	Freshwater Ecotoxicity Potential (FETP)	Evaluates the long-term potential harm of toxic substances on freshwater ecosystems.	kg 1,4-DCB eq.
Ecotoxicity	Marine Ecotoxicity Potential (METP)	Evaluates the long-term potential harm of toxic substances on marine ecosystems.	kg 1,4-DCB eq.
Resource Consumption	Water Consumption Potential (WCP)	Quantifies the impact of freshwater consumption on water resource availability.	m^3 water eq.
Terrestrial Acidification	Terrestrial Acidification Potential (TAP)	Measures the potential of emissions to acidify soils and harm terrestrial ecosystems.	kg SO_2 eq.
Ecotoxicity	Terrestrial Ecotoxicity Potential (TETP)	Evaluates the long-term potential harm of toxic substances on soil organisms and terrestrial ecosystems.	kg 1,4-DCB eq.

**Impact Category**	**Indicator Name (Full Name & Abbreviation)**	**Definition**	**Unit**
Land Use	Land Use (LU)	Assesses the impact of occupying or transforming land for activities like waste disposal facilities.	m^{2}a crop eq.
Human Toxicity	Human Toxicity Potential - cancer (HTc)	Estimates the potential risk of exposure to toxic emissions causing carcinogenic effects.	kg 1,4-DCB eq.
Human Toxicity	Human Toxicity Potential - non-cancer (HTnc)	Estimates the potential risk of exposure to toxic emissions causing non-carcinogenic health effects.	kg 1,4-DCB eq.
Resource Scarcity	Fossil Resource Scarcity Potential (FFP)	An indicator of the depletion of abiotic fossil resources (e.g., oil, coal).	kg oil eq.
Resource Scarcity	Mineral Resource Scarcity Potential (SOP)	An indicator of the depletion of abiotic mineral resources (e.g., copper, iron ores)	

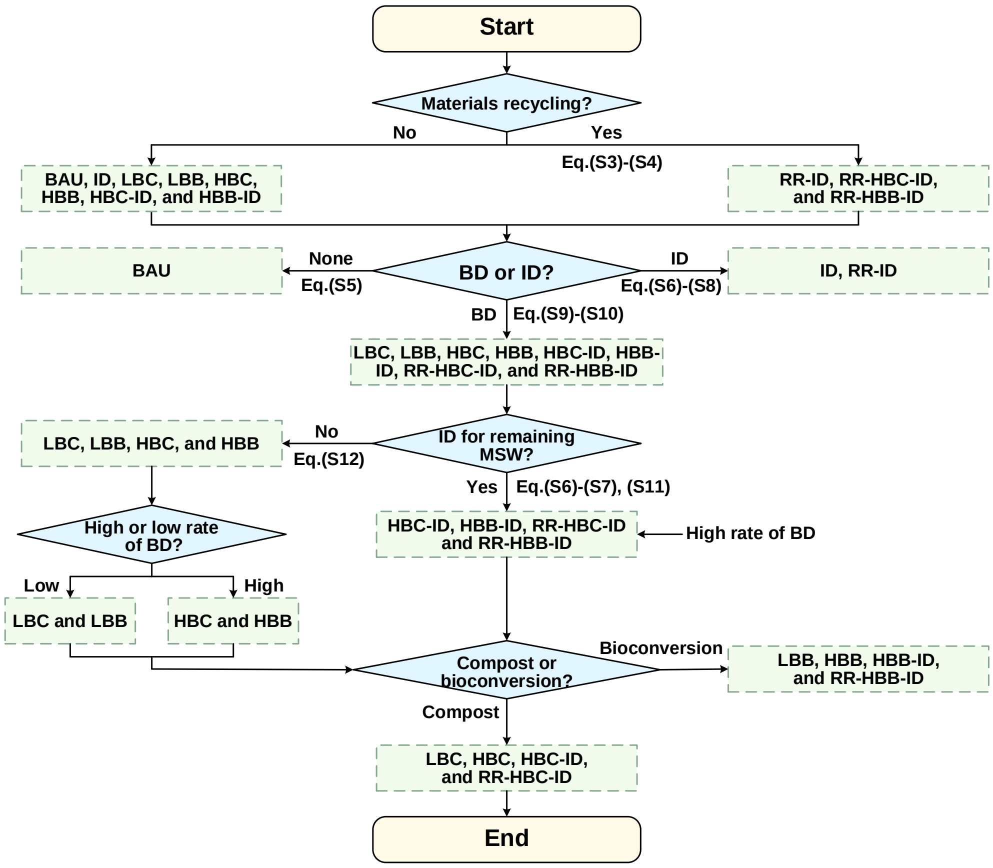

Supplementary Fig. 1 Logic diagram for the calculation of the MSW disposal structures in various scenarios. In order to promote the high value utilization of MSW, the priority for bioconversion disposal of organic components and recycling of recyclable materials will be higher than incineration and landfill in all scenarios. Therefore, in RR-ID, RR-HBC-ID, and RR-HBB-ID scenarios, a fixed ratio of recyclable materials in MSW, including paper, plastics, glass, textiles, and metals, will be recycled before incineration and landfill. And in LBC, LBB, HBC, HBB, HBC-ID, HBB-ID, RR-HBC-ID, and RR-HBB-ID scenarios, an increasing rate of organic components in MSW will be biochemically disposed before incineration and landfill. Excluding the above two measures, if the incineration-dominated MSW disposal mode is adopted, most of the remaining MSW will be combusted by 2050 with an increasing incineration rate. Otherwise, the remaining MSW will be combusted and landfilled according to the ratios of the two MSW disposal methods in 2021. ID: incineration-dominated; BD: bioconversion disposal-dominated.

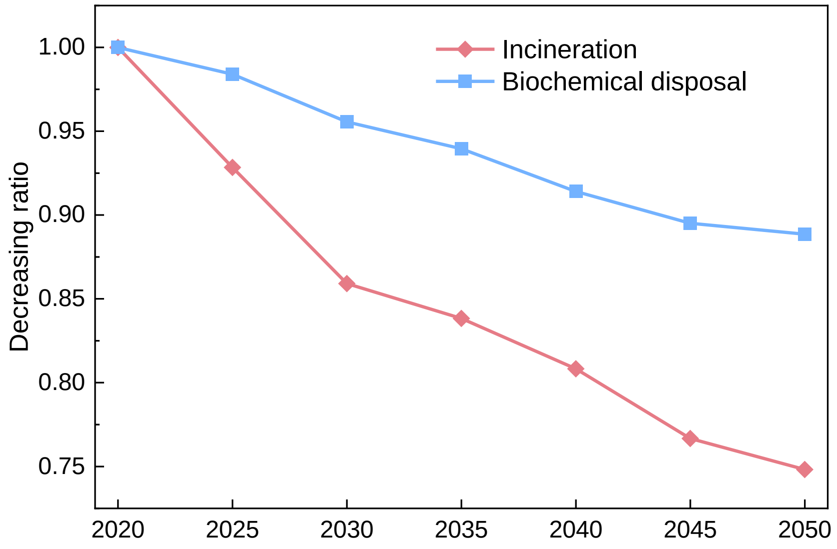

Supplementary Fig. 2 Decreasing ratios of the investment costs for MSW incineration and bioconversion disposal. It reflects the advancements in MSW incineration and bioconversion disposal technologies, resulting in a reduction of investment costs, as well as operation and management costs for these two methods of MSW disposal whose initial values in 2022 refer to [12]. The declining trends are calculated respectively based on the learning curves for gas turbine technology and bioconversion disposal technology, as presented in [13].

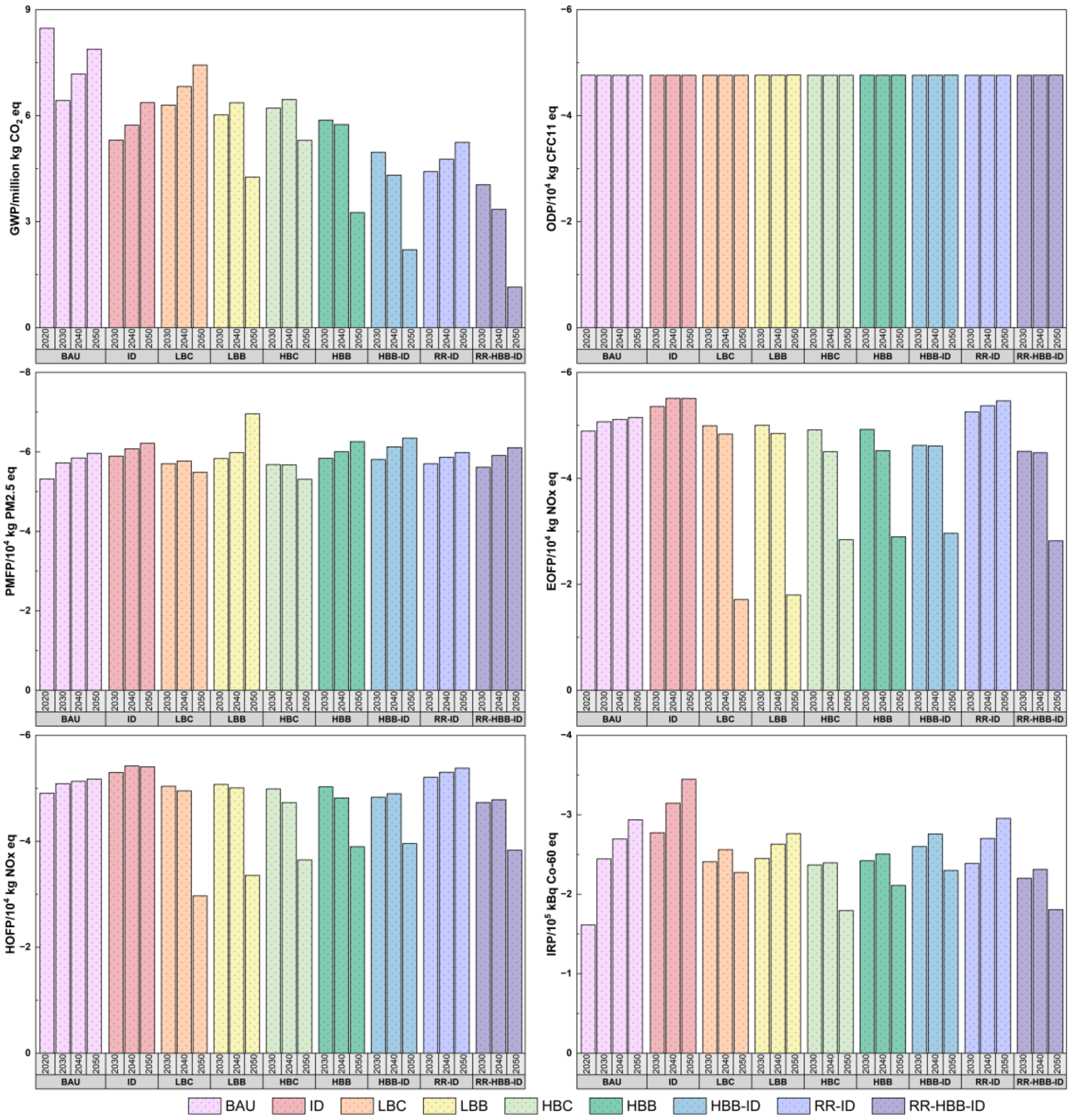

(a) Air-related impacts of MSW treatment under different scenarios

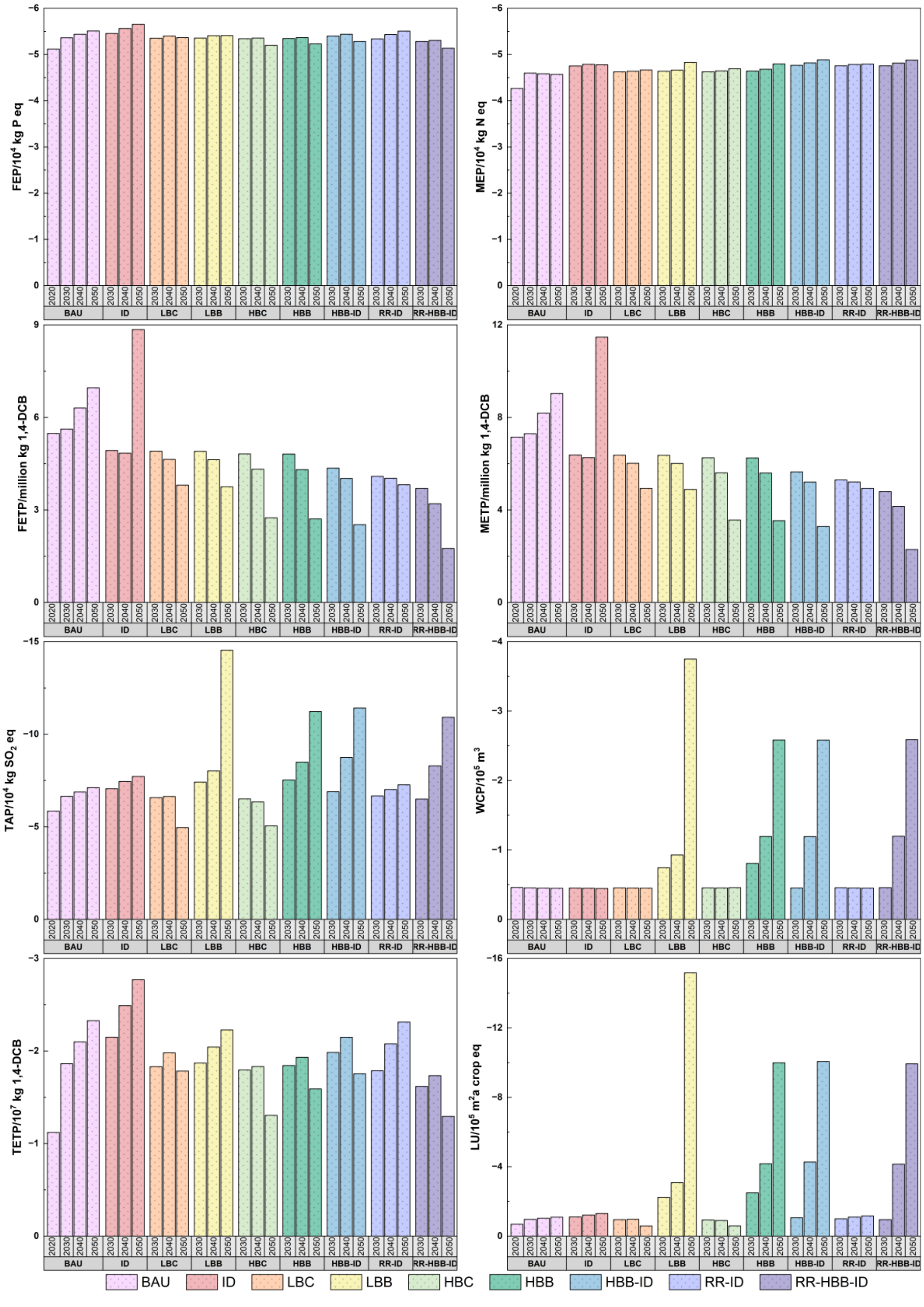

(b) Water-related and soil-related impacts of MSW treatment under different scenarios

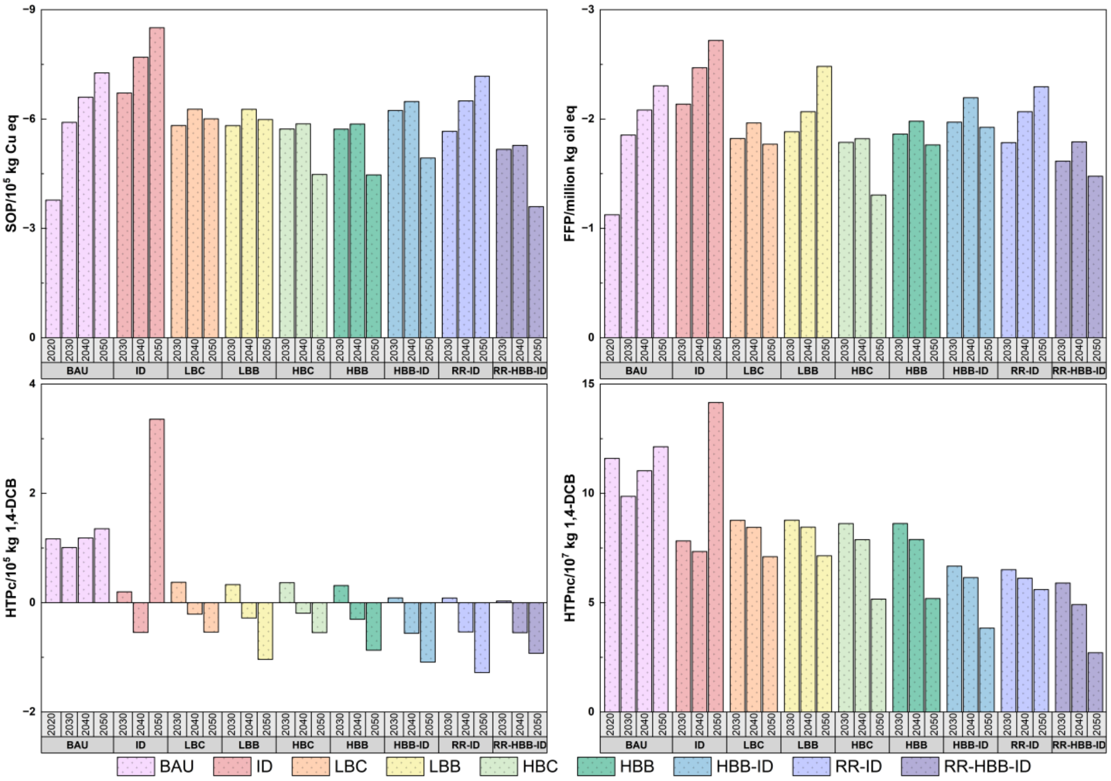

(c) Resource-depletion-related and human-related environmental impacts of MSW treatment under different scenarios

Supplementary Fig. 3 Environmental impacts of MSW treatment under different scenarios. (a)-(c) display the air-related impacts, water-related and soil-related impacts, and resource-depletion-related and human-related environmental impacts, respectively in 2020, 2030, 2040 and 2050 under nine different scenarios. These scenarios are as follows: BAU (Business-as-usual) maintains the same ratio of each disposal method as in 2021. ID (Incineration-dominated) features a gradual increase in the incineration rate until nearly all MSW is combusted by 2050. LBB (Low-rate bioconversion for food waste) and LBC (Low-rate composting for food waste) involve a low proportion of organic components biochemically disposed with compost and bioconversion, respectively, while the remaining waste are disposed in line with BAU scenario. HBC (High-rate composting for food waste) and HBB (High-rate bioconversion for food waste) entail a high proportion of organic components biochemically disposed with compost and bioconversion, respectively while the remaining waste are disposed in line with BAU scenario. HBB-ID (High-rate bioconversion for organic components + Incineration-dominated for the remaining) combines a high proportion of organic components biochemically disposed with the remaining MSW incinerated. RR-ID (Recycling + Incineration-dominated) incorporates the recycling of recyclable materials along with the remaining MSW incinerated. and RR-HBB-ID (Recycling + High-rate bioconversion + Incineration-dominated) integrates recycling, high-proportion bioconversion of organic components, and ID-based disposal of the remaining MSW.

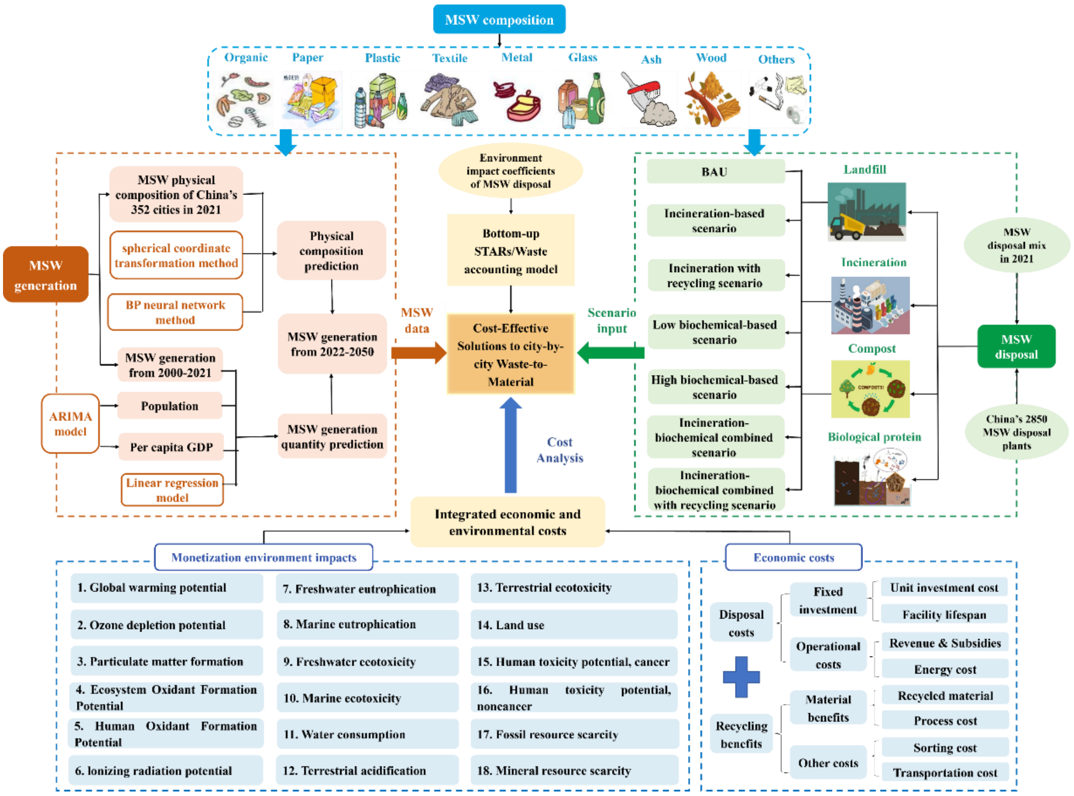

Supplementary Fig. 4 Framework of this study

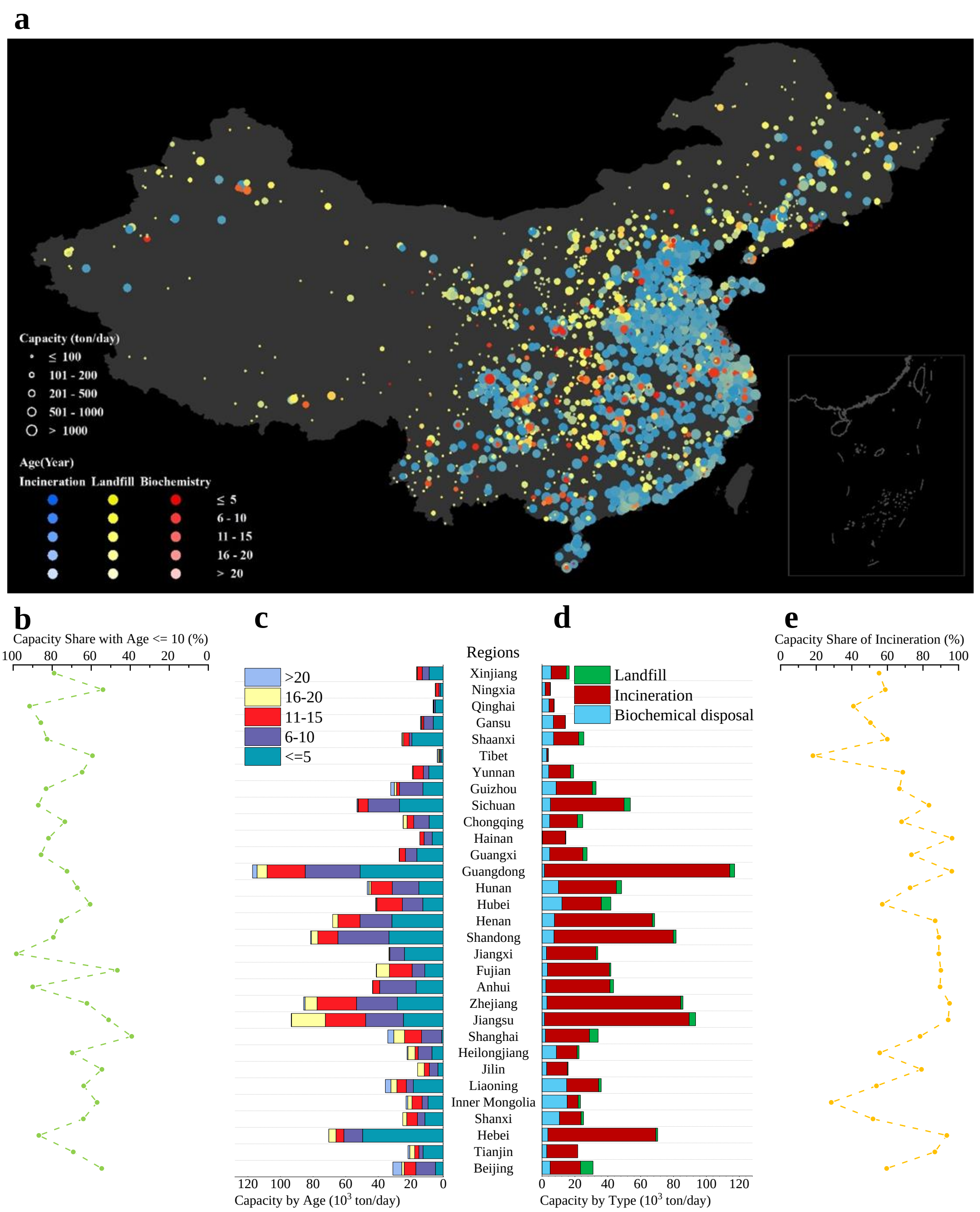

Supplementary Fig. 5 Geographical locations, operation-years, capacities, and disposal modes of MSW disposal plants in China in 2023. a. Location, capacity, age and technologies of China's existing MSW disposal plants in 2023. It shows geographical position, maximum daily disposal quantity and operation-years of the plants. The size of the dots indicates their disposal capacity of the plants, and the color of the dots shows MSW disposal technologies in which the changes of hue represent the operation-years from dark (young plants) to light (old plants). b-e. Capacity share of existing MSW disposal plants in China in 2023, by region, technology and the operation-years. b, e. The curves show capacity share of young plants (operation-years ⩽ 10) and incineration, respectively. c, d. The bars represent the distribution of disposal capacity across regions in China, categorized by operation-years (years since commissioning) and disposal modes, which are indicated by the colors of the bars, respectively.

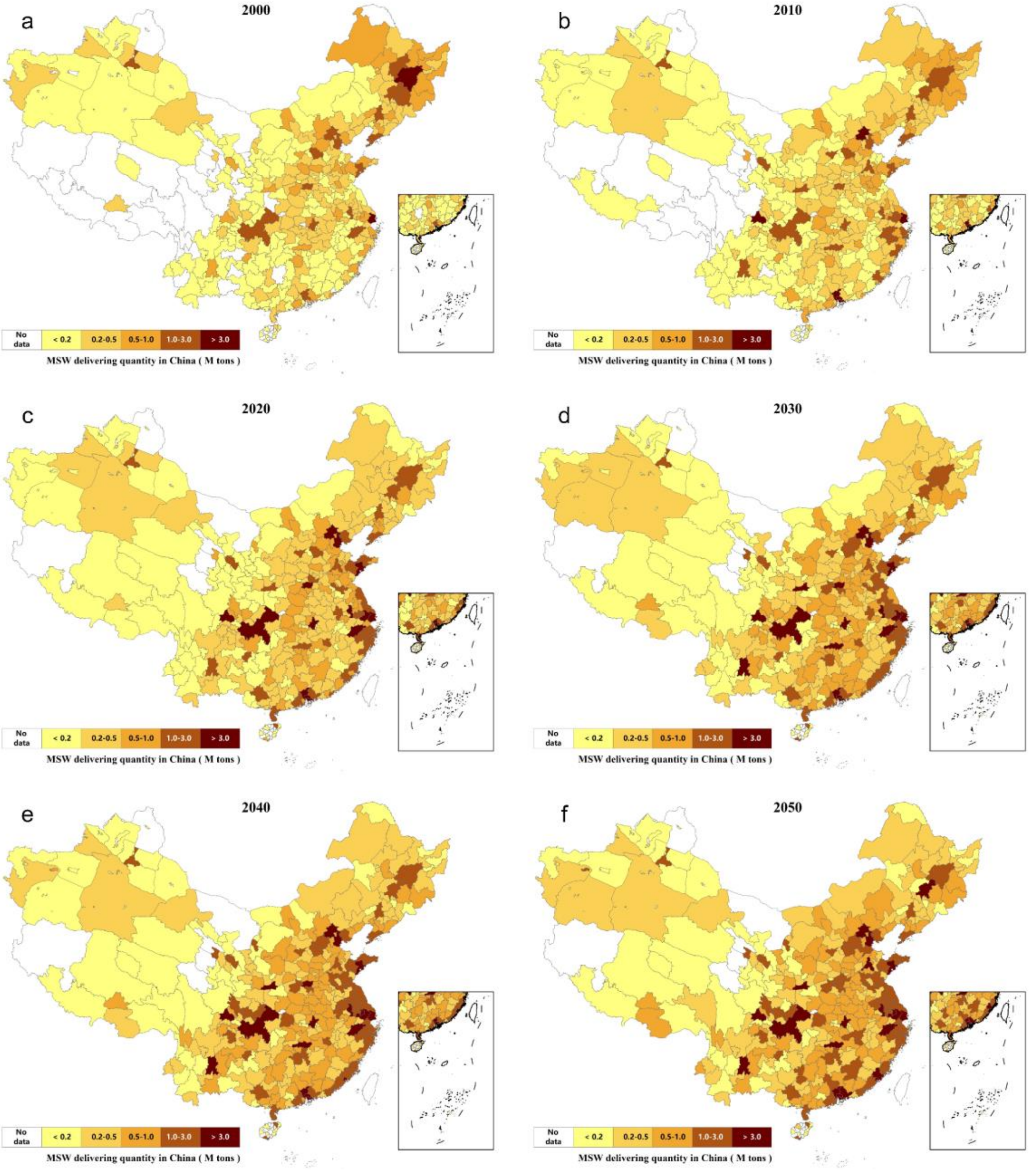

Supplementary Fig. 6 City-level MSW delivering quantity from 2000 to 2050. a-c. The historical MSW delivering quantities of 352 cities in China for the years of 2000, 2010, and 2020. d-f. The forecasted MSW delivering quantities for the years of 2030, 2040, and 2050. The variations in hues represents the MSW delivering quantities of those cities, ranging from lighter shades (lower MSW delivering quantity) to darker shades (higher MSW delivering quantity). The MSW delivering quantities of Laiwu, Qianxinan Autonomous Prefecture, and Yanglingqu are respectively added to those of Jinan, Qianxinan Buyi and Miao Autonomous Prefecture, and Xianyang, considering the changes in the administrative divisions of those areas.

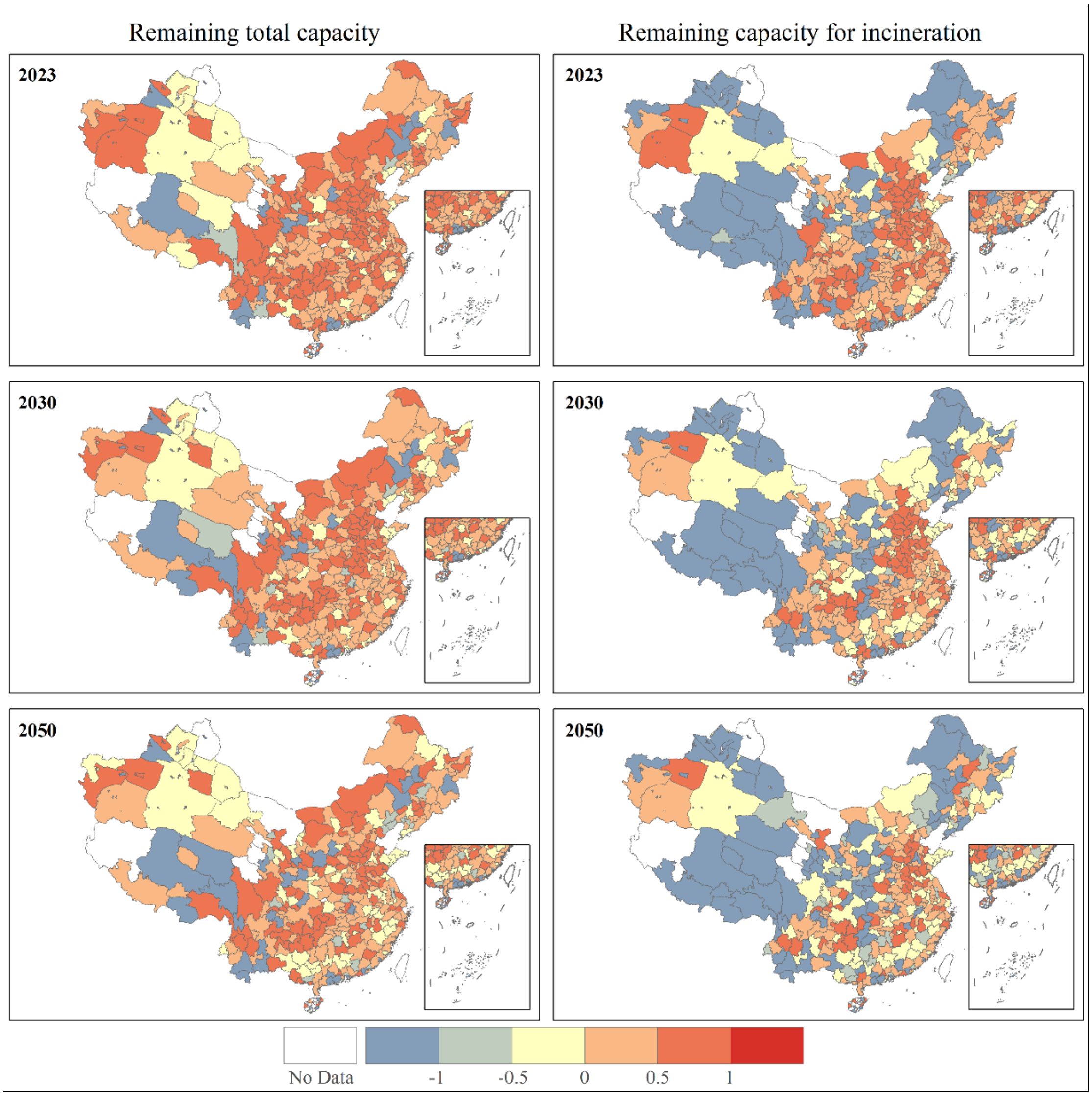

Supplementary Fig. 7 Remaining MSW disposal capacities across cities in ID mode by 2050. a, c, e. Remaining capacities in MSW disposal after subtracting the MSW delivering quantities in ID mode in 2023, 2030, and 2050 from the existing capacities. b, d, f. Remaining capacities in MSW incineration after subtracting the MSW incineration quantities in mode in 2023, 2030, and 2050 from the existing capacities.

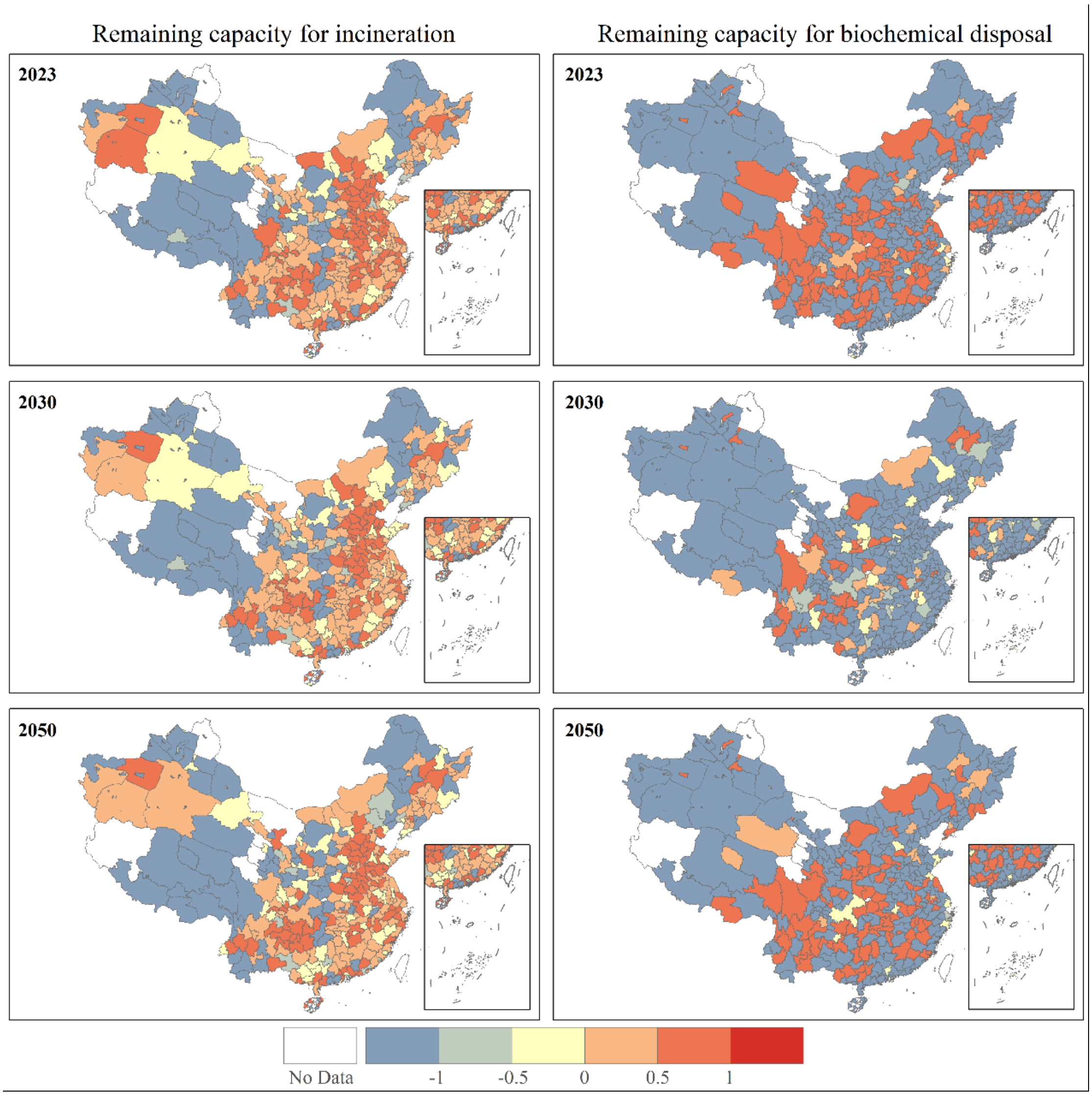

Supplementary Fig. 8 Remaining MSW disposal capacities across cities in LB mode by 2050. a, c, e. Remaining capacities in MSW incineration after subtracting the MSW incineration quantities in LB mode in 2023, 2030, and 2050 from the existing capacities. b, d, f. Remaining capacities in MSW bioconversion disposal after subtracting the quantities of MSW bioconversion disposal in LB mode in 2023, 2030, and 2050 from the existing capacities.

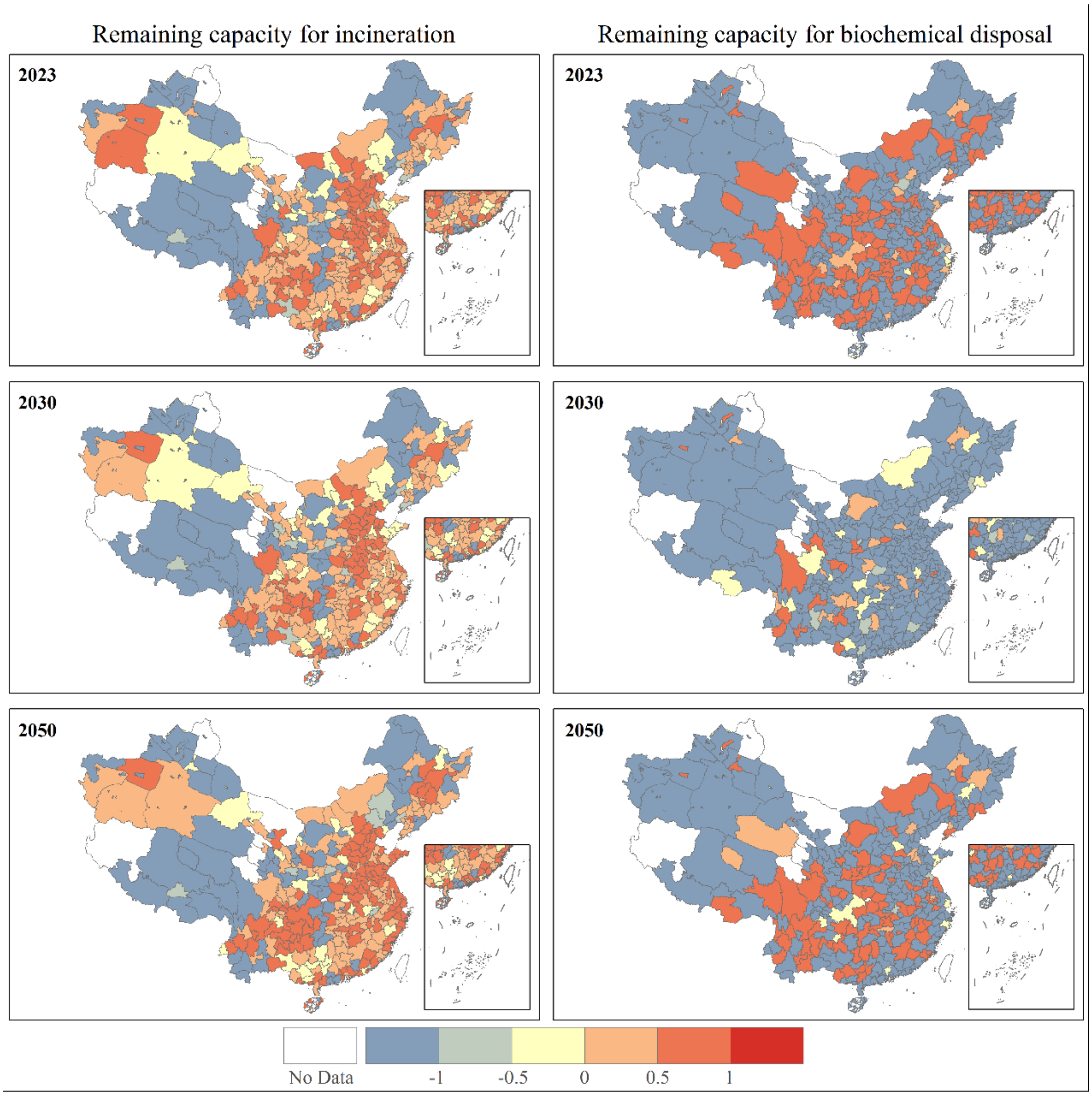

Supplementary Fig. 9 Remaining MSW disposal capacities across cities in HB mode by 2050. a, c, e. Remaining capacities in MSW incineration after subtracting the MSW incineration quantities in HB mode in 2023, 2030, and 2050 from the existing capacities. b, d, f. Remaining capacities in MSW bioconversion disposal after subtracting the quantities of MSW bioconversion disposal in HB mode in 2023, 2030, and 2050 from the existing capacities.

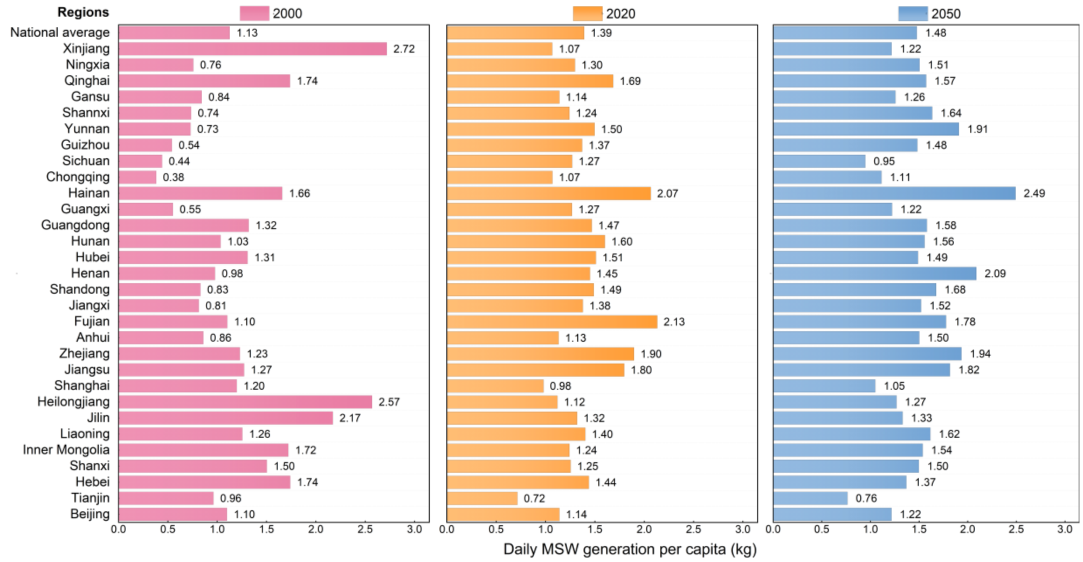

Supplementary Fig. 10 MSW generation per capita in different regions. The results for the years 2000 and 2020 are calculated based on historical data regarding population and MSW delivering quantities in various regions, while the results for the year 2050 are calculated based on the forecasts of future population and MSW delivering quantities. It is noted that we use the MSW delivering quantity to represent the MSW generation.

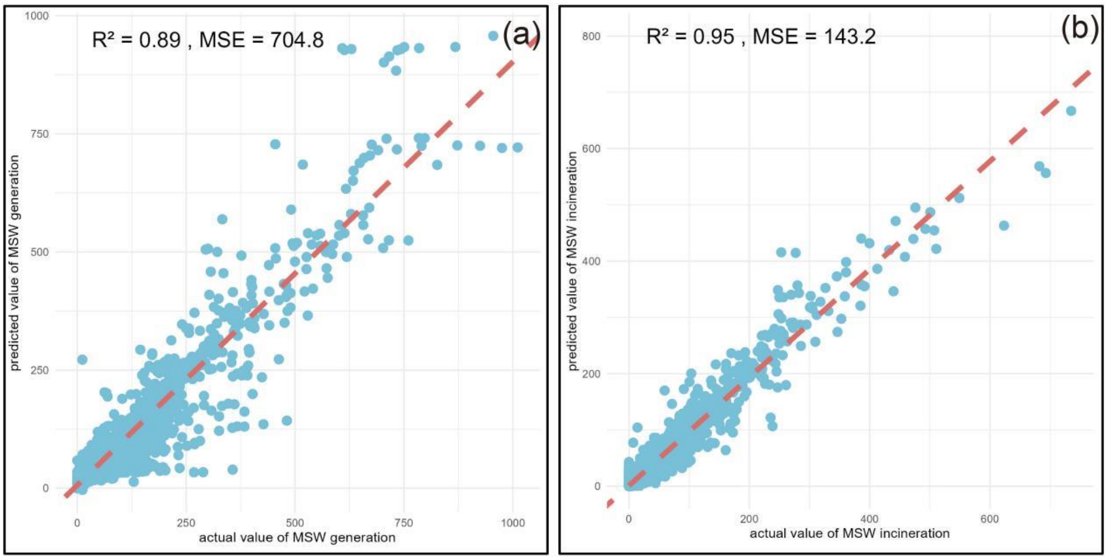

Supplementary Fig. 11 The comparison of predicted value and actual value for MSW generation (a) and MSW incineration (b). Note: the number of data is 7392.

Supplementary Table 1 Distribution of MSW disposal plant capacity based on different operation years

Regions	<=5	5-10	11-15	15-20	>20
**Anhui**	16756	22410	4264	0	85
**Beijing**	4900	12050	7091	1550	5440
**Chongqing**	8800	9420	4084	2330	150
**Fujian**	11431	7780	13960	7850	200
**Gansu**	6233.6	5855.8	1196	423	375
**Guangdong**	51140	33700	23378	6188	2750
**Guangxi**	16308	7020	3691	200	0
**Guizhou**	12560	14351.3	1850	1450	2200
**Hainan**	6180	5700	2625	0	0
**Hebei**	55680	14260	4825	4370	50
**Heilongjiang**	7048	8504.5	1870	4351	530
**Henan**	31629	19555	13570	3220	0
**Hubei**	12645.7	12574	15618.8	399	400
**Hunan**	15063	16330	13083	1185	1100
**Inner Mongolia**	9437.6	3644.6	6221	2549.8	1100
**Jiangsu**	24555	23174	24740	10750	200
**Jiangxi**	23920.5	9012.5	150	393.5	0
**Jilin**	3396	5242	3135	4070	0
**Liaoning**	18476.21	4296	5790	3670	3429.3
**Ningxia**	1859	861.6	2000	320	0
**Qinghai**	4927.37	882.87	270	203.05	60
**Shandong**	33429.2	31322	12336.9	3900	560
**Shanghai**	1100	12300	10500	6587	3650
**Shanxi**	19346.6	1648.7	3531	823	60
**Sichuan**	26983.8	19253	6009	265	550
**Tianjin**	12500	2550	2600	3000	1100
**Tibet**	1463	807	263	704	587
**Xinjiang**	8779.9	4211	2950	460	38
**Yunnan**	8997	3340	6154	464.2	120
**Zhejiang**	28320	25020	24200	7377	825
**National**	336159.7	234879.7	151141.8	54949.3	18009.3

Supplementary Table 2 Average operation years of MSW disposal plants in different regions

Regions	Incineration	Landfill	bioconversion disposal	Total
**Anhui**	8.1	2.7	-	7.8
**Beijing**	8.8	18.0	11.8	11.4
**Chongqing**	4.1	13.3	-	9.0
**Fujian**	6.8	8.8	5.5	8.0
**Gansu**	8.4	-	-	8.4
**Guangdong**	9.1	9.3	6.6	8.8
**Guangxi**	9.6	8.0	8.5	9.3
**Guizhou**	6.2	-	7.3	6.3
**Hainan**	9.5	-	-	9.5
**Hebei**	5.1	17.3	9.0	6.9
**Henan**	7.7	12.3	6.3	10.0
**Heilongjiang**	4.3	10.3	4.5	6.3

**Hubei**	6.7	6.8	10.0	7.0
**Hunan**	7.5	10.0	7.3	7.9
**Jilin**	8.4	12.7	5.3	8.4
**Jiangsu**	4.4	11.4	8.5	10.0
**Jiangxi**	8.6	10.0	9.0	9.7
**Liaoning**	11.5	12.5	21.7	14.0
**Inner Mongolia**	3.7	-	7.0	4.1
**Ningxia**	15.7	-	-	15.7
**Qinghai**	12.0	-	-	12.0
**Shandong**	4.9	9.8	8.8	8.6
**Shanxi**	13.4	17.0	8.5	13.2
**Shaanxi**	6.5	7.0	6.0	6.7
**Shanghai**	3.0	12.9	-	7.9
**Sichuan**	8.2	5.5	6.8	7.8
**Tianjin**	9.2	21.0	9.4	11.1
**Tibet**	4.0	-	-	4.0
**Xinjiang**	4.1	-	-	4.1
**Yunnan**	6.1	12.8	-	7.8
**Zhejiang**	7.2	6.9	7.7	7.0
**National**	6.6	9.4	7.6	8.0

Supplementary Table 3 Parameters and inventory data for MSW fluidized bed incineration and grate firing incineration

**Fluidized bed incineration**		**Grate firing incineration**	
**Input**			
Coal (kg · ton MSW^{-1})	96.6	Diesel (kg · ton MSW^{-1})	1.5
Lime (kg · ton MSW^{-1})	9.92	Lime (kg · ton MSW^{-1})	11.9
Activated carbon (kg · ton MSW^{-1})	0.46	Activated carbon (kg · ton MSW^{-1})	0.23
**Output**			
Electricity (kWh · ton MSW^{-1})	239.4	Electricity (kWh · ton MSW^{-1})	312
Fly ash [a] (kg · ton MSW^{-1})	81.6	Fly ash [b] (kg · ton MSW^{-1})	29.84
Bottom ash (kg · ton MSW^{-1})	114	Bottom ash (kg · ton MSW^{-1})	179.13

The Parameters and inventory data for MSW incineration refer to [12]. a. The disposal cost of fly ash is included with the other costs for MSW incineration, which amounts to 38.8 CNY per ton in this study.

Supplementary Table 4 Parameters and inventory data for MSW landfill

**Input**			
HDPE (kg · ton MSW^{-1})	2.47	Diesel (kg · ton MSW^{-1})	0.43
Leachate volume (L · ton MSW^{-1})	0.42	Electricity (kWh · ton MSW^{-1})	30
**Output**			

Landfill gas collection rate (%)	56.7	CH_4 content in Landfill gas (%)	52.2
Landfill gas generated from 1 ton of different compositions of MSW (m^3 · ton MSW^{-1}) [a]		Paper	373.95
		Wood and bamboo	365.56
		Textile	420.70
		Organic components	204.41

The Parameters and inventory data for MSW landfill refer to [12]. a. Landfill gas generated from 1 ton of different compositions is calculated based on methane generation rate constant and degradable organic carbon, which refers to [12]. The feed-in tariff for power generated from landfill gas, with an efficiency of 0.33, is consistent with that for MSW incineration power generation. All landfill gas power generation revenue from MSW in the future is included in the landfill costs for the year in which it is landfilled.

Supplementary Table 5 Parameters and inventory data for MSW compost

Input			
PAC (kg · ton MSW^{-1})	0.75	PAM (kg · ton MSW^{-1})	0.08
NaClO (kg · ton MSW^{-1})	0.01	Electricity (kWh · ton MSW^{-1})	69.66
**Output**			
Fertilizer (ton · ton MSW^{-1})	0.19		

The Parameters and inventory data for MSW compost refer to [8].

Supplementary Table 6 Parameters and inventory data for MSW bioconversion

		**Bioconversion technologies** [a]			
		**B1**	**B2**	**B3**	**B4**
**Input**	Rice hull powder (ton · ton MSW^{-1})	0.25			
	Rice/wheat bran (kg · ton MSW^{-1})		50		
	Tap water (kg · ton MSW^{-1})		426	620	7.29
	PAC (kg · ton MSW^{-1})		1.64	2.1	2.01
	PAM (kg · ton MSW^{-1})		0.16	0.21	0.2
	NaClO (kg · ton MSW^{-1})		0.02	0.03	0.03
	NaOH (kg · ton MSW^{-1})				0.41
	Plant liquid (kg · ton MSW^{-1})			0.5	0.5

	Electricity (kWh · ton MSW^{-1})	25.19	18.25	71.82	32.21
	Natural gas (m^3 · ton MSW^{-1})			4.93	
**Output**	Fertilizer (ton · ton MSW^{-1})	0.13	0.07	0.20	0.13
	Protein [b] (ton · ton MSW^{-1})	0.03	0.01	0.03	0.02
	Biooil (ton · ton MSW^{-1})		0.02	0.02	0.02

The Parameters and inventory data for MSW bioconversion refer to [8]. a. The operational benefit parameters for bioconversion are based on the weighted average of 4 technologies in [8], with ratios of 25%, respectively. B1: bioconversion for black soldier fly (BSF); B2: bioconversion for BSF and biooil; B3: bioconversion for red head fly (RHF) and biooil; B4: heat hydrolysis and bioconversion. b. Protein represents black soldier flies in B1 and B2, and red head flies in B3 and B4, respectively.

Supplementary Table 7 Prices of products, energy, and materials involved in MSW incineration, landfill, bioconversion disposal, and recycling.

**Energy and water**					
Coal (CNY · ton^{-1})	762.5	Natural gas (CNY · (m^3)$^{-1}$)	3.54	Tap water (CNY · ton^{-1})	4.1
Electricity [a] (CNY · kWh^{-1})	0.66	Diesel [a] (CNY · kg^{-1})	8.53		
**Products [b] and materials involved in incineration**					
Lime (CNY · ton^{-1})	362	Activated carbon (CNY · ton^{-1})	11633	Bottom ash (CNY · ton^{-1})	65.37
**Products and materials involved in landfill**					
HDPE (CNY · ton^{-1})	8419				
**Products and materials involved in bioconversion disposal**					
Rice hull powder (CNY · ton^{-1})	285	Rice/wheat bran (CNY · ton^{-1})	2170	PAC (CNY · kg^{-1})	1.75
PAM (CNY · kg^{-1})	13.4	NaClO (CNY · kg^{-1})	0.3	NaOH (CNY · kg^{-1})	0.87
Plant liquid (CNY · kg^{-1})	9	Fertilizer (CNY · ton^{-1})	400 [8]	Biooil (CNY · ton^{-1})	4800 [8]
Black soldier (CNY · ton^{-1})	9000 [8]	Red head fly (CNY · ton^{-1})	3000 [8]		
**Recycled materials**					
Recycled paper	3129 [14]	Recycled glass	196 [14]	Recycled plastic	1077 [12]
Recycled textile	4500 [14]	Recycled metals	6209 [14]		

a. Electricity prices for general industrial and commercial uses and diesel prices across various provinces in China are adopted, and the average values are presented above. b. The subsidized electricity price for waste incineration in China is explained in Supplementary Text 3.

Supplementary Table 8 Statistics on the composition of MSW across cities in China

%	Max	Min	Mean	SD
**Organic component**	68.38	0.41	45.07	15.24
**Paper**	53.02	6.47	26.68	13.86
**Plastic**	17.22	0.22	13.54	1.59
**Textile**	5.09	1.37	2.10	0.69
**Glass**	50.53	1.68	2.00	3.64
**Metal**	4.17	0.64	0.71	0.43
**Ash content**	64.49	0.01	6.63	6.60
**Wood and bamboo**	22.07	0.00	2.89	2.97
**Other**	1.14	0.39	0.40	0.04

Supplementary Table 9 Parameters for the recycling of paper, plastics, glass, textiles and metals in MSW

	Paper	Plastic	Glass	Textile	Metal
**Recycling**					
**Substitution ratio (%)**	80 [15]	93.5 [14]	78.1 [16]	90.09 [17]	87.5 [14]
**Processing cost (CNY · ton^{-1})**	1964 [18]	600 [19]	97.5 [20]	1573 [21]	157.5 [20]
**Collection rate (%)**	51.2 [22]	30.65 [23]	43.62 [22]	21.94 [24]	65 [25]
**Sorting and transportation**					
**Sorting cost [a] (CNY · ton^{-1})**	436.51 [25]				
**Transportation cost (CNY · ton^{-1})**	66.94 [25]				

a. The sorting cost varies with the differences in the ratios of recyclable materials to MSW across different cities.

Supplementary Table 10 Evolution of China's MSW issues and policies since reform and opening up

Time Period	Stage	Waste Management Background & Issues	Policy Responses
1980-1990	Initial Response	With accelerating urbanization, municipal waste volume increased rapidly, but waste treatment facilities were severely inadequate [26], leading to waste siege and environmental pollution issues.	1. Started construction of landfills and incinerators to handle increasing waste volume. 2. Issued Municipal Waste Management Measures (1993), initially standardizing waste treatment processes.

1990-2000	Systematic Response	Continued increase in waste volume with more complex composition [27,28], increasing organic matter, combustibles, and recyclables while decreasing inorganic content; outdated collection and transportation methods.	1. Proposed principles of reduction, resource utilization, and harmless treatment, began advocating waste sorting and resource recycling.<br>2. Construction Ministry issued Municipal Waste Treatment and Pollution Prevention Technical Policy (2000), systematically outlining technical approach of sorting as prerequisite, combining landfill, composting, incineration, and recycling.
2000-2010	Comprehensive Management Exploration	Gradual progress in waste treatment facility construction, increasing harmless treatment rate [29], but waste sorting and resource recycling needed strengthening [30].	1. Enacted Circular Economy Promotion Law (2009), promoting resource recycling and waste reduction. 2. Implemented waste sorting system, began pilot programs (2010), promoting end-point reduction.
2010-2020	Management Approach Correction	Continued growth in urban waste generation, more mixed composition; sorting ineffective due to mixed disposal at source, mixed transportation, and mismatched terminal treatment [31].	1. Waste Sorting System Implementation Plan (2017) mandated sorting in urban areas of 46 cities, targeting 35% recycling by 2020.<br>2. Notice on Comprehensive Implementation of Waste Sorting in Prefecture-level Cities (2019), aiming for complete sorting systems in all prefecture-level cities and 4 municipalities by 2025.
2020-	Comprehensive Promotion & Reform Deepening	Gradual improvement of sorting and treatment systems, significant enhancement in resource utilization and harmless treatment capacity, but continued optimization needed for sustainable development.	1. 14th Five-Year Plan for Urban Waste Sorting and Treatment Facilities Development (2021) targets 60% resource utilization rate by 2025.<br>2. Carbon Peaking and Carbon Neutrality Goals (2021) emphasizes waste reduction and resource utilization as key components of circular economy carbon reduction.

Supplementary Table 11 Local waste sorting related policies in China

City	Time	Policy
Chongqing	2019.01	Chongqing Municipal Waste Sorting Management Measures
Shanghai	2019.07	Shanghai Municipal Waste Management Regulations
Hangzhou	2019.08	New Hangzhou Municipal Waste Management Regulations
Xi'an	2019.09	Xi'an Municipal Waste Sorting Management Measures
Guangzhou	2019.12	New Guangzhou Municipal Waste Sorting Management Measures
Zhengzhou	2019.12	Zhengzhou Urban Waste Sorting Management Measures
Shenyang	2019.12	Waste Sorting Signs

Harbin	2020.02	Harbin Urban Waste Sorting Management Measures
Beijing	2020.05	New Beijing Municipal Waste Management Regulations
Suzhou	2020.06	Suzhou Municipal Waste Sorting Management Regulations
Dongguan	2020.06	Dongguan Municipal Waste Sorting Three-Year Action Plan (2020-2022)
Qingdao	2020.06	Qingdao Municipal Waste Sorting Measures
Wuhan	2020.07	Wuhan Municipal Waste Sorting Management Measures
Shenzhen	2020.09	Shenzhen Municipal Waste Sorting Management Regulations
Changsha	2020.10	Changsha Municipal Waste Management Regulations
Hohhot	2020.10	Hohhot Municipal Waste Sorting Management Measures
Nanjing	2020.11	Nanjing Municipal Waste Management Regulations
Tianjin	2020.12	Tianjin Municipal Waste Management Regulations
Hefei	2020.12	Hefei Municipal Waste Sorting Management Regulations
Chengdu	2021.03	Chengdu Municipal Waste Sorting Implementation Plan
Jinan	2021.05	Jinan Municipal Waste Reduction and Sorting Management Regulations

Supplementary Table 12 Waste incineration planning and completion since the 12th five-year plan

Year	Policy	Main content related to waste incineration	Planned capacity proportion	Completion status
2012	12th Five-Year Plan for National Urban Waste Harmless Treatment Facilities Construction	1. Regions with suitable conditions should prioritize incineration and other resource utilization treatment technologies.<br>2. By 2015, national urban waste incineration treatment facility capacity should reach over 35% of total harmless treatment capacity, with eastern regions reaching over 48%.	Urban 35%	2015: Urban 28%
2016	13th Five-Year Plan for National Urban Waste Harmless Treatment Facilities Construction	1. By end of 2020, municipal waste incineration treatment capacity should account for over 50% of total harmless treatment capacity, with eastern regions reaching over 60%.<br>2. By end of 2020, qualified municipalities, specially designated cities, and provincial capitals (built-up areas) should achieve zero landfill for raw waste.	Urban 50%	2020: Urban 59%

2021	14th Five-Year Plan for Urban Waste Classification and Treatment Facilities Development	1. By end of 2025, national urban waste incineration treatment capacity should reach about 800,000 tons/day, with urban waste incineration treatment capacity proportion around 65%.<br>2. In principle, cities at prefecture level and above, and counties with incineration treatment capacity or construction conditions, should no longer plan and build new raw waste landfill facilities; existing landfill capacity should be converted to backup facilities.	Urban 65%	2022: Urban 73%

Supplementary Table 13 Key policies in household food waste treatment industry

Policy	Time	Content	Significance
Notice on Organizing Urban Household Food Waste Resource Utilization and Harmless Treatment Pilot Work	2010.05	Proposed selecting first batch of pilot cities for Household Food Waste resource utilization and harmless treatment nationwide, gradually establishing suitable regulations, policies, and technical routes to improve resource utilization levels.	Initiated China's Household Food Waste treatment market
Resource Comprehensive Utilization Products and Services VAT Preferential Catalog	2015.06	Implemented 100% VAT refund policy for resource products: biogas fuel blocks, biogas, electricity, heat produced from Household Food Waste.	Tax incentives to increase terminal disposal enterprises' initiative
Strategic Emerging Industries Key Products and Services Guidance Catalog	2017.01	Listed Household Food Waste resource utilization and harmless treatment related equipment and technology R&D as part of resource recycling industry	Future Household Food Waste treatment technology development will be resource-oriented
Shanghai Household Food Waste Oil Treatment Management Measures Implementation Provisions	2022.06	Specified requirements for waste oil treatment units and collection units regarding bidding, collection containers, personnel training, supervision, etc.	Multiple provinces and cities issued documents promoting Household Food Waste collection and resource utilization
Beijing 14th Five-Year Urban Management Development Plan	2022.04	Promoted exploration of Household Food Waste and sewage co-treatment, actively promoted biogas resource utilization from Household Food Waste treatment facilities, explored comprehensive utilization of fly ash and incineration slag.	

Guangzhou Household Food Waste Management Measures	2021.10	Regulated Household Food Waste discharge, collection, disposal, with three-year policy validity
Chongqing Municipal Waste Management Regulations	2021.11	Encouraged Household Food Waste generators to use new technology and equipment for oil-water separation, promoting source reduction

477

References

1. Teng FY, Wang ZY, Ren K, et al. Analysis of composition characteristics and treatment techniques of municipal solid waste incineration fly ash in China[J]. Journal of Environmental Management, 2024, 357: 120783.
2. Chinese Ministry of Ecology and Environment (CMEE). (2023). Automatic monitoring data public platform for household waste incineration power plants. https://ljgk.envsc.cn.
3. Chinese National Development and Reform Commission (CNDRC). (2012). Notice on improving the price policy for waste incineration power generation. https://www.gov.cn/zwgk/2012-04/10/content_2109921.htm.
4. Zhang M, Wei JX, Li H, et al. Comparing and optimizing municipal solid waste (MSW) management focused on air pollution reduction from MSW incineration in China[J]. Science of The Total Environment, 2024, 907: 167952.
5. Zhou ZQ, Zhang L. Sustainable waste management and waste to energy: Valuation of energy potential of MSW in the Greater Bay Area of China[J]. Energy Policy, 2022, 163: 112857.
6. Xue YS, Moreno JM, Li CJ, et al. Growing community-based composting programs in China: Implementation and policy lessons from eight cases[J]. Resources, Conservation and Recycling, 2025, 212: 107882.
7. Bohm K, Hatley GA, Robinson BH, et al. Black Soldier Fly-based bioconversion of biosolids creates high-value products with low heavy metal concentrations[J]. Resources, Conservation and Recycling, 2022, 180: 106149.
8. Liu, F., Xin, L., Tang, H. et al. Regionalized life-cycle monetization can support the transition to sustainable rural food waste management in China. Nat Food 4, 797–809 (2023).
9. People's Government of China. (2024). 15 cities will create demonstration models for waste sorting [EB/OL]. Available at: https://www.beijing.gov.cn/ywdt/zybwdt/202405/t20240527_3694298.html [in Chinese]
10. Ministry of Housing and Urban-Rural Development of the People's Republic of China. (2012). Technical specification for kitchen waste treatment: CJJ 184-2012. Beijing: China Architecture & Building Press. [in Chinese]
11. Yang, N., Shao, L. M., & He, P. J. (2018). Analysis of moisture content and characteristics of municipal solid waste components in China. China Environmental Science, (3), 1033-1038. [in Chinese]
12. Fang W, Ding YM, Geng JH, et al. High potential of coupling the source-separation and incineration promotion to reduce costs based on city-level cost-benefit analysis of municipal solid waste management strategies in China[J]. Resources, Conservation and Recycling, 2023, 197: 107099.
13. Octavianthy, D., Syauqi, A., Reyseliani, N. et al. Multi-period Enviro-Economic Optimization of Municipal Solid Waste to Electricity. Waste Biomass Valor 13, 3707–3722 (2022).
14. Zaman AU. A comprehensive study of the environmental and economic benefits of resource recovery from global waste management systems[J]. Journal of

Cleaner Production, 2016, 124: 41-50.
15. Liu MZ, Tan S, Zhang MY, et al. Waste paper recycling decision system based on material flow analysis and life cycle assessment: A case study of waste paper recycling from China[J]. Journal of Environmental Management, 2020, 255: 109859.
16. Yuan XH, Wang JB, Song QM, et al. Integrated assessment of economic benefits and environmental impact in waste glass closed-loop recycling for promoting glass circularity[J]. Journal of Cleaner Production, 2024, 444: 141155.
17. Mu BN, Yu XQ, Shao YY, et al. Complete recycling of polymers and dyes from polyester/cotton blended textiles via cost-effective and destruction-minimized dissolution, swelling, precipitation, and separation[J]. Resources, Conservation and Recycling, 2023, 199: 107275.
18. Li JG, Mei MY, Han YL, et al. Life cycle cost assessment of recycled paper manufacture in China[J]. Journal of Cleaner Production, 2020, 252: 119868.
19. Faraca G, Sanchez VM, Astrup TF. Environmental life cycle cost assessment: Recycling of hard plastic waste collected at Danish recycling centres[J]. Resources, Conservation and Recycling, 2019, 143: 299-309.
20. Albizzati PF, Foster G, Gaudillat P, et al. A model to assess the environmental and economic impacts of municipal waste management in Europe[J]. Waste Management, 2024, 174: 605-617.
21. Mu BN, Yu XQ, Shao YY, et al. Complete recycling of polymers and dyes from polyester/cotton blended textiles via cost-effective and destruction-minimized dissolution, swelling, precipitation, and separation[J]. Resources, Conservation and Recycling, 2023, 199: 107275.
22. China National Resources Recycling Association. (2024). Development report on renewable resource recycling industry in China. http://www.crra.com.cn/.
23. National Development and Reform Commission. (2024). Comprehensive research report on chemical cycle of waste plastics. http://download.china.cn/ch/综合性研究报告中文版 20240419.pdf
24. National Development and Reform Commission. (2023). Research report on the current status of low-value recyclable materials recycling and utilization in http://download.china.cn/idc/中国低值可回收物回收利用现状调查报告.pdf
25. Li GH, Wang WJ, You XY. Social-economic assessment of integrated waste pickers in municipal solid waste management system: A case of Tianjin in China[J]. Journal of Cleaner Production, 2024, 434: 140302.
26. Yi, J. T. (1996). Discussion on urban domestic waste issues. Modern Urban Research, (04), 62-64. [in Chinese]
27. Wang, W. P. (2000). Research on countermeasures of urban domestic waste in China. Journal of Natural Resources, (02), 128-132. [in Chinese]
28. Du, W. P., Gao, Q. X., Zhang, E. C., et al. (2006). Analysis of municipal solid waste disposal and trends in China. Research of Environmental Sciences, (06), 115-120. [in Chinese]
29. Wang, Y. N. (2010). Research on current situation and development of municipal solid waste incineration power generation in China's large cities. Macroeconomics,

(11), 12-23. [in Chinese]

30. Wu, Y. C., & Xu, L. F. (2013). Analysis of the barrier factors of municipal solid waste classification recycling. Advanced Materials Research, 726-731, 2618-2621.
31. Peng, Y., Li, L., Peng, X. Y., et al. (2018). Development process, obstacles and countermeasures of domestic waste classification in China. China Environmental Science, 38(10), 3874-3879. [in Chinese]

Supplementary File 2

Historical MSW disposal structure in 20 typical cities. The internal pie chart represents the proportion of harmless disposal quantity to the total MSW delivering quantity, while the external ring chart indicates the proportions of various disposal technologies. Additionally, the size of the rings directly proportional to the MSW delivering quantities of typical cities in the same historical year.

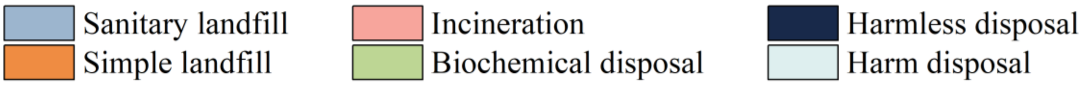

Shanghai-2000:

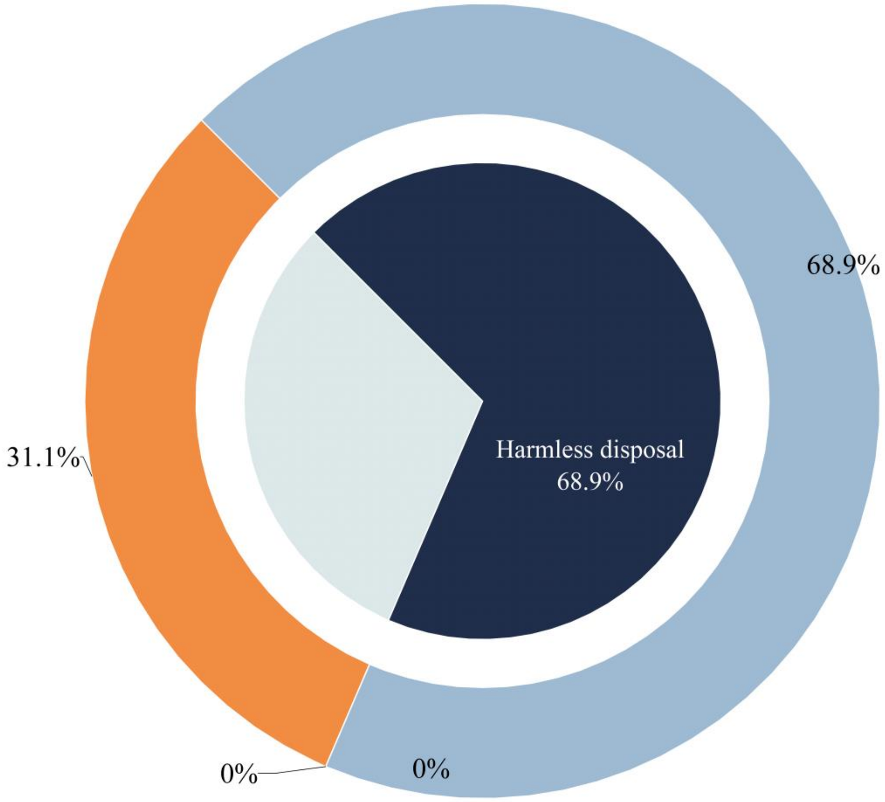

Beijing-2000:

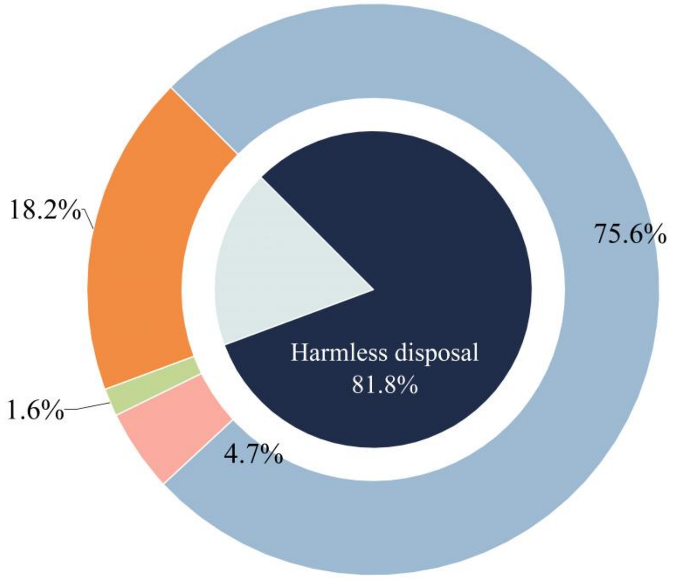

Nanchang-2000:

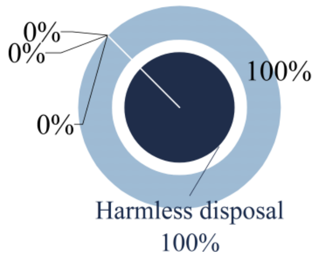

Hefei-2000:

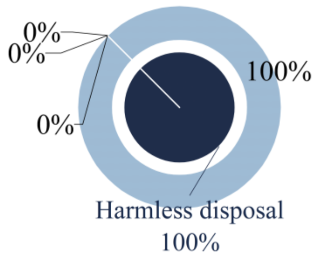

Hohhot-2000:

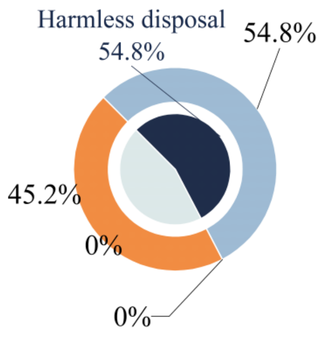

Shangqiu-2000:

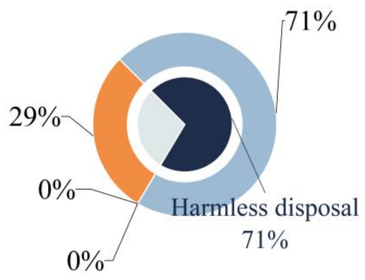

Jiangxing-2000:

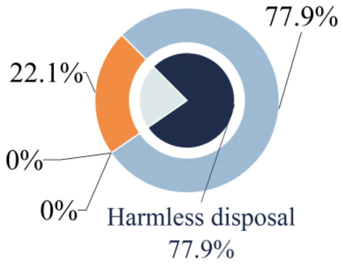

Daqing-2000:

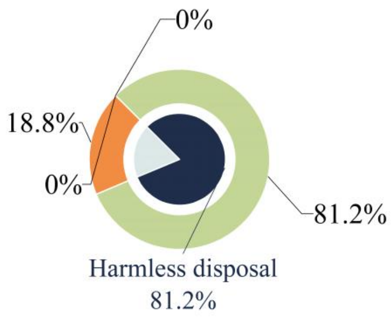

Tianjing-2000:

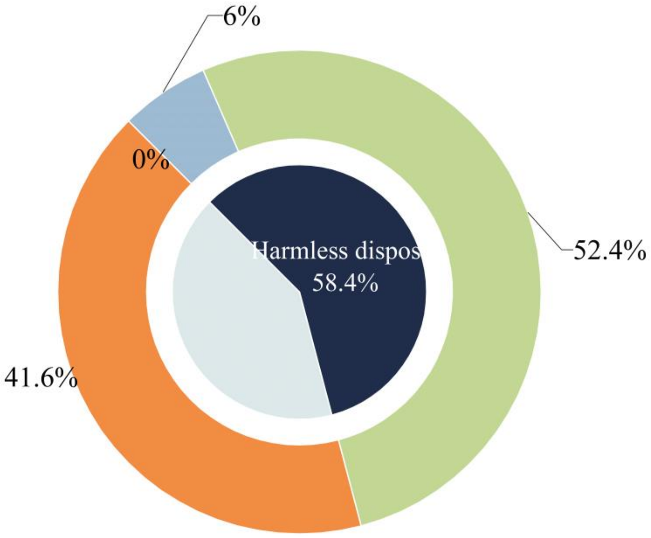

Guangan-2000:

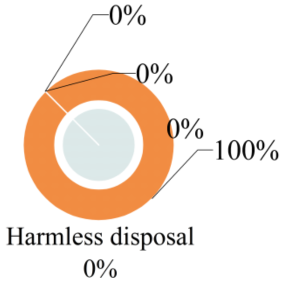

Guangzhou-2000:

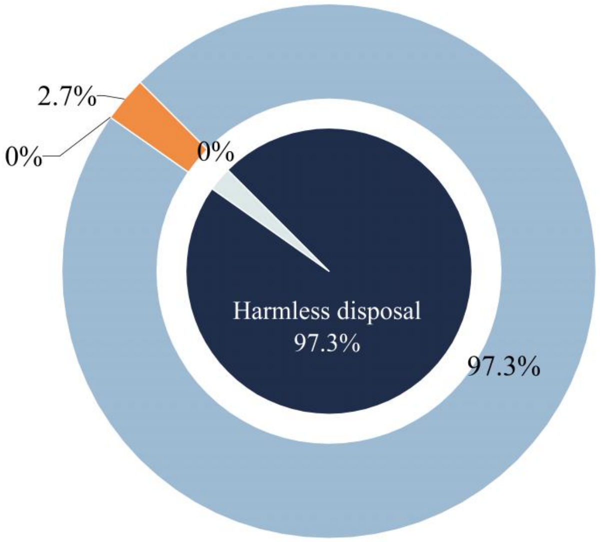

Kunming-2000:

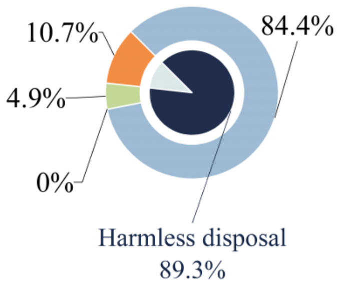

Wuhan-2000:

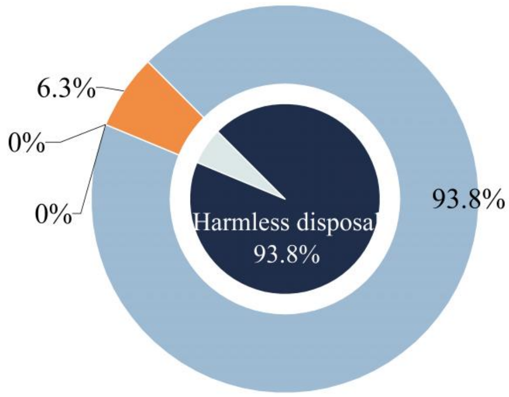

Shenzhen-2000:

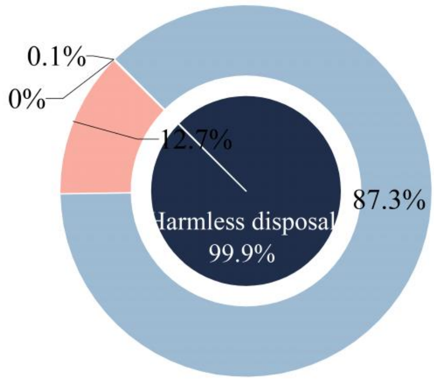

Baiyin-2000:

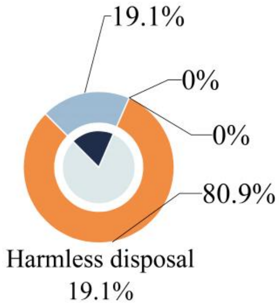

Xian-2000:

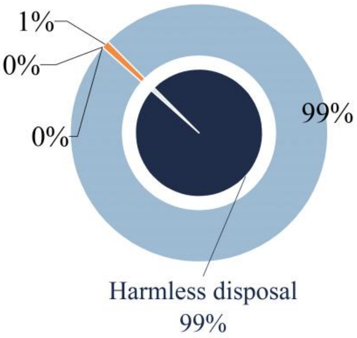

Chongqing-2000:

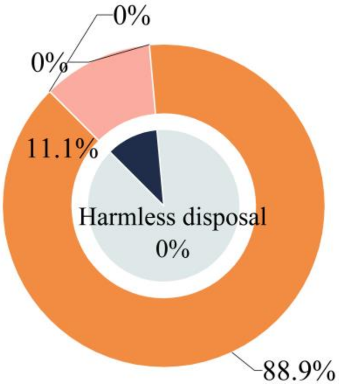

Changsha-2000:

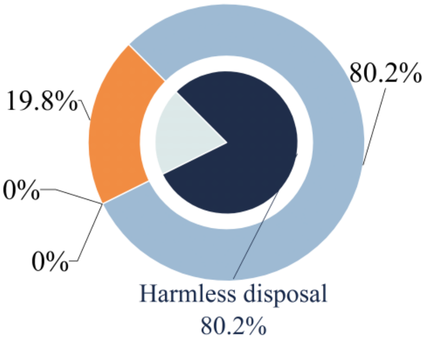

Aksu prefecture -2000:

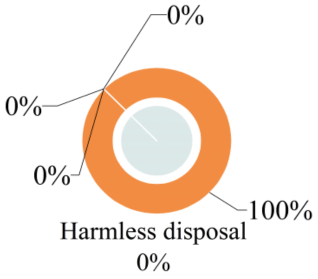
0%
0%
0%
100%
Harmless disposal
0%

Anshan-2000:

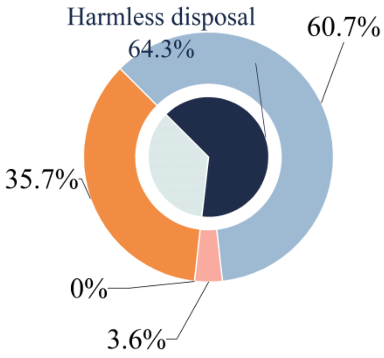
Harmless disposal
64.3%
60.7%
35.7%
0%
3.6%

Shanghai-2010:

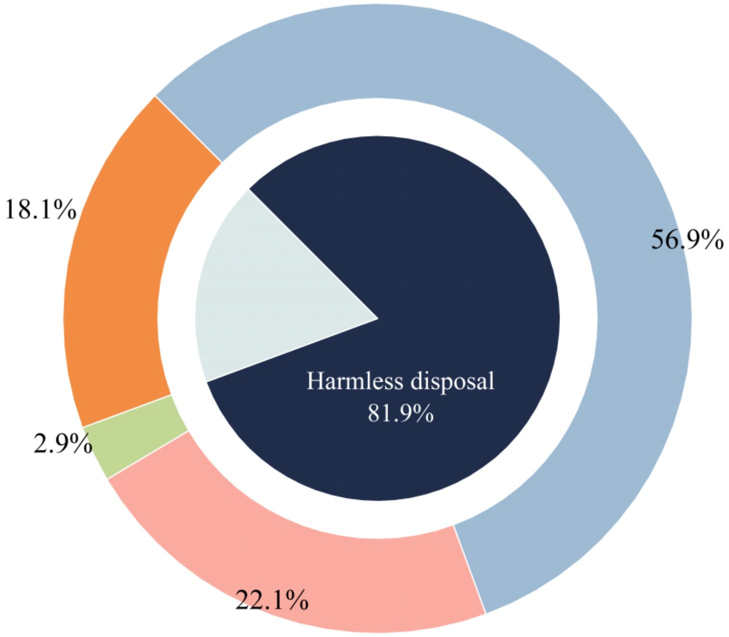

Beijing-2010:

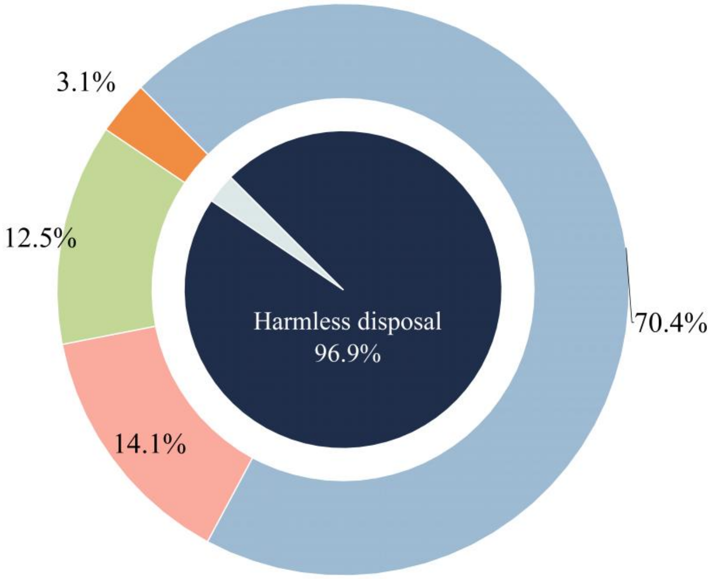

Nanchang-2010:

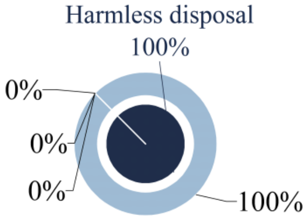

Hefei-2010:

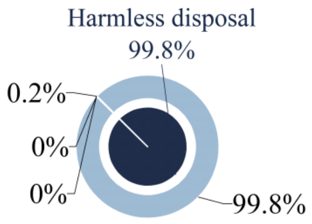

Hohhot-2010:

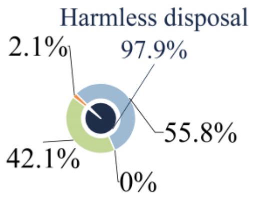

Shangqiu-2010:

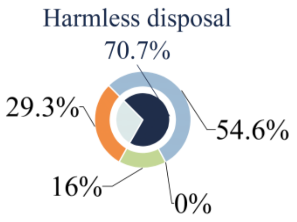

Jiaxing-2010:

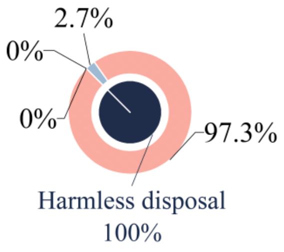

Daqing-2010:

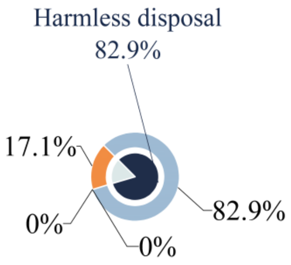

Tianjin-2010:

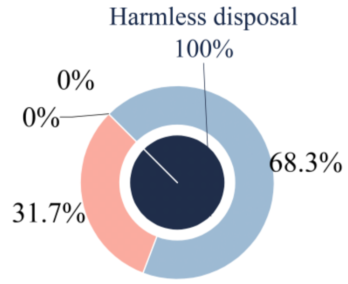

Guangan-2010:

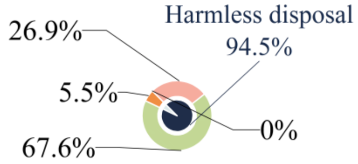

Guangzhou-2010:

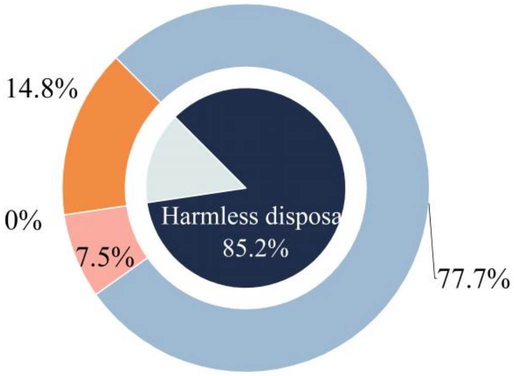

Kunming-2010:

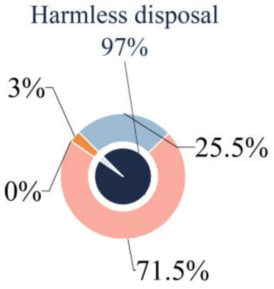

Wuhan-2010:

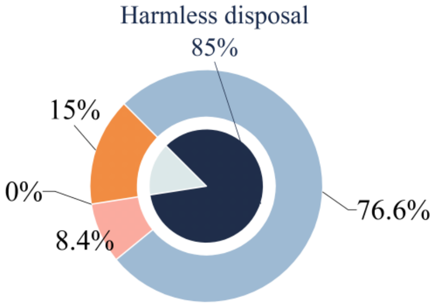

Shenzhen-2010:

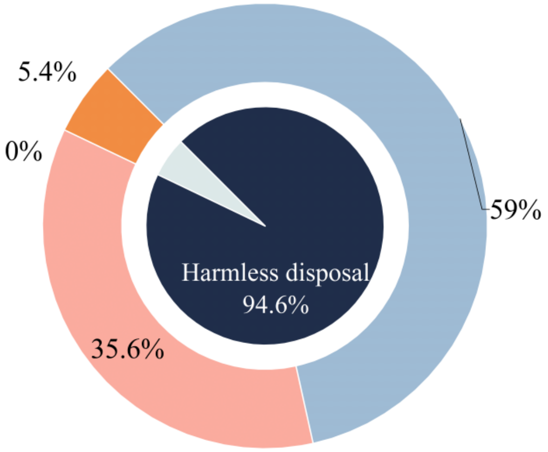

Baiyin-2010:

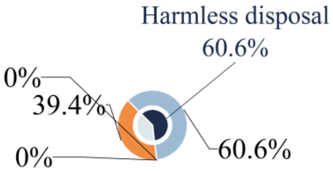

Xian-2010:

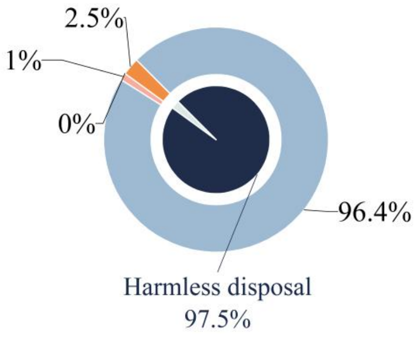

Chongqing-2010:

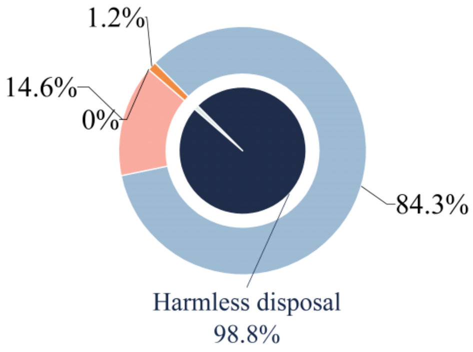

Changsha-2010:

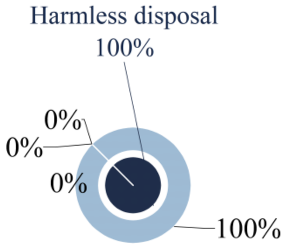

Aksu prefecture-2010:

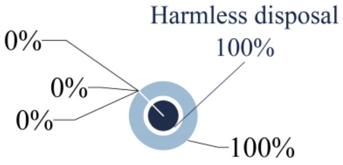

Anshan-2010:

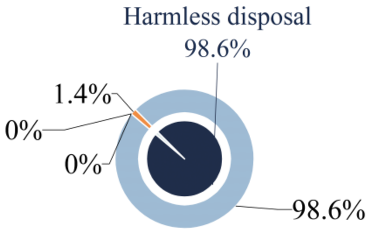

Shanghai-2020:

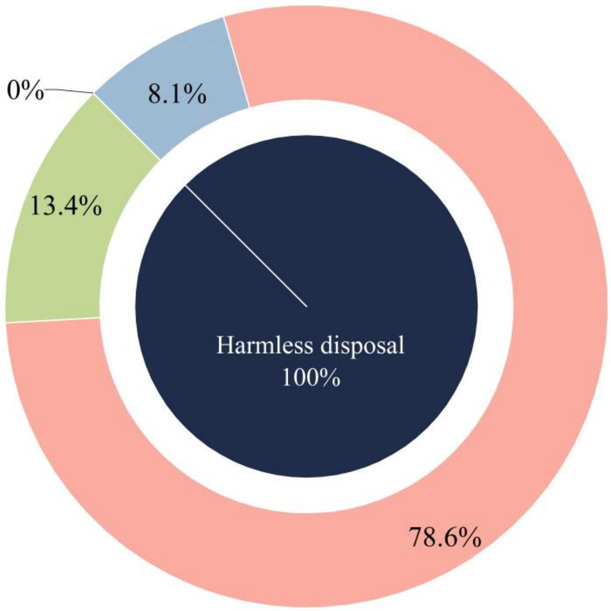

Beijing-2020:

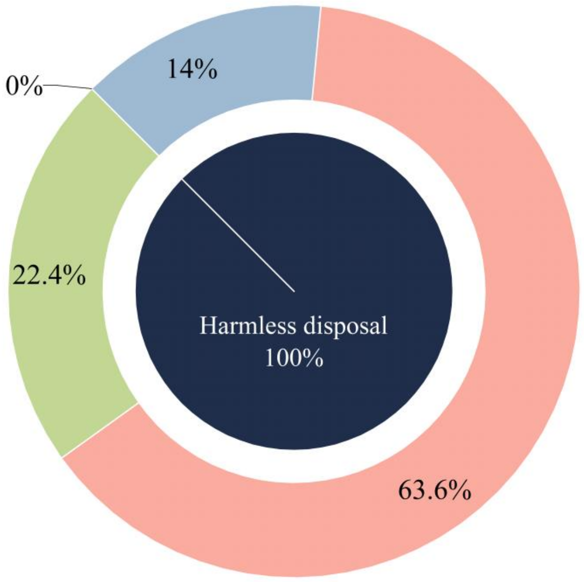

Nanchang-2020:

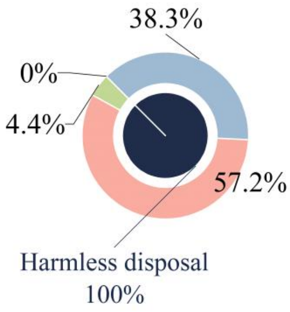

Hefei-2020:

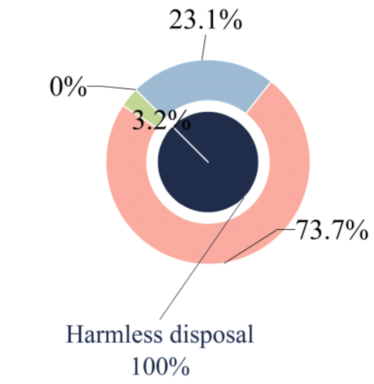

Hohhot-2020:

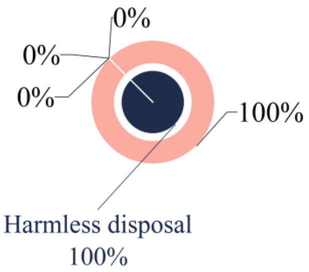

Shangqiu-2020:

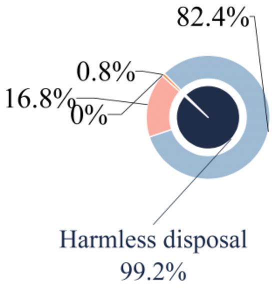

Jiaxing-2020:

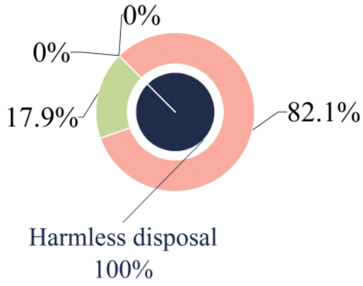

Daqing-2020:

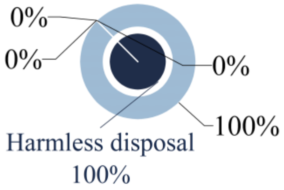

Tianjin-2020:

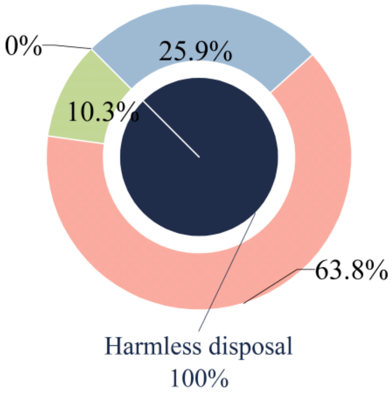

Guangan-2020:

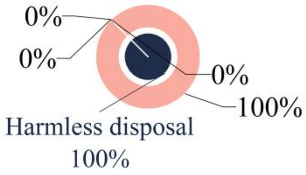

Guangzhou-2020:

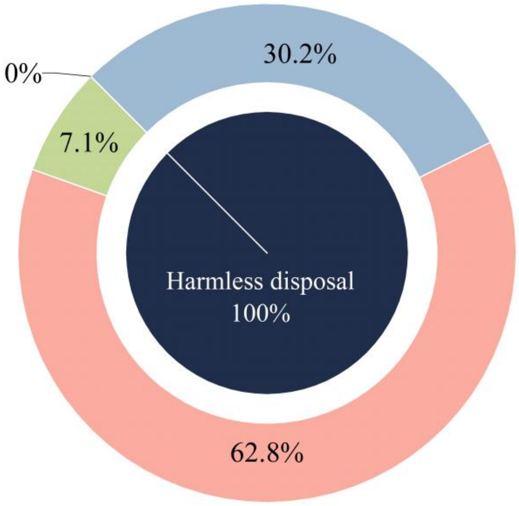

Kunming-2020:

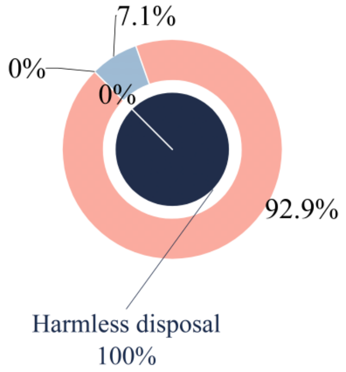

Wuhan-2020:

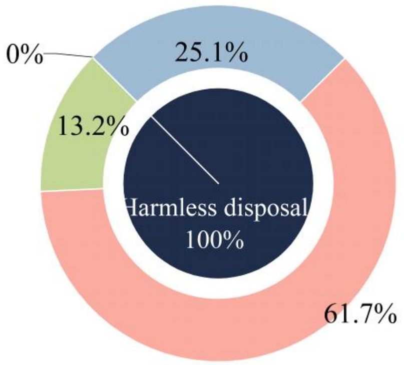

Shenzhen-2020:

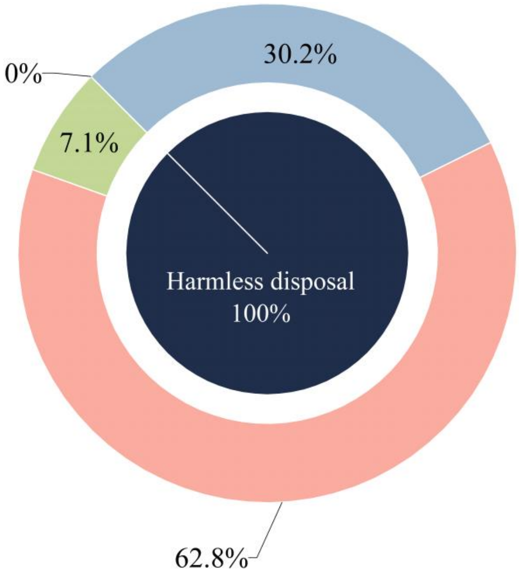

Baiyin-2020:

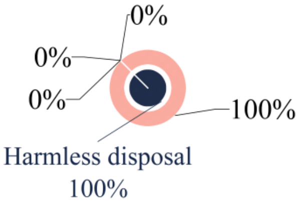

Xian-2020:

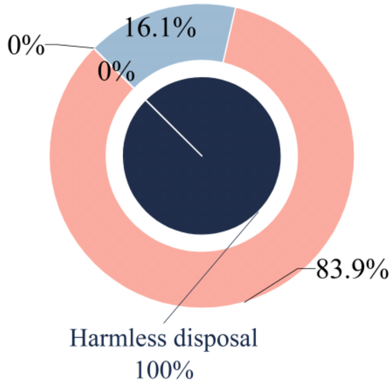

Chongqing-2020:

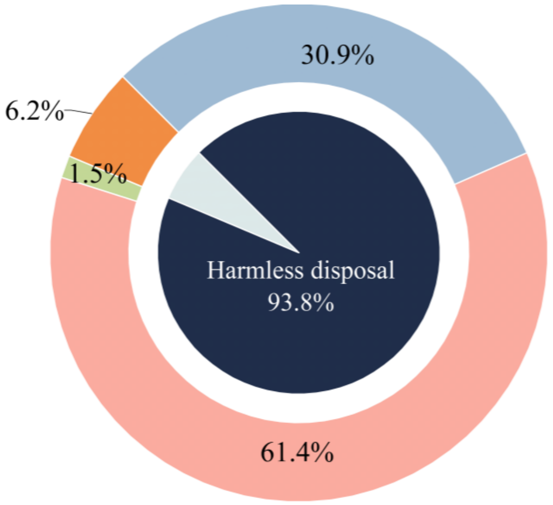

Changsha-2020

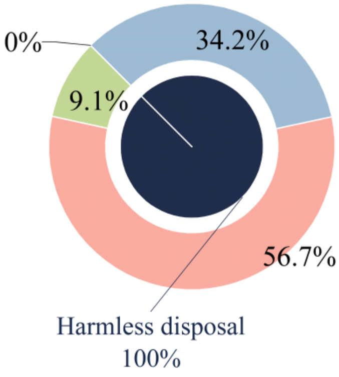

Aksu prefecture-2020:

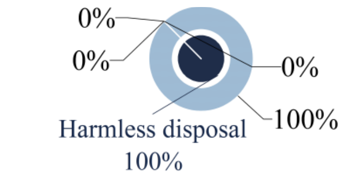

Anshan-2020:

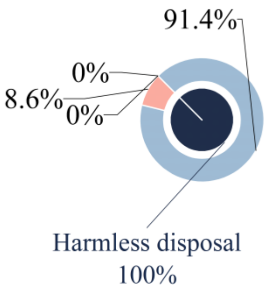

Supplement: Document S1. Figures S1–S11, Tables S1–S13, and Files S1 and S2 [file mmc1.pdf]
