# Supplementary material for: The waste-environmental-economic nexus facilitates city-specific cost-effective transition for China’s municipal solid waste treatment
Source: Innovation (Camb). 2026 Feb 12;7(7):101315. doi: 10.1016/j.xinn.2026.101315 (PMC13343423; doi:10.1016/j.xinn.2026.101315)

ARTICLE | Environment & Ecology

OPEN ACCESS

The waste-environmental-economic nexus facilitates city-specific cost-effective transition for China's municipal solid waste treatment

Hao Li,[1,2,3] Xiaolong Lu,[1] Fang Liu,[4,5,6,*] Zhe Li,[4] Xianmei Liu,[7] Bin Lu,[1,2,3] Shijun Ma,[8,*] Baojing Gu,[9,*] Chuanbin Zhou,[10] and Zhaohua Wang[1,2,3,*]

*Correspondence: liufang@zafu.edu.cn (F.L.); ucbvsms@ucl.ac.uk (S.M.); bjgu@zju.edu.cn (B.G.); wangzhaohua@bit.edu.cn (Z.W.)

Received: April 20, 2025; Accepted: February 10, 2026; Published Online: February 12, 2026; https://doi.org/10.1016/j.xinn.2026.101315

© 2026 The Author(s). Published by Elsevier Inc. on behalf of Youth Innovation Co., Ltd. This is an open access article under the CC BY license (http://creativecommons.org/licenses/by/4.0/).

GRAPHICAL ABSTRACT

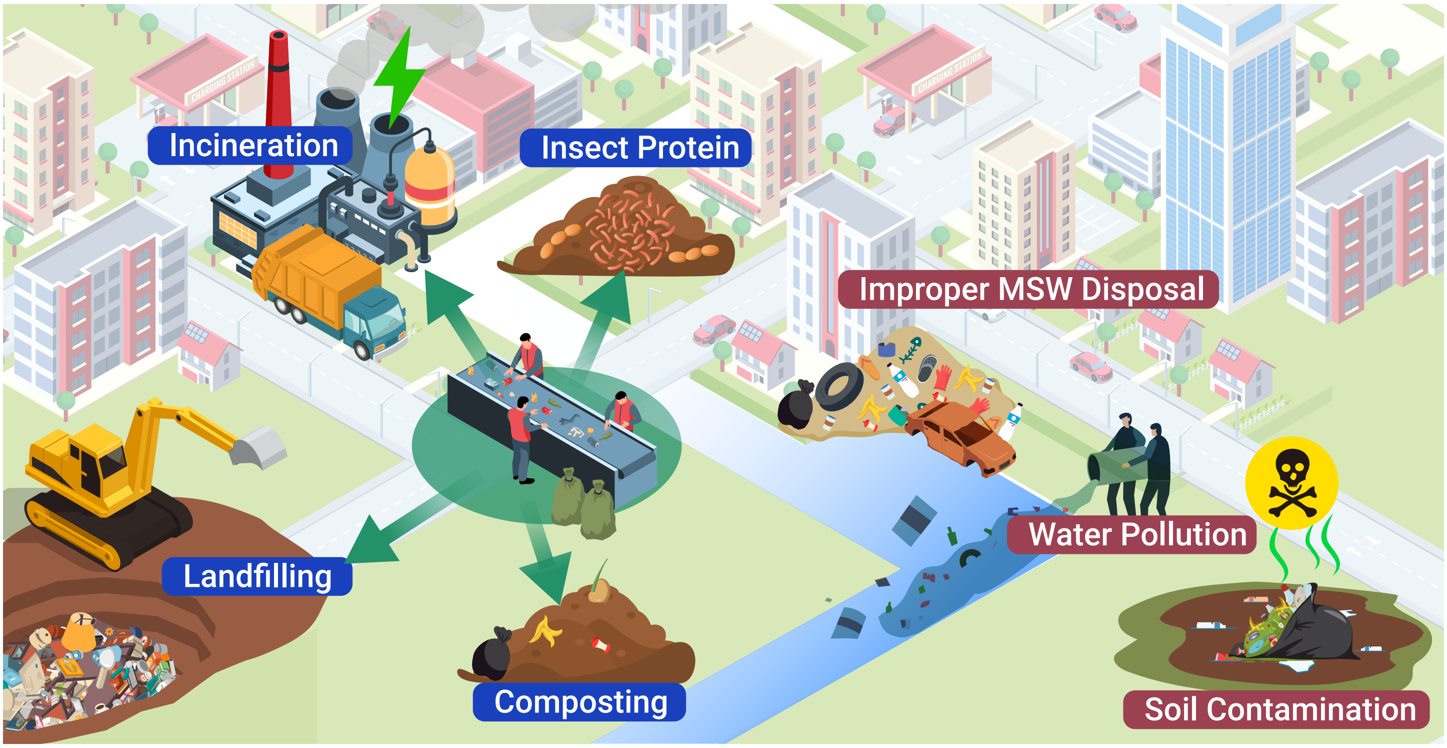

PUBLIC SUMMARY

- A waste-environmental-economic nexus framework is developed to identify optimal strategies.
- Adopting waste-to-material strategies could reduce total environmental costs by 36.9%–78.3%.
- Significant trade-offs exist between economic and environmental outcomes across regions.
- Integrated WtE and WtM strategies are expected to yield superior outcomes for >84% of cities.

The waste-environmental-economic nexus facilitates city-specific cost-effective transition for China's municipal solid waste treatment

Hao Li,[1,2,3] Xiaolong Lu,[1] Fang Liu,[4,5,6,*] Zhe Li,[4] Xianmei Liu,[7] Bin Lu,[1,2,3] Shijun Ma,[8,*] Baojing Gu,[9,*] Chuanbin Zhou,[10] and Zhaohua Wang[1,2,3,*]
[1]School of Economics, Beijing Institute of Technology, Beijing 100081, China
[2]Digital Economy and Policy Intelligentization Key Laboratory of Ministry of Industry and Information Technology, Beijing 100081, China
[3]Center for Sustainable Development and Smart Decision, Beijing Institute of Technology, Beijing 100081, China
[4]College of Economics and Management, College of Environment and Resources, College of Carbon Neutral Zhejiang A&F University, Hangzhou 311300, China
[5]Zhejiang Province Key Think Tank: Institute of Ecological Civilization and Institute of Carbon Neutrality, Zhejiang A&F University, Hangzhou 311300, China
[6]Zhejiang Key Laboratory of Ecological Environmental Damage Control and Value Transformation Zhejiang A&F University, Zhejiang 311300, China
[7]School of Economics, Hebei University of Economics and Business, Shijiazhuang 050062, P.R. China
[8]Bartlett School of Sustainable Construction, University College London, London WC1H 9BT, UK
[9]College of Environmental and Resource Sciences, Zhejiang University, Hangzhou 310058, China
[10]State Key Laboratory of Regional and Urban Ecology, Research Center for Eco-Environmental Sciences, Chinese Academy of Sciences, Beijing 100083, China
*Correspondence: liufang@zafu.edu.cn (F.L.); ucbvsms@ucl.ac.uk (S.M.); bjgu@zju.edu.cn (B.G.); wangzhaohua@bit.edu.cn (Z.W.)
Received: April 20, 2025; Accepted: February 10, 2026; Published Online: February 12, 2026; https://doi.org/10.1016/j.xinn.2026.101315
© 2026 The Author(s). Published by Elsevier Inc. on behalf of Youth Innovation Co., Ltd. This is an open access article under the CC BY license (http://creativecommons.org/licenses/by/4.0/).
Citation: Li H., Lu X., Liu F., et al., (2026). The waste-environmental-economic nexus facilitates city-specific cost-effective transition for China's municipal solid waste treatment. The Innovation **7(7)**, 101315.

The growing challenge of municipal solid waste (MSW) generation requires systematic solutions based on the waste-environment-economy (WEE) nexus. Here, we develop an integrated assessment framework that combines bottom-up economic costing, life cycle monetization, and scenario analysis to evaluate nine MSW treatment strategies in 352 Chinese cities. Our approach integrates 18 environmental impact categories with key economic performance indicators. The results demonstrate that waste-to-material strategies could reduce China's total environmental costs of MSW treatment by 36.9%–78.3%, primarily driven by substantial reductions in greenhouse gas emissions and freshwater ecotoxicity. However, the WEE nexus highlights notable regional trade-offs between economic and environmental outcomes, largely due to variations in waste composition and local infrastructure. By using a composite benefit index to identify optimal MSW management pathways, we find that integrated strategies, combining incineration with material recycling (either alone or combined with bioconversion), prove cost-effective in over 84% of the studied cities. In contrast, a bioconversion-dominated strategy is most effective for the remaining cities. These findings provide spatially targeted guidance for facilitating city-specific transitions toward feasible MSW management in China.

INTRODUCTION

The growing generation of municipal solid waste (MSW) is a significant global challenge, driven by population growth, rapid urbanization, and changing consumption patterns. Poor waste management threatens the environment, public health, and climate stability.[1,2] China, the world's largest MSW producer, contributes over 10% of global waste and faces an acute crisis. Projections indicate a potential doubling of its waste volume by 2060, intensifying pressures on already strained management systems.[3] Despite significant policy efforts, many Chinese cities still struggle with waste overflow, inadequate treatment capacity, and environmental damage.[4,5] Addressing these challenges requires a shift toward integrated, spatially tailored strategies that ensure safe disposal and improve resource recovery. Within this context, we propose a waste-environmental-economic (WEE) nexus framework to guide city-specific, cost-effective transitions of MSW treatment. Effective MSW management is essential not just for waste disposal but also for alleviating environmental pressures and supporting climate mitigation. In response, China launched a national source-separation initiative in 2019, aiming to diversify its treatment system by 2025. Key targets include treating 65% of MSW via incineration and achieving 60% resource utilization efficiency.[6] Waste-to-energy (WtE) technologies, especially incineration, are widely used for their ability to reduce waste volume, conserve land, and generate energy.[7,8] However, WtE's long-term sustainability is uncertain due to its reliance on subsidies, uneven distribution of facilities, and low energy recovery efficiency.[9,10] These limitations underscore the need to critically evaluate alternative pathways and their inherent trade-offs.

Extensive academic research on MSW treatment technologies has evolved from detailed technical analyses to broader system simulations, and from single-dimensional to multidimensional assessments. Early studies predominantly focused on the environmental, resource, or economic performance of individual treatment technologies.[11,12] For instance, numerous life cycle assessment studies have precisely quantified greenhouse gas (GHG) emissions and resource consumption associated with technologies such as incineration, landfilling, or anaerobic digestion, establishing a methodological foundation for uncovering the micro-level impacts of technological units.[13–15] However, waste management is a complex system involving classification, collection, transportation, treatment, and disposal. Improving one component does not guarantee overall system improvement. Consequently, research has shifted to macro and systemic approaches, using methods such as material flow analysis, techno-economic assessment, and integrated models to optimize long-term waste management strategies at regional or national levels.[16,17] While these macro-scale models facilitate the evaluation of synergies between technology portfolios and emission reduction targets, they often sacrifice spatial resolution for model operability. Their conclusions are generally applicable at national or large regional scales, failing to capture the substantial heterogeneity among cities, thereby limiting their usefulness for local governance.[18,19]

In terms of assessment dimensions, research has evolved from isolated environmental or economic analyses to integrated frameworks that address multiple dimensions. Monetizing environmental externalities such as carbon emissions and pollution, and integrating them with economic costs, is a crucial step in supporting decision-making.[20–23] This approach enables comprehensive net-benefit comparisons across different strategies through a unified monetary metric.[24,25] However, significant gaps persist in existing integrated studies. On one hand, most monetary assessments at national or regional levels rely on globally averaged parameters, failing to reflect the considerable variations among Chinese cities in waste composition, treatment costs, environmental carrying capacity, and other localized factors.[26] On the other hand, while some city-level studies include economic considerations, their environmental cost estimates often rely on simplified assumptions or data from broader administrative scales. These studies fail to fully integrate and spatially represent environmental externalities alongside localized economic costs.[27,28] This disconnect makes current assessment frameworks inadequate for identifying optimal treatment pathways specific to each city, limiting their usefulness for formulating context-specific policies, such as those needed for zero-waste cities.

We applied the WEE nexus framework to analyze cost-effective solutions for the MSW treatment transition in 352 Chinese cities, by effectively reconciling the trade-offs between environmental costs and economic performance. Our analysis builds on a bottom-up compilation of historical MSW-related data spanning the past two decades with projections through 2050, covering waste generation, composition, and treatment infrastructure. By integrating facility-level economic data of 1,954 treatment plants with life cycle costing and monetized environmental impacts, we evaluate scenario-specific outcomes from 2020 to 2050. The application of this framework demonstrates that a hybrid pathway integrating incineration with material recycling, in some cases supplemented by bioconversion, is optimal for over 84% of cities, providing a clear, evidence-based rationale for municipal policy transitions.

Table 1. Overview of MSW treatment scenarios

Scenarios	Scenario narrative	Incineration rate[a]	Bioconversion	Bioconversion disposal rate	Landfill rate	Materials recycling[b]
BAU	ratio of each MSW disposal structure is still same as that in 2021	same as 2021	composting	same as 2021	same as 2021	–
ID	incineration rate gradually increases, and nearly all MSW is combusted by 2050	significantly increasing	composting	significantly decreasing	significantly decreasing	–
LBC	a low proportion of food waste is treated with composting, and the remaining MSW is managed under the BAU scenario	decreasing	composting	low (30% and 50%)	decreasing	–
LBB	a low proportion of food waste is treated using bioconversion technology for protein recovery, and the remaining MSW is managed under the BAU scenario	decreasing	protein recovery	low (30% and 50%)	decreasing	–
HBC	a high proportion of food waste is treated with composting, and the remaining MSW is managed under the BAU scenario	decreasing	composting	high (50% and 100%)	decreasing	–
HBB	a high proportion of food waste is treated using bioconversion technology for protein recovery, and the remaining MSW is disposed according to BAU scenario	decreasing	protein recovery	high (50% and 100%)	decreasing	–
HBB-ID	a high proportion of food waste is treated for protein recovery, and the remaining MSW is managed under the ID scenario	–	protein recovery	high (50% and 100%)	significantly decreasing	–
RR-ID	recyclable materials undergo resource recovery, while the remaining MSW is managed under the ID scenario	significantly increasing	composting	gradually decreasing	significantly decreasing	✓
RR-HBB-ID	recyclable materials are recycled, high proportions of food waste are treated for protein recovery, and the remaining waste is managed under the ID scenario	–	protein recovery	high (50% and 100%)	significantly decreasing	✓

The difference among scenarios is based on the combination of the three modes (ID, LB, and HB) and the strategy of recyclable materials recycling, as well as the employment of biochemical disposal method, leading to distinct MSW treatment structures in each city from 2022 to 2050.
[a]In scenarios HBB-ID and RR-HBB-ID, the rate of incineration depends on the specific situation of each city considering the increase of bioconversion for food waste.
[b]Recyclable materials in MSW, including paper, plastics, glass, textiles, and metals, will be recycled based on a fixed collection rate before incineration and landfill. Their recycling rates are 22% for textiles, 44% for glass, 51% for paper, 31% for plastics, and 65% for metals. The classification for cities as less-developed or developed is based on whether the rate of biochemical disposal for organic components was lower or higher than 1% in 2021.

This study advances the existing literature in three key respects. Methodologically, the WEE framework integrates environmental external costs with detailed economic costs at the urban scale, providing a practical tool for multidimensional trade-off analysis in waste management. Analytically, the high-resolution simulation of 352 cities not only confirms the general effectiveness of hybrid pathways but, more critically, reveals that the optimal configuration of such pathways is highly contingent on local conditions. This highlights the limitation of a one-size-fits-all policy and lays a solid foundation for developing spatially differentiated strategies. In terms of decision-support, the dynamic assessment system constructed in this study translates integrated evaluation results into tailored policy intervention portfolios, such as tiered environmental tax or subsidy schemes, bridging the gap between academic analysis and practical management strategies. Together, these contributions create a scalable, transferable assessment-decision framework. This framework provides a scientific approach to aligning immediate waste management needs with long-term goals of a circular economy and carbon neutrality, applicable in both China and other rapidly urbanizing regions worldwide.

MATERIALS AND METHODS

Database construction of China's MSW treatment

MSW treatment plants dataset. We compiled a dataset of 1,954 MSW treatment plants operating across China. Each plant is classified by its primary treatment technology, although individual plants may include multiple facilities or landfill units. Information on plant location, years of operation, treatment capacity, investment, and disposal volumes was systematically collected through extensive online searches, guided by the official registry released by the Ministry of Ecology and Environment of China (https://www.mee.gov.cn/). Key data sources include the Automatic Monitoring Data Public Platform for Household Waste Incineration Power Plants (https://ljgk.envsc.cn/) and the Qichacha (https://www.qcc.com/) enterprise information platform, supplemented by annual local government reports and verified news sources.

Historical MSW-related data from 2000 to 2021. Data on MSW generation and treatment for 352 Chinese cities between 2000 and 2021 were primarily obtained from the China Urban-Rural Construction Statistical Yearbook, as detailed in Table S1.[29,30] Missing observations were addressed using the data-processing methods described in Ma et al.[31,32] This dataset provides a consistent basis for assessing current trends and identifying potential gaps in future MSW treatment capacity at both national and city levels.

Projection of MSW generation from 2021 to 2050. MSW generation is closely linked to socio-economic factors, particularly population and per capita gross domestic product. Thus, we applied a multiple linear regression model to project MSW generation from 2022 to 2050 for 292 cities with sufficient historical population and GDP data. For the remaining 60 cities, projections were generated using an autoregressive integrated moving average model. The projection methods refer to Ma et al.[31,32] The future MSW generation quantities across cities are presented in Table S6, while detailed forecasts of population, GDP per capita, and MSW generation for 2022–2050 are provided in Text S1.

Scenario setting for MSW treatment from 2022 to 2050. Future treatment pathways were defined using a structured, four-step framework to ensure consistency with policy targets, technological trends, and empirical evidence. First, the incineration-focused scenario aligns with China's 14th Five-Year Plan for Urban Domestic Waste Classification and Treatment Facilities[6] and references projections from Ma et al.[31] and Liu et al.,[24] with baseline incineration rates updated using a consistent methodology. Second, the bioconversion-oriented scenario draws on Wang et al.,[14] Liu et al.,[33] and Fang et al.,[34] with food waste bioconversion rates calibrated to MSW composition and their trajectories toward 2050 reset according to city-specific treatment structures. Third, the recyclable material scenario is primarily based on Fang et al.[34] and Mu et al.[35] Finally, a business-as-usual (BAU) scenario and combined strategies such as RR-ID and RR-LBB-ID were developed to examine integrated pathways. Accordingly, we have designed a total of nine distinct MSW treatment scenarios as outlined below. The composition of each scenario is summarized in Table 1. The future MSW disposal structures in various scenarios are presented in Table S7, with the calculation methodology detailed in Figure S1.

- BAU: the future MSW disposal structure remains unchanged from its 2021 configuration throughout the projection period.
- Incineration dominated (ID): the incineration rate continues to increase, reaching near-complete coverage of MSW treatment in China by 2050. This scenario reflects the current national policy orientation that strongly promotes WtE incineration through sustained financial support. Considering the considerable resource and environmental benefits of bioconversion for organic components such as food waste, two additional scenarios emphasizing high-value and refined treatment are proposed:
- Low-rate bioconversion (LB): the bioconversion rates for composting (LBC) and protein recovery (low-rate bioconversion [LBB]) in less-developed and developed cities gradually increase to 30% and 50% by 2050, respectively.
- High-rate bioconversion (HB): the bioconversion rates for composting (HBC) and protein recovery (high-rate bioconversion [HBB]) in less-developed and developed cities rise to 50% and 100% by 2050, respectively.
- In both LB and HB scenarios, organic waste not treated through bioconversion is directed to either incineration or landfilling. Cities are classified as less-developed or developed based on whether their bioconversion rate for organic waste was below or above 1% in 2021.
- Recyclable recovery (RR): recyclable materials, including paper, plastics, glass, textiles, and metals, are recovered rather than incinerated or landfilled, with a recycling rate varying between 0.22 and 0.65.
- Combined scenarios: three integrated scenarios are constructed by combining the above disposal modes: HBB-ID, RR-ID, and RR-HBB-ID. The RR-HBB-ID scenario represents a high-performing resource recovery pathway, in which recyclable materials are recovered for secondary production, food waste undergoes bioconversion for protein recovery, and residual MSW is treated through incineration with energy recovery.

The projected physical composition of MSW was estimated following the methodology established by Ma et al.[32] This approach employs a data-driven model that integrates field investigation data with key socio-economic factors. A back-propagation neural network was used to capture the relationship between waste composition and its underlying drivers, with data preprocessing applied to ensure consistency with compositional data properties. After validation, the model was used to reconstruct historical trends and estimate waste composition patterns for the period 2000–2021. Because future waste composition is influenced by complex and uncertain factors such as economic development, changing lifestyles, and climate impacts, this study assumes that MSW composition remains fixed at its 2021 level, as detailed in Table S3.

The revenue from secondary products and the cost of MSW treatment

The economic performance for MSW disposal strategies ($C^k_{\mathrm{MSW},t}$) in this study include treatment costs and the revenues from products sale ($R^k_{\mathrm{recycle},t}$). The costs for MSW treatment in terms of incineration, landfill, and biochemical disposal ($C^k_{disposal,t}$) involve the fixed investment cost ($C^k_{\mathrm{inv},t}$) and operational costs ($C^k_{\mathrm{ope},t}$).

$$C^k_{\mathrm{MSW},t} = R^k_{\mathrm{recycle},t} + \sum_{disposal} C^k_{disposal,t} \tag{1}$$

$$C^k_{disposal,t} = C^k_{\mathrm{inv},t} + C^k_{\mathrm{ope},t} \tag{2}$$

where *disposal* represents various methods of MSW disposal, including incineration, landfill, and bioconversion. The investment amount for MSW treatment plants is converted into investment cost for processing 1 ton of MSW ($c^k_{\mathrm{inv},t}$), which is then multiplied by the quantity of MSW disposed ($Q^k_{disposal,t}$) for the calculation of fixed investment, to reflect the differences in waste disposal intensity among cities. For the calculation of $Q^k_{disposal,t}$ refer to Text S2.

$$C^k_{\mathrm{inv},t} = c^k_{\mathrm{inv}} \cdot Q^k_{disposal,t} \tag{3}$$

$$c^k_{\mathrm{inv}} = \frac{\sum_i I^k_i}{N_{disposal} \cdot \sum_i Q^k_i} \tag{4}$$

where I^k_i and Q^k_i are the total investment amount and annual MSW disposal quantity, respectively. $N_{disposal}$ is the operational lifespan, which is 20 years for incineration plants, 50 years for landfill sites, and 15 years for biochemical disposal plants, respectively.[18,36] i represents different MSW treatment plants. In Equation 4, only MSW treatment plants in the same city are taken into consideration.

The economic costs of MSW plant investments were derived from our proprietary dataset, and the average investment cost per unit of MSW treatment was subsequently calculated for each city. Notably, there remain 279 cities in China lacking data on bioconversion plants, while 106 cities lack data on incineration plants, and 215 cities lack data on landfill sites. It is assumed that the investment costs for processing 1 ton of MSW in these cities are at the average level of the provinces in which they are located. Moreover, if a province lacks data on MSW treatment plants, the investment costs in the cities of that province are assumed to be at the average level in China.

The operational cost for MSW disposal is calculated by multiplying the quantity of MSW and operational cost for processing 1 ton of MSW including product revenue (r_{product}), subsidies (f_{subsides}), operation and management cost (c_{manage}), energy cost (c^k_{energy}), and other costs (c_{other}). The energy cost for processing 1 ton of MSW differs among cities due to the different prices of electricity and diesel, while others remain fixed. Operational revenues are mainly from secondary products sale for material recycling or produced products for landfill, incineration, and bioconversion. Operational cost and revenues for MSW management were compiled from multiple sources. Data for incineration were obtained from Zhou and Zhang[21] and Zhang et al.,[37] while data for bioconversion were sourced from Liu et al.,[33] Bohm et al.,[38] and Xue et al.[39] Additional parameters for landfill were referenced from official documents, including CNDRC[40] and CMEE.[41] Text S3 explains the specific information regarding operational benefits for MSW incineration, landfill, and biochemical disposal.

$$C^k_{\mathrm{ope},t} = c^k_{\mathrm{ope},t} \times Q^k_{disposal,t} \tag{5}$$

$$c^k_{\mathrm{ope}} = r_{\mathrm{product}} + f_{\mathrm{subsides}} - c_{\mathrm{manage}} - c^k_{\mathrm{energy}} - c_{\mathrm{other}} \tag{6}$$

The technologies for MSW incineration and bioconversion are not yet matured, indicating that they will experience a reduction in capital expenditure, specifically investment cost, due to the effects of accumulated experience and learning.[41] The decreasing ratios of investment costs for these technologies are shown in Figure S2. This rate is also applied to the operation and management cost, given its dependence on investment cost.

Recycling benefits include sorting and transportation cost ($C^k_{\mathrm{ST},t}$) as well as the revenues for secondary products sale ($R^k_{\mathrm{recycle},j,t}$), both of which depend on the quantity of collected recyclable materials ($Q^k_{\mathrm{recycle},j,t}$). The recyclable materials involved in this study include metals, glass, plastics, paper, and textiles. The calculation of $Q^k_{\mathrm{recycle},j,t}$ refers to Text S2.

$$R^k_{\mathrm{recycle},t} = \sum_j R^k_{\mathrm{recycle},j,t} - C^k_{\mathrm{ST},t} \tag{7}$$

$$R^k_{\mathrm{recycle},j,t} = \left(\varphi_j p_{\mathrm{recycle},j} - c_{\mathrm{recycle},j}\right) \times Q^k_{\mathrm{recycle},j,t} \tag{8}$$

$$C^k_{\mathrm{ST},t} = \left(c_{\mathrm{sort}} + c_{\mathrm{transport}}\right) \times \sum_j Q^k_{\mathrm{recycle},j,t} \tag{9}$$

where φ_j is the substitution ratio of recycled materials. $p_{\mathrm{recycle},j}$ is the price of recycled materials. $c_{\mathrm{recycle},j}$ is the cost for processing 1 ton of recyclable materials. c_{sort} and $c_{\mathrm{transport}}$ are the sorting cost and transportation cost for collecting 1 ton of recyclable materials, respectively. j represents different recyclable materials. Moreover, it should be noted that the recycling benefits vary due to the differences in MSW composition across different cities.

Calculation of environmental costs for MSW disposal

To evaluate the environmental impacts of different MSW disposal strategies, we utilized ReCiPe 2016 LCA indicators, which consist of 18 indicators (global warming potential, ozone depletion potential, particulate matter formation potential, photochemical and human oxidant formation potential for ecosystems, ionizing radiation potential, freshwater eutrophication potential, marine eutrophication potential, freshwater ecotoxicity potential, marine ecotoxicity potential, water consumption potential, terrestrial acidification potential, terrestrial ecotoxicity potential, land use, human toxicity potential for cancer, and non-cancer effects, fossil resource scarcity potential, and mineral resource scarcity potential, covering five aspects: air, water, soil, human, and resource depletion, as detailed in Table 2. Chinatax[RCP], a regionalized monetization model applicable to ReCiPe 2016 LCA indicators, was employed to calculate these 18 indicators across cities in China based on the current national environmental tax framework and regional carbon abatement costs, which represent the most policy-relevant valuation benchmark at the time of analysis. Selection of environmental indicators and their corresponding monetization factors refer to Liu et al., as detailed in Table S11.[33] Then, the externality costs for MSW disposal ($E^k_{\mathrm{MSW},t}$) could be calculated based on these 18 indicators and corresponding monetization factors. It is important to note that, while this approach grounds the cost estimates in actual regulatory mechanisms, the potential future evolution of environmental taxation policies is not modeled, representing a defined boundary condition for this long-term assessment. And the externality costs will vary with MSW disposal structures by 2050 due to differences in the emissions of pollutants from various MSW disposal methods.

Table 2. LCA indicators in the ReCiPe model

Type	Name	Unit	Equation
Air related	global warming potential (GWP)	kg CO_2 eq.	S29
	ozone depletion potential (ODP)	kg CFC-11 eq.	S30-S33
	particulate matter formation (PMFP)	kg PM2.5 eq.	S13-S15
	photochemical oxidant formation potential: ecosystems (EOFP)	kg NOx eq.	
	photochemical oxidant formation potential: humans (HOFP)	kg NOx eq.	
	ionizing radiation potential (IRP)	kBq Co-60 eq.	S40-S41
Water related	freshwater eutrophication (FEP)	kg P eq.	S13-S15
	marine eutrophication (MEP)	kg P eq.	
	freshwater ecotoxicity (FETP)	kg 1,4-DCB eq.	S16
	marine ecotoxicity (METP)	kg 1,4-DCB eq.	S17
	water consumption (WCP)	m^3 water eq.	–[a]
Soil related	terrestrial acidification (TAP)	kg SO_2 eq.	S13-S15
	terrestrial ecotoxicity (TETP)	kg 1,4-DCB eq.	S36-S39
	land use (LU)	$m^2 \times$ year annual cropland eq.	S20-S28
Human related	human toxicity potential, cancer (HT_c)	kg 1,4-DCB eq.	S34-S45
	human toxicity potential, non-cancer (HT_{nc})	kg 1,4-DCB eq.	
Resource depletion related	fossil resource scarcity (FFP)	kg oil eq.	S19
	mineral resource scarcity (SOP)	kg Cu eq.	S18

The specific explanation on the LCA indicators in the ReCiPe model refers to the work of Liu et al.[33]
[a]The average resource taxes (or fees) on surface water in each province were used as the monetization factors of the indicator WCP in 31 provinces.

$$E^k_{MSW,t} = \sum_{l=1}^{18}\left(\omega^k_l \times M^k_{l,t}\right) \tag{10}$$

$$M^k_{l,t} = \sum_{disposal}\left(e^l_{disposal,t} \times Q^k_{disposal,t}\right) \tag{11}$$

where ω^k_l is the monetization factor. $M^k_{l,t}$ are the equivalent emissions of pollutants from MSW disposal or recycling. $e^l_{disposal,t}$ is the emission intensity of MSW disposal (incineration, landfill, and biochemical disposal), respectively. l represents different LCA indicators. Moreover, it is noted that the positive impacts of technological improvements on long-term emissions from MSW incineration are considered. Specifically, emissions from MSW incineration are expected to decrease to 90%, 80%, and 70% of that in 2020 by 2030, 2040, and 2050, respectively. The specific calculation of various monetization factors is detailed in Text S4.

Approach to identifying the optimal MSW disposal strategy for each city

To comprehensively evaluate the economic performance and environmental performance of different MSW disposal modes and achieve a city-specific trade-off between these two dimensions, a composite benefit index for various MSW disposal modes is constructed. First, the absolute maximum economic (or environmental) cost value among all scenarios is selected as the benchmark for each city. The economic (and environmental) cost values of each scenario are then normalized relative to this maximum value to derive an economic (and environmental) benefit index. This normalization process aims to eliminate the effects of dimensional differences and measure the relative optimization level of economic (or environmental) performance within the range of all considered scenarios. Subsequently, considering that the environmental performance and economic performance are equally important, the composite benefit index ($SCORE^{k,s}_{MSW,t}$) is defined as the sum of the economic benefit index and the environmental benefit index. Based on this framework, by comparing the composite benefit indices across different scenarios, the optimal MSW disposal strategy that balances both economic and environmental performance can be identified for each city.

$$SCORE^{k,s}_{MSW,t} = \frac{-C^{k,s}_{MSW,t}}{\max_s\left(\left|C^{k,s}_{MSW,t}\right|\right)} + \frac{-E^{k,s}_{MSW,t}}{\max_s\left(\left|E^{k,s}_{MSW,t}\right|\right)} \tag{12}$$

where s represents different scenarios.

Validation and sensitivity analysis

To validate the macro-level accuracy of our city-aggregated bottom-up framework and assess potential deviations, we conducted a two-step verification. First, we compared our aggregated national-level results (e.g., total treatment costs, GHG emissions) for key baseline years with those reported in peer-reviewed national-scale studies (e.g., Liu et al.[33] and Ma et al.[31]). The differences were found to be within a relatively narrow and acceptable range for macro-level scenario analyses. Second, we performed a sensitivity analysis by perturbing key facility-level parameters, such as introducing plausible variation in operational costs and environmental impact intensities within their documented ranges, and re-aggregating to the city and national levels. The resulting variation in total national environmental and economic costs remained within a bounded and limited margin. These analyses confirm that, while micro-heterogeneity among individual plants exists, our aggregation approach does not introduce significant systematic bias at the macro-scale, and the estimated deviations are not substantial enough to alter the comparative ranking or primary conclusions of our scenario analysis.

RESULTS

Trajectories of city-level MSW generation and treatment in China from 2000 to 2050

China's total MSW generation increased from 113 million tons (Mt) in 2000 to 256 Mt in 2021, with significant variation across cities. Wealthier and more populous areas tend to generate more waste, leading to concentration in eastern coastal regions and provincial capitals. In 2022, the top 5 MSW producers were Shanghai, Beijing, Shenzhen, Chongqing, and Chengdu. Of these, Shenzhen experienced the greatest rise in MSW generation from 2000 to 2021, a trend driven by income and population growth, followed by Chongqing and Chengdu.

The historical evolution of MSW treatment structures across 352 cities is detailed in Figure 1. MSW treatment strategies vary across Chinese cities. While most have significantly adopted incineration, others (e.g., Danzhou, Karamay, Panzhihua, and Yuxi) have followed different paths. The shift from landfilling

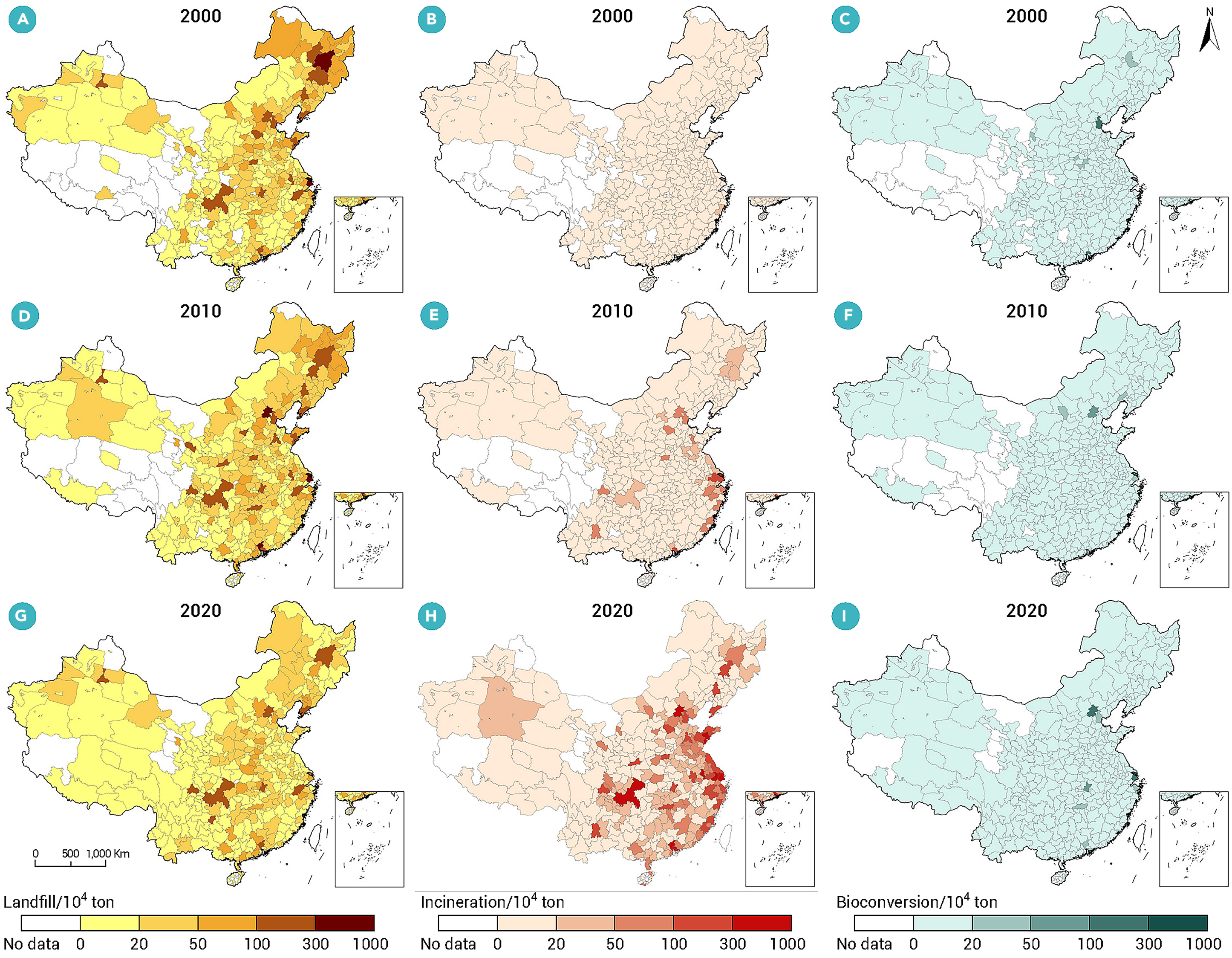

Figure 1. Evolution of MSW treatment in China (A–I) MSW amounts treated by landfill, incineration, and bioconversion for 352 cities in 2000, 2010, and 2020.

to incineration with energy recovery is driven by land scarcity, stricter environmental regulations, and the advantages of incineration in reducing waste volume, recovering heat, and generating power.[12,42,43] By 2021, China's harmless treatment rate for MSW had reached 99.9%, with incineration, landfilling, and bioconversion accounting for 72.5%, 21.1%, and 6.5%, respectively. Notably, 33.8% of cities relied on incineration to treat more than 90% of their MSW, and 46.0% of cities used it for over 80%. High shares of bioconversion were observed in both less-developed cities (e.g., Leshan and Yingkou) and more developed ones (e.g., Beijing and Wuhan).

A panel data regression model, based on projected population and GDP per capita, estimates that MSW generation across the 352 cities will reach 358.6 Mt by 2050, a 52.5% increase from 2020, aligning with earlier projections by Wei et al.[44] The projected annual growth rate of 1.4% from 2022 to 2050 is similar to the World Bank's estimate of 1.7% for the East Asia-Pacific region, but lower than the historical rate of 1.9% from 2000 to 2021. By 2050, Shenzhen, Wuhan, and Shanghai are each projected to generate over 10 Mt of MSW annually, followed by Chengdu at 9.6 Mt and Beijing at 8.6 Mt. Nevertheless, waste growth in these major cities is expected to slow after 2021. In contrast, several cities in central and western China, such as Dazhou, Zhongwei, Haidong, Xuchang, and Chuzhou, are projected to experience rapid growth, with MSW generation increasing more than 3-fold. This pattern reflects the general association between city size and waste generation, although this link is expected to weaken as urbanization slows.[45]

To systematically identify feasible MSW treatment strategies for cities across China, we designed and developed nine scenarios based on combinations of four principal disposal methods: landfilling, incineration, bioconversion (for composting and protein recovery), and recyclable materials recovery. For instance, in the ID scenario, incineration increases to 95.8% by 2030 and reaches 99.3% by 2050. Meanwhile, the HBB and LBB scenarios assume that 81.0% and 52.2% of organic waste, respectively, will go to protein recovery via bioconversion by 2050. By 2050, developed cities (including direct-administered municipalities, provincial capitals, and pilots such as Taizhou and Deyang) are expected to treat over 50% of MSW through bioconversion. In contrast, many less-developed cities and other pilots (e.g., Guangyuan and Yichun) will likely still rely mainly on incineration. The RR-HBB-ID scenario offers an ideal integrated framework. It maximizes resource use by combining three streams: recycling materials (e.g., paper, plastics, textiles, metals, and glass), converting food waste at high rates to recover protein, and incinerating residual waste for energy. This approach is based on current city practices and each city's level of bioconversion technology.

Environmental costs in future MSW treatment transitions

Physical indicators provide key insights into the environmental burdens and trade-offs of each waste treatment scenario. Under the BAU scenario, GHG emissions from MSW treatment are expected to decrease by about 7.0% from 2020 to 2050, mainly due to technological advancements. The ID strategy achieves a more pronounced reduction, lowering GHG emissions by 19.2% compared with BAU by 2050, which is largely attributable to the mitigation of landfill methane. The most significant emission cuts are associated with WtM strategies. Notably, the RR-HBB-ID scenario attains up to 85.5% GHG

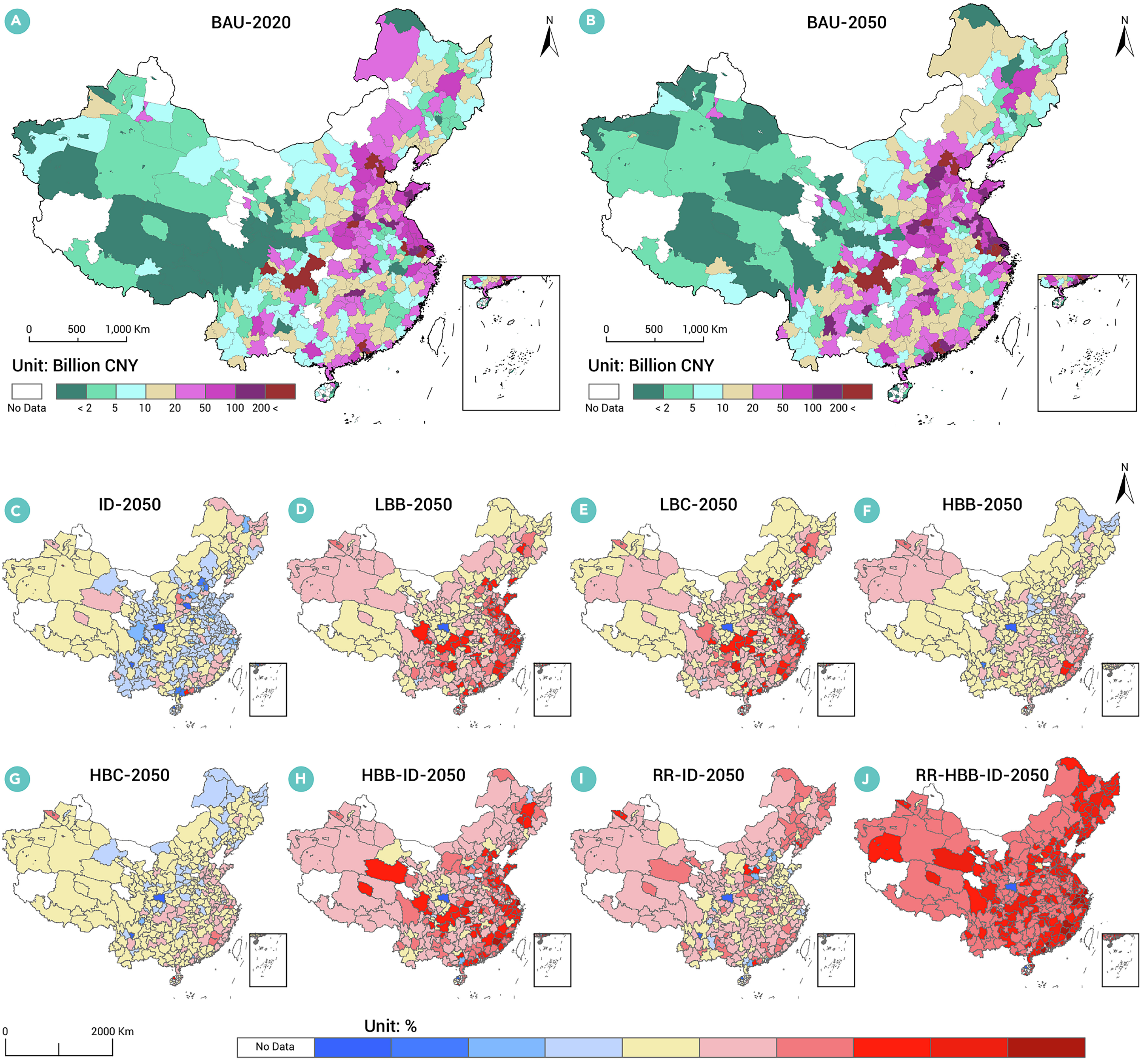

Figure 2. Comparison of environmental costs at city-level across different scenarios (A and B) The monetized environmental costs in 2020 and 2050 under the business-as-usual (BAU) scenario, respectively, based on the assessment of 18 environmental impact categories associated with MSW treatment. (C–J) The differences in environmental costs between the BAU scenario and eight alternative waste-to-energy (WtE) and waste-to-material (WtM) scenarios in 2050. The red color highlights significant reductions in environmental costs when transitioning from the BAU scenario to WtE or WtM strategies.

reduction through improved resource recovery and the avoidance of emissions from conventional disposal pathways. Advanced incineration with energy recovery offers a relative advantage in fossil resource conservation, with the potential to save approximately 2,700 tons of oil equivalent by substituting primary fuel extraction. Spatial analysis highlights a critical trade-off: although incineration reduces carbon footprints, it may intensify regional air pollution and aquatic toxicity in regions with insufficient emission controls. In contrast, large-scale composting and bioconversion enhance carbon sequestration and soil health but necessitate diligent management to avert nutrient runoff and freshwater eutrophication. These results confirm that optimizing the environmental performance of waste management requires alignment with regional priorities, establishing a foundation for integrated physical and economic assessments. National and city-specific environmental impacts are detailed in Figure S3 and the supplemental information (extended data), respectively.

To quantify environmental externalities in the MSW management system, we convert 18 environmental impact indicators into monetary values using region-specific monetization factors. The significantly higher environmental costs of incineration and landfilling make a strong case for shifting to WtM approaches. Specifically, landfilling incurs environmental costs approximately 1.8 times those of incineration and 228 times higher than traditional composting. In contrast, bioconversion for protein recovery can generate net environmental benefits. Our scenario evaluations, based on full life cycle climate and environmental impacts, provide a standardized framework for designing policy instruments, such as environmental taxes and compensation mechanisms. Comparative environmental costs at the city level are shown in Figure 2.

Under the BAU scenario, total environmental costs increase by 23.0% from 2020 to 2050, primarily driven by growing MSW treatment volumes (Figure 1). However, by 2030, costs stabilize near 2020 levels as reductions

in landfill impacts offset higher incineration costs. Shifts to WtE or WtM strategies achieve significant reductions in environmental costs by 2050, with declines ranging from 36.9% for ID scenario to 78.2% for the RR-HBB-ID scenario. However, bioconversion for composting and protein recovery shows minimal cost divergence (Δ ≈ 2%), reflecting reduced reliance on incineration, which lowers its external costs.

There is significant variability in environmental costs across cities, with the greatest burdens concentrated in economically developed and densely populated urban areas. In 2020, Beijing, Tianjin, Suzhou, Chongqing, and Nanjing recorded the highest environmental costs. By 2050, under the BAU scenario, the ranking is expected to change, with Beijing, Tianjin, Suzhou, Wuhan, and Chengdu showing the highest impacts. These megacities have high MSW treatment demands, mainly met through incineration and landfilling, which significantly increase their environmental footprints. While Suzhou and Tianjin generate less MSW than Chongqing, their higher GDP per capita results in greater environmental costs per unit of waste. Rising costs in Wuhan and Chengdu are linked to demographic and economic factors, amplified by national development initiatives such as the Western Development Plan and the Belt and Road infrastructure project. Cities in central and eastern China see the greatest gains from bioconversion adoption, especially in protein recovery compared with composting. However, cities in the western regions may face rising environmental costs due to their continued dependence on incineration for waste disposal.

Environmental impact trade-offs are critically shaped by indicator variability (Figure 3). Freshwater ecotoxicity contributes over 98.8% to total environmental costs, while soil-related impacts (particularly terrestrial ecotoxicity) exert net benefits offsetting 4.6%–15.2% of the costs. Bioconversion methods, including composting and protein recovery, significantly reduce freshwater ecotoxicity and enhance resource recovery versus BAU/ID scenarios. However, hybrid WtM-WtE scenarios (HBB-ID and RR-HBB-ID) perform worse than standalone WtE in terms of mineral conservation and human health protection. This limitation arises from the limited capacity of biochemical products (compost and protein substitutes) to fully replace traditional products such as fertilizers and animal-derived proteins. Our findings highlight that substantial life cycle environmental cost reductions require prioritizing biochemical treatment and overcoming substitution barriers.

Economic-environmental trade-offs in MSW treatment transition

The net economic benefit of each MSW treatment scenario was assessed as the difference between total revenues (from energy/products and government subsidies) and total costs (including investment, operation, management, and other expenses). The formula is: net economic benefit = (product income + subsidies) − (investment + O&M costs + other costs). Notably, a negative economic benefit represents a net cost of MSW treatment. First, investment data for 1,954 MSW treatment plants across 352 cities in China were collected (Figure 4). With MSW disposal fees ranging from CNY 50–200 per ton, significantly lower than environmental costs, targeted environmental taxes or subsidies could incentivize the adoption of WtM strategies. The increasing adoption of waste incineration and bioconversion has notably reduced environmental burdens. While most cities see modest economic gains in all scenarios, disparities between profitable and unprofitable cities widen in WtE and WtM scenarios. As shown in Figure 5, cities operating at a deficit face increased financial pressure, while profitable cities see greater benefits, especially under the HBB-ID and RR-HBB-ID scenarios. These differences are mainly due to variations in facility utilization rates, which affect unit investment costs for biochemical disposal and influence the economic viability of WtE and WtM strategies. For example, cities such as Chongqing, Kunming, Guangzhou, and Dongguan achieve substantial economic benefits under the HBB-ID scenario, whereas Dalian, Harbin, Jinan, Shanghai, Shenzhen, and Suzhou incur higher economic costs. The BAU and ID scenarios impose relatively smaller economic burdens. Some cities, including Shanghai, Suzhou, and Dalian, even achieve modest gains under these scenarios, largely due to lower investment costs associated with incineration.

As shown in Figures 5A and 5B, MSW treatment pathways in China vary significantly across scenarios. From 2020 to 2050, environmental costs rise under the BAU scenario but decrease substantially when shifting to WtE and WtM scenarios, with WtM proving particularly cost-effective. While economic costs under the BAU scenario increase by 125.6% from 2020 to 2050, the implementation of WtM strategies could lead to a 189% reduction compared with 2020. At the national level, the RR-HBB-ID scenario is identified as the most effective for reducing environmental costs, while the RR-ID scenario performs best in improving economic benefits. The combined application of these strategies could lead to a 78.3% reduction in environmental costs and a 33.9% increase in economic benefits by 2050. Consequently, these findings provide strong support for pivoting from landfill-dependent systems toward integrated WtE and WtM approaches.

In general, Chinese cities exhibit four distinct cost profiles shaped by differentials in waste volume, composition, and local infrastructure. Megacities such as Beijing and Shanghai focus on waste reduction, even though they face higher operational costs. Coastal cities with strong regulatory frameworks, such as Xiamen and Quanzhou, maintain low environmental costs through well-managed waste systems. Inland industrial cities such as Zhengzhou have higher environmental costs, mainly due to their continued dependence on landfilling. Cities such as Xi’an and Hanzhong benefit from advantageous waste composition, characterized by lower organic content and elevated shares of paper and wood, which collectively mitigate contamination risks and reduce treatment expenses. This marked regional variation underscores the need for city-specific waste management strategies. Megacities are best served by integrated or rapid-reduction treatment solutions, while smaller cities can improve outcomes by aligning treatment technologies with local waste characteristics and modernizing infrastructure.

City-specific solutions in WEE nexus development

Our city-level analysis reveals that synergistic waste treatment pathways, particularly the RR-HBB-ID model, strike the optimal balance between economic and environmental costs and are poised to become the dominant future strategy. As shown in Figure 6, macro-scale projections further indicate that, by 2030, 141 and 73 cities are projected to adopt the RR-HBB-ID and RR-ID models, respectively. By 2050, the number of cities implementing the RR-HBB-ID model is expected to reach 156, with an additional 91 and 49 cities adopting the RR-ID and HBB-ID models, while only 43 cities are anticipated to retain the singular ID mode. This indicates that integrated MSW treatment strategies, including RR-HBB-ID, RR-ID, and HBB-ID, are the most optimal and cost-effective solutions for over 84% of the cities studied. This trend underscores a fundamental shift from traditional, discrete treatment modalities toward integrated pathways that combine energy recovery with material recycling.

However, by 2050, most cities will face a trade-off between economic and environmental performance, requiring local policymakers to choose between the economically focused RR-ID strategy and the environmentally focused RR-HBB-ID strategy. For instance, cities with greater economic sensitivity, such as Jilin and Jinan, are better suited for the RR-ID strategy, while those focusing on environmental goals, such as Xiamen and Weihai, will benefit more from the RR-HBB-ID strategy. This difference is mainly due to significant variations in the economic costs of applying bioconversion technologies across cities. Notably, for cities such as Beijing, Chongqing, and Chengdu, the RR-HBB-ID model offers an optimal solution, balancing both economic and environmental benefits.

Overall, our analysis points to a nationwide transition, shifting from heavy reliance on WtE conversion to an integrated resource recovery system that develops both WtE and WtM pathways together. This synergistic approach delivers substantial environmental benefits at a manageable economic cost, proving more cost-effective and sustainable than energy-focused pathways alone. This transition will accelerate GHG mitigation and represent a significant step forward for urban waste management in China, moving closer to circular economy goals.

DISCUSSION

MSW management is closely tied to key global challenges such as climate change, resource scarcity, and public health. As the world’s largest producer of MSW, China has focused on waste incineration, especially in high-density cities such as Shanghai, Shenzhen, and Guangzhou. This focus is driven by incineration’s efficiency in reducing waste volume, generating reliable energy, and addressing waste accumulation quickly. In contrast, bioconversion technologies promote sustainability through resource recycling, GHG reduction,

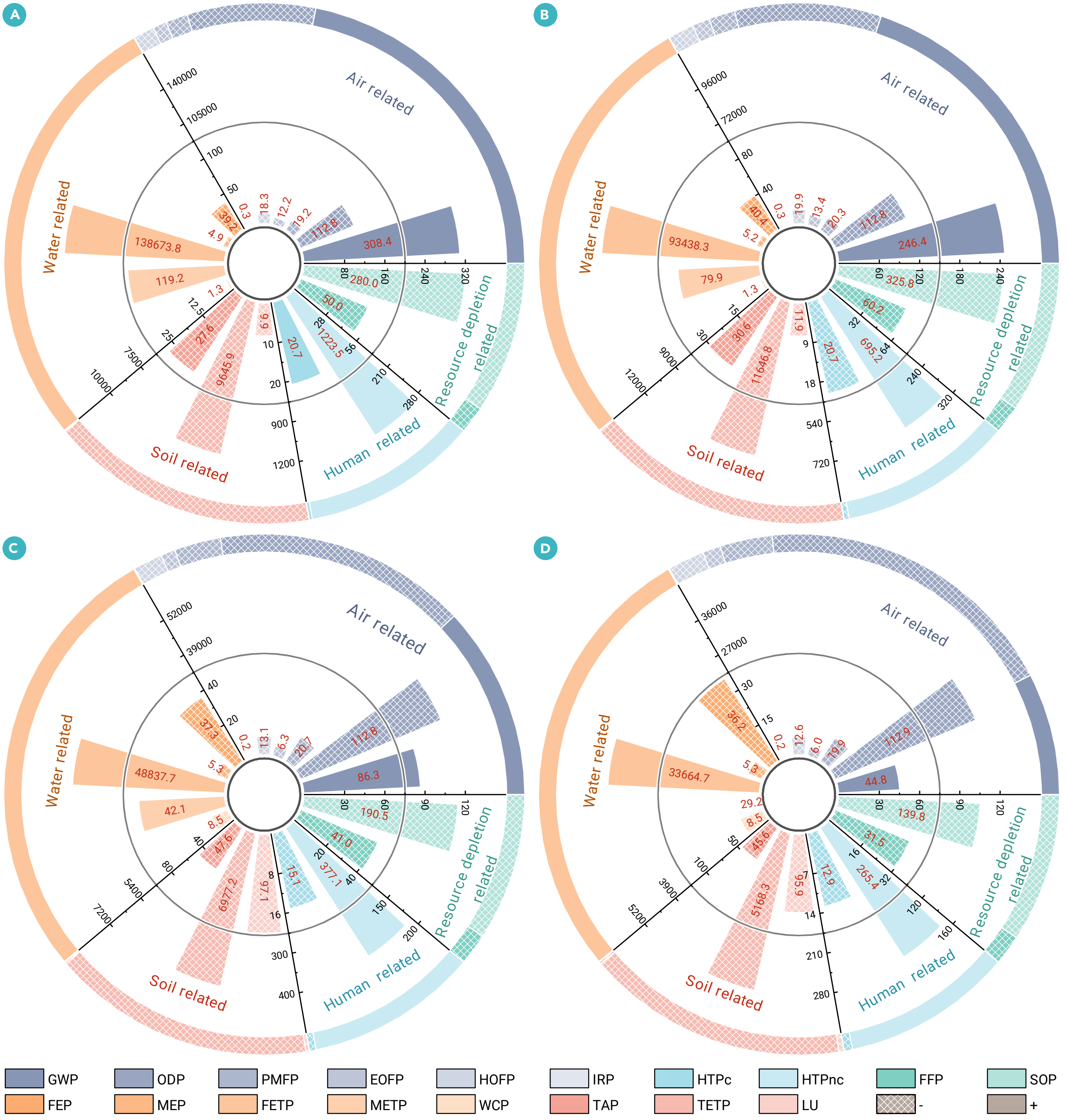

Figure 3. Monetization results of environmental costs in different scenarios (A–D) The monetization results of various environmental cost categories in 2050 under the BAU, ID, HBB-ID, and RR-HBB-ID scenarios. The color-coded bars and rings represent different environmental cost indicators, with white grids indicating environmental benefits and solid colors representing environmental costs. The height of each bar corresponds to the specific monetized value of the respective indicator, while the length of each ring reflects the relative contribution of the indicator within its category. The environmental indicators evaluated in this study include: global warming potential (GWP), ozone depletion potential (ODP), particulate matter formation potential (PMFP), photochemical oxidant formation potential for ecosystems (EOFP) and humans (HOFP), ionizing radiation potential (IRP), freshwater eutrophication potential (FEP), marine eutrophication potential (MEP), freshwater ecotoxicity potential (FETP), marine ecotoxicity potential (METP), water consumption potential (WCP), terrestrial acidification potential (TAP), terrestrial ecotoxicity potential (TETP), land use (LU), human toxicity potential for cancer (HTc) and non-cancer effects (HTnc), fossil resource scarcity potential (FFP), and mineral resource scarcity potential (SOP).

and value-added products, but are limited by high operational costs, slow processing speeds, and scalability challenges. This technological divide reflects the tension between urgent disposal needs and long-term environmental goals.

China has made significant progress in addressing its historical "waste siege," shifting the core issue from inadequate treatment capacity to structural imbalances and the need for improved resource recovery. By 2024, China's harmless MSW treatment capacity reached 1.16 million tons per day, well above the

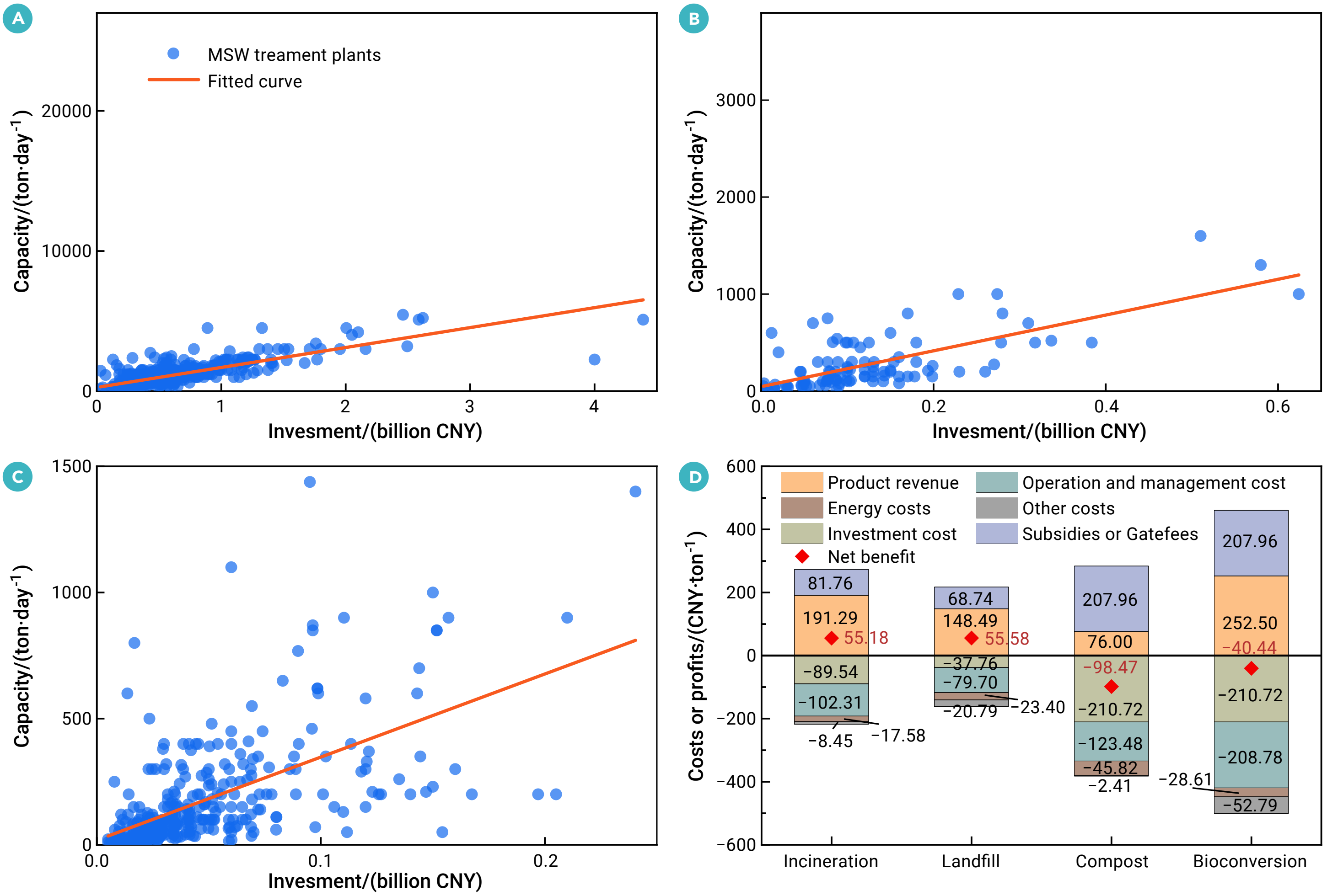

Figure 4. Investment for MSW plants and economic costs (or benefits) of different MSW disposal methods (A–C) Investment amounts and disposal capacities for incineration plants, biochemical disposal plants, and landfill sites in this study. The orange lines represent the fitting curves between investment and capacity for the three kinds of MSW disposal facilities. Additionally, the three panels share identical *x* and *y* axis scaling. (D) Benefits and costs of incineration, landfill, traditional compost and bioconversion for 1 ton of MSW. The colors of the bars denote various revenue and cost items, while the red five-pointed stars represent the net benefit.

annual waste volume of 262 million tons, leaving incineration plants operating at about 60% capacity.[46,47] Following the Implementation Plan for the Domestic Waste Sorting System in 2017, MSW classification has been widely implemented, targeting full urban coverage by 2025,[48] while the 14th Five-Year Plan mandates a recycling rate of no less than 35%,[49] marking a policy evolution from end-pipe reduction toward integrated source reduction and whole-process resource utilization. However, downstream recycling, refined management, and high-value conversion remain underdeveloped due to profit-driven enterprise behavior, resulting in slow policy implementation and unresolved environmental externalities. This highlights the need for innovative approaches that account for environmental costs and promote sustainable MSW transitions.

Notably, over 84% of Chinese cities achieve optimal cost-effectiveness under the RR-HBB-ID, RR-ID, and HBB-ID scenarios, suggesting that hybrid systems are the most practical solution for most cities. Furthermore, the unexpected net benefits from soil-related impacts show that WtM strategies provide ecosystem services beyond waste management, such as soil restoration and improved agricultural sustainability, aligning waste management with broader goals such as land degradation neutrality and climate adaptation. This study confirms that the RR-HBB-ID approach minimizes both economic and external costs, while achieving the most significant reductions in key environmental impacts, such as GHG emissions and aquatic toxicity.

However, effective implementation requires city-specific solutions. In northern cities with harsh climates and slow waste classification progress, such as Harbin and Datong, regulated incineration remains viable. Developed northern cities, such as Beijing, Jinan, and Tianjin, should adopt hybrid systems combining advanced biological treatment with limited incineration. The Yangtze River Delta should focus on insect-based bioconversion and anaerobic digestion, supported by environmental taxes before 2030. Less-developed southern cities with high populations may implement composting systems with environmental taxes after 2030. Smaller cities need customized solutions that fit their local infrastructure and resource limitations.

Our analysis highlights the importance of technological complementarity in a synergistic hybrid system, rather than advocating for a complete replacement of incineration with biotreatment in megacities. Incineration is crucial for base-load treatment, stabilizing waste streams, and mitigating risks, while WtM bioprocessing focuses on source-separated organics to improve resource recovery and reduce environmental impacts.

We further propose differentiated transition pathways tailored to city types such as megacities, high-density coastal cities, and smaller inland cities, considering factors such as land availability, waste composition, and regional socioeconomic conditions. The shift toward integrated WtE and WtM systems will be gradual, relying on the development of sorting systems, technological advances, cost reductions, and supportive policies. This section provides a decision-support framework for optimizing long-term waste management strategies within the limits of treatment capacity, rather than promoting a one-size-fits-all solution.

Notably, emerging bioconversion technologies advance circular economy objectives by producing alternative protein feeds and reducing reliance on conventional agricultural systems, while also underscoring the need to transition from traditional thermal treatment methods. Composting requires low initial investment but is highly dependent on the marketability and distribution efficiency of the compost. In contrast, anaerobic digestion, on the other hand, requires significant capital investment, with profitability tied to long-term

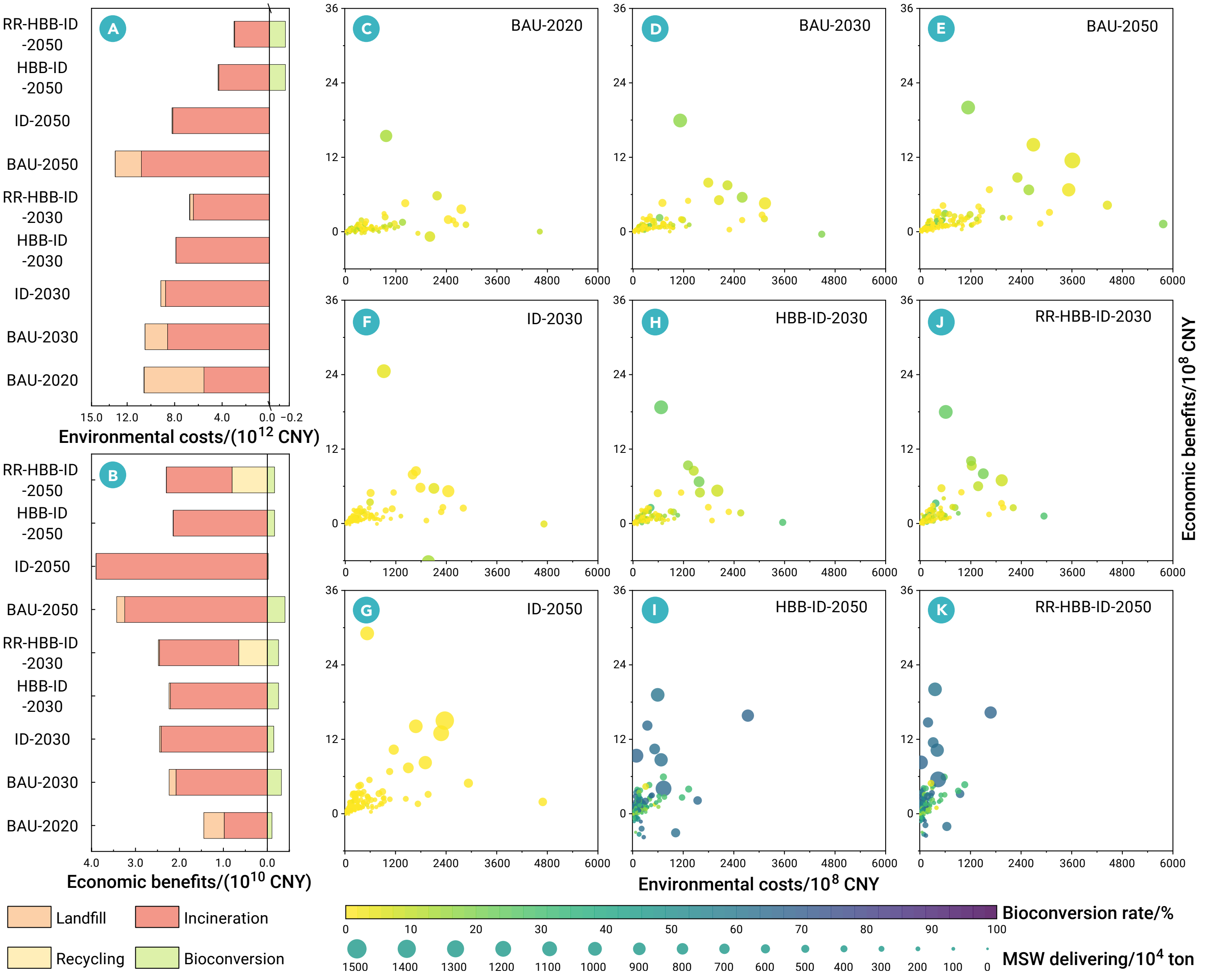

Figure 5. Economic benefits and environmental costs at city and national levels across scenarios (A and B) National-level environmental costs and economic benefits, respectively, across scenarios. The colors of the bars denote various MSW disposal methods, including incineration, landfill, biochemical disposal, and recyclable materials recycling. (C–K) City-level economic benefits and environmental costs under different scenarios. The color of each dot represents the biochemical disposal rate as a percentage of total MSW disposal, while the dot size corresponds to the quantity of MSW generated. It is important to note that this study does not account for the environmental costs associated with the recycling process or the environmental benefits derived from the use of recycled materials, which may result in slightly higher environmental costs in scenarios RR-HBB-ID and RR-ID. Additionally, a few cities, such as Beijing and Shenzhen, are excluded from the graphs due to their significantly higher economic benefits or environmental costs compared with other cities, which would otherwise distort the visualization.

energy revenues from biogas use. Protein recovery such as black soldier fly systems employs a diversified income model that combines waste treatment fees with the sale of valorized products, notably insect protein and organic fertilizers, capitalizing on the substantial commercial potential of insect-based commodities.

Metropolitan areas face dual challenges of limited land and public opposition, leading to the adoption of integrated waste treatment parks or small, decentralized facilities within urban infrastructure, such as underground systems or co-located transfer stations, to fit high-density areas. While urban kitchen waste streams provide a consistent feedstock, operational flexibility remains essential to manage input fluctuations.

Consequently, future MSW strategies will favor context-specific integration of complementary processes, such as combining black soldier fly bioconversion with anaerobic digestion or co-composting, to maximize waste reduction, resource recovery, and energy generation, supporting zero-waste cities and circular economy goals. However, constraints such as high operational costs, intensive preprocessing needs, and the lack of standardized product regulations still hinder widespread adoption. To fully realize the potential of biological waste treatment, enhanced policy support, technological improvements, and institutional innovation are necessary to align economic incentives with environmental goals and progress toward a sustainable, resource-efficient waste management system.

To support a targeted transition, region-specific environmental taxes should be imposed on incineration and landfilling, with revenues directed to subsidize WtM infrastructure in underserved areas. Current subsidies for WtE technologies should be gradually redirected to support WtM pathways, especially bioconversion, using market-based incentives such as feed-in tariffs for bio-products and inclusion in carbon credit systems. National standards and certification systems for compost and insect protein products must be established to build market confidence and support the scaling of secondary resource circulation. These coordinated measures would internalize environmental externalities, correct current market failures, and accelerate the shift toward a circular waste management system.

Policy implementation must fully account for regional heterogeneity. Our analysis reveals substantial variations in waste composition, environmental

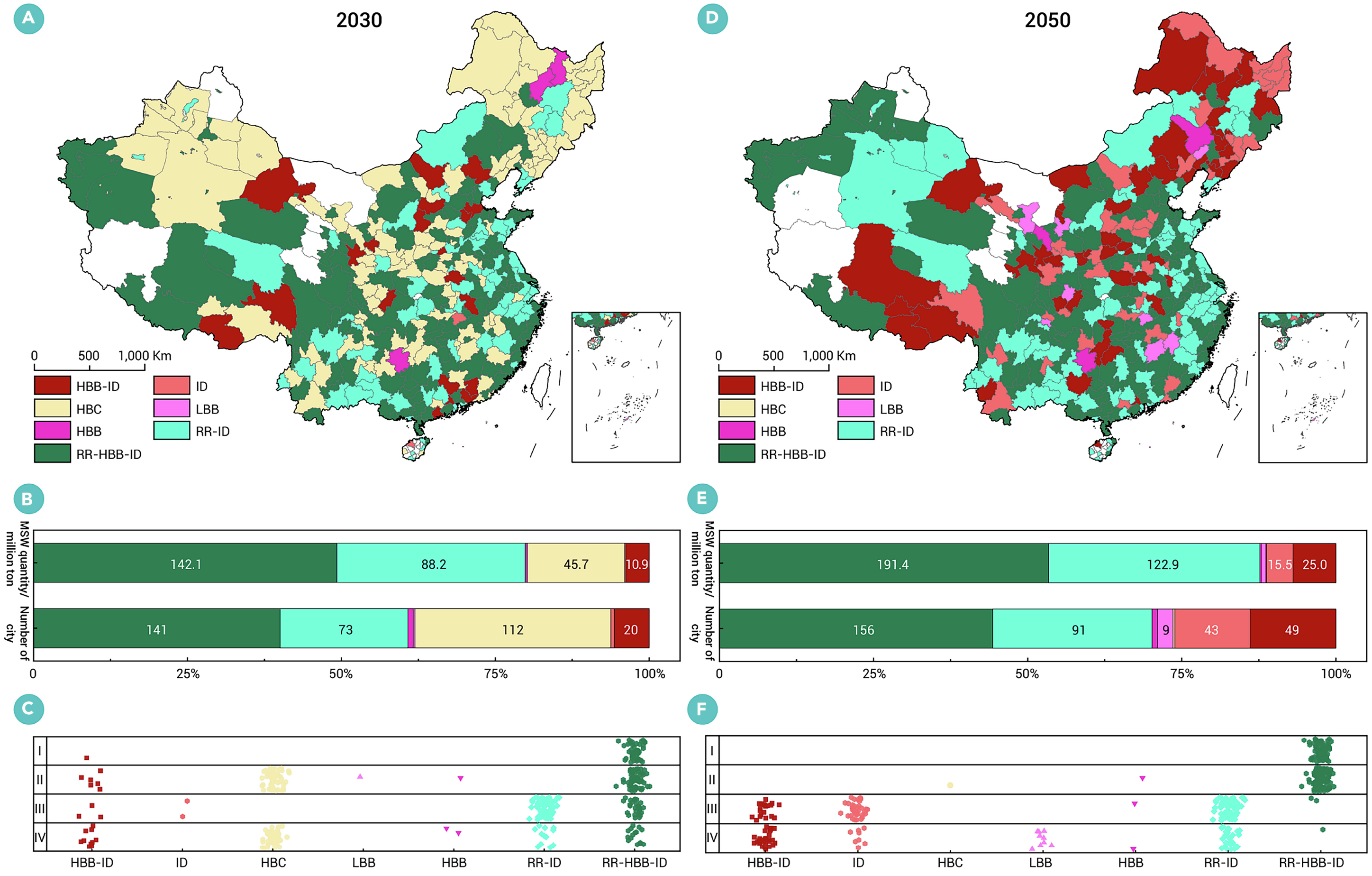

Figure 6. Cost-effective MSW treatment solutions across China's cities (A and D) City-level optimal MSW treatment strategies balancing economic and environmental performance in 2030 and 2050, respectively. (B and E) The national penetration rates of various MSW treatment strategies, respectively, in 2030 and 2050, categorized by both the number of cities and MSW treatment quantity. The red numbers represent the number of cities and MSW treatment quantity of the other strategies. (C and F) The relationship between the comprehensive optimal MSW treatment strategy and both environmentally optimal and economically optimal strategies across cities. I shows that the environmentally optimal strategy and the economically optimal strategy are the same. II and III show that the comprehensive optimal strategy is consistent with the environmentally or economically optimal strategy, respectively. IV shows that the comprehensive optimal strategy is neither an environmentally nor an economically optimal strategy, which means a compromise strategy. The colors of above panels represent different MSW treatment strategies.

carrying capacity, and economic development levels across Chinese cities, necessitating place-based policy design. In wealthier and environmentally sensitive eastern cities, integrated RR-HBB-ID strategies should be prioritized. Western cities that still rely on incineration need adjusted environmental taxes to manage transition costs, along with technology transfer and financial support. For central cities facing economic and environmental trade-offs, a flexible transition mechanism could provide a choice between RR-ID and RR-HBB-ID pathways based on local conditions.

Furthermore, we recommend implementing a "transition dynamics management" system, where city-level monitoring and evaluation regularly update strategy portfolios to stay aligned with technological advancements and urban development needs. This precision-targeted, adaptive governance framework would accelerate China's "zero-waste cities" initiative and provide a replicable policy model for MSW transitions worldwide.

This study has several limitations. First, it does not fully incorporate the economic costs and practical impacts of MSW sorting policies. While some direct costs are included in our operational estimates, modeling the effectiveness of sorting remains difficult due to data limitations and systemic complexities. Second, the analysis assumes a constant MSW composition after 2021 as a practical baseline. This simplifies the assessment of treatment strategy transitions, although waste composition will evolve with socio-economic and policy changes. Developing dynamic predictive models for waste streams will be key to improving long-term strategic planning. Third, heterogeneity in technology, operational cost, and environmental impact among MSW treatment plants remains uncharacterized. Future work will aim to incorporate these factors to improve analytical accuracy.

RESOURCE AVAILABILITY

Materials availability

This study did not generate new unique materials.

Data and code availability

- Source data are all provided with this paper in the supplementary files.
- The code that supports the findings of this study are available from the corresponding author upon reasonable request.

FUNDING AND ACKNOWLEDGMENTS

This study is supported by the National Natural Science Foundation of China (reference nos. 72321002, 72573021, 72141302, 72104023, 72533002, 72222017, 72504023, and 72243001), the Natural Science Foundation of Hebei Province of China (reference no. G2025207029), Key Research Projects of Philosophy and Social Sciences of China Ministry of Education (reference no. 21JZD027), Beijing Natural Science Foundation (reference no. 9254036), and the China Postdoctoral Science Foundation (reference no. 2024M764152).

AUTHOR CONTRIBUTIONS

Z.W. conceived of and designed the study. F.L., X.L., X.L., Z.L., and S.M. processed and analyzed data. H.L. and B.L. led the writing of the paper. B.G. and C.Z. contributed to the review and revision.

DECLARATION OF INTERESTS

The authors declare no competing interests.

SUPPLEMENTAL INFORMATION

It can be found online at https://doi.org/10.1016/j.xinn.2026.101315.

REFERENCES

1. Gautam, M. and Agrawal, M. (2021). Greenhouse gas emissions from municipal solid waste management: A review of global scenario. In Footprint Case Studies: Municipal Solid Waste Management, Sustainable Road Transport and Carbon Sequestration, S.S. Muthu, ed. (Springer), pp. 1–25
2. Gómez-Sanabria, A. and Lindl, F. (2024). The crucial role of circular waste management systems in cutting waste leakage into aquatic environments. *Nat. Commun.* **15**:5443. DOI:10.1038/s41467-024-49555-9
3. Zhang, C., Dong, H., Geng, Y. et al. (2022). Machine learning based prediction for China's municipal solid waste under the shared socioeconomic pathways. *J. Environ. Manage.* **312**:114918. DOI:10.1016/j.jenvman.2022.114918
4. Fan, X., Chu, Z., Chu, X. et al. (2023). Quantitative evaluation of the consistency level of municipal solid waste policies in China. *Environ. Impact Assess. Rev.* **99**:107035. DOI:10.1016/j.eiar.2023.107035
5. Zhang, J., Du, H., Wang, T. et al. (2024). Tracking the carbon flows in municipal waste management in China. *Sci. Rep.* **14**:1471. DOI:10.1038/s41598-024-51698-0
6. The National Development and Reform Commission of China & the Ministry of Housing and Urban-Rural Development of China (NDRCC & MHURDC) (2021). 14th Five-Year Plan for the Development of Urban Domestic Waste Classification and Treatment Facilities. https://www.ndrc.gov.cn/fzggw/jgsj/zys/sjdt/202105/t20210514_1279909.html
7. Zhou, Y., Shan, Y., Guan, D. et al. (2020). Sharing tableware reduces waste generation, emissions and water consumption in China's takeaway packaging waste dilemma. *Nat. Food* **1**:552–561. DOI:10.1038/s43016-020-00145-0
8. Song, J., Sun, Y. and Jin, L. (2017). PESTEL analysis of the development of the waste-to-energy incineration industry in China. *Renew. Sustain. Energy Rev.* **80**:276–289. DOI:10.1016/j.rser.2017.05.066
9. Ding, Y., Zhao, J., Liu, J.W. et al. (2021). A review of China's municipal solid waste (MSW) and comparison with international regions: Management and technologies in treatment and resource utilization. *J. Clean. Prod.* **293**:126144. DOI:10.1016/j.jclepro.2021.126144
10. Cui, W., Su, H., Liu, X. et al. (2024). Resource utilization potential of municipal solid waste affects the sustainable development goals progress in China. *Resour. Conserv. Recycl.* **205**:107562. DOI:10.1016/j.resconrec.2024.107562
11. Singh, C.K., Kumar, A. and Roy, S.S. (2018). Quantitative analysis of the methane gas emissions from municipal solid waste in India. *Sci. Rep.* **8**:2913. DOI:10.1038/s41598-018-21326-9
12. Gómez-Sanabria, A., Kiesewetter, G., Klimont, Z. et al. (2022). Potential for future reductions of global GHG and air pollutants from circular waste management systems. *Nat. Commun.* **13**:106. DOI:10.1038/s41467-021-27624-7
13. Chen, X., Li, J., Liu, Q. et al. (2022). Emission characteristics and impact factors of air pollutants from municipal solid waste incineration in Shanghai, China. *J. Environ. Manage.* **310**:114732. DOI:10.1016/j.jenvman.2022.114732
14. Wang, Z., Wang, S., Li, H. et al. (2023). Synergistic effects of economic benefits, resource conservation and carbon mitigation of kitchen waste recycling from the perspective of carbon neutrality. *Resour. Conserv. Recycl.* **199**:107262. DOI:10.1016/j.resconrec.2023.107262
15. Li, Z., Fan, T.W., Lun, M.S. et al. (2024). Optimization of municipal solid waste incineration for low-NOx emissions through numerical simulation. *Sci. Rep.* **14**:19309. DOI:10.1038/s41598-024-69019-w
16. Tong, H., Shen, Y., Zhang, J. et al. (2018). A comparative life cycle assessment on four waste-to-energy scenarios for food waste generated in eateries. *Appl. Energy* **225**:1143–1157. DOI:10.1016/j.apenergy.2018.05.062
17. Bian, R., Chen, J., Zhang, T. et al. (2022). Influence of the classification of municipal solid wastes on the reduction of greenhouse gas emissions: A case study of Qingdao City, China. *J. Clean. Prod.* **376**:134275. DOI:10.1016/j.jclepro.2022.134275
18. Hoy, Z.X., Woon, K.S., Chin, W.C. et al. (2023). Curbing global solid waste emissions toward net-zero warming futures. *Science* **382**:797–800. DOI:10.1126/science.adg3177
19. Nuss, P., Bringezu, S. and Gardner, K.H. (2012). Waste-to-materials: the longterm option. In Waste to Energy: Opportunities and Challenges for Developing and Transition Economies, A. Karagiannidis, ed. (Springer), pp. 189–205
20. Li, K., Ward, H., Lin, H.X. et al. (2024). Economic viability requires higher recycling rates for imported plastic waste than expected. *Nat. Commun.* **15**:7578. DOI:10.1038/s41467-024-51923-4
21. Zhou, Z. and Zhang, L. (2022). Sustainable waste management and waste to energy: Valuation of energy potential of MSW in the Greater Bay Area of China. *Energy Policy* **163**:112857. DOI:10.1016/j.enpol.2022.112857
22. Ng, W.P.Q., Lam, H.L., Varbanov, P.S. et al. (2014). Waste-to-Energy (WTE) network synthesis for Municipal Solid Waste (MSW). *Energy Convers. Manag.* **85**:866–874. DOI:10.1016/j.enconman.2014.01.004
23. Zargar, T.I., Alam, P., Khan, A.H. et al. (2023). Characterization of municipal solid waste: Measures towards management strategies using statistical analysis. *J. Environ. Manage.* **342**:118331. DOI:10.1016/j.jenvman.2023.118331
24. Liu, B., Wang, P., Zhou, J. et al. (2024). Refocusing on effectiveness over expansion in urban waste-energy-carbon development in China. *Nat. Energy* **10**:215–225. DOI:10.1038/s41560-024-01683-8
25. Zhou, C., Yang, G., Ma, S. et al. (2021). The impact of the COVID-19 pandemic on waste-to-energy and waste-to-material industry in China. *Renew. Sustain. Energy Rev.* **139**:110693. DOI:10.1016/j.rser.2020.110693
26. Thu, L.T., Nguyen, L.B., Guillaume, B. et al. (2016). Quantifying environmental externalities with a view to internalizing them in the price of products, using different monetization models. *Resour. Conserv. Recycl.* **109**:13–23. DOI:10.1016/j.resconrec.2016.01.018
27. Amadei, A.M., De Laurentiis, V., Sala, S. et al. (2021). A review of monetary valuation in life cycle assessment: State of the art and future needs. *J. Clean. Prod.* **329**:129668. DOI:10.1016/j.jclepro.2021.129668
28. Arfan, M., Eriksson, O., Wang, Z. et al. (2023). Life cycle assessment and life cycle costing of hydrogen production from biowaste and biomass in Sweden. *Energy Convers. Manag.* **291**:117262. DOI:10.1016/j.enconman.2023.117262
29. Ministry of Housing and Urban-Rural Development of the People's Republic of China (MOHURD) (2021). China Urban-Rural Construction Statistical Yearbook
30. National Bureau of Statistics of China (NBSC) (2017). China Urban-Rural Construction Statistical Yearbook
31. Ma, S., Deng, N., Zhao, C. et al. (2024). Decreasing greenhouse gas emissions from the municipal solid waste sector in Chinese cities. *Environ. Sci. Technol.* **58**:11342–11351. DOI:10.1021/acs.est.4c00408
32. Ma, S., Zhou, C., Chi, C. et al. (2020). Estimating physical composition of municipal solid waste in China by applying artificial neural network method. *Environ. Sci. Technol.* **54**:9609–9617. DOI:10.1021/acs.est.0c01802
33. Liu, F., Xin, L., Tang, H. et al. (2023). Regionalized life-cycle monetization can support the transition to sustainable rural food waste management in China. *Nat. Food* **4**:797–809. DOI:10.1038/s43016-023-00842-6
34. Fang, W., Ding, Y., Geng, J. et al. (2023). High potential of coupling the source-separation and incineration promotion to reduce costs based on city-level cost-benefit analysis of municipal solid waste management strategies in China. *Resour. Conserv. Recycl.* **197**:107099. DOI:10.1016/j.resconrec.2023.107099
35. Mu, B., Yu, X., Shao, Y. et al. (2023). Complete recycling of polymers and dyes from polyester/cotton blended textiles via cost-effective and destruction-minimized dissolution, swelling, precipitation, and separation. *Resour. Conserv. Recycl.* **199**:107275. DOI:10.1016/j.resconrec.2023.107275
36. Lim, L.H., Tan, P., Chan, W.P. et al. (2023). A techno-economic assessment of the reutilisation of municipal solid waste incineration ash for CO2 capture from incineration flue gases by calcium looping. *Chem. Eng. J.* **464**:142567. DOI:10.1016/j.cej.2023.142567
37. Zhang, M., Wei, J., Li, H. et al. (2024). Comparing and optimizing municipal solid waste (MSW) management focused on air pollution reduction from MSW incineration in China. *Sci. Total Environ.* **907**:167952. DOI:10.1016/j.scitotenv.2023.167952
38. Bohm, K., Hatley, G.A., Robinson, B.H. et al. (2022). Black soldier fly-based bioconversion of biosolids creates high-value products with low heavy metal concentrations. *Resour. Conserv. Recycl.* **180**:106149. DOI:10.1016/j.resconrec.2022.106149
39. Xue, Y., Moreno, J.M., Li, C. et al. (2025). Growing community-based composting programs in China: Implementation and policy lessons from eight cases. *Resour. Conserv. Recycl.* **212**:107882. DOI:10.1016/j.resconrec.2024.107882
40. Chinese National Development and Reform Commission (CNDRC) (2012). Notice on improving the price policy for waste incineration power generation. https://www.gov.cn/zwgk/2012-04/10/content_2109921.htm
41. Chinese Ministry of Ecology and Environment (CMEE) (2023). Automatic monitoring data public platform for household waste incineration power plants. https://ljgk.envsc.cn
42. Wang, Y., Fang, M., Lou, Z. et al. (2024). Methane emissions from landfills differentially underestimated worldwide. *Nat. Sustain.* **7**:496–507. DOI:10.1038/s41893-024-01307-9
43. Zhao, Q., Tang, W., Han, M. et al. (2023). Estimation of reduced greenhouse gas emission from municipal solid waste incineration with electricity recovery in prefecture- and county-level cities of China. *Sci. Total Environ.* **875**:162654. DOI:10.1016/j.scitotenv.2023.162654
44. Wei, J., Li, H., Liu, J. et al. (2022). Curbing dioxin emissions from municipal solid waste incineration: China's action and global share. *J. Hazard. Mater.* **435**:129076. DOI:10.1016/j.jhazmat.2022.129076
45. Lu, M., Zhou, C., Wang, C. et al. (2024). Worldwide scaling of waste generation in urban systems. *Nat. Cities* **1**:126–135. DOI:10.1038/s44284-023-00021-5
46. Wang, Y. (2025). Not enough garbage to burn: The industrial transformation behind the phenomenon. *Science and Technology Daily*. https://www.stdaily.com/web/gdxw/2025-06/30/content_362879.html
47. Ministry of Ecology and Environment of the People's Republic of China (MEE) (2025). 2024 Report on the State of China's Ecological Environment. https://www.mee.gov.cn/hjzl/sthjzk/zghjzkgb/202506/P020250604527010717462.pdf
48. The State Council of the People's Republic of China (SCC) (2023). China aims to basically achieve full coverage of garbage sorting by 2025. https://www.gov.cn/yaowen/shipin/202305/content_6875839.html
49. Ministry of Housing and Urban-Rural Development & National Development and Reform Commission (MHURD & NDRC) (2022). The 14th Five-Year Plan for National Urban Infrastructure. *Development*. http://www.gov.cn/zhengce/zhengceku/2022-08/01/content_5703626.htm

The Innovation, Volume 7

Supplemental Information

The waste-environmental-economic nexus facilitates city-specific cost-effective transition for China's municipal solid waste treatment

Hao Li, Xiaolong Lu, Fang Liu, Zhe Li, Xianmei Liu, Bin Lu, Shijun Ma, Baojing Gu, Chuanbin Zhou, and Zhaohua Wang

Supplementary files

Supplementary Text 1 Forecasts of population (POP), per capita gross domestic product (PCGDP) and MSW generation from 2022 to 2050

Projection of POP and PCGDP. The POP and PCGDP data from 2022 to 2050 are the basis for forecasting MSW generation in Chinese cities. Based on the historical POP and PCGDP data covering the period from 1988 to 2021, the autoregressive integrated moving average (ARIMA) model is used to forecast POP and PCGDP from 2022 to 2050 for each Chinese city.

$$POP_t^k = a_1 \cdot POP_{t-1}^k + a_2 \cdot POP_{t-2}^k + \cdots + a_n \cdot POP_{t-n}^k + \varepsilon_t + b_1 \cdot \varepsilon_{t-1} + b_2 \cdot \varepsilon_{t-2} + \cdots + b_m \cdot \varepsilon_{t-m} \quad \text{(S1)}$$

$$\begin{aligned} PCGDP_t^k = {} & a_1 \cdot PCGDP_{t-1}^k + a_2 \cdot PCGDP_{t-2}^k + \cdots + a_n \cdot PCGDP_{t-n}^k \\ & + \varepsilon_t + b_1 \cdot \varepsilon_{t-1} + b_2 \cdot \varepsilon_{t-2} + \cdots + b_m \cdot \varepsilon_{t-m} \end{aligned} \quad \text{(S2)}$$

Where POP_t^k and $PCGDP_t^k$ are the population and per capita gross domestic product, respectively. ε_t is the random error. n and m are integers that are often referred to as autoregressive and moving average, respectively. k and t represent different cities and years, respectively.

Projection of MSW generation. The MSW generation is closely related to socioeconomic indicators, especially population (POP) and per capita gross domestic product (PCGDP). Thus, we use multiple linear regression model to forecast the quantity of MSW generation from 2022 to 2050 for the 292 China's cities with historical POP and PCGDP data, while the autoregressive integrated moving average (ARIMA) model is used for the other 60 cities.

$$Q_{\mathrm{MSW},t}^k = a_{\mathrm{POP}} \cdot POP_t^k + a_{\mathrm{PCGDP}} \cdot PCGDP_t^k + a_0 \quad \text{(S3)}$$

$$Q_{\mathrm{MSW},t}^k = a_1 \cdot Q_{\mathrm{MSW},t-1}^k + a_2 \cdot Q_{\mathrm{MSW},t-2}^k + \cdots + a_n \cdot Q_{\mathrm{MSW},t-n}^k + \varepsilon_t + b_1 \cdot \varepsilon_{t-1} + b_2 \cdot \varepsilon_{t-2} + \cdots + b_m \cdot \varepsilon_{t-m} \quad \text{(S4)}$$

Where $Q_{\mathrm{MSW},t}^k$ is the quantity of MSW generation. POP_t^k and $PCGDP_t^k$ are the population and per capita gross domestic product, respectively. a_{POP}, a_{PCGDP} and a_0 are the regression coefficients. ε_t is the random error. a_n and b_m are the coefficients. n and m are integers that are often referred to as autoregressive and moving average, respectively. k and t represent different cities and years, respectively.

Table 1 Data source and processing of MSW treatment amount

Item	Historical Data (2001–2021)	Future Projections (2022–2050)
Data Source	China Urban and Rural Construction Statistical Yearbook	Projections based on socio-economic indicators (POP, PCGDP)
Coverage	352 prefecture-level cities	352 prefecture-level cities
Prediction Methodology	Actual observed values	Multiple linear regression + ARIMA (for generation amount)<br>Logistic curve + proportional allocation

Item	Historical Data (2001–2021)	Future Projections (2022–2050)
		(for treatment structure)

Notes:historical data on municipal solid waste (MSW) generation across cities from 2000 to 2021 were sourced from the China Urban and Rural Construction Statistical Yearbook (MOHURD, 2021), the China City Statistical Yearbook (NBSC, 2018), as well as related datasets and methodologies from Ma et al. (2020, 2024).

Supplementary Text 2 Calculation for the quantities of MSW disposal and recycling from 2022 to 2050 in various scenarios.

In RR-ID, RR-HBC-ID, and RR-HBB-ID scenarios, recyclable materials in MSW are expected to be collected and recycled as much as possible.

$$Q^k_{\text{recycle},j,t} = \alpha_{\text{recycle},j} \times \rho^k_j \times Q^k_{\text{MSW},t} \tag{S5}$$

$$Q^k_{\text{MSW},t}{}' = Q^k_{\text{MSW},t} - \sum_j Q^k_{\text{recycle},j,t} \tag{S6}$$

Where $Q^k_{\text{recycle},j,t}$ is the quantity of collected recyclable materials. $Q^k_{\text{MSW},t}$ is the quantity of MSW generation. $\alpha_{\text{recycle},j}$ is the collection rate. ρ^k_j is the ratio of recyclable materials to the total quantity of MSW. $Q^k_{\text{MSW},t}{}'$ represents the updated quantity of MSW that needs disposal. j represents different recyclable materials.

The disposal of the remaining MSW is either incineration-dominant (ID), bioconversion disposal-dominant (BD), or still the same as that in 2021. In scenario BAU, the ratios of MSW incineration, landfill, and bioconversion disposal ($\xi^k_{\text{inciner},0}$, $\xi^k_{\text{landfill},0}$, and $\xi^k_{\text{bioche},0}$) are expected to remain consistent with the levels in 2021.

$$\begin{cases} Q^k_{\text{inciner},t} = \xi^k_{\text{inciner},0} \times Q^k_{\text{MSW},t} \\ Q^k_{\text{landfill},t} = \xi^k_{\text{landfill},0} \times Q^k_{\text{MSW},t} \\ Q^k_{\text{bioche},t} = \xi^k_{\text{bioche},0} \times Q^k_{\text{MSW},t} \end{cases} \tag{S7}$$

Where $Q^k_{\text{inciner},t}$, $Q^k_{\text{landfill},t}$, and $Q^k_{\text{bioche},t}$ are the quantity of MSW incineration, landfill, and bioconversion disposal, respectively.

With the incineration-dominant strategy, including ID and RR-ID scenarios, the incineration rate ($\alpha^k_{\text{inciner},t}$) is expected to gradually increase, as detailed in Supplementary Text 5, and nearly all remaining MSW will be combusted by 2050. Then, the ratios of landfill and bioconversion disposal ($\zeta^k_{\text{landfill},0}$ and $\zeta^k_{\text{bioche},0}$) in the remaining MSW are expected to remain consistent with the levels in 2021.

$$Q^k_{\text{inciner},t} = \alpha^k_{\text{inciner},t} \times Q^k_{\text{MSW},t} \tag{S8}$$

$$Q^k_{\text{MSW},t}{}' = Q^k_{\text{MSW},t} - Q^k_{\text{inciner},t} \tag{S9}$$

$$\begin{cases} Q^k_{\text{bioche},t} = \zeta^k_{\text{bioche},0} \times Q^k_{\text{MSW},t} \\ Q^k_{\text{landfill},t} = \zeta^k_{\text{landfill},0} \times Q^k_{\text{MSW},t} \end{cases} \tag{S10}$$

With the bioconversion disposal-dominant strategy, including LBC, LBB, HBC, HBB, HBC-ID, HBB-ID, RR-HBC-ID, and RR-HBB-ID scenarios, the organic

components in MSW will be prioritized for bioconversion disposal.

$$Q^k_{\text{bioche},t} = \alpha^k_{\text{bioche},t} \times \rho^k_{\text{organic}} \times Q^k_{\text{MSW},t} \tag{S11}$$

$$Q^k_{\text{MSW},t}{}' = Q^k_{\text{MSW},t} - Q^k_{\text{bioche},t} \tag{S12}$$

Where ρ^k_{organic} is the ratio of organic components to the total quantity of MSW. $\alpha^k_{\text{bioche},t}$ is the bioconversion disposal rate of organic components. However, the rate of this disposal will be constrained by the advancement of bioconversion disposal technologies in different cities. In LBC and LBB scenarios, the bioconversion disposal rates of organic components in less-developed and developed cities are expected to gradually increase to 30% and 50%, respectively. In contrast, in HBC, HBB, HBC-ID, HBB-ID, RR-HBC-ID, and RR-HBB-ID scenarios, these rates are expected to reach 50% and 100%. The classification of cities as less-developed or developed is based on whether the rate of this disposal there was lower or higher than 1% in 2021.

After the bioconversion disposal of certain organic components, the incineration rate of the remaining MSW is expected to gradually increase in HBC-ID, HBB-ID, RR-HBC-ID, and RR-HBB-ID scenarios, as detailed in equation (S8)-(S9). Then, the final remaining MSW will be landfilled.

$$Q^k_{\text{landfill},t} = Q^k_{\text{MSW},t} \tag{S13}$$

In contrast, in BC, LBB, HBC, and HBB scenarios, the ratios of incineration and landfill ($\psi^k_{\text{landfill},0}$ and $\psi^k_{\text{bioche},0}$) in the remaining MSW are expected to remain consistent with the levels in 2021 after the bioconversion disposal of certain organic components.

$$\begin{cases} Q^k_{\text{inciner},t} = \psi^k_{\text{inciner},0} \times Q^k_{\text{MSW},t} \\ Q^k_{\text{landfill},t} = \psi^k_{\text{landfill},0} \times Q^k_{\text{MSW},t} \end{cases} \tag{S14}$$

Moreover, in LBC, HBC, HBC-ID, and RR-HBC-ID scenarios, traditional compost is utilized for the bioconversion disposal of organic components. In contrast, bioconversion is utilized for this disposal in LBB, HBB, HBB-ID, and RR-HBB-ID scenarios.

Supplementary Text 3 Clarification on MSW incineration, landfill and Bioconversion disposal

MSW Incineration: Incineration not only minimizes the physical space required for MSW disposal but also harnesses energy that can be utilized for electricity generation, receiving a strong endorsement from Chinese government. Nevertheless, a significant amount of fly ash, which contains heavy metals, soluble salts, and dioxins, is produced during the incineration process, posing environmental toxicity concerns [1]. There are two primary technologies for MSW incineration: fluidized bed incineration and grate firing incineration. The calculation of operational benefits for MSW incineration considers these two technologies, whose respective ratios are 7.8% and 92.2%, according to data released by the Ministry of Ecology and Environment of China in June 2023 [2]. The converted grid-connected electricity output per ton of MSW is 280 kWh, and a nationwide uniform benchmark feed-in tariff with subsidies contained is implemented, set at 0.65 CNY/kWh [3], according to the price policy for

waste incineration power generation established by the Chinese National Development and Reform Commission. Moreover, we compiled the gate fees for waste incineration projects across various provinces in China, which ranged from 59.65 to 247.24 CNY/ton.

MSW landfill: Landfill is one of the most common methods for MSW disposal worldwide. While it is cost-effective, it poses risks related to greenhouse gas emissions and environmental pollution [4]. Once landfilled, organic components, as well as paper, textiles, and wood and bamboo in MSW, will gradually degrade and produce landfill gas, which contains a significant amount of methane. Therefore, landfill gas is also an energy resource and can provide benefits [5]. Moreover, we compiled the gate fees for waste landfill projects across various provinces in China, which ranged from 15 to 110 CNY/ton.

MSW bioconversion disposal: bioconversion disposal aims to convert organic components in MSW into valuable by-products, demonstrating better environmental performance compared to incineration and landfill. One of the predominant methods is compost. During the composting phase, the organic components are decomposed under aerobic conditions by microorganisms, resulting in the production of compost. This process promotes a circular economy by recycling organic components in MSW back into the ecosystem [6]. On the other hand, bioconversion has emerged as an innovative and sustainable approach for organic waste management. This process involves the utilization of insects to convert various types of organic waste into high-value protein and biomass, creating new revenue streams for organic waste bioconversion disposal[7]. Moreover, we compiled data on the unit subsidy costs for MSW bioconversion disposal projects from various cities, including Beijing, Chongqing, Shenzhen, Changsha, Harbin, Hohhot, Jinan, Qingdao, Zaozhuang, and Xianyang. These cities represent diverse geographical locations and economic development levels. Subsequently, based on three key indicators (population, GDP per capita, and the quantity of organic components in per capita MSW), we assigned the benchmark subsidy values from these representative cities to other cities with similar socio-economic and waste characteristics. The resulting subsidies for MSW bioconversion disposal across different cities range from 168 to 338.6 CNY/ton.

Supplementary Text 4 Calculation for the monetization factors of LCA indicators

The calculation equations on the monetization factors of LCA indicators are presented below, and the specific explanations refer to [8].

PMFP, FEP, MEP, EOFP, HOFP and TAP:

$$\upsilon_{d,l}^{k} = \frac{1}{q_{d,l}^{k}} \times r_{d,l}^{k} \quad \text{(S15)}$$

$$\delta_{d,l}^{k} = \frac{s_{d,l}^{k} \times h_{d,l}^{k}}{\sum\left(s_{d,l}^{k} \times h_{d,l}^{k}\right)} \quad \text{(S16)}$$

$$\omega_{l}^{k} = \sum\left(\delta_{d,l}^{k} \times \upsilon_{d,l}^{k}\right) \quad \text{(S17)}$$

Where $\upsilon_{d,l}^{k}$ is the monetary intermediate factor. $q_{d,l}^{k}$ is the pollutant equivalent

value. $r_{d,l}^{k}$ is the tax on emissions. $\delta_{d,l}^{k}$ is the influence potential coefficient. $\mathrm{s}_{d,l}^{k}$ is the characterization factor. $h_{d,l}^{k}$ is the total emissions amount in 2020. l represents different indicators. d represents the pollutants related to the indicators.

FETP and METP:

$$\omega_{\mathrm{FETP}}^{k}=\frac{r_{\mathrm{FETP}}^{k}}{q_{\mathrm{FETP}}^{k}} \tag{S18}$$

$$\omega_{\mathrm{METP}}^{k}=\omega_{\mathrm{FETP}}^{k}\times\frac{\mathrm{SD_{marine}}}{\mathrm{SD_{fresh}}}\times\frac{\mathrm{CF_{marine}}}{\mathrm{CF_{fresh}}} \tag{S19}$$

Where $\mathrm{SD_{marine}}$ is the species density in marine water. $\mathrm{SD_{fresh}}$ is the species density in fresh water. $\mathrm{CF_{marine}}$ is the conversion factor from midpoint to endpoint for METP. $\mathrm{CF_{fresh}}$ is the conversion factor from midpoint to endpoint for FETP.

FFP and SOP:

$$\omega_{\mathrm{SOP}}^{k}=\overline{\mathrm{p}}_{\mathrm{Cu,2020}}\times\mathrm{Tax}_{\mathrm{Cu}}^{k} \tag{S20}$$

$$\omega_{\mathrm{FFP}}^{k}=\overline{\mathrm{p}}_{\mathrm{Oil,2011\text{-}2020}}\times\mathrm{Tax}_{\mathrm{Oil}}^{k} \tag{S21}$$

Where $\overline{\mathrm{p}}_{\mathrm{Cu,2020}}$ and $\overline{\mathrm{p}}_{\mathrm{Oil,2011\text{-}2020}}$ are the average prices of copper and oil, respectively. $\mathrm{Tax}_{\mathrm{Cu}}^{k}$ and $\mathrm{Tax}_{\mathrm{Oil}}^{k}$ are the copper tax on mineral processing and oil tax, respectively.

LU:

$$\mathrm{MSA}_{\mathrm{f}\to\mathrm{c}}=\frac{\left(S_{\mathrm{f}}-S_{\mathrm{c}}\right)}{S_{\mathrm{f}}} \tag{S22}$$

$$\mathrm{MSA}_{\mathrm{g}\to\mathrm{c}}=\frac{\left(S_{\mathrm{g}}-S_{\mathrm{c}}\right)}{S_{\mathrm{g}}} \tag{S23}$$

$$\mathrm{MFR_{CPI}}=\frac{\mathrm{CPI_{EU,2021}}}{\mathrm{CPI_{EU,2020}}} \tag{S24}$$

$$\omega'_{\mathrm{LU,f,2020}}=\rho_{\mathrm{f}}\times\mathrm{MSA}_{\mathrm{f}\to\mathrm{c}}\times\frac{\omega'_{\mathrm{LU,f,2021}}}{10{,}000}\times\mathrm{MFR_{CPI}} \tag{S25}$$

$$\omega'_{\mathrm{LU,g,2020}}=\rho_{\mathrm{g}}\times\mathrm{MSA}_{\mathrm{g}\to\mathrm{c}}\times\frac{\omega'_{\mathrm{LU,f,2021}}}{10{,}000}\times\mathrm{MFR_{CPI}} \tag{S26}$$

$$\omega'_{\mathrm{LT,f,2020}}=0.5\times\rho_{\mathrm{f}}\times\mathrm{MSA}_{\mathrm{f}\to\mathrm{c}}\times\frac{\omega'_{\mathrm{LT,f,2021}}}{10{,}000}\times\frac{1}{\mathrm{N_{rel,f}}}\times\mathrm{MFR_{CPI}} \tag{S27}$$

$$\omega'_{\mathrm{LT,g,2020}}=0.5\times\rho_{\mathrm{g}}\times\mathrm{MSA}_{\mathrm{g}\to\mathrm{c}}\times\frac{\omega'_{\mathrm{LT,g,2021}}}{10{,}000}\times\frac{1}{\mathrm{N_{rel,g}}}\times\mathrm{MFR_{CPI}} \tag{S28}$$

$$\mathrm{MFR_{PPP}}=\frac{\mathrm{PPP_{China,2020}}}{\mathrm{PPP_{EU,2020}}} \tag{S29}$$

$$\omega_{\mathrm{LU,2020}}^{k}=\left(\sum\omega'_{\mathrm{LT,f,2020}}+\sum\omega_{\mathrm{LT,2020}}^{k}\right)\times\mathrm{MFR_{PPP}}\times\mathrm{MFR}_{\mathrm{lut}}^{m} \tag{S30}$$

Where $\mathrm{MSA}_{\mathrm{f}\to\mathrm{c}}$ is the mean species abundance when forest is transformed to cropland. S_{f}, S_{c}, S_{f} and are the relative species richness of forest, grassland, and annual cropland, respectively. $\mathrm{MSA}_{\mathrm{g}\to\mathrm{c}}$ is the mean species abundance when grassland is transformed to cropland. $\mathrm{CPI}_{U,t}$ is the consumer price index. $\mathrm{MFR}_{\mathrm{CPI}}$ is the modification factor of consumer price index. $\omega'_{\mathrm{LU,f},t}$ and $\omega'_{\mathrm{LU,g},t}$ are the average monetization factor of LU in China. ρ_{f} and ρ_{g} are the proportions of forest biomes grassland biomes to the total global terrestrial area, respectively. $\omega'_{\mathrm{LT,f},t}$ and $\omega'_{\mathrm{LT,g},t}$ are the monetization factor of land transformation. $\mathrm{N}_{\mathrm{rel,f}}$ and $\mathrm{N}_{\mathrm{rel,g}}$ are the recovery time (years) for species richness of forest biomes and grassland biomes, respectively. $\mathrm{PPP}_{U,t}$ is the purchasing power parity. $\mathrm{MFR}_{\mathrm{PPP}}$ is the modification factor of purchasing power parity. $\mathrm{MFR}^{m}_{\mathrm{lut}}$ is the modification factor of land use tax. U represents different countries.

GWP:

$$\bar{\omega}_{\mathrm{GWP}} = \frac{\sum_{s=1}^{5}\left(\mathrm{AC}_s \times \mathrm{AP}_s\right)}{\sum_{s=1}^{5}\mathrm{AP}_s} \tag{S31}$$

Where $\mathrm{AC}_{\mathrm{sec}}$ is the abated amount of CO_2. $\mathrm{AP}_{\mathrm{sec}}$ is the CO_2 abatement cost. s represents different sectors including five major sectors—construction, cement, power, the iron and steel industry, and the petrochemical industry.

ODP:

$$\chi^{k}_{\mathrm{ODP}} = \frac{\mathrm{Income}^{k}}{\overline{\mathrm{Income}}} \tag{S32}$$

$$\omega_{\mathrm{ODP,2006}} = \frac{\mathrm{Invest}_{\mathrm{total}}}{\mathrm{ODS}_{\mathrm{total}}} \tag{S33}$$

$$\mathrm{MF}^{k}_{\mathrm{ozone}} = \frac{\mathrm{OC}^{k}_{\mathrm{ozone}}}{\overline{\mathrm{OC}}_{\mathrm{ozone}}} \tag{S34}$$

$$\omega^{k}_{\mathrm{ODP,2020}} = \omega_{\mathrm{ODP,2006}} \times \chi^{k}_{\mathrm{ODP}} \times \mathrm{MF}^{k}_{\mathrm{ozone}} \times \frac{\mathrm{CPI}_{2020}}{\mathrm{CPI}_{2006}} \tag{S35}$$

Where χ^{k}_{ODP} is the economic modifying factor. Income^{k} is the per capita income. $\overline{\mathrm{Income}}$ is the average per capita income in China. $\mathrm{Invest}_{\mathrm{total}}$ is the total investment in ozone-depleting substances abatement. $\mathrm{ODS}_{\mathrm{total}}$ is the total reduction amount of ozone-depleting substances. $\mathrm{MF}^{k}_{\mathrm{ozone}}$ is the modification factor of ODP. $\mathrm{OC}^{k}_{\mathrm{ozone}}$ is the ozone concentration. $\overline{\mathrm{OC}}_{\mathrm{ozone}}$ is the average ozone concentration in China.

$\mathbf{HT_c}$ and $\mathbf{HT_{nc}}$:

$$V_{2020} = \frac{\mathrm{CPI}_{2020}}{\mathrm{CPI}_{2018}} \times V_{2018} \tag{S36}$$

$$\omega^{k}_{\mathrm{HT}} = \sum V_{2020} \times \mathrm{CF}_{(\mathrm{non})\mathrm{cancer}} \times \chi^{k}_{\mathrm{HT}} \tag{S37}$$

Where V_t is the value of life expectancy of Chinese residents. $\mathrm{CF}_{(\mathrm{non})\mathrm{cancer}}$ is the conversion factor.

TETP:

$$\text{Intense}^k = \frac{\text{Consume}_{\text{ph}}^k}{\text{Area}_{\text{sown}}^k} \times \frac{\text{M}_{\text{ph}}^k \times 2}{\text{M}_{\text{P}_2\text{O}_5}^k} \tag{S38}$$

$$\text{MFR}_{\text{intensity}}^k = \frac{\text{Intense}^k}{\overline{\text{Intense}}} \tag{S39}$$

$$\omega_{\text{TETP}}{}' = \omega_{\text{TETP,Stepwise}} \times \frac{\text{CPI}_{\text{EU,2019}}}{\text{CPI}_{\text{EU,2020}}} \times \frac{\text{PPP}_{\text{China,2020}}}{\text{PPP}_{\text{EU,2020}}} \tag{S40}$$

$$\omega_{\text{TETP}}^k = \sum \omega_{\text{TETP}}{}' \times \text{MFR}_{\text{intensity}}^k \times \chi_{\text{TETP}}^k \tag{S41}$$

Where Intense^k is the application intensity of phosphorus. $\text{Consume}_{\text{ph}}^k$ is the consumption amount of phosphate. $\text{Area}_{\text{sown}}^k$ is the total sown area of crops. M_{ph}^k and $\text{M}_{\text{P}_2\text{O}_5}^k$ are the molecular weight of phosphorus and phosphorus pentoxide, respectively. $\text{MFR}_{\text{intensity}}^k$ is the modification factor of fertilization intensity $\overline{\text{Intense}}$ is the average application intensity of phosphorus in China. $\omega_{\text{TETP}}{}'$ is the average monetization factor for TETP in China. $\omega_{\text{TETP,Stepwise}}$ is the monetization factor of TETP for the Stepwise model in 2019.

IRP:

$$\text{MFR}^k = \frac{\text{DR}^k}{\overline{\text{DR}}} \tag{S42}$$

$$\omega_{\text{IR}}^k = \text{IR}_{\text{Ecotax}} \times \frac{\text{CPI}_{\text{EU,2020}}}{\text{CPI}_{\text{EU,2019}}} \times \frac{\text{PPP}_{\text{China,2020}}}{\text{PPP}_{\text{EU,2020}}} \times \text{MFR}^k \times \chi_{\text{IR}}^k \tag{S43}$$

Where DR^k is the air absorption dose rate of γ-rays. $\overline{\text{DR}}$ is the average air absorption dose rate of γ-rays in China. $\text{IR}_{\text{Ecotax}}$ is the average monetization factor of IR in Ecotax 2006 in Switzerland.

Supplementary Text 5 Increase trend in the incineration rates from 2022 to 2050 in ID, HBC-ID, HBB-ID, RR-ID, RR-HBC-ID, and RR-HBB-ID scenarios

The quantity of MSW incineration in each city follows the logistic curve and grows close to the quantity of MSW generation over time. Notably, as of 2021, there remain 93 cities in China with an incineration rate of 0, with nearly 70% located in the Northwest China, Southwest China and Central China. It is assumed that the incineration rates in these cities will reach the average level of the provinces in which they are located in the future.

$$Q_{\text{inciner},t}^k = \frac{\exp(\beta_0 + \beta_1 \cdot T)}{1 + \exp(\beta_0 + \beta_1 \cdot T)} \times Q_{\text{MSW},t}^k \tag{S44}$$

Where β_0 and β_1 are the constants. T denotes the year.

Supplementary Text 6 Review on waste management policies in China

Waste management is not only crucial for environmental protection and urban sanitation but also an essential component of the circular economy. Through proper waste sorting and treatment, resource waste can be effectively reduced, enabling resource regeneration and recycling, contributing to building a resource-saving and environmentally friendly society, and promoting harmonious economic and

environmental development.

China's waste management policies have evolved from initial response to systematic promotion and comprehensive deepening, gradually achieving a development path from disposal to waste sorting and refined management, as detailed in Supplementary Table 12. Based on this process, China's waste management policies show three main trends:

(1) Disposal methods have shifted from primarily sanitary landfills to incineration, encouraging the development of waste-to-energy incineration.

Waste incineration is a crucial technological choice for waste resource utilization and greenhouse gas reduction. Since the 12th Five-Year Plan, the National Development and Reform Commission has clearly set development targets for waste incineration plants and related treatment capacity in national urban waste treatment planning, promoting the rapid development of China's waste incineration industry. China's municipal waste incineration volume has maintained rapid growth over the past decade, with incineration treatment volume exceeding landfill volume for the first time in 2019 in terms of proportion of total harmless treatment. The volume increased from 38.76 million tons in 2012 to 232 million tons in 2022, with the proportion of urban waste incineration in harmless treatment reaching 75% by 2022, achieving the 14th Five-Year Plan target.

On the other hand, in August 2023, the National Development and Reform Commission, Ministry of Finance, and National Energy Administration issued the Notice on Implementing Comprehensive Green Power Certificate Coverage to Promote Renewable Energy Power Consumption, which proposed improving green certificate trading and achieving full coverage of renewable energy power through green certificates. This marks the beginning of waste incineration green certificate trading. Through green certificate trading, waste-to-energy enterprises can obtain additional revenue streams, thereby improving their economic benefits. This incentive mechanism can attract more enterprises to participate in waste incineration treatment, alleviate financial pressure in waste treatment, and accelerate the construction and operation of waste incineration facilities.

(2) Waste sorting has been gradually implemented from pilot areas to wider coverage.

After rapid growth during the 13th Five-Year Plan period, the solid waste industry needs efficient, high-quality development, with waste sorting being a key approach to improving waste resource utilization efficiency. In addition to national-level policies, provinces and cities have also successively issued relevant policies to promote the development of municipal waste treatment industry. Currently, 21 provinces (autonomous regions) and 173 cities have issued local regulations and government rules on waste sorting. Forty-six key cities have taken the lead in establishing relatively complete waste sorting systems for collection, transportation, and disposal, as detailed in Supplementary Table 13. The coverage rate of waste sorting in residential communities in cities at prefecture level and above has reached 92.6% [9].

(3) Household food waste management has become stricter, with increased focus on resource utilization.

Household food waste refers to easily perishable organic waste discarded in daily household life, such as fruits and vegetables, food trimmings, leftover food, and fruit peels [10]. It is a major component of household waste in China. Household food waste is characterized by high water content, high organic content, and easy degradability [11]. During urban waste collection and treatment, it can easily contaminate other components, hindering the recovery of valuable materials. Therefore, separate treatment of Household food waste is crucial for reducing mixed waste contamination and improving resource recovery rates.

In 2010, the National Development and Reform Commission and other departments launched 100 pilot cities to promote Household food waste resource utilization and harmless treatment. Since 2019, as various regions accelerated mandatory waste sorting, Shanghai, Beijing, Guangdong, and Fujian have successively issued detailed regulations on Household food waste management. These regulations specify requirements for waste reduction, unified collection and disposal, supervision, and resource utilization. Compared to other types of waste, Household food waste collection has higher standards and stricter inspection requirements to ensure safe, efficient, environmentally friendly treatment and resource utilization.

In conclusion, comprehensive management and high-quality development of urban municipal waste has become a new development trend in China. With accelerating urbanization and improving living standards, urban waste output continues to increase. This poses higher requirements for waste treatment, compelling national and local governments to adopt more efficient and environmentally friendly treatment measures. First, waste sorting has become a crucial part of urban management. Through source separation and refined management, it effectively reduces the difficulty and cost of waste treatment. Second, various regions are actively introducing advanced waste treatment technologies, such as waste-to-energy incineration and kitchen waste resource utilization, improving waste treatment efficiency and resource utilization rates. Furthermore, continuous improvement of policies and regulations, along with rising public environmental awareness, has promoted the establishment and optimization of comprehensive waste management systems. Looking ahead, the goal of high-quality development includes not only achieving harmless waste treatment but also maximizing resource utilization and sustainable environmental protection. Optimizing waste treatment structure and improving municipal waste resource utilization levels will become key industry directions.

Supplementary Text 7 Definition of Environmental Impact Indicators

Impact Category	Indicator Name (Full Name & Abbreviation)	Definition	Unit
Climate Change	Global Warming Potential (GWP)	Measures the contribution of greenhouse gas emissions to global warming over a specified time horizon.	kg CO_2 eq.

Impact Category	Indicator Name (Full Name & Abbreviation)	Definition	Unit
Ozone Depletion	Ozone Depletion Potential (ODP)	Quantifies the potential of emissions to destroy the stratospheric ozone layer.	kg CFC-11 eq.
Air Quality (Human Health)	Particulate Matter Formation Potential (PMFP)	Measures the potential of emissions to form particulate matter (PM2.5), which affects human respiratory health.	kg PM2.5 eq.
Air Quality (Ecosystems)	Photochemical Oxidant Formation Potential - Ecosystems (EOFP)	Indicates the potential of emissions to form ground-level ozone (smog), harming ecosystem health.	kg NOx eq.
Air Quality (Human Health)	Photochemical Oxidant Formation Potential - Human Health (HOFP)	Indicates the potential of emissions to form ground-level ozone, affecting human health.	kg NOx eq.
Radiation	Ionizing Radiation Potential (IRP)	Measures the impact of emissions that release ionizing radiation on human health and the environment.	kBq Co-60 eq.
Water Eutrophication	Freshwater Eutrophication Potential (FEP)	Assesses the potential of nutrient emissions to cause over-enrichment and oxygen depletion in freshwater bodies.	kg P eq.
Water Eutrophication	Marine Eutrophication Potential (MEP)	Assesses the potential of nutrient emissions to cause over-enrichment in marine ecosystems.	kg P eq.
Ecotoxicity	Freshwater Ecotoxicity Potential (FETP)	Evaluates the long-term potential harm of toxic substances on freshwater ecosystems.	kg 1,4-DCB eq.
Ecotoxicity	Marine Ecotoxicity Potential (METP)	Evaluates the long-term potential harm of toxic substances on marine ecosystems.	kg 1,4-DCB eq.
Resource Consumption	Water Consumption Potential (WCP)	Quantifies the impact of freshwater consumption on water resource availability.	m^3 water eq.
Terrestrial Acidification	Terrestrial Acidification Potential (TAP)	Measures the potential of emissions to acidify soils and harm terrestrial ecosystems.	kg SO_2 eq.
Ecotoxicity	Terrestrial Ecotoxicity Potential (TETP)	Evaluates the long-term potential harm of toxic substances on soil organisms and terrestrial ecosystems.	kg 1,4-DCB eq.

Impact Category	Indicator Name (Full Name & Abbreviation)	Definition	Unit
Land Use	Land Use (LU)	Assesses the impact of occupying or transforming land for activities like waste disposal facilities.	m^2a crop eq.
Human Toxicity	Human Toxicity Potential - cancer (HTc)	Estimates the potential risk of exposure to toxic emissions causing carcinogenic effects.	kg 1,4-DCB eq.
Human Toxicity	Human Toxicity Potential - non-cancer (HTnc)	Estimates the potential risk of exposure to toxic emissions causing non-carcinogenic health effects.	kg 1,4-DCB eq.
Resource Scarcity	Fossil Resource Scarcity Potential (FFP)	An indicator of the depletion of abiotic fossil resources (e.g., oil, coal).	kg oil eq.
Resource Scarcity	Mineral Resource Scarcity Potential (SOP)	An indicator of the depletion of abiotic mineral resources (e.g., copper, iron ores)	

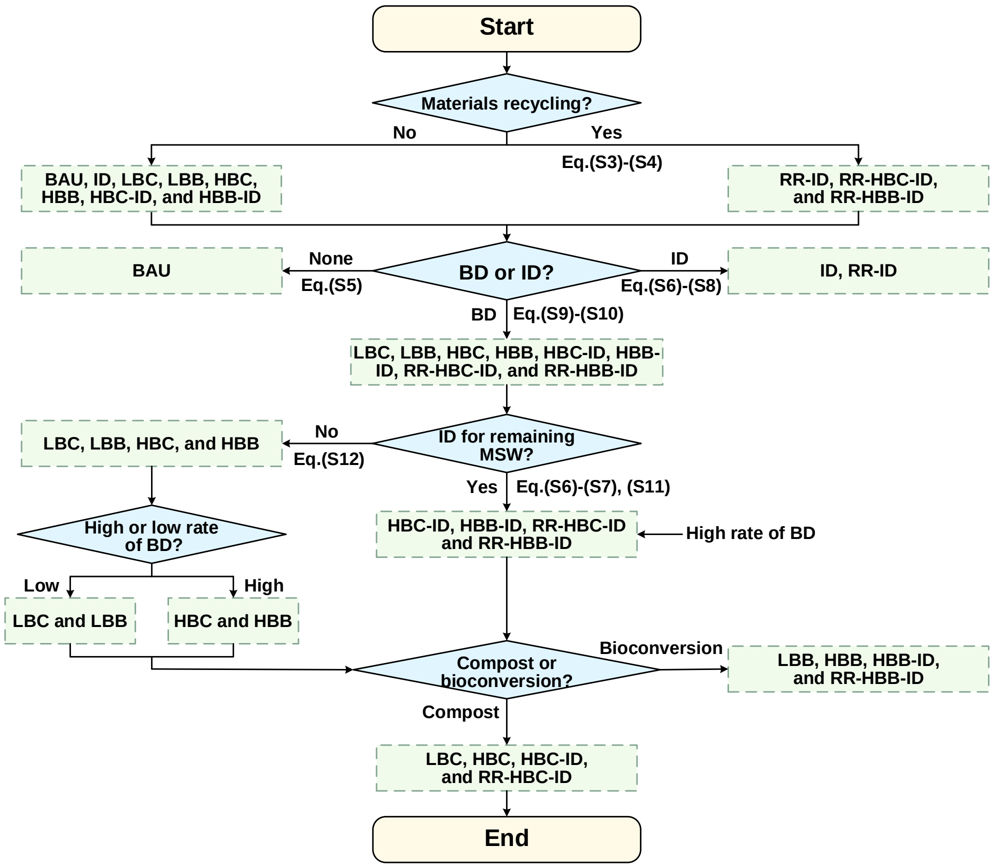

Supplementary Fig. 1 Logic diagram for the calculation of the MSW disposal structures in various scenarios. In order to promote the high value utilization of MSW, the priority for bioconversion disposal of organic components and recycling of recyclable materials will be higher than incineration and landfill in all scenarios. Therefore, in RR-ID, RR-HBC-ID, and RR-HBB-ID scenarios, a fixed ratio of recyclable materials in MSW, including paper, plastics, glass, textiles, and metals, will be recycled before incineration and landfill. And in LBC, LBB, HBC, HBB, HBC-ID, HBB-ID, RR-HBC-ID, and RR-HBB-ID scenarios, an increasing rate of organic components in MSW will be biochemically disposed before incineration and landfill. Excluding the above two measures, if the incineration-dominated MSW disposal mode is adopted, most of the remaining MSW will be combusted by 2050 with an increasing incineration rate. Otherwise, the remaining MSW will be combusted and landfilled according to the ratios of the two MSW disposal methods in 2021. ID: incineration-dominated; BD: bioconversion disposal-dominated.

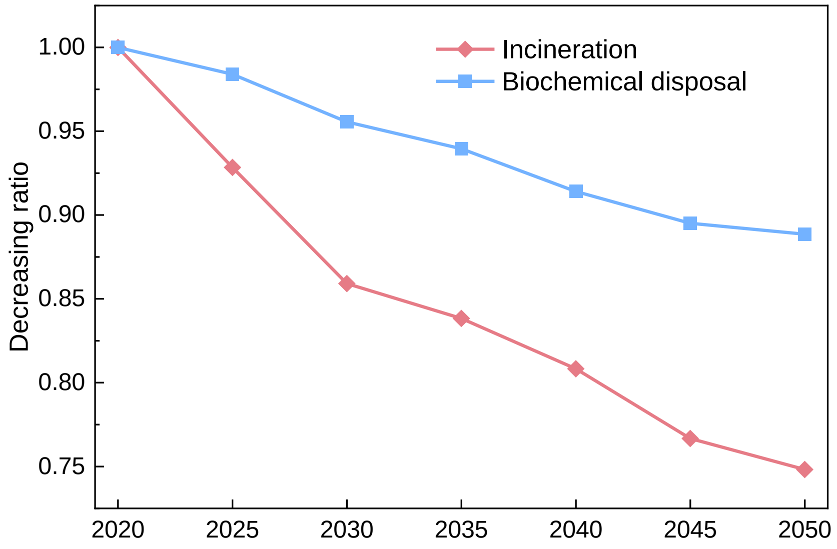

Supplementary Fig. 2 Decreasing ratios of the investment costs for MSW incineration and bioconversion disposal. It reflects the advancements in MSW incineration and bioconversion disposal technologies, resulting in a reduction of investment costs, as well as operation and management costs for these two methods of MSW disposal whose initial values in 2022 refer to [12]. The declining trends are calculated respectively based on the learning curves for gas turbine technology and bioconversion disposal technology, as presented in [13].

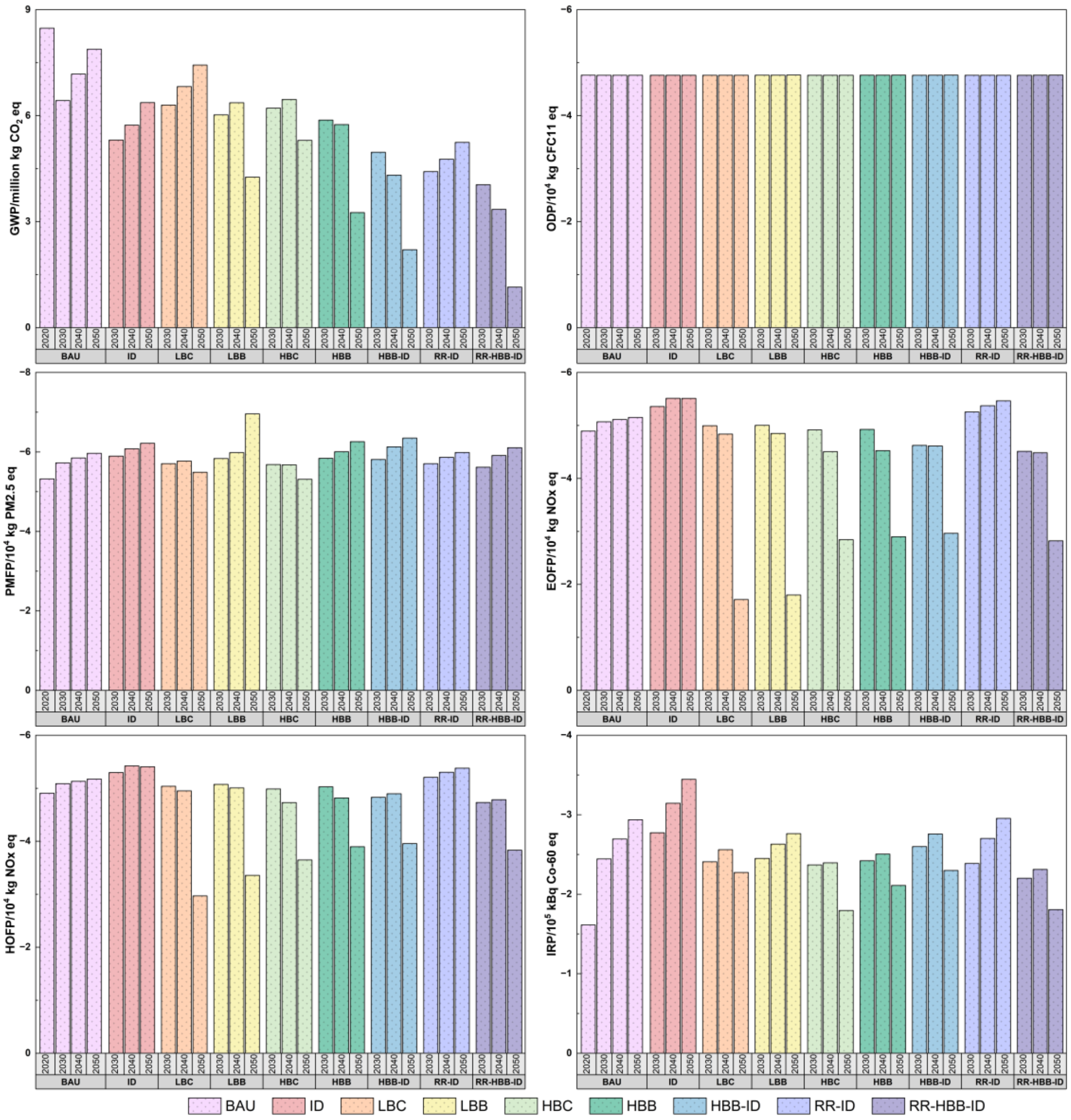

(a) Air-related impacts of MSW treatment under different scenarios

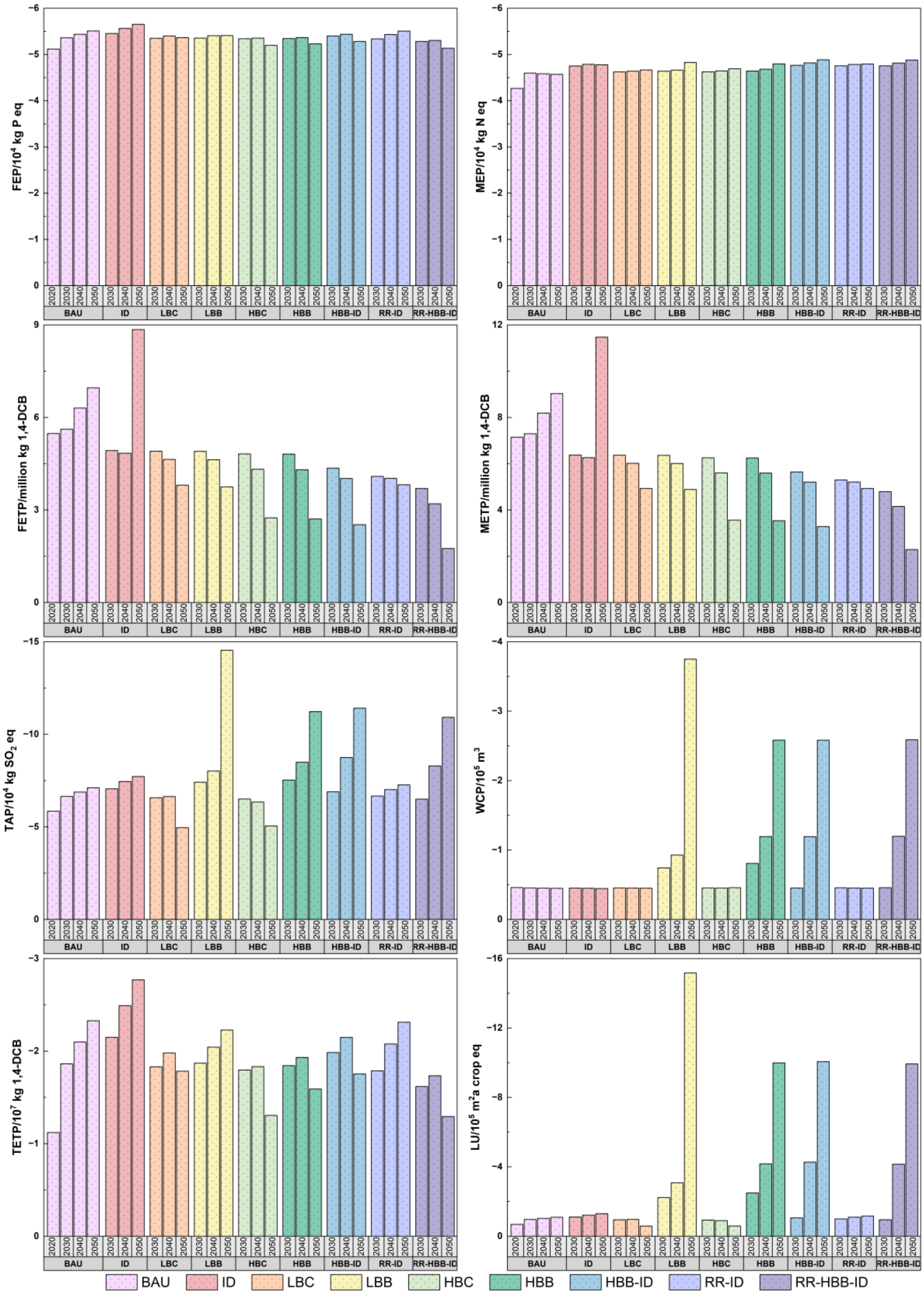

(b) Water-related and soil-related impacts of MSW treatment under different scenarios

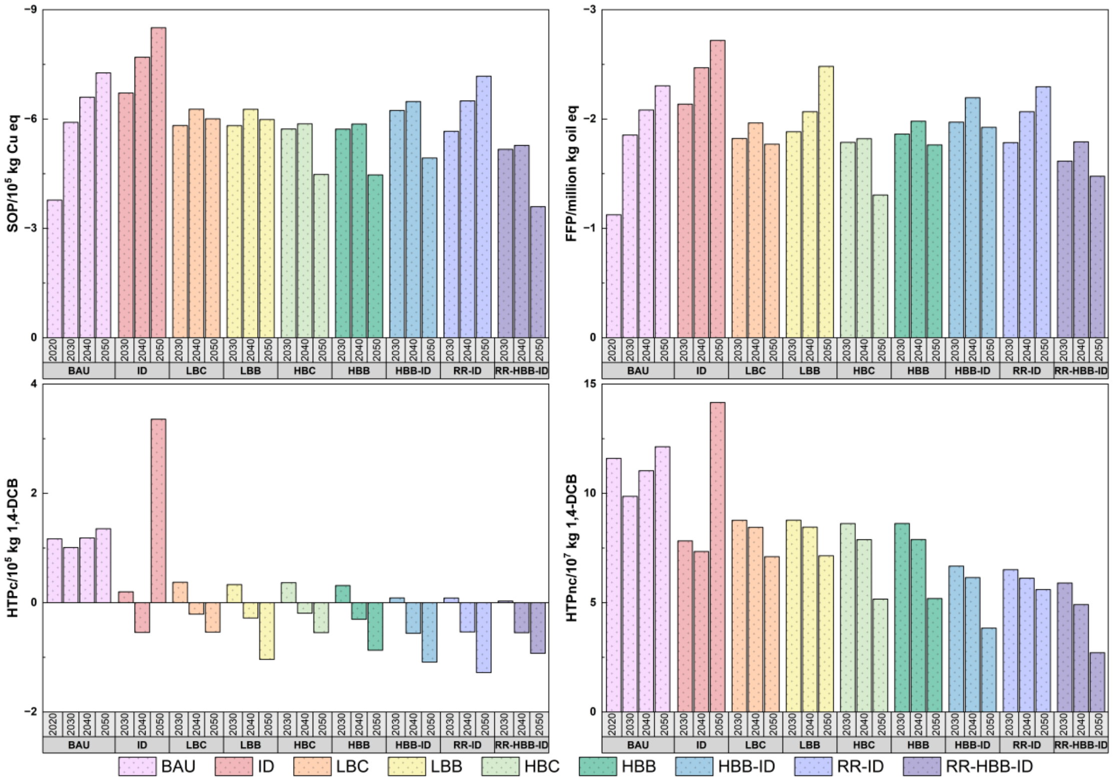

(c) Resource-depletion-related and human-related environmental impacts of MSW treatment under different scenarios

Supplementary Fig. 3 Environmental impacts of MSW treatment under different scenarios. (a)-(c) display the air-related impacts, water-related and soil-related impacts, and resource-depletion-related and human-related environmental impacts, respectively in 2020, 2030, 2040 and 2050 under nine different scenarios. These scenarios are as follows: BAU (Business-as-usual) maintains the same ratio of each disposal method as in 2021. ID (Incineration-dominated) features a gradual increase in the incineration rate until nearly all MSW is combusted by 2050. LBB (Low-rate bioconversion for food waste) and LBC (Low-rate composting for food waste) involve a low proportion of organic components biochemically disposed with compost and bioconversion, respectively, while the remaining waste are disposed in line with BAU scenario. HBC (High-rate composting for food waste) and HBB (High-rate bioconversion for food waste) entail a high proportion of organic components biochemically disposed with compost and bioconversion, respectively while the remaining waste are disposed in line with BAU scenario. HBB-ID (High-rate bioconversion for organic components + Incineration-dominated for the remaining) combines a high proportion of organic components biochemically disposed with the remaining MSW incinerated. RR-ID (Recycling + Incineration-dominated) incorporates the recycling of recyclable materials along with the remaining MSW incinerated. and RR-HBB-ID (Recycling + High-rate bioconversion + Incineration-dominated) integrates recycling, high-proportion bioconversion of organic components, and ID-based disposal of the remaining MSW.

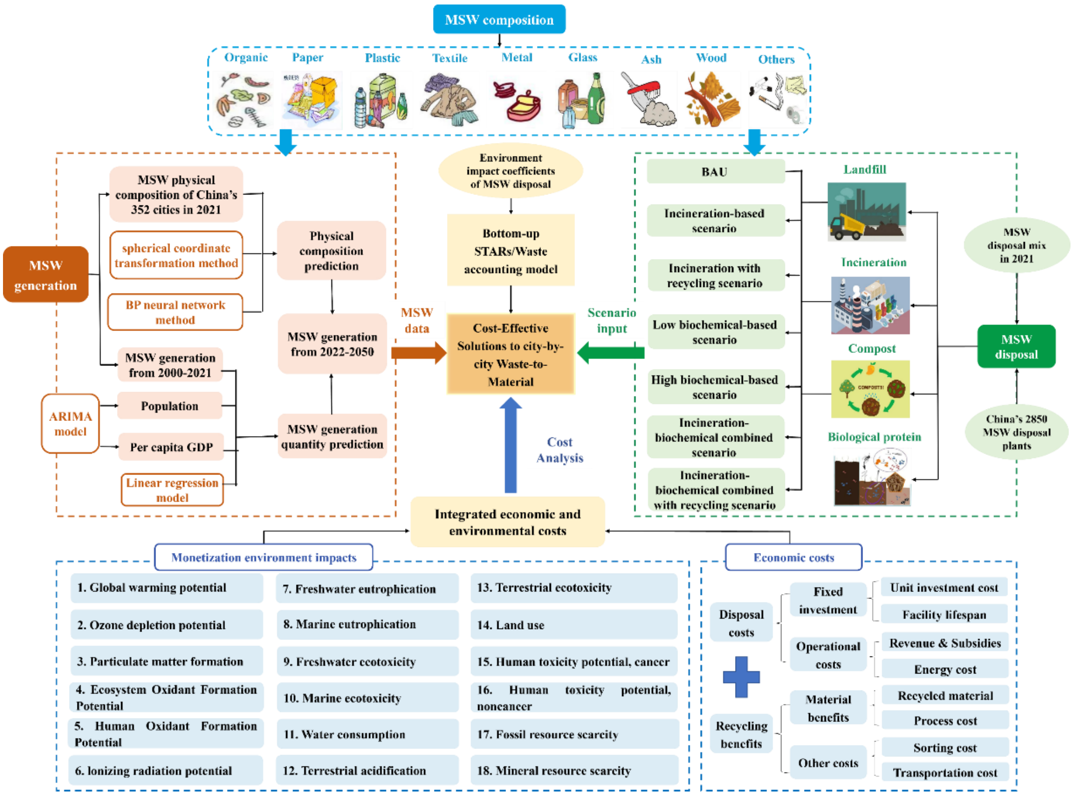

Supplementary Fig. 4 Framework of this study

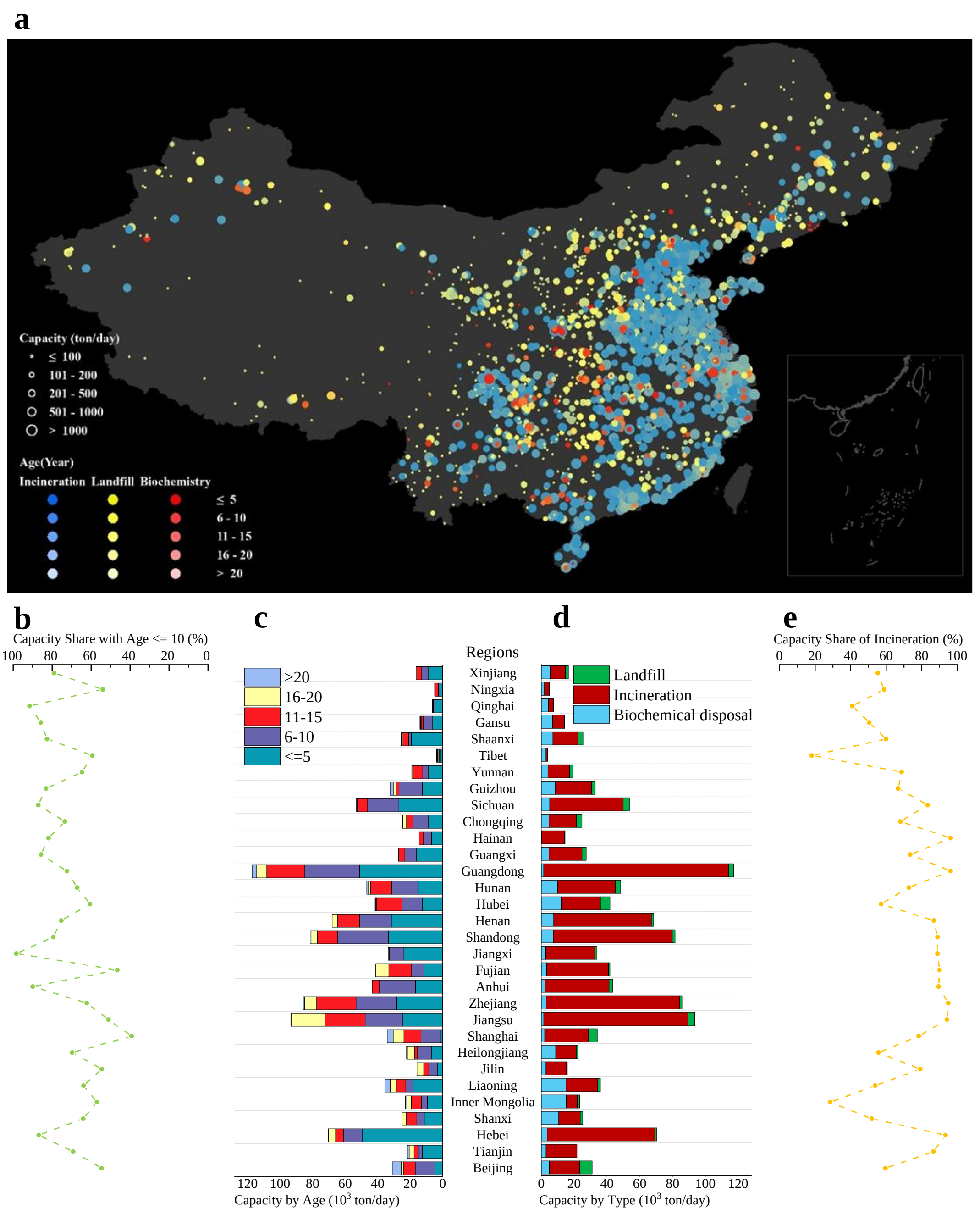

Supplementary Fig. 5 Geographical locations, operation-years, capacities, and disposal modes of MSW disposal plants in China in 2023. a. Location, capacity, age and technologies of China's existing MSW disposal plants in 2023. It shows geographical position, maximum daily disposal quantity and operation-years of the plants. The size of the dots indicates their disposal capacity of the plants, and the color of the dots shows MSW disposal technologies in which the changes of hue represent the operation-years from dark (young plants) to light (old plants). b-e. Capacity share of existing MSW disposal plants in China in 2023, by region, technology and the operation-years. b, e. The curves show capacity share of young plants (operation-years ⩽ 10) and incineration, respectively. c, d. The bars represent the distribution of disposal capacity across regions in China, categorized by operation-years (years since commissioning) and disposal modes, which are indicated by the colors of the bars, respectively.

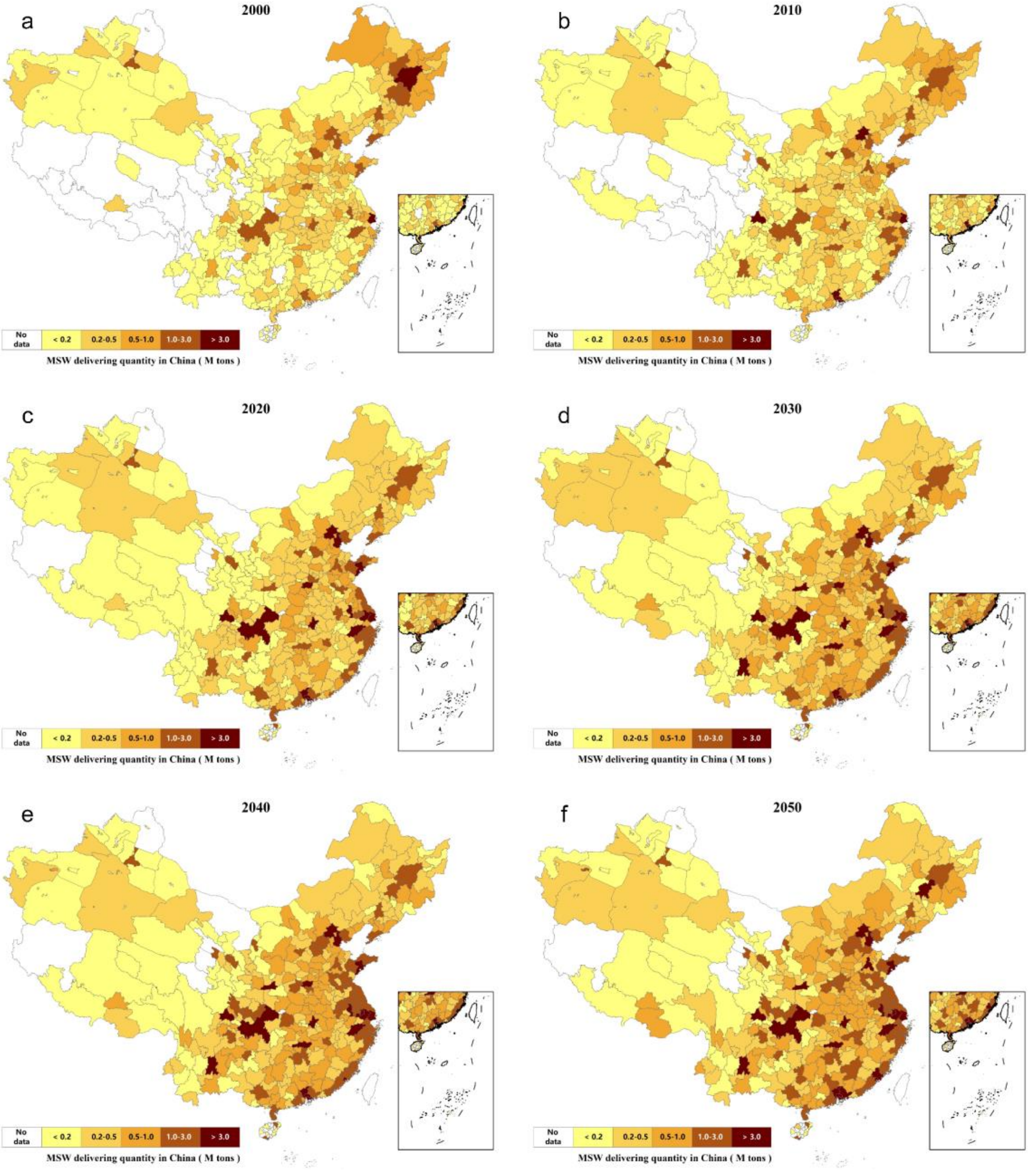

Supplementary Fig. 6 City-level MSW delivering quantity from 2000 to 2050. a-c. The historical MSW delivering quantities of 352 cities in China for the years of 2000, 2010, and 2020. d-f. The forecasted MSW delivering quantities for the years of 2030, 2040, and 2050. The variations in hues represents the MSW delivering quantities of those cities, ranging from lighter shades (lower MSW delivering quantity) to darker shades (higher MSW delivering quantity). The MSW delivering quantities of Laiwu, Qianxinan Autonomous Prefecture, and Yanglingqu are respectively added to those of Jinan, Qianxinan Buyi and Miao Autonomous Prefecture, and Xianyang, considering the changes in the administrative divisions of those areas.

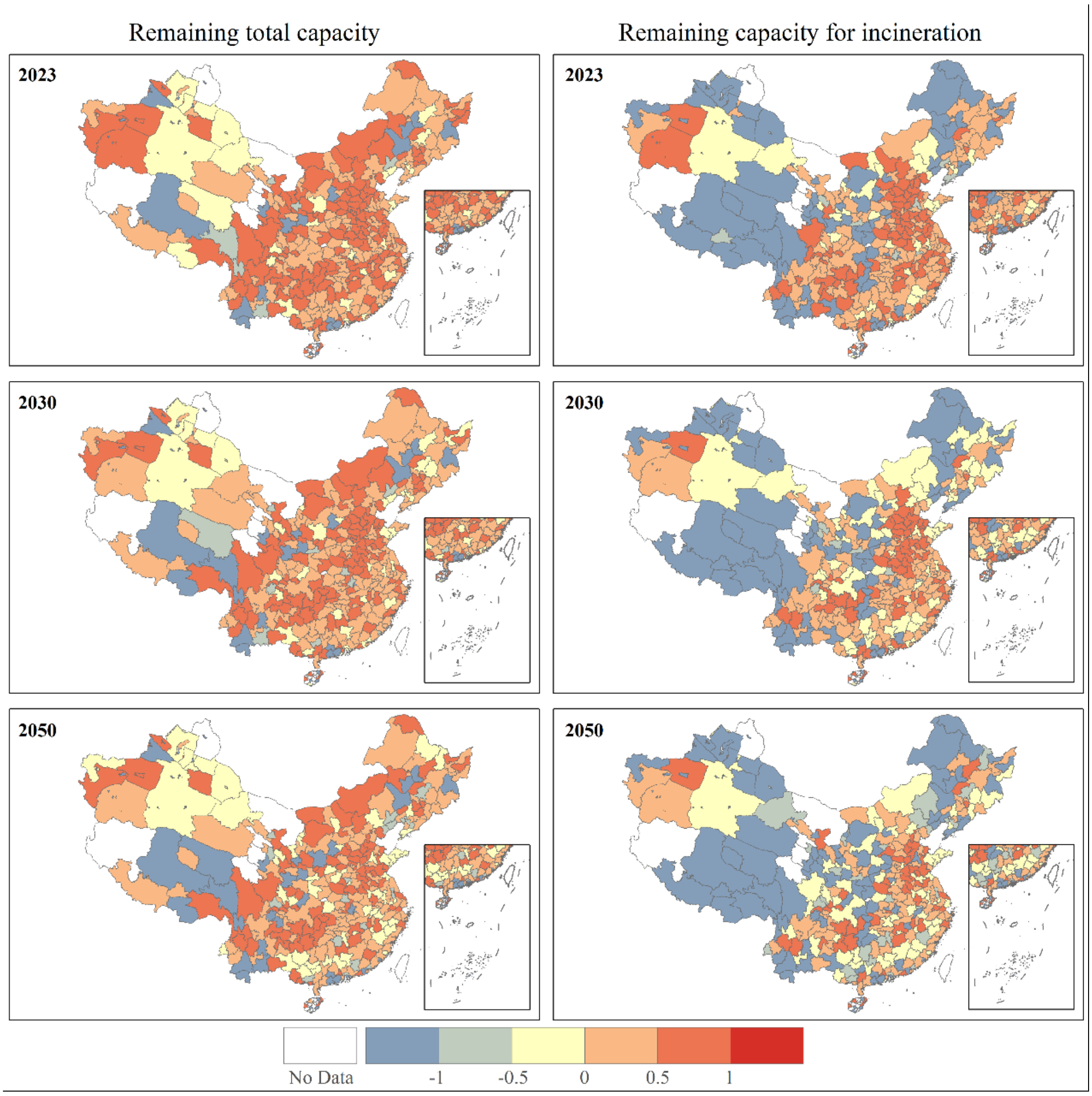

Supplementary Fig. 7 Remaining MSW disposal capacities across cities in ID mode by 2050. a, c, e. Remaining capacities in MSW disposal after subtracting the MSW delivering quantities in ID mode in 2023, 2030, and 2050 from the existing capacities. b, d, f. Remaining capacities in MSW incineration after subtracting the MSW incineration quantities in mode in 2023, 2030, and 2050 from the existing capacities.

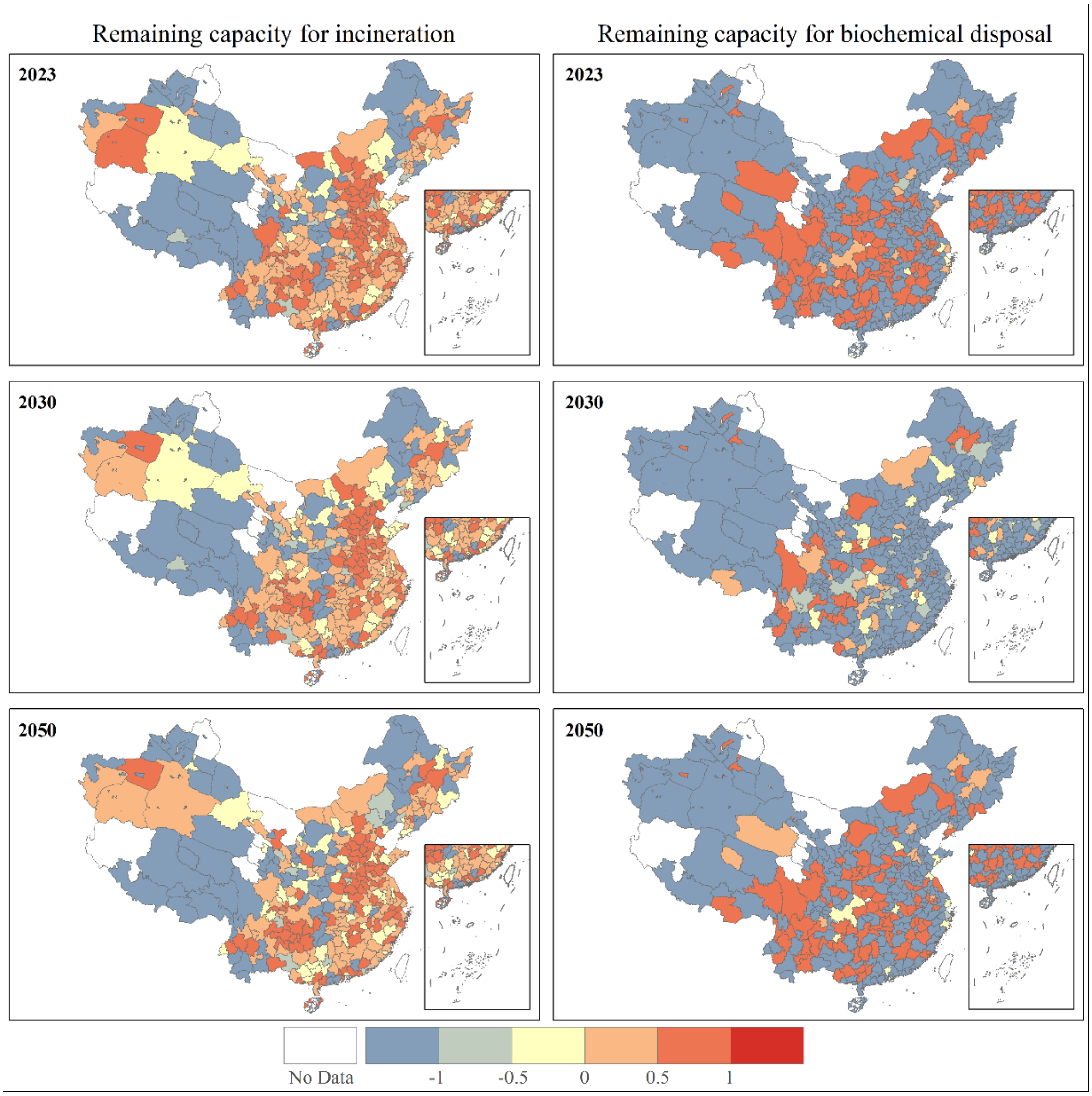

Supplementary Fig. 8 Remaining MSW disposal capacities across cities in LB mode by 2050. a, c, e. Remaining capacities in MSW incineration after subtracting the MSW incineration quantities in LB mode in 2023, 2030, and 2050 from the existing capacities. b, d, f. Remaining capacities in MSW bioconversion disposal after subtracting the quantities of MSW bioconversion disposal in LB mode in 2023, 2030, and 2050 from the existing capacities.

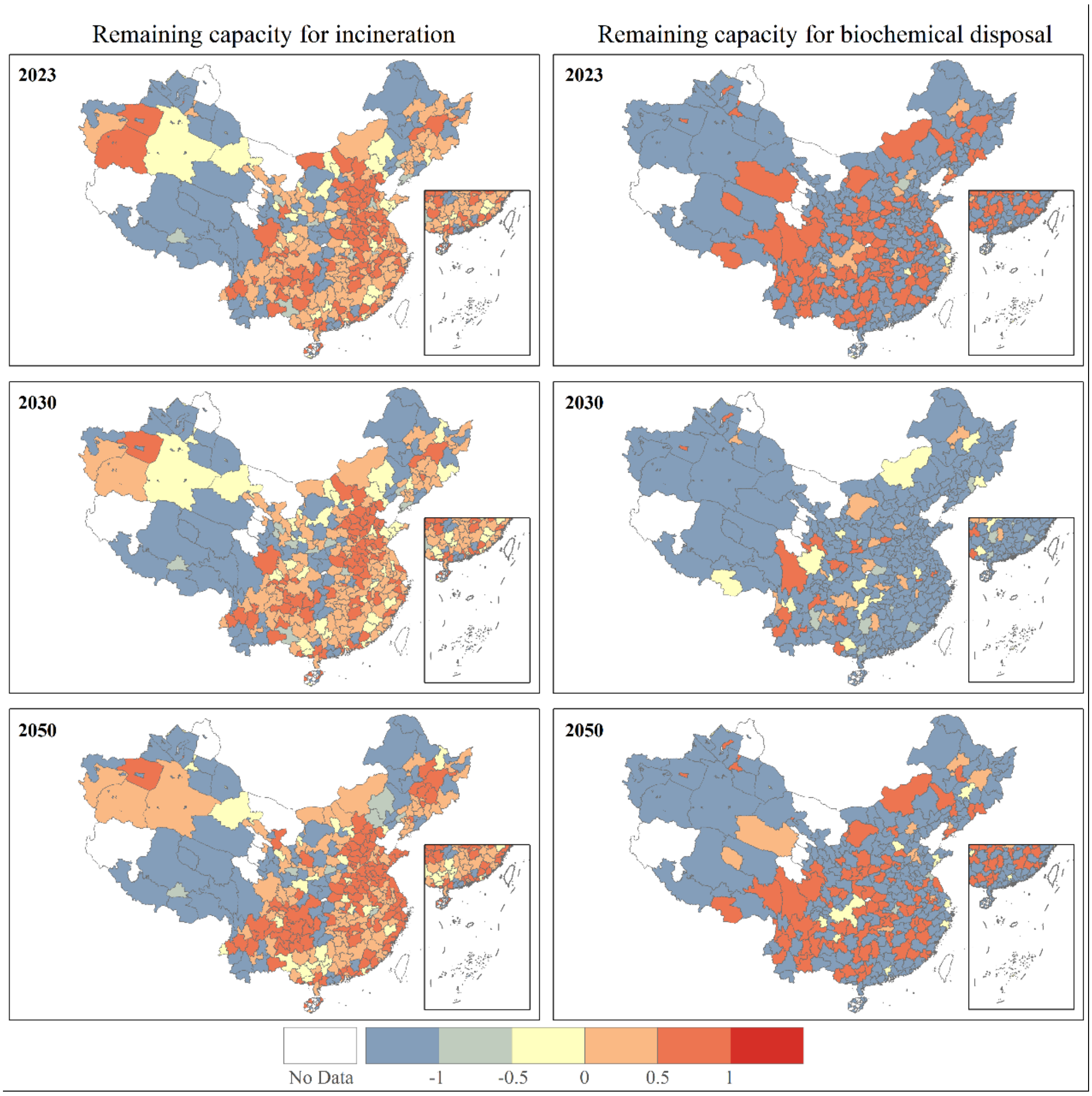

Supplementary Fig. 9 Remaining MSW disposal capacities across cities in HB mode by 2050. a, c, e. Remaining capacities in MSW incineration after subtracting the MSW incineration quantities in HB mode in 2023, 2030, and 2050 from the existing capacities. b, d, f. Remaining capacities in MSW bioconversion disposal after subtracting the quantities of MSW bioconversion disposal in HB mode in 2023, 2030, and 2050 from the existing capacities.

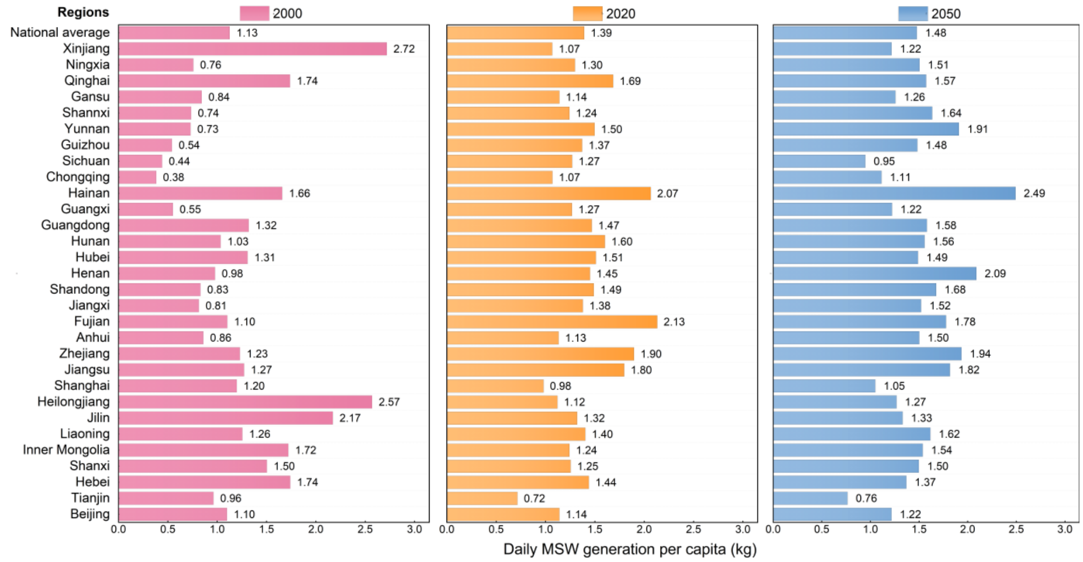

Supplementary Fig. 10 MSW generation per capita in different regions. The results for the years 2000 and 2020 are calculated based on historical data regarding population and MSW delivering quantities in various regions, while the results for the year 2050 are calculated based on the forecasts of future population and MSW delivering quantities. It is noted that we use the MSW delivering quantity to represent the MSW generation.

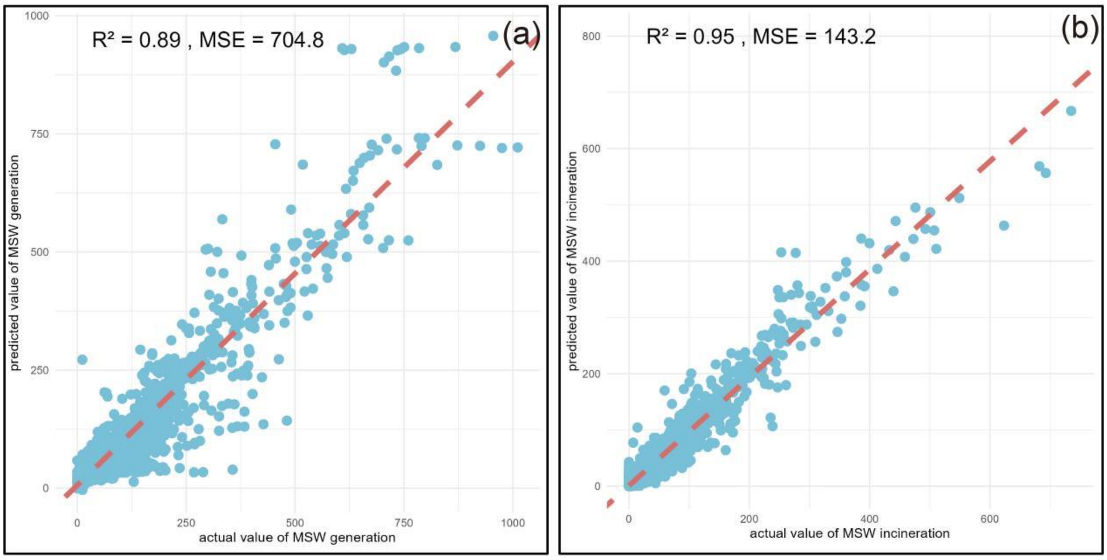

Supplementary Fig. 11 The comparison of predicted value and actual value for MSW generation (a) and MSW incineration (b). Note: the number of data is 7392.

Supplementary Table 1 Distribution of MSW disposal plant capacity based on different operation years

Regions	<=5	5-10	11-15	15-20	>20
**Anhui**	16756	22410	4264	0	85
**Beijing**	4900	12050	7091	1550	5440
**Chongqing**	8800	9420	4084	2330	150
**Fujian**	11431	7780	13960	7850	200
**Gansu**	6233.6	5855.8	1196	423	375
**Guangdong**	51140	33700	23378	6188	2750
**Guangxi**	16308	7020	3691	200	0
**Guizhou**	12560	14351.3	1850	1450	2200
**Hainan**	6180	5700	2625	0	0
**Hebei**	55680	14260	4825	4370	50
**Heilongjiang**	7048	8504.5	1870	4351	530
**Henan**	31629	19555	13570	3220	0
**Hubei**	12645.7	12574	15618.8	399	400
**Hunan**	15063	16330	13083	1185	1100
**Inner Mongolia**	9437.6	3644.6	6221	2549.8	1100
**Jiangsu**	24555	23174	24740	10750	200
**Jiangxi**	23920.5	9012.5	150	393.5	0
**Jilin**	3396	5242	3135	4070	0
**Liaoning**	18476.21	4296	5790	3670	3429.3
**Ningxia**	1859	861.6	2000	320	0
**Qinghai**	4927.37	882.87	270	203.05	60
**Shandong**	33429.2	31322	12336.9	3900	560
**Shanghai**	1100	12300	10500	6587	3650
**Shanxi**	19346.6	1648.7	3531	823	60
**Sichuan**	26983.8	19253	6009	265	550
**Tianjin**	12500	2550	2600	3000	1100
**Tibet**	1463	807	263	704	587
**Xinjiang**	8779.9	4211	2950	460	38
**Yunnan**	8997	3340	6154	464.2	120
**Zhejiang**	28320	25020	24200	7377	825
**National**	336159.7	234879.7	151141.8	54949.3	18009.3

Supplementary Table 2 Average operation years of MSW disposal plants in different regions

Regions	Incineration	Landfill	bioconversion disposal	Total
**Anhui**	8.1	2.7	-	7.8
**Beijing**	8.8	18.0	11.8	11.4
**Chongqing**	4.1	13.3	-	9.0
**Fujian**	6.8	8.8	5.5	8.0
**Gansu**	8.4	-	-	8.4
**Guangdong**	9.1	9.3	6.6	8.8
**Guangxi**	9.6	8.0	8.5	9.3
**Guizhou**	6.2	-	7.3	6.3
**Hainan**	9.5	-	-	9.5
**Hebei**	5.1	17.3	9.0	6.9
**Henan**	7.7	12.3	6.3	10.0
**Heilongjiang**	4.3	10.3	4.5	6.3

**Hubei**	6.7	6.8	10.0	7.0
**Hunan**	7.5	10.0	7.3	7.9
**Jilin**	8.4	12.7	5.3	8.4
**Jiangsu**	4.4	11.4	8.5	10.0
**Jiangxi**	8.6	10.0	9.0	9.7
**Liaoning**	11.5	12.5	21.7	14.0
**Inner Mongolia**	3.7	-	7.0	4.1
**Ningxia**	15.7	-	-	15.7
**Qinghai**	12.0	-	-	12.0
**Shandong**	4.9	9.8	8.8	8.6
**Shanxi**	13.4	17.0	8.5	13.2
**Shaanxi**	6.5	7.0	6.0	6.7
**Shanghai**	3.0	12.9	-	7.9
**Sichuan**	8.2	5.5	6.8	7.8
**Tianjin**	9.2	21.0	9.4	11.1
**Tibet**	4.0	-	-	4.0
**Xinjiang**	4.1	-	-	4.1
**Yunnan**	6.1	12.8	-	7.8
**Zhejiang**	7.2	6.9	7.7	7.0
**National**	6.6	9.4	7.6	8.0

Supplementary Table 3 Parameters and inventory data for MSW fluidized bed incineration and grate firing incineration

**Fluidized bed incineration**		**Grate firing incineration**	
**Input**			
Coal (kg · ton MSW^{-1})	96.6	Diesel (kg · ton MSW^{-1})	1.5
Lime (kg · ton MSW^{-1})	9.92	Lime (kg · ton MSW^{-1})	11.9
Activated carbon (kg · ton MSW^{-1})	0.46	Activated carbon (kg · ton MSW^{-1})	0.23
**Output**			
Electricity (kWh · ton MSW^{-1})	239.4	Electricity (kWh · ton MSW^{-1})	312
Fly ash [a] (kg · ton MSW^{-1})	81.6	Fly ash [b] (kg · ton MSW^{-1})	29.84
Bottom ash (kg · ton MSW^{-1})	114	Bottom ash (kg · ton MSW^{-1})	179.13

The Parameters and inventory data for MSW incineration refer to [12]. a. The disposal cost of fly ash is included with the other costs for MSW incineration, which amounts to 38.8 CNY per ton in this study.

Supplementary Table 4 Parameters and inventory data for MSW landfill

**Input**			
HDPE (kg · ton MSW^{-1})	2.47	Diesel (kg · ton MSW^{-1})	0.43
Leachate volume (L · ton MSW^{-1})	0.42	Electricity (kWh · ton MSW^{-1})	30
**Output**			

Landfill gas collection rate (%)	56.7	CH_4 content in Landfill gas (%)	52.2
Landfill gas generated from 1 ton of different compositions of MSW (m^3 · ton MSW^{-1}) [a]		Paper	373.95
		Wood and bamboo	365.56
		Textile	420.70
		Organic components	204.41

The Parameters and inventory data for MSW landfill refer to [12]. a. Landfill gas generated from 1 ton of different compositions is calculated based on methane generation rate constant and degradable organic carbon, which refers to [12]. The feed-in tariff for power generated from landfill gas, with an efficiency of 0.33, is consistent with that for MSW incineration power generation. All landfill gas power generation revenue from MSW in the future is included in the landfill costs for the year in which it is landfilled.

Supplementary Table 5 Parameters and inventory data for MSW compost

Input			
PAC (kg · ton MSW^{-1})	0.75	PAM (kg · ton MSW^{-1})	0.08
NaClO (kg · ton MSW^{-1})	0.01	Electricity (kWh • ton MSW^{-1})	69.66
**Output**			
Fertilizer (ton • ton MSW^{-1})	0.19		

The Parameters and inventory data for MSW compost refer to [8].

Supplementary Table 6 Parameters and inventory data for MSW bioconversion

		Bioconversion technologies [a]			
		**B1**	**B2**	**B3**	**B4**
**Input**	Rice hull powder (ton · ton MSW^{-1})	0.25			
	Rice/wheat bran (kg · ton MSW^{-1})		50		
	Tap water (kg · ton MSW^{-1})		426	620	7.29
	PAC (kg · ton MSW^{-1})		1.64	2.1	2.01
	PAM (kg · ton MSW^{-1})		0.16	0.21	0.2
	NaClO (kg · ton MSW^{-1})		0.02	0.03	0.03
	NaOH (kg · ton MSW^{-1})				0.41
	Plant liquid (kg · ton MSW^{-1})			0.5	0.5

	Electricity (kWh · ton MSW^{-1})	25.19	18.25	71.82	32.21
	Natural gas (m^3 · ton MSW^{-1})			4.93	
**Output**	Fertilizer (ton · ton MSW^{-1})	0.13	0.07	0.20	0.13
	Protein [b] (ton · ton MSW^{-1})	0.03	0.01	0.03	0.02
	Biooil (ton · ton MSW^{-1})		0.02	0.02	0.02

The Parameters and inventory data for MSW bioconversion refer to [8]. a. The operational benefit parameters for bioconversion are based on the weighted average of 4 technologies in [8], with ratios of 25%, respectively. B1: bioconversion for black soldier fly (BSF); B2: bioconversion for BSF and biooil; B3: bioconversion for red head fly (RHF) and biooil; B4: heat hydrolysis and bioconversion. b. Protein represents black soldier flies in B1 and B2, and red head flies in B3 and B4, respectively.

Supplementary Table 7 Prices of products, energy, and materials involved in MSW incineration, landfill, bioconversion disposal, and recycling.

**Energy and water**					
Coal (CNY · ton^{-1})	762.5	Natural gas (CNY · (m^3)$^{-1}$)	3.54	Tap water (CNY · ton^{-1})	4.1
Electricity [a] (CNY · kWh^{-1})	0.66	Diesel [a] (CNY · kg^{-1})	8.53		
**Products [b] and materials involved in incineration**					
Lime (CNY · ton^{-1})	362	Activated carbon (CNY · ton^{-1})	11633	Bottom ash (CNY · ton^{-1})	65.37
**Products and materials involved in landfill**					
HDPE (CNY · ton^{-1})	8419				
**Products and materials involved in bioconversion disposal**					
Rice hull powder (CNY · ton^{-1})	285	Rice/wheat bran (CNY · ton^{-1})	2170	PAC (CNY · kg^{-1})	1.75
PAM (CNY · kg^{-1})	13.4	NaClO (CNY · kg^{-1})	0.3	NaOH (CNY · kg^{-1})	0.87
Plant liquid (CNY · kg^{-1})	9	Fertilizer (CNY · ton^{-1})	400 [8]	Biooil (CNY · ton^{-1})	4800 [8]
Black soldier (CNY · ton^{-1})	9000 [8]	Red head fly (CNY · ton^{-1})	3000 [8]		
**Recycled materials**					
Recycled paper	3129 [14]	Recycled glass	196 [14]	Recycled plastic	1077 [12]
Recycled textile	4500 [14]	Recycled metals	6209 [14]		

a. Electricity prices for general industrial and commercial uses and diesel prices across various provinces in China are adopted, and the average values are presented above. b. The subsidized electricity price for waste incineration in China is explained in Supplementary Text 3.

Supplementary Table 8 Statistics on the composition of MSW across cities in China

%	Max	Min	Mean	SD
**Organic component**	68.38	0.41	45.07	15.24
**Paper**	53.02	6.47	26.68	13.86
**Plastic**	17.22	0.22	13.54	1.59
**Textile**	5.09	1.37	2.10	0.69
**Glass**	50.53	1.68	2.00	3.64
**Metal**	4.17	0.64	0.71	0.43
**Ash content**	64.49	0.01	6.63	6.60
**Wood and bamboo**	22.07	0.00	2.89	2.97
**Other**	1.14	0.39	0.40	0.04

Supplementary Table 9 Parameters for the recycling of paper, plastics, glass, textiles and metals in MSW

	Paper	Plastic	Glass	Textile	Metal
			**Recycling**		
**Substitution ratio (%)**	80 [15]	93.5 [14]	78.1 [16]	90.09 [17]	87.5 [14]
**Processing cost ($CNY \cdot ton^{-1}$)**	1964 [18]	600 [19]	97.5 [20]	1573 [21]	157.5 [20]
**Collection rate (%)**	51.2 [22]	30.65 [23]	43.62 [22]	21.94 [24]	65 [25]
			**Sorting and transportation**		
**Sorting cost [a] ($CNY \cdot ton^{-1}$)**			436.51 [25]		
**Transportation cost ($CNY \cdot ton^{-1}$)**			66.94 [25]		

a. The sorting cost varies with the differences in the ratios of recyclable materials to MSW across different cities.

Supplementary Table 10 Evolution of China's MSW issues and policies since reform and opening up

Time Period	Stage	Waste Management Background & Issues	Policy Responses
1980-1990	Initial Response	With accelerating urbanization, municipal waste volume increased rapidly, but waste treatment facilities were severely inadequate [26], leading to waste siege and environmental pollution issues.	1. Started construction of landfills and incinerators to handle increasing waste volume. 2. Issued Municipal Waste Management Measures (1993), initially standardizing waste treatment processes.

1990-2000	Systematic Response	Continued increase in waste volume with more complex composition [27,28], increasing organic matter, combustibles, and recyclables while decreasing inorganic content; outdated collection and transportation methods.	1. Proposed principles of reduction, resource utilization, and harmless treatment, began advocating waste sorting and resource recycling.<br>2. Construction Ministry issued Municipal Waste Treatment and Pollution Prevention Technical Policy (2000), systematically outlining technical approach of sorting as prerequisite, combining landfill, composting, incineration, and recycling.
2000-2010	Comprehensive Management Exploration	Gradual progress in waste treatment facility construction, increasing harmless treatment rate [29], but waste sorting and resource recycling needed strengthening [30].	1. Enacted Circular Economy Promotion Law (2009), promoting resource recycling and waste reduction. 2. Implemented waste sorting system, began pilot programs (2010), promoting end-point reduction.
2010-2020	Management Approach Correction	Continued growth in urban waste generation, more mixed composition; sorting ineffective due to mixed disposal at source, mixed transportation, and mismatched terminal treatment [31].	1. Waste Sorting System Implementation Plan (2017) mandated sorting in urban areas of 46 cities, targeting 35% recycling by 2020.<br>2. Notice on Comprehensive Implementation of Waste Sorting in Prefecture-level Cities (2019), aiming for complete sorting systems in all prefecture-level cities and 4 municipalities by 2025.
2020-	Comprehensive Promotion & Reform Deepening	Gradual improvement of sorting and treatment systems, significant enhancement in resource utilization and harmless treatment capacity, but continued optimization needed for sustainable development.	1. 14th Five-Year Plan for Urban Waste Sorting and Treatment Facilities Development (2021) targets 60% resource utilization rate by 2025.<br>2. Carbon Peaking and Carbon Neutrality Goals (2021) emphasizes waste reduction and resource utilization as key components of circular economy carbon reduction.

Supplementary Table 11 Local waste sorting related policies in China

City	Time	Policy
Chongqing	2019.01	Chongqing Municipal Waste Sorting Management Measures
Shanghai	2019.07	Shanghai Municipal Waste Management Regulations
Hangzhou	2019.08	New Hangzhou Municipal Waste Management Regulations
Xi'an	2019.09	Xi'an Municipal Waste Sorting Management Measures
Guangzhou	2019.12	New Guangzhou Municipal Waste Sorting Management Measures
Zhengzhou	2019.12	Zhengzhou Urban Waste Sorting Management Measures
Shenyang	2019.12	Waste Sorting Signs

Harbin	2020.02	Harbin Urban Waste Sorting Management Measures
Beijing	2020.05	New Beijing Municipal Waste Management Regulations
Suzhou	2020.06	Suzhou Municipal Waste Sorting Management Regulations
Dongguan	2020.06	Dongguan Municipal Waste Sorting Three-Year Action Plan (2020-2022)
Qingdao	2020.06	Qingdao Municipal Waste Sorting Measures
Wuhan	2020.07	Wuhan Municipal Waste Sorting Management Measures
Shenzhen	2020.09	Shenzhen Municipal Waste Sorting Management Regulations
Changsha	2020.10	Changsha Municipal Waste Management Regulations
Hohhot	2020.10	Hohhot Municipal Waste Sorting Management Measures
Nanjing	2020.11	Nanjing Municipal Waste Management Regulations
Tianjin	2020.12	Tianjin Municipal Waste Management Regulations
Hefei	2020.12	Hefei Municipal Waste Sorting Management Regulations
Chengdu	2021.03	Chengdu Municipal Waste Sorting Implementation Plan
Jinan	2021.05	Jinan Municipal Waste Reduction and Sorting Management Regulations

Supplementary Table 12 Waste incineration planning and completion since the 12th five-year plan

Year	Policy	Main content related to waste incineration	Planned capacity proportion	Completion status
2012	12th Five-Year Plan for National Urban Waste Harmless Treatment Facilities Construction	1. Regions with suitable conditions should prioritize incineration and other resource utilization treatment technologies.<br>2. By 2015, national urban waste incineration treatment facility capacity should reach over 35% of total harmless treatment capacity, with eastern regions reaching over 48%.	Urban 35%	2015: Urban 28%
2016	13th Five-Year Plan for National Urban Waste Harmless Treatment Facilities Construction	1. By end of 2020, municipal waste incineration treatment capacity should account for over 50% of total harmless treatment capacity, with eastern regions reaching over 60%.<br>2. By end of 2020, qualified municipalities, specially designated cities, and provincial capitals (built-up areas) should achieve zero landfill for raw waste.	Urban 50%	2020: Urban 59%

2021	14th Five-Year Plan for Urban Waste Classification and Treatment Facilities Development	1. By end of 2025, national urban waste incineration treatment capacity should reach about 800,000 tons/day, with urban waste incineration treatment capacity proportion around 65%.<br>2. In principle, cities at prefecture level and above, and counties with incineration treatment capacity or construction conditions, should no longer plan and build new raw waste landfill facilities; existing landfill capacity should be converted to backup facilities.	Urban 65%	2022: Urban 73%

Supplementary Table 13 Key policies in household food waste treatment industry

Policy	Time	Content	Significance
Notice on Organizing Urban Household Food Waste Resource Utilization and Harmless Treatment Pilot Work	2010.05	Proposed selecting first batch of pilot cities for Household Food Waste resource utilization and harmless treatment nationwide, gradually establishing suitable regulations, policies, and technical routes to improve resource utilization levels.	Initiated China's Household Food Waste treatment market
Resource Comprehensive Utilization Products and Services VAT Preferential Catalog	2015.06	Implemented 100% VAT refund policy for resource products: biogas fuel blocks, biogas, electricity, heat produced from Household Food Waste.	Tax incentives to increase terminal disposal enterprises' initiative
Strategic Emerging Industries Key Products and Services Guidance Catalog	2017.01	Listed Household Food Waste resource utilization and harmless treatment related equipment and technology R&D as part of resource recycling industry	Future Household Food Waste treatment technology development will be resource-oriented
Shanghai Household Food Waste Oil Treatment Management Measures Implementation Provisions	2022.06	Specified requirements for waste oil treatment units and collection units regarding bidding, collection containers, personnel training, supervision, etc.	Multiple provinces and cities issued documents promoting Household Food Waste collection and resource utilization
Beijing 14th Five-Year Urban Management Development Plan	2022.04	Promoted exploration of Household Food Waste and sewage co-treatment, actively promoted biogas resource utilization from Household Food Waste treatment facilities, explored comprehensive utilization of fly ash and incineration slag.	

Guangzhou Household Food Waste Management Measures	2021.10	Regulated Household Food Waste discharge, collection, disposal, with three-year policy validity
Chongqing Municipal Waste Management Regulations	2021.11	Encouraged Household Food Waste generators to use new technology and equipment for oil-water separation, promoting source reduction

477

References

1. Teng FY, Wang ZY, Ren K, et al. Analysis of composition characteristics and treatment techniques of municipal solid waste incineration fly ash in China[J]. Journal of Environmental Management, 2024, 357: 120783.
2. Chinese Ministry of Ecology and Environment (CMEE). (2023). Automatic monitoring data public platform for household waste incineration power plants. https://ljgk.envsc.cn.
3. Chinese National Development and Reform Commission (CNDRC). (2012). Notice on improving the price policy for waste incineration power generation. https://www.gov.cn/zwgk/2012-04/10/content_2109921.htm.
4. Zhang M, Wei JX, Li H, et al. Comparing and optimizing municipal solid waste (MSW) management focused on air pollution reduction from MSW incineration in China[J]. Science of The Total Environment, 2024, 907: 167952.
5. Zhou ZQ, Zhang L. Sustainable waste management and waste to energy: Valuation of energy potential of MSW in the Greater Bay Area of China[J]. Energy Policy, 2022, 163: 112857.
6. Xue YS, Moreno JM, Li CJ, et al. Growing community-based composting programs in China: Implementation and policy lessons from eight cases[J]. Resources, Conservation and Recycling, 2025, 212: 107882.
7. Bohm K, Hatley GA, Robinson BH, et al. Black Soldier Fly-based bioconversion of biosolids creates high-value products with low heavy metal concentrations[J]. Resources, Conservation and Recycling, 2022, 180: 106149.
8. Liu, F., Xin, L., Tang, H. et al. Regionalized life-cycle monetization can support the transition to sustainable rural food waste management in China. Nat Food 4, 797–809 (2023).
9. People's Government of China. (2024). 15 cities will create demonstration models for waste sorting [EB/OL]. Available at: https://www.beijing.gov.cn/ywdt/zybwdt/202405/t20240527_3694298.html [in Chinese]
10. Ministry of Housing and Urban-Rural Development of the People's Republic of China. (2012). Technical specification for kitchen waste treatment: CJJ 184-2012. Beijing: China Architecture & Building Press. [in Chinese]
11. Yang, N., Shao, L. M., & He, P. J. (2018). Analysis of moisture content and characteristics of municipal solid waste components in China. China Environmental Science, (3), 1033-1038. [in Chinese]
12. Fang W, Ding YM, Geng JH, et al. High potential of coupling the source-separation and incineration promotion to reduce costs based on city-level cost-benefit analysis of municipal solid waste management strategies in China[J]. Resources, Conservation and Recycling, 2023, 197: 107099.
13. Octavianthy, D., Syauqi, A., Reyseliani, N. et al. Multi-period Enviro-Economic Optimization of Municipal Solid Waste to Electricity. Waste Biomass Valor 13, 3707–3722 (2022).
14. Zaman AU. A comprehensive study of the environmental and economic benefits of resource recovery from global waste management systems[J]. Journal of

Cleaner Production, 2016, 124: 41-50.

15. Liu MZ, Tan S, Zhang MY, et al. Waste paper recycling decision system based on material flow analysis and life cycle assessment: A case study of waste paper recycling from China[J]. Journal of Environmental Management, 2020, 255: 109859.
16. Yuan XH, Wang JB, Song QM, et al. Integrated assessment of economic benefits and environmental impact in waste glass closed-loop recycling for promoting glass circularity[J]. Journal of Cleaner Production, 2024, 444: 141155.
17. Mu BN, Yu XQ, Shao YY, et al. Complete recycling of polymers and dyes from polyester/cotton blended textiles via cost-effective and destruction-minimized dissolution, swelling, precipitation, and separation[J]. Resources, Conservation and Recycling, 2023, 199: 107275.
18. Li JG, Mei MY, Han YL, et al. Life cycle cost assessment of recycled paper manufacture in China[J]. Journal of Cleaner Production, 2020, 252: 119868.
19. Faraca G, Sanchez VM, Astrup TF. Environmental life cycle cost assessment: Recycling of hard plastic waste collected at Danish recycling centres[J]. Resources, Conservation and Recycling, 2019, 143: 299-309.
20. Albizzati PF, Foster G, Gaudillat P, et al. A model to assess the environmental and economic impacts of municipal waste management in Europe[J]. Waste Management, 2024, 174: 605-617.
21. Mu BN, Yu XQ, Shao YY, et al. Complete recycling of polymers and dyes from polyester/cotton blended textiles via cost-effective and destruction-minimized dissolution, swelling, precipitation, and separation[J]. Resources, Conservation and Recycling, 2023, 199: 107275.
22. China National Resources Recycling Association. (2024). Development report on renewable resource recycling industry in China. http://www.crra.com.cn/.
23. National Development and Reform Commission. (2024). Comprehensive research report on chemical cycle of waste plastics. http://download.china.cn/ch/综合性研究报告中文版 20240419.pdf
24. National Development and Reform Commission. (2023). Research report on the current status of low-value recyclable materials recycling and utilization in http://download.china.cn/idc/中国低值可回收物回收利用现状调查报告.pdf
25. Li GH, Wang WJ, You XY. Social-economic assessment of integrated waste pickers in municipal solid waste management system: A case of Tianjin in China[J]. Journal of Cleaner Production, 2024, 434: 140302.
26. Yi, J. T. (1996). Discussion on urban domestic waste issues. Modern Urban Research, (04), 62-64. [in Chinese]
27. Wang, W. P. (2000). Research on countermeasures of urban domestic waste in China. Journal of Natural Resources, (02), 128-132. [in Chinese]
28. Du, W. P., Gao, Q. X., Zhang, E. C., et al. (2006). Analysis of municipal solid waste disposal and trends in China. Research of Environmental Sciences, (06), 115-120. [in Chinese]
29. Wang, Y. N. (2010). Research on current situation and development of municipal solid waste incineration power generation in China's large cities. Macroeconomics,

(11), 12-23. [in Chinese]
30. Wu, Y. C., & Xu, L. F. (2013). Analysis of the barrier factors of municipal solid waste classification recycling. Advanced Materials Research, 726-731, 2618-2621.
31. Peng, Y., Li, L., Peng, X. Y., et al. (2018). Development process, obstacles and countermeasures of domestic waste classification in China. China Environmental Science, 38(10), 3874-3879. [in Chinese]

Supplementary File 2

Historical MSW disposal structure in 20 typical cities. The internal pie chart represents the proportion of harmless disposal quantity to the total MSW delivering quantity, while the external ring chart indicates the proportions of various disposal technologies. Additionally, the size of the rings directly proportional to the MSW delivering quantities of typical cities in the same historical year.

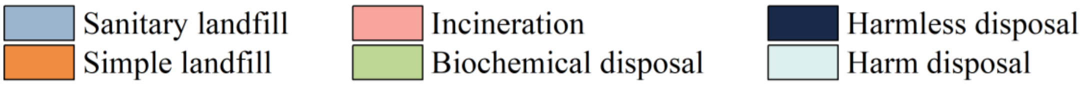

Shanghai-2000:

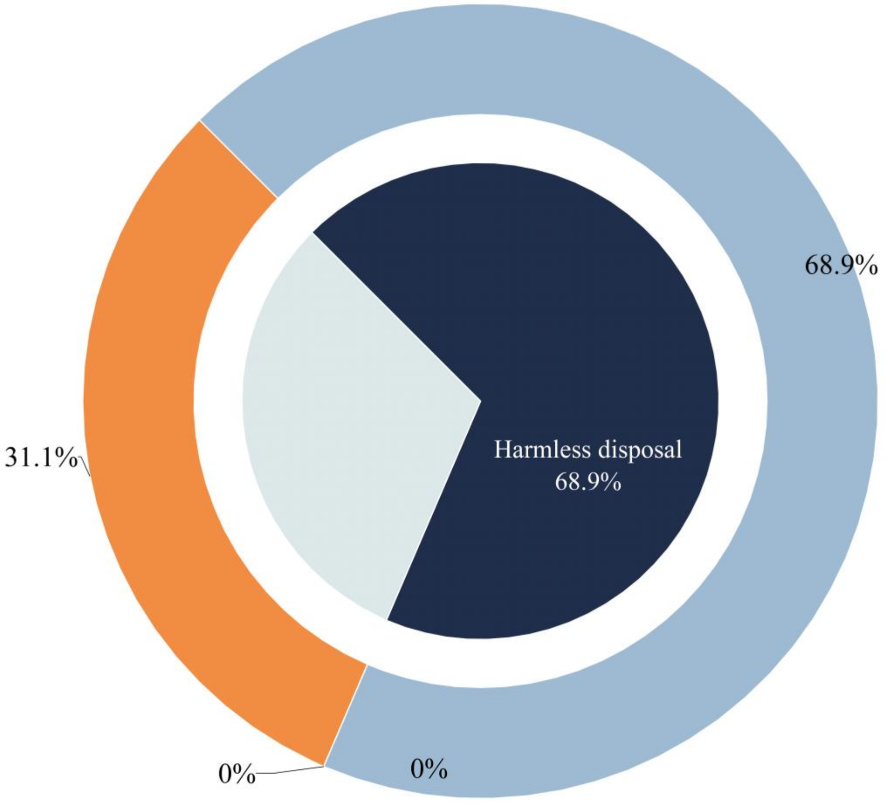

Beijing-2000:

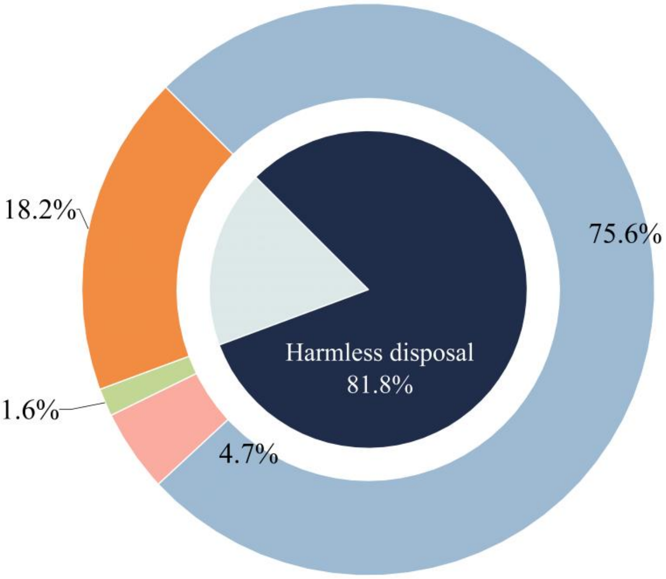

Nanchang-2000:

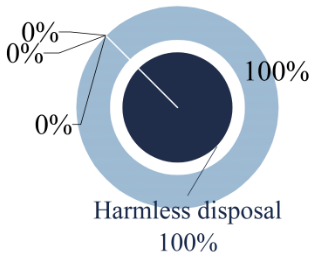

Hefei-2000:

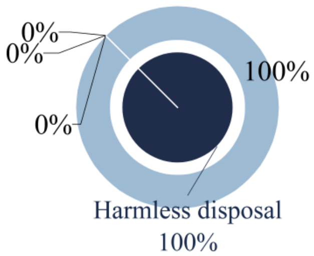

Hohhot-2000:

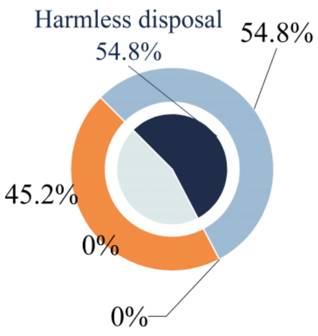

Shangqiu-2000:

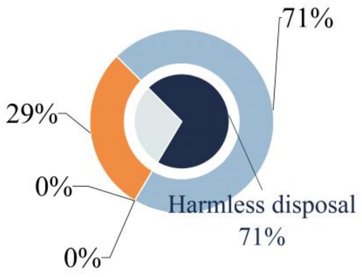

Jiangxing-2000:

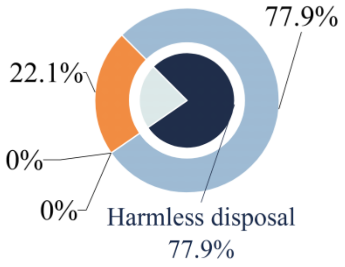

Daqing-2000:

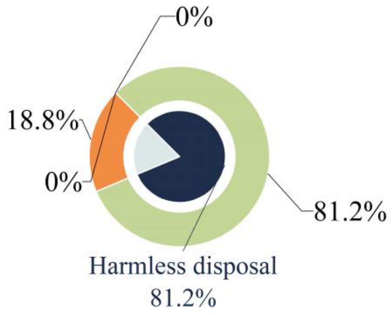

Tianjing-2000:

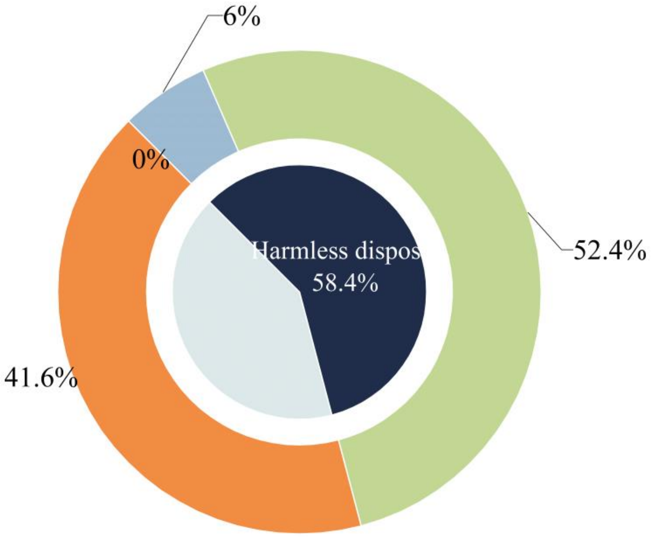

Guangan-2000:

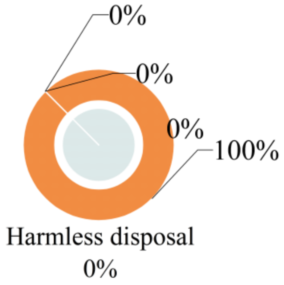

Guangzhou-2000:

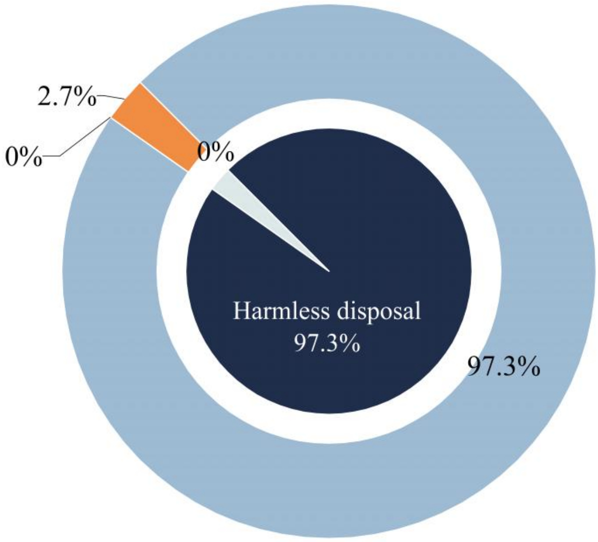

Kunming-2000:

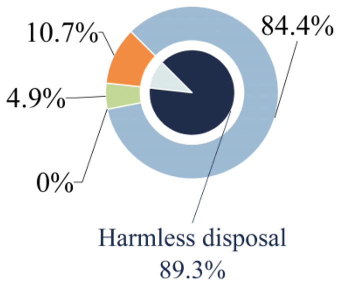

Wuhan-2000:

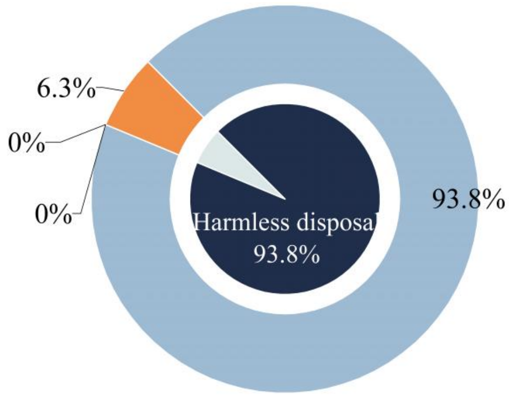

Shenzhen-2000:

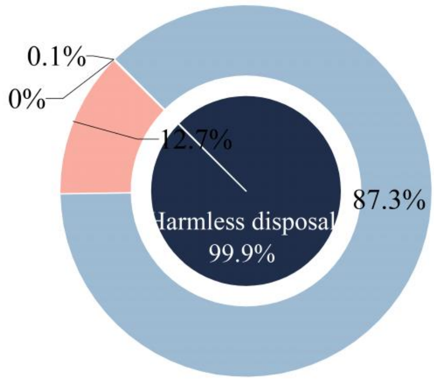

Baiyin-2000:

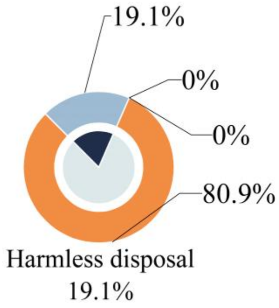

Xian-2000:

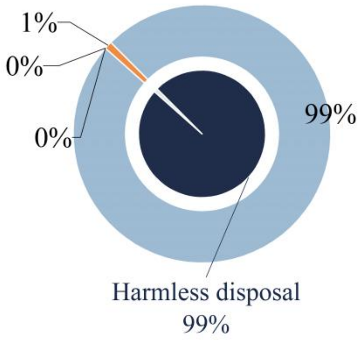

Chongqing-2000:

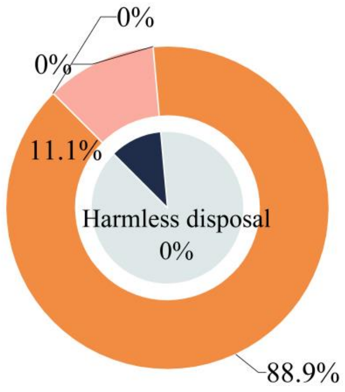

Changsha-2000:

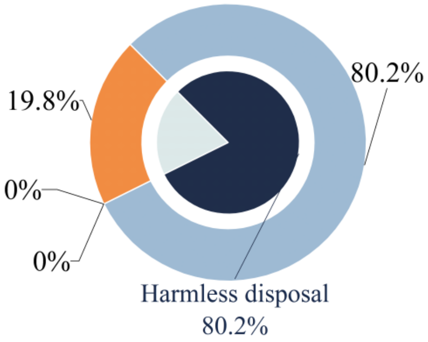

Aksu prefecture -2000:

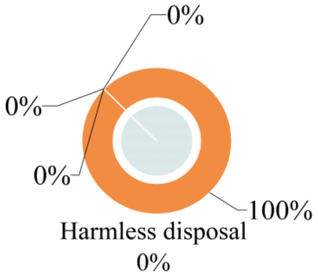
0%
0%
0%
100%
Harmless disposal
0%

Anshan-2000:

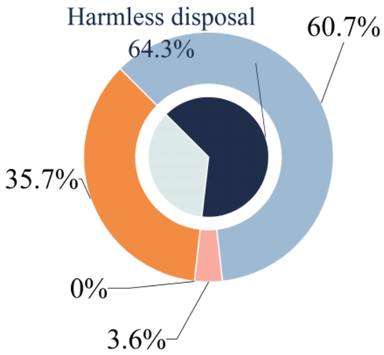
Harmless disposal
64.3%
60.7%
35.7%
0%
3.6%

Shanghai-2010:

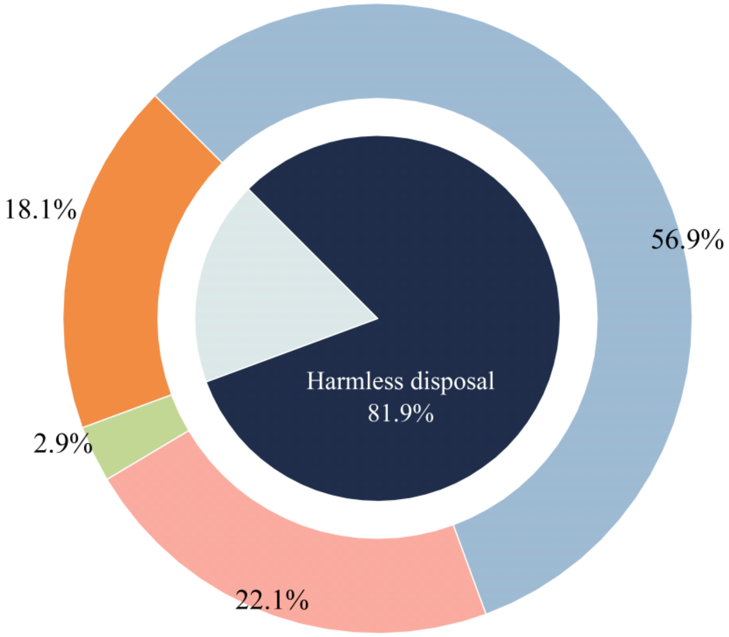

Beijing-2010:

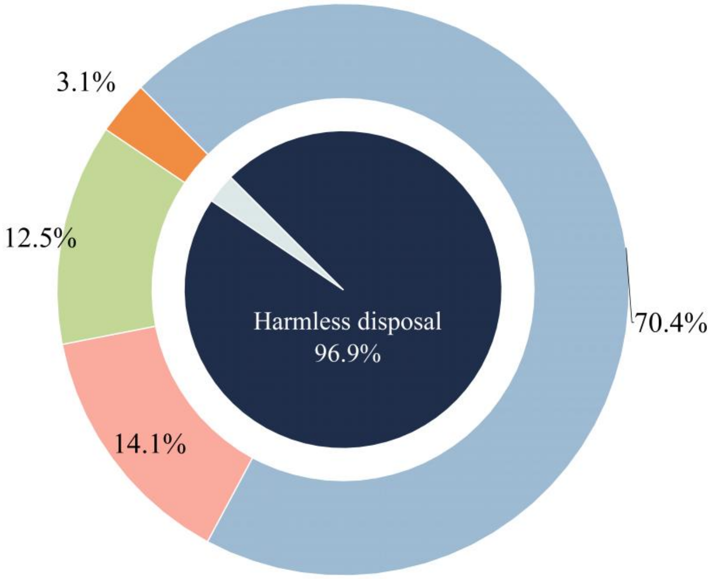

Nanchang-2010:

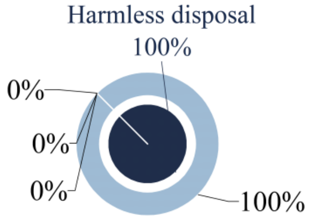

Hefei-2010:

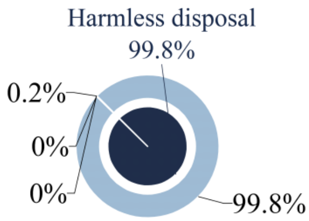

Hohhot-2010:

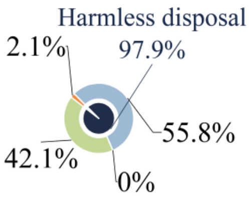

Shangqiu-2010:

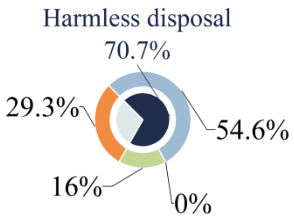

Jiaxing-2010:

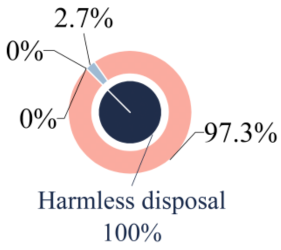

Daqing-2010:

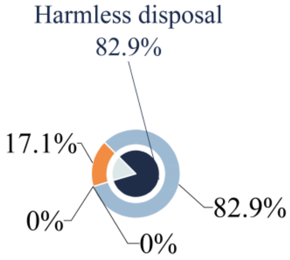

Tianjin-2010:

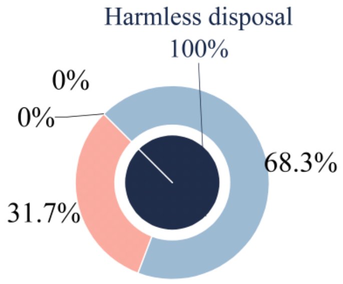

Guangan-2010:

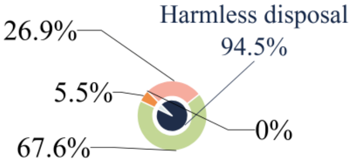

Guangzhou-2010:

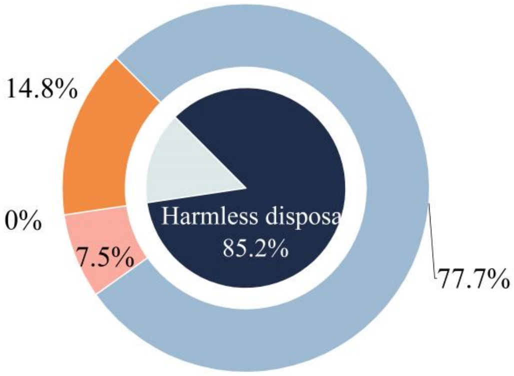

Kunming-2010:

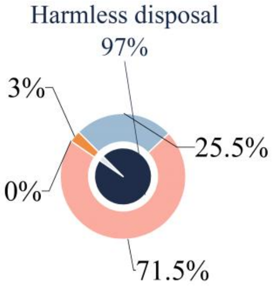

Wuhan-2010:

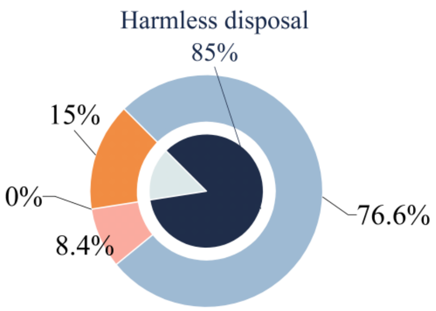

Shenzhen-2010:

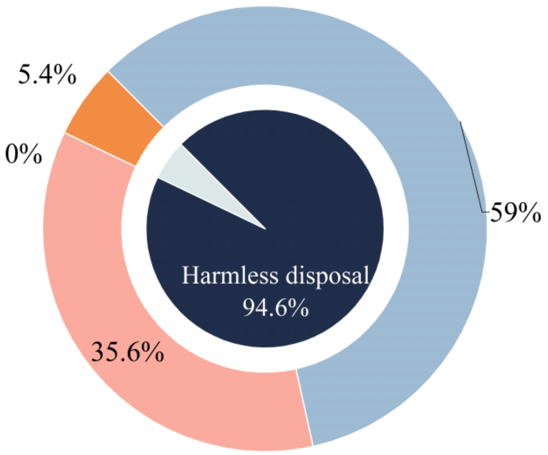

Baiyin-2010:

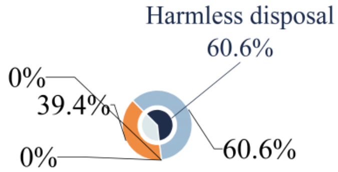

Xian-2010:

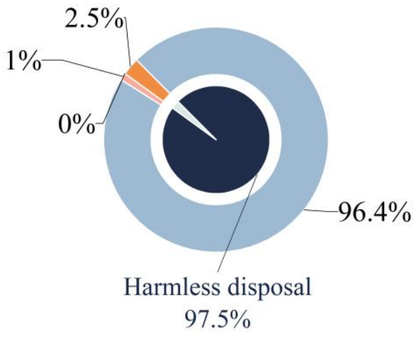

Chongqing-2010:

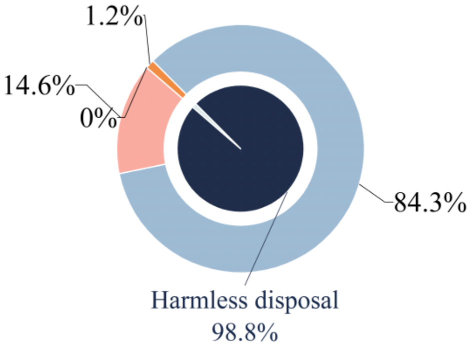

Changsha-2010:

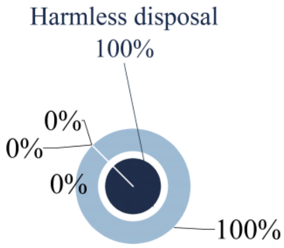

Aksu prefecture-2010:

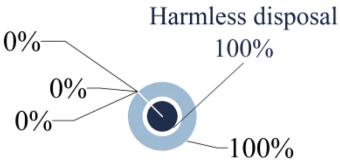

Anshan-2010:

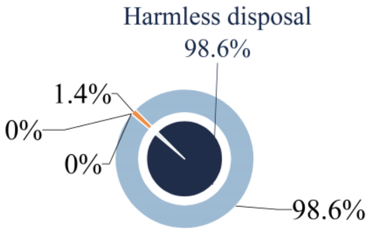

Shanghai-2020:

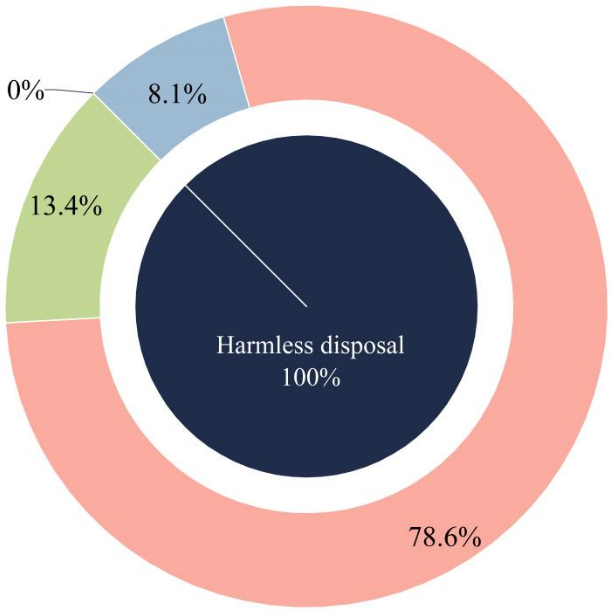

Beijing-2020:

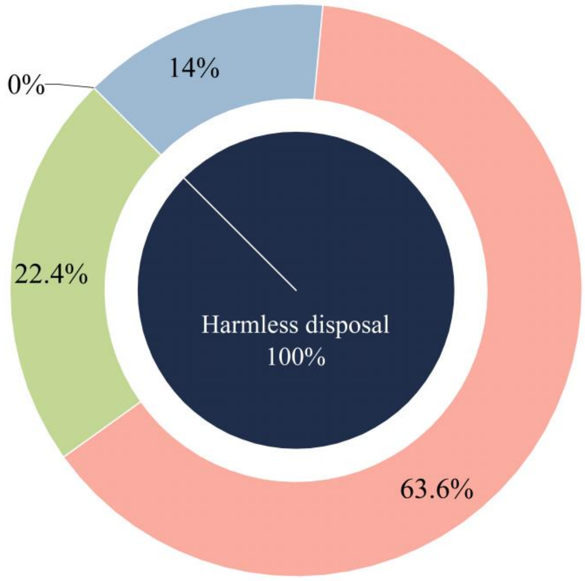

Nanchang-2020:

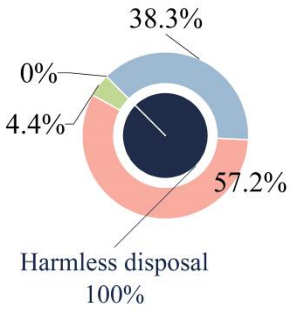

Hefei-2020:

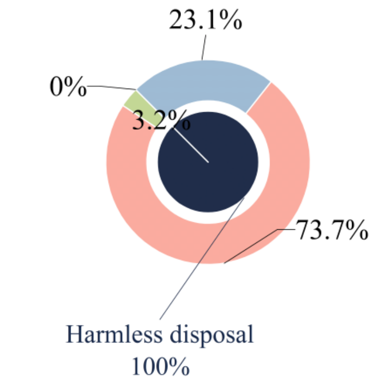

Hohhot-2020:

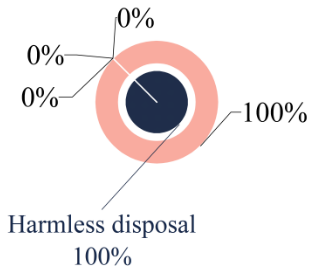

Shangqiu-2020:

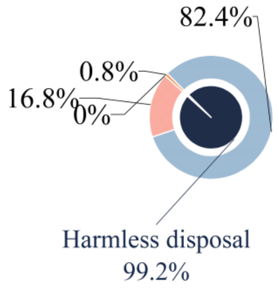

Jiaxing-2020:

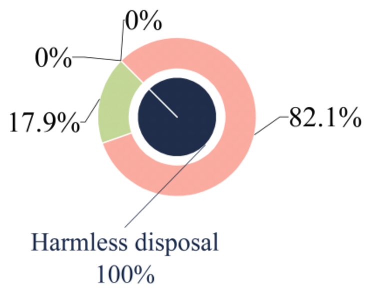

Daqing-2020:

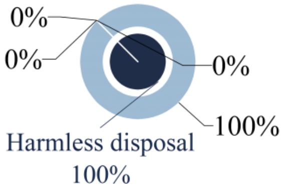

Tianjin-2020:

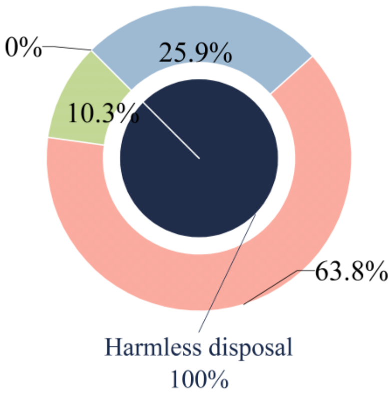

Guangan-2020:

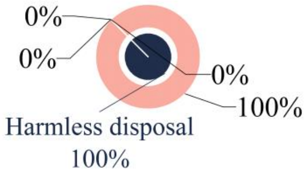

Guangzhou-2020:

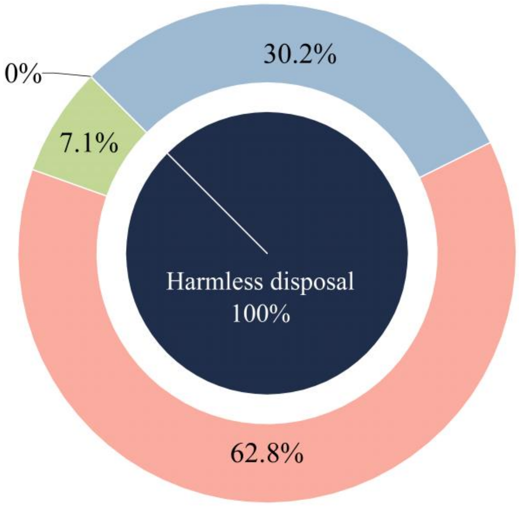

Kunming-2020:

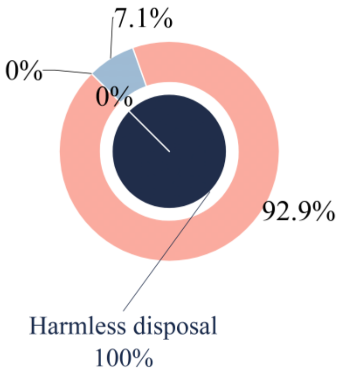

Wuhan-2020:

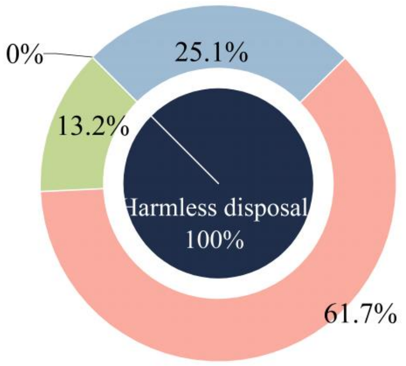

Shenzhen-2020:

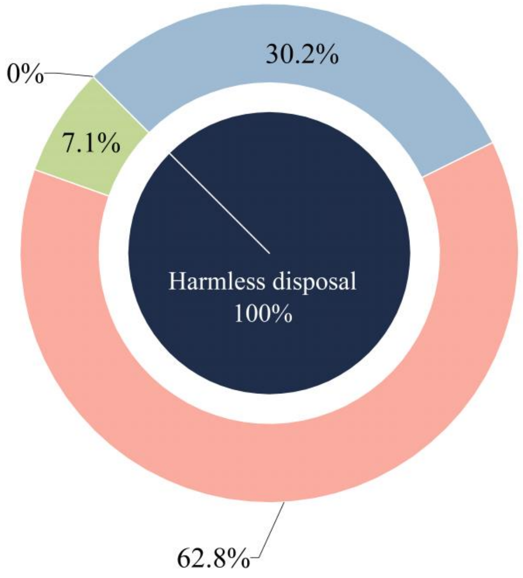

Baiyin-2020:

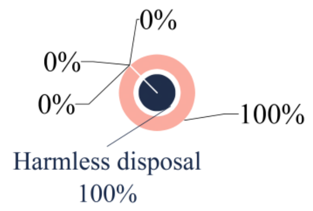

Xian-2020:

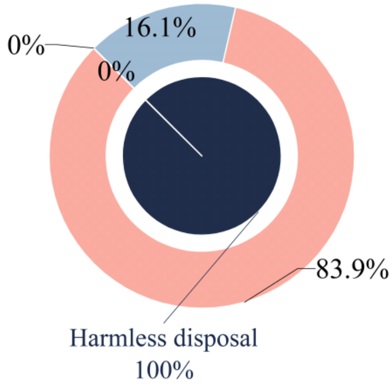

Chongqing-2020:

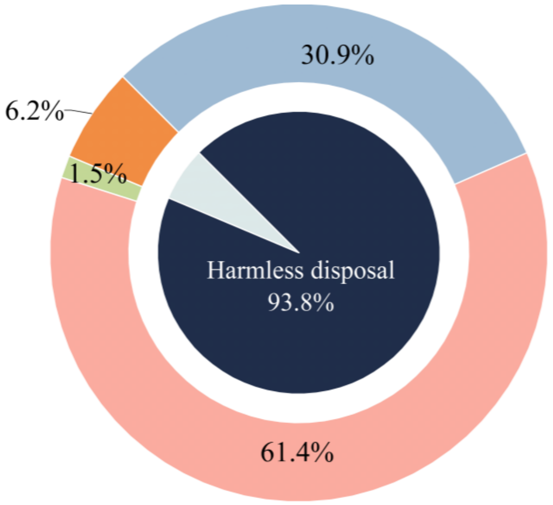

Changsha-2020

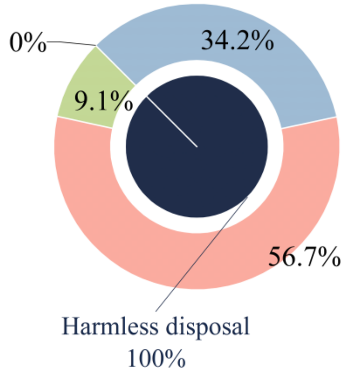

Aksu prefecture-2020:

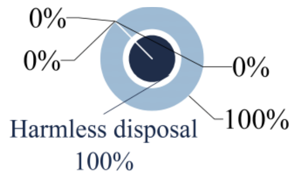

Anshan-2020:

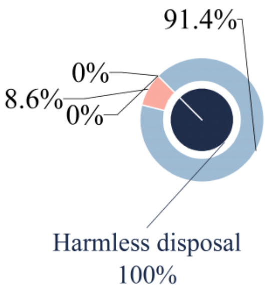

Supplement: Document S2. Article plus supplemental information [file mmc2.pdf]
